# Bioinformatic Analyses of the Ataxin-2 Family Since Algae Emphasize Its Small Isoforms, Large Chimerisms, and the Importance of Human Exon 1B as Target of Therapies to Prevent Neurodegeneration

**DOI:** 10.3390/ijms27031499

**Published:** 2026-02-03

**Authors:** Georg W. J. Auburger, Jana Key, Suzana Gispert, Isabel Lastres-Becker, Luis-Enrique Almaguer-Mederos, Carole Bassa, Antonius Auburger, Georg Auburger, Aleksandar Arsovic, Thomas Deller, Nesli-Ece Sen

**Affiliations:** 1 Institute for Clinical Neuroanatomy, Faculty of Medicine, Goethe University, 60590 Frankfurt am Main, Germany; t.deller@em.uni-frankfurt.de; 2 Experimental Neurology, Clinic of Neurology, Faculty of Medicine, Goethe University, 60590 Frankfurt am Main, Germany; jana.key88@gmail.com (J.K.); gispert-sanchez@em.uni-frankfurt.de (S.G.); ilbecker@iib.uam.es (I.L.-B.); lalmaguermederos@gmail.com (L.-E.A.-M.); arsovicalexandar@gmail.com (A.A.); 3 Institute of Neurophysiology, Neuroscience Center, Goethe University, 60590 Frankfurt am Main, Germany; 4 Department of Biochemistry, Medical College, Autonomous University of Madrid (UAM), 28029 Madrid, Spain; 5 Instituto de Investigación Sanitaria La Paz (IdiPaz), 28029 Madrid, Spain; 6 Instituto de Investigaciones Biomédicas Sols-Morreale (CSIC-UAM), 28029 Madrid, Spain; 7 Centro de Investigación Biomédica en Red, Área Enfermedades Neurodegenerativas, CIBERNED, Instituto de Salud Carlos III, 28031 Madrid, Spain; 8 genOway, F-69362, 69007 Lyon, France; bassa@genoway.com; 9 Clarity Labs, Sydney, NSW 2210, Australia; toniauburger@gmail.com; 10 AKTORmed, 93073 Neutraubling, Germany; auburger.g@gmail.com; 11 Department of Molecular and Cellular Biology, Faculty of Sciences III, University of Geneva, 1211 Geneva 4, Switzerland

**Keywords:** evolution, endosymbiosis, terrestrialization, gene duplication, mitochondrial targeting sequence, polyglutamine neurotoxicity, antisense oligonucleotides, polyAla repeat, LSm-fold, unfolded protein response

## Abstract

Polyglutamine expansion in Ataxin-2 (ATXN2) is responsible for rare, dominantly inherited Spinocerebellar Ataxia type 2 (SCA2). Together with its paralog Ataxin-2-like (ATXN2L), both proteins have received much interest, since the deletion of their yeast and fly orthologs alleviates TDP-43-triggered neurotoxicity in Amyotrophic Lateral Sclerosis models. Their typical structure across evolution combines LSm with LSm-Associated Domains and a PAM2 motif. To understand the physiological regulation and functions of Ataxin-2 homologs, the phylogenesis of sequences was analyzed. Human ATXN2 harbors multiple alternative start codons, e.g., from an intrinsically disordered sequence (IDR) present since armadillo, or from the polyQ sequence that arose since amphibians, or from the LSm domain since primitive eukaryotes. Multiple smaller isoforms also exist across the C-terminus. Therapeutic knockdown of polyQ expansions in human ATXN2 should selectively target exon 1B. PolyQ repeats developed repeatedly, usually framed and often interrupted by (poly)Pro, originally near PAM2. The LSmAD sequence appeared in algae as the characteristic Ataxin-2 feature with strong conservation. Frequently, Ataxin-2 has added domains, likely due to transcriptional readthrough of neighbor genes during cell stress. These chimerisms show enrichment of rRNA processing; nutrient store mobilization; membrane strengthening via lipid, protein, and glycosylated components; and cell protrusions. Thus, any mutation of Ataxin-2 has complex effects, also affecting membrane resilience.

## 1. Introduction

Ataxin-2 (human gene symbol *ATXN2*, protein symbol ATXN2) was discovered as the disease protein responsible for Spinocerebellar Ataxia type 2 (SCA2) [1,2,3] and became widely known because its deletion mitigates the disease progression of TDP-43-triggered Amyotrophic Lateral Sclerosis (ALS) or Fronto-Temporal Dementia (FTD) [4,5] and perhaps also a Tau-triggered form of Parkinson-plus syndrome that was named Progressive Supranuclear Palsy (PSP) [6,7,8,9]. SCA2 is a rare neurodegenerative process with autosomal dominant inheritance, starting in the spinal cord, brainstem, and cerebellum and leading to a typical combination of muscle cramps, deficient locomotor coordination, intention tremor, and slowed saccadic eye movements, so that patients over the course of 6–25 years become wheelchair-bound, bedridden, and die (usually from swallowing problems) [10,11,12,13,14]. The ages of clinical manifestation occur earlier in successive generations of a pedigree, and the molecular cause of this “anticipation of onset age” is the expansion of a polyglutamine (polyQ) repeat domain near the N-terminus of ATXN2 [15,16,17]. In most healthy individuals worldwide, this domain would contain 22Q, normally encoded by (CAG)_8_-CAA-(CAG)_4_-CAA-(CAG)_8_ [18]. The CAA interruptions in this repeat stabilize the length during genome replication [19], and once they are lost, the DNA polymerase will have difficulty copying the exact length, leading to somatic mosaicism, anticipation over generations, and eventually the appearance of a pure CAG repeat of excessive size that causes neurotoxicity with accumulation of the encoded polyQ expansion [20,21,22,23]. It is reasonable to assume that any expansion beyond 23Q contributes to disease risk, but a controversy exists as to whether this can be formally proven for sizes > 24 or >27. Clearly, at intermediate sizes of 31Q to 33Q, this neurotoxicity becomes relevant in a polygenic manner, affecting only the most vulnerable neurons (spinal and cortical motor neurons in ALS and FTD, respectively, or the nigrostriatal dopaminergic pathway in Parkinson’s disease and PSP) [4,24,25,26,27,28,29,30,31]. From the threshold of 34Q upward, the neurotoxicity is strong enough to act in a monogenic manner with increasing severity in the multi-system neurodegeneration known as SCA2, either with spinocerebellar deficits upon adult onset [32,33,34] or with developmental delay and seizures upon pediatric onset caused by very long polyQ domains [35]. Expansions where one CAA interruption is still present have been reported several times to be associated with a clinical picture of Parkinson’s disease or ALS [36,37,38,39,40]. Preliminary data, mainly from animal models, also suggest that ATXN2 modifies the disease course of other spinocerebellar ataxias (SCA1/SCA3), adult motor neuron disease (ALS/FTD), and tauopathy-associated Parkinsonism [4,5,6,7,9,16,28,41,42,43,44,45,46,47].

However, it is completely unclear how ATXN2 exerts this role as an important modifier protein for neurodegenerative processes. ATXN2 localizes normally to the cytoplasm. A minor quantity of ATXN2 associates with the Golgi apparatus [48,49,50], modulates endoplasmic reticulum (ER) dynamics [51], and associates with the endocytosis complex at the plasma membrane [52,53], where it mediates the fast recycling of the Notch receptor [54]. Thus, the role of ATXN2 in membrane dynamics was documented early on but was not investigated intensely, while an additional role of ATXN2 for RNA has caught most of the attention. The main quantity of ATXN2 is present at the rough ER, where translation of membrane proteins and secreted proteins occurs [55], and associates with polyribosomes [56]. Some ATXN2 was reported to interact with actin and fimbrin [57,58,59], and furthermore, some evidence in favor of microtubule association was published [8,60,61,62,63], so a role of ATXN2 for cell protrusions is apparent. The diffuse distribution of ATXN2 throughout the cytoplasm changes dramatically in periods of cell stress, when it relocalizes with damaged RNA to the cytoplasmic stress granules, where RNA surveillance and triage occur [64,65,66]. Oxidative stress, in particular, is known to cause the maximal recruitment of RNA and translation machinery to stress granules, and one report suggested that ATXN2 influences the fate of superfluous mitochondria as the main source of oxidative stress [67], a notion that is strengthened by evidence that ATXN2 controls the expression levels of PINK1 as the protein kinase, which phosphorylates PARKIN to initiate the autophagic elimination of mitochondria, and that ATXN2 associates with PARKIN as the ubiquitin ligase, which tags mitochondrial outer membrane proteins as targets of removal [68,69,70].

It is still unclear how these localizations reflect ATXN2 functions and interactions in specific pathways. As novel insights, this study analyzes Ataxin-2 orthologs (for brevity, this review will use the term Ataxin-2 as a generic term to include all homologs with similar structure throughout evolution) to document isoforms and the evolution of different domains and regions, thus elucidating their regulation and functional context. The discovery of extra domains that were integrated with Ataxin-2 orthologs into chimeric proteins during evolution, and their explanation as transcriptional readthrough across neighbor genes, triggers the notion that mutations in Ataxin-2 have complex effects on its isoforms and also on the function of genomic neighbors.

The bioinformatic efforts in the current manuscript must take into account how multi-domain proteins such as Ataxin-2 became necessary, and how extra-domains are still getting added even in vertebrates. Recent studies in *Euglena gracilis* microalgae observed that cells with additional energy from endosymbionts can increase their cytoplasmic volume and show highly flexible cell shapes [71]. However, the diffusion of substrates, cofactors, and proteins becomes insufficient for efficient metabolism in such larger cells. This causes selection pressure that favors assembling multi-component complexes non-covalently, or even better, placing several domains from single pathways within one protein, rather than relying on diffusion. In this manner, the assembly of the LSm domain, LSmAD, PAM2 motif, and IDRs into one large protein would maximize the throughput in a chain of reactions that are important for eukaryotic life. The classical domain combination that constitutes Ataxin-2 is clearly able to respond to oxidative stress and to manage some form of damage control, for RNAs and possibly other vulnerable compounds.

Importantly, diverse eukaryotic species live in different niches, so they had to adapt their metabolic processes further. Therefore, additional domains were appended to the core features of Ataxin-2 in a minority of organisms. The spectrum of variation between these additional domains and functions reflects changes in stressors, as well as alternative stress responses among eukaryotic phyla. In our view, available data on this “tinkering of nature” [72,73] can be exploited to understand the core and accessory mutation effects not only for Ataxin-2 but for any poorly understood protein. The compilation of such additional domains and the analysis of statistically enriched features among them might contribute to understanding how nature modifies and optimizes the core role of an enigmatic protein like Ataxin-2. This approach could elucidate how Ataxin-2 impacts several overlapping pathways. The wealth of sequencing data on species from all kingdoms of life, which is available in databases such as UniProt/UniParc, NCBI/GenBank, and EMBL, can provide many novel insights into physiological connections between metabolic responses and the specific abiotic stressors in each habitat. This approach consumes an enormous amount of time when performed manually but should be amenable to automation and is applicable to many scientific questions.

Overall, this study aimed to understand the modular composition of Ataxin-2 and derive recommendations on which parts are optimal targets for therapy.

## 2. Results

### 2.1. Overall Structure of Ataxin-2 Family Members Across Evolution

**Figure 1:**
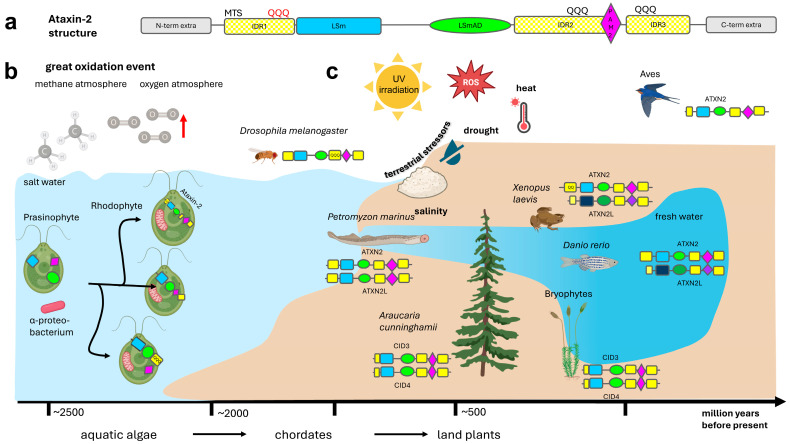
Schematic of the evolution of the Ataxin-2 protein family. (**a**) The structure of all members of the Ataxin-2 protein family contains an intrinsically disordered region 1 (IDR1, in yellow color) near the N-terminus, which may contain a polyQ domain, e.g., in primates, then an LSm domain with 5 beta-barrels flanked by an alpha-helix on each side (in blue color), after a spacer sequence followed by an LSm-Associated-Domain (LSmAD, in green color) with some alpha-helix structures, then IDR2 sometimes containing a polyQ domain, e.g., in insects, a linear PAM2 motif (purple color), IDR3 sometimes containing a polyQ domain, e.g., in mucorales fungi, and variant C-terminal sequences. The IDRs show poor sequence conservation across evolution (illustrated by hatched color). In less than 5% of Ataxin-2 orthologs, there are extra domains at the N-terminus or C-terminus (grey symbols) that are mostly functioning in membrane stress resistance or rRNA processing. (**b**) Ataxin-2 orthologs are not present in eubacteria, archebacteria, or glaucophytes that evolved before the great oxidation event in the atmosphere. Ataxin-2 orthologs are documented in their typical and complete structure since rhodophytes that exploited rising oxygen levels via endosymbiotic mitochondria and chloroplasts, while defending against oxidative stress by carotenoid pigments. (**c**) Whereas one gene copy of *Ataxin-2* is sufficient in insects and nematodes, and is again sufficient for flying birds, during the process of terrestrialization two independent gene duplications were conserved: from conifers to mosses and flowering plants (named as CID3/CID4) on the one hand, and from chordate fish to ray-finned fish, amphibia, reptiles until mammals (named as ATXN2/ATXN2L) on the other hand. PolyQ domains near the N-terminus or near the C-terminus already existed in algae, are found frequently upstream from the PAM2 motif in insects, and have expanded steadily in the unusually long N-terminus of ATXN2 since amphibians. Created in BioRender. Key, J. (https://BioRender.com/8073e0f) (accessed on 8 December 2025) is licensed under CC BY 4.0.

The multi-domain protein Ataxin-2 family has three conserved sequence motifs in a defined order [74,75,76]. Their structures are dominated by the conspicuous LSm fold (see https://alphafold.ebi.ac.uk/entry/A0A370Q1D4, (accessed on 1 December 2025) or https://www.rcsb.org/structure/AF_AFA0A044URM1F1 (accessed on 1 December 2025), with the LSm-associated domain (LSmAD) also standing out in alpha-fold predictions, while most remaining Ataxin-2 is characterized by intrinsically disordered regions (IDRs), where the linear PAM2 motif is usually embedded within the C-terminal half. The three successive domains—surrounded by IDRs and, in less than 5% of species, by extra domains of known function—are shown schematically in Figure 1a and are meticulously assessed in text paragraphs below.

Many functional insights are known for two of the three highly conserved domains, but the third, least-studied domain was found to be characteristic of Ataxin-2:

(1) Firstly, just after the ancestral N-terminus, the Like-SM (LSm) fold has a typical structure of small five-strand anti-parallel beta sheets (β1, β2, β3, β4, β5), with SH3-type barrel tertiary structure. The ancestral quaternary structure is characterized by assembly into an LSm hexameric or heptameric ring, with U-rich RNA oligonucleotides binding inside the LSm torus lumen. Overall, the LSm domain in human ATXN2 and ATXN2L (synthetic full-length entries UniProt Q99700 and Q8WWM7) comprises 78 amino acid residues. LSm domains have descended from bacterial proteins like *Escherichia coli* Hfq and YlxS, as well as archaeal Sm1/Sm2, which serve as RNA chaperones and in ribosomal pathways [77,78]. In eukaryotes, various Sm proteins in the nucleus form a heteroheptameric ring that is crucial for intron splicing [79]. Like-Sm proteins were subsequently also described in the cytoplasm, where LSM2-16 combine the RNA-binding sequence with a methyl-transferase domain [80,81], whereas Ataxin-2 combines the RNA-binding sequence with the LSmAD and the PAM2 motif [76,82]. Our datamining effort confirmed that the LSm sequence of Ataxin-2 has relevant similarity to the LSm domains of LSM2-16, so BlastP searches frequently confuse different families, and the sequence variability of the LSm domain is so strong in low organisms that current InterPro and Pfam algorithms fail to detect it in approximately one third of Ataxin-2 orthologs. The LSm/LSmAD region in Ataxin-2 binds to the RNA helicase DDX6 [64]. Together with the RNA helicase DDX6 and the LSm-containing factor LSM12, Ataxin-2 was found to influence circadian post-transcriptional regulation and olfactory habituation in fly neurons [83,84]. It is therefore important to note that an RNA helicase domain was chimerically added to Ataxin-2 orthologs in several species (see below for individual protein database entries).
(2) Secondly, after a disordered bridge region of, usually, 50–60 amino acids, the LSmAD sequence stands out with a predicted alpha-fold structure. However, experimental analysis showed only a modest presence of α-helical structural elements, with a considerable degree of flexibility, devoid of tertiary structure and without RNA binding capacity [77]. Human Ataxin-2 LSmAD sequence (amino acids 409–477 in the synthetic full-length UniProt entry Q99700) contains a putative clathrin-mediated trans-Golgi signal (residues 414–416) and an ER exit signal (residues 426–428). Indeed, experimental analysis confirmed that the deletion of 42 residues within LSmAD causes Golgi dispersion [50]. Overall, the ancient protein module comprising LSm and LSmAD with their connecting bridge sequences extends across some 250 amino acid residues in a very stable size across evolution, while most length variability of Ataxin-2 orthologs is due to IDR composition and length across the C-terminal half and sometimes in short N-terminal regions. Here, it is important to note that our datamining effort found practically all LSmAD-containing sequences to represent Ataxin-2 orthologs, so the extremely well-conserved LSmAD domain is the unique characteristic feature of the Ataxin-2 family and is perfectly suited for the BlastP search for orthologs.
(3) Thirdly, the 14-residues short linear motif known as PAM2 was named Poly(A)-binding protein interacting Motif 2. It connects to an MLLE sequence in the PABP C-terminus (also known as CTC, short for carboxy-terminal conserved domain), in dependence on nearby phosphorylation sites [62,78,79,80,81]. In plants, the PAM2 motif extends over 19 residues that contain a tandem duplicate of the core sequence [76]. Interestingly, its location is always outside globular domains [82], at approximately three-fifths of the protein length. It clearly functions to interact with mRNA 3′ tails, and it exists as a component of over a dozen different eukaryotic proteins [82], several of which are known for their regulation of mRNA translation versus decay [79]. These protein families include PAIP1/2, LARP4, eRF3/GSPT1/2, TTC3, USP10, PAN3, GW182, Tob1/2, and other factors, so our datamining effort found its usefulness for Ataxin-2 ortholog searches to be limited. Particularly in low organisms, the sequence variability of this motif makes its automated recognition by current InterPro and Pfam algorithms doubtful. The PAM2 motif was shown to prevent the phase separation of Ataxin-2 in cellular growth periods, while it localizes to the translation apparatus at the rough ER, promoting the relocation of Ataxin-2 to stress granules after cellular damage [55,56,83].

### 2.2. Compilation of Ataxin-2 Family Protein Sequences Until Excavata, Amoebozoa, and Algae

Conservation of Ataxin-2 sequences across evolution was used to judge the relevance of each domain and feature for physiology and pathology. Using the BlastP algorithm to search UniProt–UniParc, NCBI, and EMBL databases for ATXN2 homologs, the longest or otherwise relevant isoforms for many organisms are compiled in Table S1, but it was impossible in the current study to compile all isoforms and assess their genomic coding sequences. Separate tabs in this table show either an overview of the longest orthologs (Table S1A), or specifically the N-terminal intrinsically disordered domain 1 (IDR1) before the polyQ domain (Table S1B); sequences across the polyQ domain with its flanking residues and interrupting residues (Table S1C); the IDR1 sequences around and after the polyQ domain (Table S1D); structured sequences around and after the LSm motif (Table S1E); structured sequences around the LSmAD motif (Table S1F); the intrinsically disordered domain 2 (IDR2, Table S1G); sequences across the PAM2 motif within IDR2 (Table S1H); and ordered sequences before and after IDR3 with C-terminal variability (Table S1I). Some ATXN2L orthologs are compiled in Table S2. While this compilation cannot be exhaustive, it covers the main classes of eukaryotic organisms.

It is difficult to pinpoint the origin of Ataxin-2, given that precursors of Ataxin-2 in the databases cannot reliably be distinguished from fragmented Ataxin-2 due to sequencing problems, proteolysis, or isoforms. Furthermore, the absence of Ataxin-2 orthologs from species or organism classes may simply be due to incomplete sequence documentation, so only crude conclusions are possible. But as the prime result, Ataxin-2 orthologs were clearly identified in all eukaryotic kingdoms until amoebozoa, protozoa, and algae, but not in archaebacteria or eubacteria. Details are provided in the following paragraphs of this chapter.

Figure 1b depicts the evolution and domain structure of Ataxin-2 proteins in all kingdoms of life. Among amorphea–amoebozoa, Ataxin-2 homologs as in UniProt entries A0A151ZGE7 and Q55DE7 with 900–1100 aa in *Dictyostelium* are already complete with N-terminal polyQ stretches, LSm, LsmAD, and a typical PAM2 motif recognized by the InterPro or Pfam algorithms.

Among protozoa, Ataxin-2 homologs like A0A1Z5KDA2 and A0A9N8E681 with 700–900 aa in stramenopiles have this multi-domain structure and C-terminal polyQ stretches. It is noteworthy that the most ancient among them have chloroplasts surrounded by four membranes as a testimony to secondary endosymbiosis [84]. In phaeophytes (brown algae), as a photosynthetic class among stramenopiles, UniProt–UniParc entries A0A6H5L901, D7FVW6, and D7G1N4 in *Ectocarpus* species represent typical Ataxin-2 orthologs with short polyQ stretches upstream from LSm.

Interestingly, A0A0G4IZN6 with 400 aa in rhizaria contains a structured sequence not recognized as LSm by current algorithms, then LSmAD, IDRs with PAM2 embedded (PAM2 sequence takpkLNANAKTFT-MSAAAKEFV), but no polyQ stretch.

In naegleria from the excavata/discoba-schizopyrenida branch (that alternates between amoeboid and flagellate stages, having mitochondria with discoid cristae similar to *Euglenozoa* [85]), the UniProt entries A0A6A5CC11, A0AA88H5V6, and D2V0R5 with about 900 aa represent complete Ataxin-2 orthologs with N-terminal polyQ stretches.

In the closely related euglenozoan branch (the only photosynthetic branch among the excavata), smaller complete orthologs are documented. A0A0L1KIJ6 with >800 aa in *Perkinsela* shows a potential LSm coexisting with LSmAD and a primitive PAM2 motif (LNPNATAFLP) without polyQ stretches; A4H699 or A4HUM3 or Q4QHA3 in *Leishmania* (potential PAM2 precursor core sequence PNPSATPFVP), A0A1X0NQC5 in *Trypanosoma* (potential PAM2 core FNPAATPYTP), W6KMY5 in *Phytomonas* (potential PAM2 core PNPAAAPFVP), A0A0M9FU89 in *Leptomonas* (potential PAM2 core PNPSATPFVP), all with >500 aa, show an LSm and LSmAD coexisting with a C-terminus that contains several polyQ stretches. Trypanosomal A0A061J3B7 with 279 aa, and A0A0S4IS39 with 213 amino acids, show LSm and LSmAD coexisting without further elements. Given that trypanosomes were reported to have exchanged genetic material with green algae and other archaeplastida [86,87], orthologs of Ataxin-2 were studied in detail among algae.

Ataxin-2 orthologs are also documented among alveolata (A0A813ABV5 with >1100 aa that contain an extra 2-hydroxyacid dehydrogenase domain, with homology to bacterial SerA protein, which is essential for the biosynthesis of L-serine as precursor of phospholipids and sphingolipids). Among cryptista, one protein with significant LSmAD homology is documented (L1I7C3 with 522 aa).

In contrast, Ataxin-2 homologs appeared absent from the diplomonad and parabasalid–metamonad branches of excavata protists, which lack mitochondria, so these protists depend on nutrients and energy from hosts. In the monocercomonoides species from excavata, endosymbiont mitochondria were completely lost again [88], and an Ataxin-2 homolog could not be detected, while the microsporidia that lost mitochondria but retained mitosomes with FeS-metabolism-dependent oxidative stress [89] did preserve the presence of Ataxin-2 orthologs (e.g., A0A4Q9L9Z0 and A0A086J3B3). These findings support the concept that Ataxin-2 is needed to protect cells from the oxidative stress emanating from endosymbionts.

Among algae, many entries for Ataxin-2 orthologs exist from chlorophytes (green algae) with well above 1000 aa (e.g., A0A8J4CZB7 or A0A383VPF7) that contain LSm, LSmAD, polyQ stretches shortly after LSmAD, and typical PAM2 motifs embedded in the long C-terminal IDRs, as well as a short N-terminal IDR, in close structural and length similarity to human ATXN2L and ATXN2. In the particularly interesting green algae *Cymbomonas tetramitiformis*, whose ancestors are thought to have played a crucial role in the primary endosymbiosis of plastids [90], A0AAE0L4G1 is documented as a typical Ataxin-2 ortholog of 668 aa without polyQ stretch. Similarly, among archaeplastida–rhodophytes (red algae), the three entries A0A1X6NXA5, A0A5J4YT85, and A0A2V3IVX2 (noteworthy for a C-terminal uninterrupted 30Q repeat) represent Ataxin-2 orthologs.

However, no Ataxin-2 homologs were detected in glaucophytes (green-blue algae, which have plastids surrounded by an inner membrane containing a remaining peptidoglycan layer, suggesting they are still synthesized by plastids as by ancestral cyanobacteria; glaucophytes have mitochondria with the typical eukaryotic flat cristae surrounded by a double membrane and use asexual reproduction). They are considered the basal archaeplastida that engulfed cyanobacteria in an act of primary endosymbiosis within a terrestrial freshwater ecosystem [91,92,93,94,95]. Therefore, Ataxin-2 orthologs apparently were not needed for the engulfment of mitochondria or plastids. Instead, the appearance of Ataxin-2 might be associated with the gained ability to synthesize robust inner membranes by eukaryotes rather than by plastids. Apparent absence of Ataxin-2 homologs was also observed in the mesostigma branch of green algae, which contains primitive plate-like chloroplasts, lacking a periplastidal ER [96,97], and was therefore placed at the basis of divergence between chlorophyta (green algae) and streptophyta (land plants). This finding agrees with the notion that Ataxin-2 presence correlates with eukaryotic synthesis of organellar membranes.

Overall, the appearance of Ataxin-2 ancestors occurred only after glaucophytes had developed the endosymbiosis of mitochondria and after primary endosymbiosis of plastids (Figure 1b). Ataxin-2 ancestors are only documented since rhodophytes learnt to cope with the oxidative stress due to sun exposure and mitochondria/chloroplast presence [98]. Rhodophytes still have chloroplasts without external ER or unstacked (stroma) thylakoids, but developed α- and β-carotene (provitamin-A), lutein, and zeaxanthin as new antioxidative pigments [99], reproducing sexually, while lacking centrioles and flagella. Carotenoids play a basic role as membrane reinforcers in view of their rigid conjugated double-bond backbone as phylogenetic precursors of sterols [100,101]. When marine life forms subsequently moved to freshwater habitats and underwent terrestrialization, duplications of the *Ataxin-2* gene occurred (Figure 1c).

### 2.3. Gene Duplication ATXN2/ATXN2L in Animals, and CID3/CID4 in Plants, upon Entering Freshwater and Land

**Figure 2:**
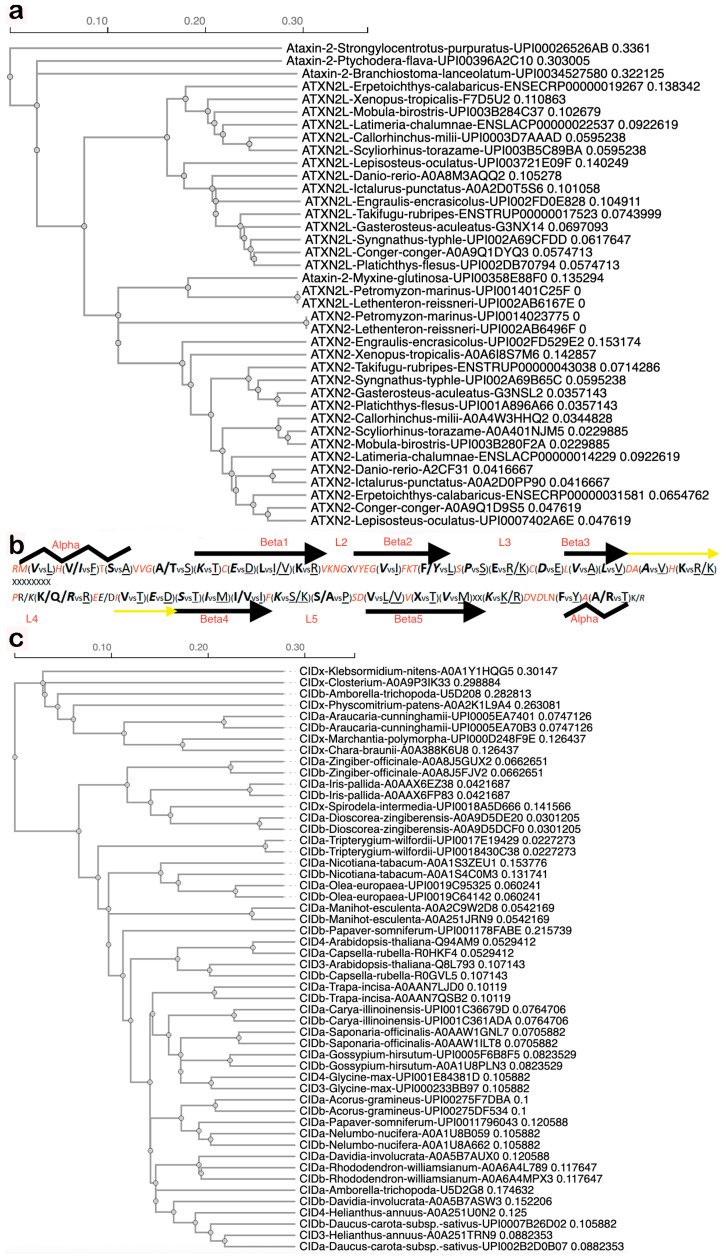
Duplication of the *Ataxin-2* gene resulted in animal transcripts encoding ATXN2 versus ATXN2L, with characteristic differences in sequence and structure, while an independent duplication resulted in plant transcripts encoding CIDa versus CIDb, which co-evolved without consistent distinguishing features. (**a**) Guide tree of LSm domain evolution in fish generated by CLUSTAL Omega 1.2.4 Multiple Sequence Alignment software (accessed on 24 October 2025) shows single copy Ataxin-2 protein sequences (always with species name and database entry ID) in sea urchins, acorn worms, hagfish and early chordates (*Branchiostoma*), while other chordates (*Petromyzon marinus*, *Lethenteron reissneri*) already have two copies documented with point mutations, which remain typical and lead to the divergence of ATXN2 from ATXN2L in all descended species into two separate groups. We use the term Ataxin-2 when an organism has only one copy or to refer to the gene family, while ATXN2 or ATXN2L are used to distinguish between the two copies of a species that are encoded by the duplicated genes. (**b**) Fish **ATXN2** versus ATXN2L sequence specificities, in red color if invariable, in *italics* if conserved in ATXN2 until human, showing the invariable sequences to correspond to linker regions between predicted alpha and beta structures, whereas the 5 beta sheets typical for LSm folds (depicted as arrows) and the surrounding alpha-helices (as zigzag lines) exhibit characteristic signature residues that differentiate **ATXN2** vs. ATXN2L. (**c**) The guide tree of LSm sequences during plant terrestrialization shows single-copy Ataxin-2 orthologs, named CIDx, in green algae, mosses, liverworts, and duckweed, while double-copy Ataxin-2 orthologs (randomly dubbed CIDa or CIDb when there were no criteria to assign them to the CID3 group or the CID4 group) are documented since evergreen conifer trees and the earliest flowering plants that still bear tracheids. While the two copies in each species usually localize together or nearby, a big exception was observed for *Amborella,* which had one primitive copy closely related to *Closterium* algae and one sophisticated copy that exhibited sequences akin to flowering *Rhododendron*.

Datamining with STRING homology searches revealed that the gene duplication giving rise to two copies encoding land animal ATXN2 versus ATXN2L proteins is not only documented from mammals via *Xenopus laevis* to *Danio rerio* but is documented during earlier evolution until lampreys (UPI0014023775 vs. UPI001403D160 in *Petromyzon marinus*, UPI002AB6496F vs. UPI002AB6167E in *Lethenteron reissneri*) (Supplementary Material S1). Lampreys start life as larvae in freshwater, irrespective of whether they later live in coastal sea areas, rivers, or lakes. ATXN2 orthologs among fish species share more similarity with each other than with ATXN2L orthologs (Figure 2a), with specific residues being characteristic of ATXN2 versus ATXN2L (Figure 2b). The Pro vs. Ser difference at the beginning of the L3 sequence within the LSm fold would cause a bend in the peptide chain and possibly result in a beta-turn only in ATXN2 orthologs, whereas the Ser/Ala vs. Pro difference in the center of L5 may cause such a bend or beta-turn only in ATXN2L orthologs (Figure 2b). This suggests that different interactors and functions for ATXN2 versus ATXN2L have evolved. Thus, two copies of Ataxin-2 with differing point mutations are first detected in chordate lampreys as jawless fish that derive from the Devonian period, some 360 million years ago [102]. The gene duplication without distinguishing sequence variants may also be present in other chordates, like hagfish. Indeed, three rounds of genome duplication events are known to have occurred during fish evolution, one of them during the Devonian [103]. With many genes duplicated, chordates were enabled to develop notochords and neural tubes with dorsal versus ventral specifications, which are established via signaling with retinoic acid (a carotenoid vitamin-A derivative) to control homeobox expression and somite formation [104,105,106,107,108,109,110,111], a pathway that establishes anterior–posterior gradients and is particularly a pathway for the development of the hindbrain, cerebellum, and spinal cord, which are affected most strongly in SCA2 and ALS [112].

The question of whether *ATXN2* or *ATXN2L* is the duplicated copy was assessed by genomics. The relevant human chromosome locus on 12q contains the gene order *PPP1CC*–*CCDC63*–*MYL2*–*VHRT*–*CUX2*–*PHETA1*–*SH2B3*–***ATXN2***–*U7*–*BRAP*–*ACAD10*–*ALDH2*–*MAPKAPK5*–*TMEM116*–*ERP29*–*NAA25* (underlined genes share the same transcriptional direction; *U7* refers to the 63 bp uridine-rich non-coding snRNA ENST00000607576.1, a potential binding target of Sm domains, which is positioned on the opposite strand downstream from polyQ-encoding exon 1 and upstream from exon 2 of human *ATXN2* as the start of the LSm sequence encoding exon cluster), whereas the locus on 16p contains the gene order *SULT1A1*–*NPIPB8*–*EIF3C*–*NPIPB9*–***ATXN2L***–*TUFM*–*SH2B1*–*ATP2A1*–*RABEP2*–*CD19*, according to the human genome annotation in the UCSC genome browser. The synteny of *ATXN2* with *SH2B3*, and of *ATXN2L* with *SH2B1*, is conserved since *Nile tilapia* fish. Before the gene duplication event, in fish such as *Callorhinchus milii* (elephant shark) or *Oryzias latipes* (medaka), the genome contains only the *SH2B3*–*ATXN2*–*BRAP* locus. It is tempting to speculate about a functional interaction of ATXN2 with SH2B3 and ATXN2L with SH2B1, given that all these proteins contain proline-rich motifs and associate with GRB2 during PI3K/AKT growth signaling at neuronal membranes [53,113,114,115,116]. Such a potential competition for GRB2 would be a relatively recent development during evolution, given that *SH2B3* is not a neighbor gene of *ATXN2* in the genomes of lancelet fish, *D. melanogaster* flies, or *C. elegans* nematodes, and is therefore not crucial for the ancient functions of Ataxin-2. However, it is conceivable that *SH2B3*, *ATXN2*, and *BRAP* are jointly relevant for human diseases such as SCA2, ALS, cancer, and longevity, either via their gene variants in linkage disequilibrium or via protein interactions [29,117,118]. Overall, *ATXN2* clearly evolved from ancient Ataxin-2 in its original genomic context, while *ATXN2L* represents the duplicated copy in a new genomic environment.

ATXN2L is thought to be missing in birds. However, non-flying but feather-bearing kiwi birds in northern New Zealand have ATXN2 and ATXN2L entries in UniProt (*Apteryx mantelli* A0ABM4FEG3 and A0ABM4G1B9). Apparently, ATXN2L got lost before the evolution of flight, because ostriches, emu, and other kiwi species do not record ATXN2L sequences. It is known that large segmental deletions occurred when the macroevolution of avian genomes favored constrained size and became relatively static [119].

Among plants, two Ataxin-2 homologous gene copy translation products are not only documented in eudicot brassicales such as *Arabidopsis thaliana* (Q94AM9 vs. Q8L793) and *Capsella rubella* (R0HKF4 vs. R0GVL5) and other flowering land plants but also in paleodicots/ANA-grade angiosperms such as amborellales (U5D2G8 vs. U5D208 in *Amborella trichopoda*) and in gymnosperms such as conifers–araucariales (UPI0005EA7401 vs. UPI0005EA70B3 in *Araucaria cunninghamii*) (Supplementary Material S2). The ancestral angiosperms, like amborellales as well as gymnosperms, underwent enormous diversification after two whole genome duplication events since 319 million years ago during the late carboniferous period [120], which was possibly the ancestral event where CID3/CID4 appeared. While the Ataxin-2 orthologs in conifers still have >800 amino acids, similar to chlorophyte Ataxin-2 orthologs, CID3 and CID4 in flowering plants show a length of around 600 residues, consistent with the notion that some of their stress resistance properties were lost during evolution to flowering plants due to unusually short IDRs at either end of the proteins.

Conifers were the first trees among land plants. They are still the dominating plant species, e.g., in the taiga of Siberia, where they show exceptional resilience to winters with temperatures below the freezing point, managing to stay evergreen. Their cold hardiness involves the sensing of low temperatures and a short photoperiod to trigger molecular adaptations, such as (i) improved membrane fluidity, with the development of epicuticular wax (composed mainly of nonacosane, which is used as a component of paraffin, as a 29-carbon straight-chain alkane that is derived from very-long-chain fatty acids [121]); (ii) the mobilization of solutes such as glycogen to lower the freezing point and minimize cold shock of vulnerable RNA; and (iii) the downregulation of photosynthesis and cell growth in parallel to upregulated expression of cold-induced proteins [122].

Importantly, there are no specific residues that are characteristic of either CID3 or CID4. Instead, both proteins encoded by duplicated gene copies in each organism were more similar to each other than to the orthologs in other species (Figure 2c). Thus, they appear to have co-evolved in different organisms, adapting similarly to the changes of interactor molecules in dependence on various ecological niches. Apparently, both were conserved and remain expressed because a higher gene dosage was beneficial, but they did not assume different roles.

A curious exception to the high similarity of both Ataxin-2 orthologs within each species is observed in amborellales, where the protein encoded by one gene copy has a sequence with homology to flowering land plants. Amborellales are known to undergo extensive horizontal gene transfer from neighboring plants [123], and this might provide an explanation.

Overall, events that duplicated the *Ataxin-2* gene occurred twice during macroevolution while moving out of marine life, with both duplications becoming conserved with independent fates. On the one hand, one event among chordates of shallow waters was inherited by land animals, resulting in ATXN2 versus ATXN2L proteins acquiring separate functions reflected by different protein lengths. On the other hand, another *Ataxin-2* gene duplication occurred among conifers as early tree forms with unusual stress resilience, resulting in CID3 versus CID4, which lost some protein length and did not develop consistent distinguishing features. This is compatible with the notion that mechanical stress due to gravitational forces, more sunlight intensity, heat and cold exposure, and oxidative stress made it advantageous to have two gene copies when marine life forms underwent terrestrialization. The fact that flying birds lost the *Ataxin-2* gene duplication might suggest that the higher weights of organismal bodies on land and the mechanical force of waves in shallow water require strengthened cell membranes. In contrast, life-forms that are suspended by buoyant forces in deep ocean water or in the air have less demand on membrane stability.

**Figure 3:**
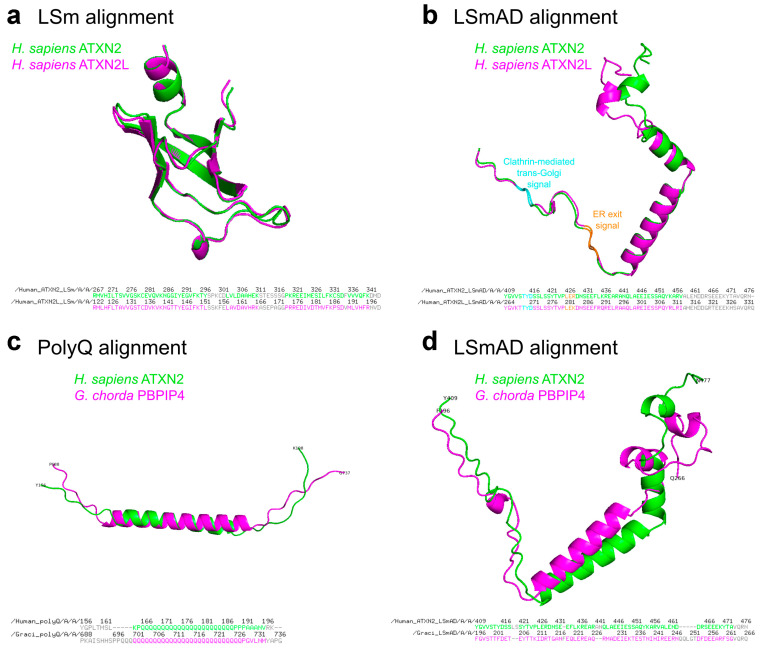
(**a**,**b**) Structural alignment of LSm and LSmAD domains of human ATXN2 and ATXN2L. (**a**) LSm domains of ATXN2 (aa267–344) and ATXN2L (aa122–199) were extracted from the AlphaFold predicted structures (AF-Q99700-F1-v6 and AF-Q8WWM7-F1-v6, respectively) and aligned using PyMOL, version 3.1.0 (accessed on December 9, 2025). The amino acid sequences of the domains and aligned residues are presented below. (**b**) LSmAD domains of ATXN2 (aa409–477) and ATXN2L (aa264–333) were extracted from the AlphaFold predicted structures (AF-Q99700-F1-v6 and AF-Q8WWM7-F1-v6, respectively) and aligned using PyMOL. Clathrin-mediated trans-Golgi signal and ER exit signal within the LSmAD domain are marked in cyan and orange, respectively. The amino acid sequences of the domains and aligned residues are presented below. (**c**,**d**) Structural alignment of polyQ and LSmAD domains of human ATXN2 and its *Gracilariopsis chorda* ortholog, previously named polyadenylate-binding protein-interacting protein 4 (PBPIP4). (**c**) PolyQ domains of human ATXN2 (aa166–188) and *G. chorda* PBPIP4 (aa698–727) were extracted from the AlphaFold predicted structures (AF-Q99700-F1-v6 and AF-A0A2V3IVX2-F1-v6, respectively) and aligned using PyMOL. The amino acid sequences of the domains and aligned residues are presented below. (**d**) LSmAD domains of human ATXN2 (aa409–477) and *G. chorda* PBPIP4 (aa196–266) were extracted from the AlphaFold predicted structures (AF-Q99700-F1-v6 and AF-A0A2V3IVX2-F1-v6, respectively) and aligned using PyMOL. The amino acid sequences of the domains and aligned residues are presented below.

A comparison of the LSm and LSmAD sequences in human ATXN2 versus ATXN2L reveals very similar predicted structures, while pinpointing the putative crucial differences (Figure 3a,b). Additional comparison of the polyQ domain and the LSm domain in the human species versus their first appearance in the red alga *Gracilariopsis chorda* underlines the strong conservation of predicted structures and presumably also their functions (Figure 3c,d).

### 2.4. Genomic Comparison of Exon–Intron Structure for Human and Murine Ataxin-2 Versus Ataxin-2-like

Mapping of the individual exons within genomic sequences (as documented at https://www.ncbi.nlm.nih.gov/gene/6311, accessed on 15 August 2025) shows that human *ATXN2L* exons on chromosome 16 (Supplementary Material S3) stretch across 13,823 bp, with large blocks of non-coding sequences after exon 1, then (with the LSm domain encoded by exons 3–5) large non-coding blocks again after exon 6, then (with LSmAD encoded by exons 7–8) after exon 10, and again (with PAM2 motif encoded by exon 15) after the final exon 24B.

Human *ATXN2* exons on chromosome 12 (previously studied in [124]) have a similar distribution overall (as shown at https://www.ncbi.nlm.nih.gov/gene/11273, accessed on 15 August 2025), but they are found across a >10-fold larger genomic area with 146,713 bp (Supplementary Material S4), suggesting that much more sophisticated control over expression and splicing of exon clusters has become possible. Massive blocks of non-coding sequences are found after exon 1 (where the polyQ repeat is encoded), then (with the LSm domain encoded by exons 3B–5) again after exon 5, a smaller block after exon 6, then (with LSmAD encoded by exons 7–8) smaller blocks each after constitutive exon 9, alternative exon 10, constitutive exon 11, and massive blocks again after exon 14 (encoding IDR2, with the PAM2 motif encoded by exon 16), exon 18A, exon 18B, exon 20, exon 21, and the final exon 25.

The extraordinary similarity of translation starts, aligned exon boundaries (Supplementary Material S5, employing CLUSTAL Omega 1.2.4), and the genomic clustering of several exons around the LSm, around the LSmAD, around the PAM2 motif, and the C-terminal intrinsically disordered sequences may reflect a joint regulation of these exon clusters and separate functions in common for each cluster.

In addition to the ancient conserved and aligned exons, human *ATXN2* has developed alternative splicing at least for exons 1A, 1B, 2, 3A, 10, 21, 23B, and 25 ([124,125,126], UCSC genome browser, accessed on August 8, 2025). Clearly, the regulation of human *ATXN2* appears much more sophisticated than *ATXN2L*, given that it shows many more genomic non-coding control sequences between exons, at least three alternative translation starts ([127], UniProt, NCBI protein database) within the extended N-terminus containing the expanded polyQ stretch, many options for alternative splicing, longer sequences encoded by exons 11 and 13, and a much more variable C-terminus. Further analysis of murine *Atxn2* versus *Atxn2l* confirmed the strong adherence to exon boundaries but also documented several alternatively spliced exons for *Atxn2l* (Supplementary Material S5 [128,129]). The fact that Ataxin-2-like remained relatively unchanged during evolution, while Ataxin-2 underwent numerous adaptations to fine-tune its functions, indicates that human Ataxin-2 has acquired additional features and that SCA2/ALS pathogenesis involves diverse mechanisms, which are not necessarily modelled in lower organisms.

Importantly, the human ATXN2 sequence shows two methionine residues as potential start codons at the initial LSm sequence, and database entries confirm that corresponding isoforms are translated. Similarly, the initial LSm sequence of ATXN2L orthologs contains one or two methionine residues. While such an Ataxin-2 translation start right at LSm was normal in ancient species like *Trypanosoma*, *Perkinsela,* and other protists, and was still producing the main isoforms among reptiles (Table S1A), a short intrinsically disordered N-terminal extension with IDR1 became usual already among protists, fungi, worms, and insects. An extension of the N-terminal IDR1 occurred progressively for ATXN2 homologs since fish, with one methionine at the LSm start being lost. However, there is database evidence that a minor isoform that starts from the LSm domain has always existed from zebrafish to primates (Table S1D).

### 2.5. C-Terminal Fragment Isoforms Are Prominent According to Exon Expression Analyses, and C-Terminal Epitopes Are the Target of Most Current Commercial Antibodies

Interestingly, RNAseq-based analyses of individual exon expression strength in central nervous tissue (as documented at https://www.gtexportal.org/home/gene/ATXN2, accessed on 8 October 2025) reveal that the exons named 22, 23, 24A, and the C-term alternatively spliced final exon in Supplementary Material S6 have the highest scores (in spinal cord, 1.48 and 1.26, respectively, encoding C-terminal intrinsically disordered sequences with seven Met residues). Only about half of these maximum expression levels are observed for exon 15 (containing one Met), with scores at 0.644, and exons 16 (encoding PAM2), 17 (three Met), 18 (two Met), and 19 (three Met), with scores above 0.8. Less than a quarter of these maximum expression levels are found for exons 5–12 (around the LSmAD sequence, including one Met in exon 5 and six Met in exons 9–12), with scores above 0.407. Around a sixth of the maximum expression levels are apparent for exons 2–4 (encoding the LSm fold start, including two Met), with scores between 0.174 and 0.252. Particularly low expression at 3% of maximum levels is seen for exons 1A and 1B (encoding the N-terminal intrinsically disordered sequence and the polyQ domain, including two Met residues), with scores at 0.04.

Also, the GTEx Gene Model based on ENSEMBL-documented transcripts of human *ATXN2* (as documented at https://www.gtexportal.org/home/gene/ATXN2, accessed on 8 October 2025) indicates the existence of many transcripts that comprise only the C-term disordered sequences or begin at sequences downstream from PAM2, from LSmAD, and from LSm. Transcripts encoding only the LSm domain are always combined with LSmAD, but transcripts encoding only the LSmAD domain (entry ENST00000471866.5) or the sequences between LSmAD and PAM2 (ENST00000546483.1) are documented. Overall, only half of the transcripts, including all alternatively spliced transcripts, span the length of the *ATXN2* gene, a tenth spans only the N-terminal parts, while some 40% span across the C-terminal parts. The N-terminal parts seem less relevant than models of 3D-structure around the conspicuous LSm-fold would suggest, while the C-terminal intrinsically disordered sequences have a little-appreciated importance. Therefore, despite the notion of synthetic full-length Ataxin-2 being a multi-domain protein of >1300 residues, cells appear to have retained during evolution a great ability to use most of its modules selectively.

**Figure 4:**
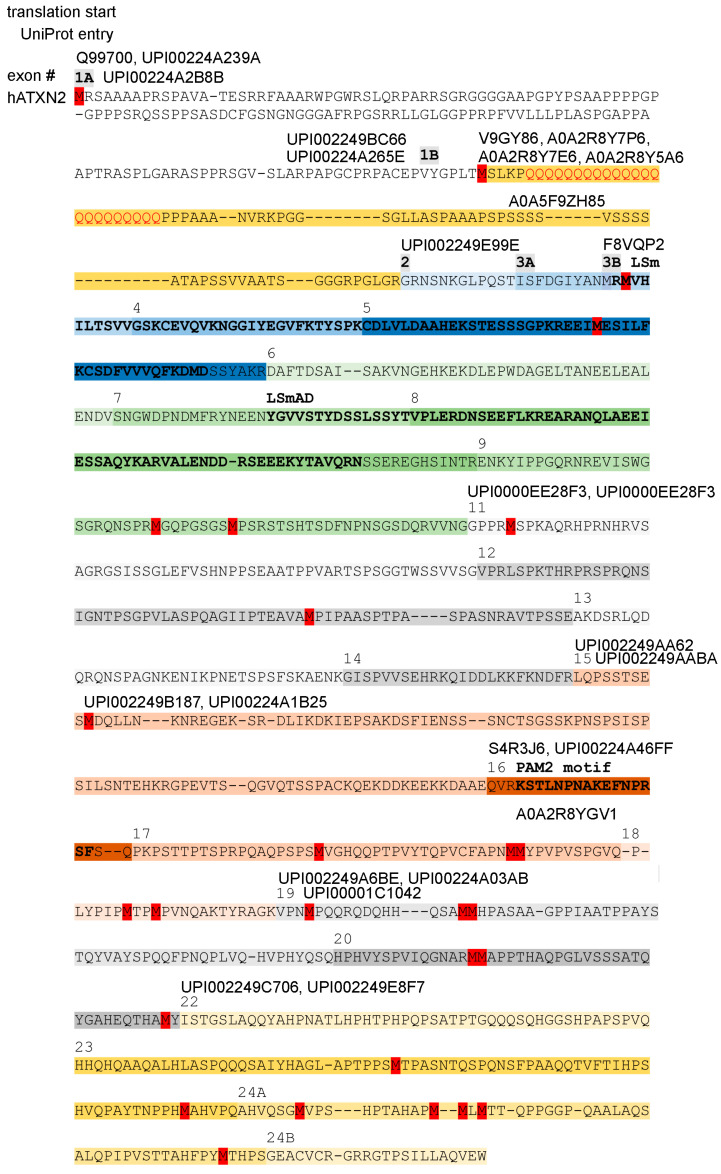
Multiple translation starts (methionine residues in red color), resulting in many long and short isoforms, are documented in protein databases. The scheme shows a version of human ATXN2 without the sequences encoded by alternatively spliced exons 10 and 21, corresponding to the alignment of human ATXN2 versus ATXN2L and murine ATXN2 versus ATXN2L in Supplementary Material S5. The coding of amino acids by individual exons is illustrated by different background color hues, with the exon number (#) indicated above the starting amino acid, highlighting the LSm and LSmAD domain as well as the PAM2 motif above the residues that stand out in bold letters. UniProt–UniParc database entry IDs are placed at different positions above the amino acid thread to indicate where each isoform of ATXN2 converges with the consensus sequence, while ignoring any isoform-specific start codon and downstream divergent amino acids that precede the constitutive ATXN2 fragments. The existence of multiple large and short isoforms of ATXN2 protein is supported by the analysis of mRNA isoforms (Figure S1) and by the molecular weight of bands detected in immunoblots by antibodies that target epitopes in the C-terminal half.

These transcript findings suggest that short protein isoforms may exist with a Met start codon in the center or towards the C-terminal end of human ATXN2, which were previously considered as partial sequence fragments. Indeed, database entry Q99700.2 reflects a translation start from the N-term disordered sequence, NP_002964.4 reflects a start from the polyQ-repeat, and NP_001297052.1 reflects a start from the LSm domain, but there are also entries like XP_059732367.1, where the start lies upstream from LSmAD; EAW97959.1-EAW97960.1-A0A2K6UG79-A0A8C5XNX2-A0A811ZS54-A0A8C4TUK8-A0A3S2PRM8-A0A3Q3N8E0-A0A3Q3FSG1-A0A672RDQ6, where the start lies downstream from LSmAD; A0A2K5CKD3, where the start occurs downstream from LSmAD but the PAM2 motif is selectively excluded; A0A7J8EWQ7/A0A7J7R7F2, where the start lies just upstream from PAM2; or A0A7J7S175-A0A7J8BWL6-A0A5F5Y0Q1-A0A212D9N5-A0A5N4C9F7-A0A5N4C9H8-A0A401TLV8, where the start is located downstream from PAM2. A schematic depiction of the human ATXN2 protein sequence with potential methionine start codons, isoform candidates with UniProt–UniParc database entries, and the encoding exon structure is illustrated in Figure 4.

Commercial antibodies were often raised against full-length overexpressed Ataxin-2. However, in those cases where the companies used a fragment of Ataxin-2 as an immunogen and revealed the epitope residues, a bias towards the more abundantly expressed epitopes downstream from LSmAD is obvious. Few commercial antibodies exist against the expanded polyQ domain, against IDR1 sequences, against the region across LSm or LSmAD, and against the sequences between LSm and LSmAD, while most targeted antibodies detect immunogens downstream from LSmAD (Figure S1 depicts the relevant immunogens relative to the exon structure of isoforms according to GTEx database data). Reporting bias selects figures that represent the 120–130 kDa isoforms, while bands with smaller molecular weight are usually interpreted as unspecific cross-reactivity or are ignored. Thus, there is an important unmet need to study the C-terminal isoforms of ATXN2.

ATXN2 was reported to homo-dimerize or multimerize, and it is important to consider the fact that LSm-containing proteins assemble in homo-hexameric or homo-heptameric rings around RNA. Thus, these expression data would be compatible with a scenario where Ataxin-2 isoforms integrate into assemblies that are dominated by intrinsically disordered domains that promote RNP granule formation during microtubular transport. One sixth of the components would bear the LSm head that can be activated to bind a specific single-stranded AU-rich RNA sequence directly, and then mediate the association of a helicase to linearize the RNA, presumably in a stimulus-dependent manner at the end of transport, e.g., in a synapse before stimulus-dependent translation.

Given that the polyQ-encoding exon accounts for only 3% of maximum expression in the spinal cord, it seems plausible that there is a long temporal delay before polyQ-triggered accumulation and aggregation of N-terminal ATXN2 sequences become prominent in postmitotic neuronal tissue. In a mouse mutant with an ATXN2-Q100 expansion KnockIn, the levels of phosphopeptides from LSm to C-term in non-neuronal tissue are around 10% compared to WT [130], confirming that the expansion can lead to partial loss-of-function effects. However, in postmitotic neural tissue (the spinal cord) at 14 months of age, selectively, a phosphopeptide close to the polyQ-repeat before the LSm domain accumulates > 110-fold relative to WT, and a phosphopeptide in the isoform that starts 160 residues even further upstream at the N-terminus accumulates > 30-fold [131]. This suggests that either alternatively spliced expression of small isoforms or partial degradation via proteolysis is employed by cells, and the disease mechanisms in SCA2 and ALS do not necessarily concern the full-length protein. ATXN2-fragment-specific disease mechanisms should also be taken into account.

### 2.6. Most N-Terminal Start Codon with Subsequent Fragment Appears in Armadillo Only for ATXN2

As mentioned above, the analysis of the ATXN2 start codons in humans and closely related primates shows at least three different translation starts across the N-terminus for isoforms in *Homo sapiens* and four different isoforms in *Pongo abelii*, as well as *Macaca nemestrina* (Table S1A). The most N-terminal alternatively spliced sequence starts with M(X)R(PX)S/T(XX)A… and is clearly conserved with quite constant length from primates until artiodactyla, and according to one database entry, even in armadillos (Table S1B), suggesting that it has functional relevance. This N-terminal extension is not observed for ATXN2L, so its function is specific to ATXN2. It starts with a sequence that has a strong likelihood to act as a mitochondrial targeting signal (score of 0.9937 in MitoProt v1.101, with predicted cleavage site at residue 144; score of 0.75 at TPpred3 for residues 1–38), and then contains three proline-rich motifs (PRMs) at amino acid residues 55–71, 117–123, and 142–148 in human ATXN2 (Uni-Prot Q99700).

It is highly interesting that a duplication event of 21 base pairs was observed at the end of this exon 1A-encoded fragment, where human ATXN2 amino acid residues 145–151 get repeated in tandem (ARPA PGCPRPA PGCPRPA CEPV). This dup21 variant affects the last of the PRMs mentioned above. It was observed in a Japanese patient with a clinical, imaging, and electrophysiological phenotype of SCA2 who carried no CAG-repeat expansion in *ATXN2*, suggesting that this variant plays a pathogenic role. Recombinant ATXN2 protein with the dup21 variant showed clear aggregation tendency and caused reduced cellular viability upon transient transfection of a Schwann cell line. Even more interestingly, it triggered recognition of the normal polyQ domain length by the monoclonal 1C2 antibody as if it had the abnormal epitope characteristics of an expanded polyQ domain, so apparently, the proximity between the duplicated PRM and the polyQ domain leads to conformational changes in the ATXN2 N-terminus that seem pathognomonic [132].

Another duplication event of nine bases was reported more towards the N-terminus in this exon 1A-encoded fragment, where human ATXN2 amino acid residues 38–40 get repeated in tandem (PARR SGR SGR GGGG). This dup9 variant was observed in a small Swedish family where a father and his daughter were diagnosed with Spinocerebellar Ataxia type 3 (SCA3) due to polyQ-expansions in the ATXN3 disease protein. In both cases, the additional presence of the *ATXN2* dup9 mutation was accompanied by unusually early disease manifestations and a Parkinsonian phenotype upon clinical, neuroimaging and neuropathology studies. Furthermore, this dup9 variant was also present in two patients diagnosed with the C9ORF72 type of ALS, appearing again to be associated with an unusually early age at onset and with Parkinsonian features. A bidirectional study of expression (*ATXN2*-S/AS) revealed significantly higher expression of this dup9 variant [133]. This potential impact of the dup9 variant on expression levels seems credible, given that a CpG island for promoter activity is situated within exon 1 of human *ATXN2* [134].

Sequences with significant homology (BlastP score < 5 × 10^−7^) to this fragment are not found in ATXN2 orthologs among paenungulata and marsupialia but instead are contained in the SKI family transcriptional corepressor 1 (SKOR1), actin nucleation-promoting factor (WAS), and Splicing Factor 3B subunit (SF3B4) from *Vombatus ursinus*, *Phasolarctos cinereus*, and *Monodelphis domestica*. In humans, close homologies exist with sequences within Transcription initiation factor TFIID subunit 4 (TAF4, 1.1 × 10^−9^) and the Splicing factor, proline- and glutamine-rich (SFPQ, 7.8 × 10^−8^), and also with proline-rich protein 36 (PRR36, 4.6 × 10^−8^), Formin-2 (FMN2, 2.1 × 10^−7^), and actin-binding protein WASF2 (8.5 × 10^−7^).

Structural prediction software in InterPro characterizes this sequence as an intrinsically disordered region (IDR) with low complexity and proline-rich stretches, and the previous literature has named all sequences upstream from the LSm domain as IDR1 of ATXN2 [62]. Thus, the above observations are in agreement with a genome-wide survey concluding that Pro-rich sequences are involved in actin/cytoskeletal-associated functions, RNA splicing/turnover, DNA binding/transcription, and cell signaling [135,136]. At the N-terminus, this proline-rich sequence may influence the folding of nascent ATXN2. Its presence in recombinant full-length ATXN2 constructs for in vitro experiments, as well as the absence of such N-terminal sequences from ATXN2-null organisms, may have contributed to reports that ATXN2 is involved in actin–plastin pathways and in actin–endocytosis pathways [52,53,57,58,137]. However, the effects of Ataxin-2 on mitochondrial biogenesis, mitophagy, and oxidative stress responses cannot be attributed only to this IDR1, given that such an impact is already documented in the yeast ortholog Pbp1p (subsequently referred to as PBP1) [67,68,69,70,138,139,140,141].

### 2.7. The Usual Start Codon in Human ATXN2/ATXN2L Is Followed by polyQ and a Repeat-Rich Fragment, Which Elongates Since Yeast/Insects

**Figure 5:**
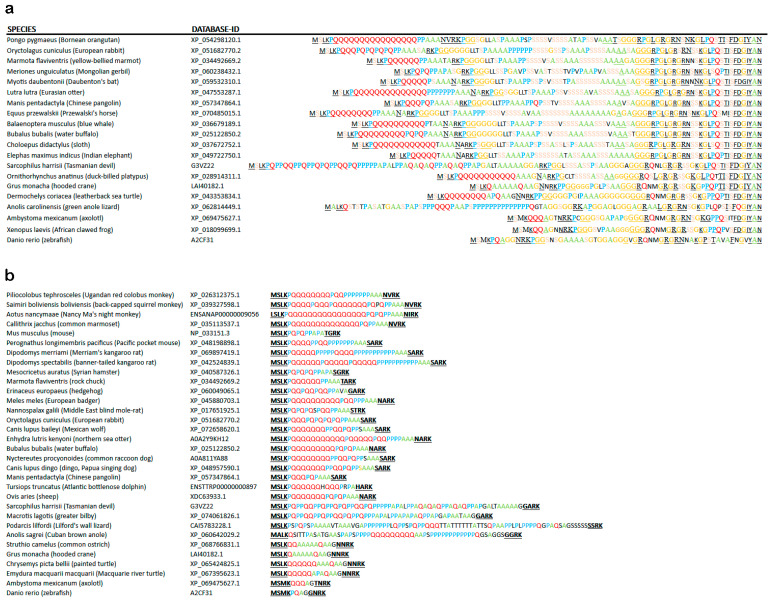
Evolution of the N-terminal IDR1 of ATXN2 (encoded by exons 1B, 2, and 3) with multiple repeat structures since ray-finned fish. (**a**) Noticeable length extension is documented, with the human sequence being too long to be included. While the sequences encoded by exons 2 and 3A were quite conserved, and the MS(M/L)K(P/Q) motif downstream from the translation start remained stable, a lengthening of polyQ (in red letters), polyP (blue letters), polyA (green), polyG (orange), and polyS (pink) domains is apparent since turtles, reptiles, and marsupials (see also Table S1A). The polyQ repeat, whose expansion is pathogenic in the human disorders SCA2 and ALS13, has sizes of *n* ≥ 3 since axolotl amphibia, *n* ≥ 5 since turtles and other reptiles, *n* ≥ 10 since *Platypus* and *Dasypus* (armadillo) mammals, *n* ≥ 15 since carnivores and bats, and *n* ≥ 20 since primates. Conserved sequence patterns were underlined for easy visibility. (**b**) Non-random preference for Pro and Ala as interrupting residues or flanking residues across the polyQ domain. PolyA repeats arose next to polyQ in turtles, and substituted for polyQ in flying birds, while polyP repeats were intermingled with polyQ and polyA in lizards. Pro residues came to flank the polyQ repeat and to replace residues within the polyQ repeat since marsupials like the Tasmanian devil or the greater bilby. Thus, Pro and Ala residues appear to be exchangeable with Glu residues, with the physiological function of this IDR1 within ATXN2 remaining intact.

The second alternatively spliced exon in ATXN2 encodes the highly conserved translation start sequence MS(L/M)K(P)n(Q)n(P)n to produce an additional N-terminal fragment of up to 50–120 aa that contains several repeats, namely polyQ, polyPro, polyAla, polyGly and polySer, with one or several prolines usually at each side of the polyQ repeat. Within this sequence downstream from the polyQ repeat and before the polySer repeat, 17 amino acid residues enable N-terminal proteolysis of mammalian ATXN2 [142]. The entry A0A2K5X824 in *Macaca nemestrina* suggests that an alternative start sequence MGPHHVAEAP(A)_n_T(A)n can also be used, producing a fragment that contains a polyA repeat instead of the polyQ repeat. This fragment immediately before the LSm domain start does not show a relatively constant length, in contrast to the previous paragraph, but instead shows a massive extension across phylogenesis. A close inspection of the sequences compiled in Table S1D reveals the repeated appearance of polyQ repeats in testudines, again in monotremata, in proboscidea, and in primates. Instead of an N-terminal mammalian polyQ-repeat (MSLKPQQQQQQQQQQQQQQQQPPAAA), which is found in primate monkeys (XP_054298120.1), birds and reptiles show polyAla (MSLKQAAAAAQAA in *Struthio camelus*, see entry XP_068766831.1), which seems to fulfil an analogous function [143] (Figure 5a). Both repeat types are known for their pathogenic expansion in patients [144,145]. PolyAla and polyGly repeats are found at each side of the fragment. In the center, a polySer repeat without or with interruptions has appeared with increasing size since monotremata. Around the first 100 amino acid residues of the human sequence are predicted to have an intrinsically disordered structure. The fact that this fragment extends in length since yeast/insects together with its poor conservation of individual amino acid residues may suggest that several repeats function as spacers between domains that interact, e.g., during autoregulation.

The N-terminal fragment before the LSm domain in ATXN2L, in comparison, has only doubled in length since *Danio rerio* and contains only short repeats without much variation. It starts since *Xenopus* with the conserved sequence M(L/S)(K/M)(P/Q/K)(Q/P)(P/Q). About the first fifty amino acids of human ATXN2L are predicted to show an intrinsically disordered structure. The total length of IDR1 sequences upstream from the LSm domain reaches 250 aa residues in human ATXN2L.

In contrast to previous assertions that long polyQ domains in N-terminal ATXN2 are exclusively found in primates, this datamining effort also observed ATXN2 orthologs with a Q > 5 domain in flying lemur, pika, rodents such as hamster/beaver/kangaroo rat, bats, carnivores, horses, unpaired and paired ungulates, armadillo, sloth, aardvark, duck-billed platypus, alligator, and leatherback sea turtle.

In human ATXN2 (UniProt Q99700), the InterPro sequence analysis shows the polyQ repeat at residues 166–188 to have a high alpha-fold confidence, and the aa residues 156–202 (YGPLTMSLKPQQQQQQQQQQQQQQQQQQQQQQQPPPAAANVRKPGGS), especially the residues downstream from the polyQ repeat, to display significant homology with the beta-sandwich domain in Sec23/24. The Sec23/24 heterodimer regulates the trafficking of membrane proteins, phospholipids, sphingolipids, and sterols via COPII-coated vesicles from the ER to the Golgi apparatus and to elongating cell protrusions [146]. This homology is not detected by the InterPro algorithm in other species. A fly dATX2 isoform indeed colocalizes with Sec23, but this was reported for isoform C, which starts from the LSm domain, similar to isoform A, but no Sec23 colocalization was found for isoform B, which starts 60 aa before the LSm domain begins [147]. While it remains unclear if Sec23 is relevant in this context, human ATXN2 indeed associates with the ER, the disruption of Ataxin-2 in flies and nematodes disrupts ER dynamics, and the polyQ-expansion in human and mouse ATXN2, as well as the deletion of mouse ATXN2, leads to disturbed sterol and sphingolipid homeostasis [51,54,55,137,147,148,149,150]. To what degree N-terminal sequences of Ataxin-2 contribute to these effects remains to be elucidated.

The pathogenic expansion of the polyQ repeat within this translation start sequence to sizes ≥ 33Q is the monogenic cause of autosomal dominant SCA2, and at sizes 31 < (Q)n < 33, it contributes in a polygenic manner to the risk of sporadic ALS. It is interesting to note here that despite this long-term neurotoxicity of >31Q stretches, long polyQ repeats appear among ATXN2 orthologs initially towards the C-terminus already in single-cell organisms like red algae (UniProt A0A2V3IVX2), amoebozoa (Q55DE7), and dinoflagellates (A0A813ABV5, where an extremely long 73Q repeat is interrupted at three positions by single Met residues, or A0AA36IYW2, where a 41Q repeat is interrupted at 11 positions by single or double Ala residues), as well as insects like *Hymenoptera* (A0A158NW33, A0A836FT30, F4X2F6, A0A834JVP6, A0A834JR87), so they seem to play a relevant role for the functions of Ataxin-2 but are not documented for ATXN2L orthologs so far. Smaller polyQ repeat sizes 5 < (Q)n ≤ 31 are frequent among Ataxin-2 homologs towards the C-terminus (e.g., in chlorophytes such as A0A835VQM0, A0A9W6BKV0, nematodes such as MCP9264076.1, or insects such as Q8SWR8).

After this repeat-rich fragment but before the LSm domain, a connecting sequence is encoded by exons 2 and 3A in ATXN2 (for ATXN2L encoded by exon 2 and the first nine residues from exon 3), which appears in isoforms initiated from the first or second translation start codon and should therefore be regulated with exon 1B. However, their respective exons are separated genomically from exon 1, clustering instead with the subsequent exons that encode the LSm domain, possibly suggesting that this sequence may be regulated as an LSm modifier.

### 2.8. The Role of Proline Flanking Residues and Interrupting Residues for the polyQ Repeat

The observation that the pathogenic N-terminal polyQ repeat of ATXN2-IDR1 is adjacent to, or framed by, polyPro repeats or Pro residues (Figure 5b) is also documented for the pathogenic polyQ expansions in Huntington’s disease [151]. Whereas PRMs are thought to require the presence of an Arg residue for their interactions with actin and endocytosis apparatus, the polyPro residues or Pro residues at the flanks or within polyQ repeats may play different roles. Pro residues are thought to modulate the poorly solvent polyQ stretch towards an α-helix secondary structure [152] as well as mediating interactions, e.g., with SH3 domains [153,154] and with membrane lipid bilayers [155]. The presence of polyPro helices in the intrinsically disordered N-terminal domain of human CPEB3 was shown to modulate solubility, acting as an amyloid breaker [156]. Importantly, proline- or glutamine-rich activation domains are a common feature of transcription factors [157], so in the case of cytoplasmic Ataxin-2 orthologs, it is plausible that these sequences influence the binding to RNA.

Prolines not only serve a role in flanking the polyQ repeat. They are also frequently used to interrupt the polyQ domain. The interspersion of prolines or polyPro sequences within the polyQ repeat is observed not only in primates and some rodentia but also in lagomorpha, pholidota, artiodactyla, marsupiala, and squamata (Table S1C). Detailed studies of Ataxin-2 orthologs with amino acid residues interrupting its polyQ domain indicate that Pro >>> His >> Ala > Met = Arg = Ser = Gly are most used to interrupt polyQ stretches. The flanking residue on the left side is most frequently Pro >> His > Met = Lys = Gly = Val = Ala = Arg, while the flanking residue on the right side is more often Pro >>> Met = Arg = His > Ala = Asp = Glu = Gly. Overall, the amino acid residues that flank and interrupt the polyQ domain are non-random. Flanking (poly)Pro or interrupting Pro was the most frequent feature throughout the evolution of Ataxin-2 orthologs.

### 2.9. Ancient Start Codon Preceding LSm Domain as an Optional Third Start in Human ATXN2

The optional third translation starting immediately upstream from LSm was in very frequent use during evolution until reptiles and is still documented in primates. The first of two methionine residues that may serve as a start codon in exon 3B was lost in muridae and cricetidae among rodentia, together with insectivora (VRMVHILTSVV… instead of MRMVHILTSVV…), but lagomorpha, dermoptera, and primates still have this first methionine (see Table S1E). Here, an isoform with abundant expression in spinocerebellar tissue can be produced that corresponds to Ataxin-2 from LSm until the C-terminus, without the appended N-terminus. This very ancient translation start codon produces an isoform with all core ATXN2 sequences (e.g., in XP_067991570.1). Deletion experiments in *Drosophila melanogaster* indicated that the LSm domain stimulates mRNA translation. At the same time, it antagonizes the assembly of ribonucleoprotein granules [83]. Via RNA binding, the LSm domain influences the assembly and stability of microtubules in *Drosophila melanogaster* neurites [63].

The crucial residues, with high conservation rate in the LSm domain of ATXN2 from mammals to insects, versus ATXN2L from mammals to amphibians, are compiled in Figure 2a,b. The LSm sequence variation in Ataxin-2 orthologs from nematodes to single-cell organisms is extensive, with insertions at various sites, so that multiple sequence alignments have difficulty defining core amino acids. It is worth noting again that the LSm sequence has limited usefulness in identifying Ataxin-2 orthologs, while the consensus LSmAD across evolution (and IDR2/IDR3 within organism phyla) is much more powerful. Indeed, BlastP searches with the latter consensus sequences among fungi identify more than half of Ataxin-2 orthologs that have no recognized LSm domain, suggesting that the algorithms employed by InterPro and Pfam to identify LSm-folds need to be optimized. It is unlikely that the LSm-fold acts as a commodity rather than a necessity for Ataxin-2 functions, given that interference with this LSm domain is embryonically lethal.

The LSm domain, as an ancient RNA-binding motif with oligo(U)-specificity [158,159], is known to derive from Hfq protein homologs in bacteria/archaea, where it assembles in homoheptameric rings and performs RNA chaperone functions [160]. Hfq-binding RNAs were experimentally identified in *Salmonella typhimurium* bacteria, share a similar structure composed of three stem-loops, are enriched in the interacting sequence motif 5′-AAYAAYAA-3′, and show Hfq-association at AU-rich single-strand sequences adjacent to the loops, or the polyU end of sRNAs [161,162,163,164,165,166,167,168,169,170,171]. Hfq interacts with regulatory short RNAs, modulates RNA decay, acts in ribosome biogenesis, and influences mRNA translation, showing localizations in the cytoplasm, in the nucleoid, and at the bacterial cell membrane [172,173,174,175]. Importantly, the loss of Hfq causes increased membrane permeability and more sensitivity to oxidative stress, activating envelope stress responses via the sigma transcription factor RpoS for envelope stress [172,176,177,178,179,180,181], as well as leading to the activation of outer membrane proteins, enzymes, and transporters, which are involved in amino acid uptake and biosynthesis, sugar uptake / metabolism, and cell energetics [172,180,182].

Hfq homologs were adapted in eukaryotic nuclei as Sm modules in the heteroheptameric ring of splice proteins, and in eukaryotic cytoplasm in more than a dozen proteins implicated in RNA processing or decay, translation control, the telomerase RNA biogenesis, cell signaling, and gametogenesis [183,184,185,186,187,188,189,190,191,192,193,194]. Outside of ring structures, the LSm domain exists, e.g., in LSM11 as a factor associated with the methylosome [195] or LSM12 as a possible methyltransferase and/or NAADP receptor [192,196]. Thus, the LSm-fold sequence consensus is not specific for Ataxin-2 orthologs. However, the LSm domain still associates preferentially with AU-rich sequences, usually in the 3′ untranslated region of mRNAs [197].

### 2.10. RNA Processing Factors May Bind to the LSm-LSmAD Region, or Be Added to N/C-Termini of Ataxin-2 Orthologs, in Dependence on the Biochemical Needs of Different Ecological Niches

The region including the LSm and the LSmAD sequence contains around 250 residues, and in mammalian ATXN2, it was observed to mediate binding to the RNA helicase DDX6 [64,198] and to repress the illegitimate RAN translation of the CAG-repeat [199]. It is therefore interesting that *Coptotermes formosanus* termites show a N-terminal stable addition of a DEAD box RNA helicase domain (DDX52 homologous) to their Ataxin-2 ortholog (UniProt entry A0A6L2P9Q7), *Trichogramma brassicae* wasps have a C-terminal addition of a DEAD box helicase (A0A6H5ISX2), and *Coemansia* sp. fungi have a C-terminal addition of an RNA recognition motif (A0A9W7Z4B9) (see Table S3). These observations support the concept that eukaryotic organisms have optimized multi-domain proteins in order to concentrate all cofactors and substrates of a pathway in one place, thus maximizing throughput efficiency.

In analogy, further RNA processing functions that complement Ataxin-2 roles may have been integrated into its multi-domain structure, to ensure the maximal efficiency needed for different environmental stresses. Observations of various domains added at the N- or C-terminus of Ataxin-2 orthologs are therefore compiled in Table S3.

A C-terminal addition of a sequence with homology to HhH-GPD domains, like NTH1, occurred in *Rhodotorula* sp. CCFEE 5036 fungus (A0A4U0VMY8). HhH-GPD, like NTH1, recognizes and repairs 8-oxoguanine and other oxidative damage in DNA [200,201].

N-terminal addition of a tetratricopeptide domain plus a SAM-dependent 23S rRNA methyltransferase domain in *Paramicrosporidium saccamoebae* fungus (A0A2H9TMQ1), only an N-terminal 23S rRNA methyltransferase domain in *Actinomortierella ambigua* fungus (KAF9164688.1), or C-terminal addition of a 28S rRNA (cytosine-C5)-methyltransferase domain in *Pseudozyma flocculosa* fungus (A0A5C3F1S9) and a C-terminal SAM-dependent RNA (cytosine-C5)-methyltransferase domain in *Wallemia ichthyophaga* fungus (A0A4T0IZQ6) to the Ataxin-2 sequence provide further support for the relevance of this pathway. Tetratricopeptide repeat contacts bind RNA and assemble the splice apparatus [202], while eukaryotic 5-methylcytosine (m^5^C) RNA methyltransferases are crucial for responses to oxidative stress [203,204].

Similarly, the N-terminal addition of ribosomal protein L9 N-terminal and C-terminal domains to the Ataxin-2 sequence in *Coemansia interrupta* fungus (A0A9W8LDP4), *Coemansia spiralis* fungus (A0A9W8GBK0), *Coemansia biformis* fungus (A0A9W8CWC1), and *Coemansia pectinata* fungus (A0A9W8H1X6) underlines this concept.

A ribonuclease inhibitor LRR repeat was added to the Ataxin-2 ortholog of *Rhizoctonia solani* fungi (A0A074SQD8) and of *Ceratobasidium* sp. fungi (A0A8H8MCZ2). The ribonuclease inhibitor serves to constrain the cytotoxicity of RNase A [205].

Regulation of the RNA polymerase was achieved by the addition of a CBF-B/NF-YA transcription factor domain in *Rhododendron giersonianum* shrub (A0AAV6I6Y5), or by the addition of an ACID domain of the MED25 complex in *Certonardoa semiregularis* sea stars (UPI001FC18B4E) to their Ataxin-2 orthologs. The MED25 complex regulates RNA polymerase via transcription modulators such as MYC [206].

The chimeric C-terminal addition of a RIO2 kinase domain in the bony fish *Anabarilius grahami* to the Ataxin-2 ortholog (ROL51429.1) points to rRNA processing, given that yeast RIO2 is required for the final endonucleolytic cleavage of 20S pre-rRNA at site D in the cytoplasm, converting it into the mature 18S rRNA [207]. The human ortholog RIOK2 also modulates neurotoxicity in mitochondrial complex I deficiency. In addition, it modulates ageing via telomere shortening [208,209]. It is important to note here that the *RIOK2* gene has been genomically localized next to the *ATXN2* gene since bony fish.

As further evidence that Ataxin-2 acts in RNA surveillance pathways, an N-terminal addition of a BRAP2 signature with an RNA-binding RRM motif, followed by a C3H2C3-type RING-H2 finger and a UBP-type zinc finger, occurred in the Ataxin-2 ortholog from *Pituophis catenifer* snake (XP_070807217.1). BRAP2 regulates cell survival via the antioxidant transcription factor NRF2 (SKN-1 in nematodes) and via the DNA repair factor BRCA1 [210,211,212]. Again, it is noteworthy that the *BRAP* gene is the next neighbor of the *ATXN2* gene upstream from its translation start on chromosome 12q in the human genome.

Only a HECT-type E3 ubiquitin transferase domain was added to the N-terminus of Ataxin-2 in *Furculomyces boomerangus* fungus (A0A2T9YUP5), implicating this ortholog in protein degradation pathways.

Despite current concepts that Ataxin-2 family members impact RNA processing mostly for mRNA transcript turnover with a poly(A)-tail via the PAM2 motif, the chimeric extra domains added to Ataxin-2 are clearly enriched for rRNA processing functions like methylation, rather than for mRNA decay versus repair management. Thus, this protein family appears to contribute to adaptations of the translation machinery after oxidative stress, with the LSm domain providing direct binding to RNA, while the variation in LSm sequences between orthologs and paralogs may relate to differences in the target sequence.

However, the structured Ataxin-2 sequences from LSm to LSmAD are not only important for RNA processing and quality control. Observations in *Drosophila melanogaster* show that Ataxin-2, but not its intrinsically disordered sequences, is crucial for the pathfinding of neurites in vivo [213], and, interestingly, these roles of Ataxin-2 were also fine-tuned by chimeric extra domains in some species.

### 2.11. LSmAD Became the Hallmark of Ataxin-2 in Rhodophytes and Protists

The LSmAD sequence of ATXN2 in *Homo sapiens* is encoded by exons 7 and 8, with genomic clustering suggesting that exons 6 and 9 are co-regulated with LSmAD.

The LSmAD sequence is unique and is the hallmark for the Ataxin-2 protein family, in contrast to the LSm domain that also exists in the LSM1-16 protein family for each species and in contrast to the PAM2 motif that also occurs in PAIP1/2, LARP4, eRF3/GSPT1/2, TTC3, USP10, PAN3, GW182, Tob1/2 and in other factors across the diverse species [214,215]. Since the presence of an LSmAD sequence defines the Ataxin-2 family, it is very unfortunate that there are no insights into the LSmAD functions or into the molecular interaction partners of LSmAD. Regarding the interaction of LSm with target mRNAs, the LSmAD sequence has an enhancing role in *Drosophila melanogaster* brains [216], and indeed, it was shown to modulate LSm conformation [77]. Interestingly, it was also reported that LSmAD mediates the localization of ATXN2 to the Golgi apparatus [50]. Luckily, LSmAD is highly conserved in sequence and length across evolution, so it is uniquely helpful to identify orthologs of Ataxin-2. Using BlastP to search for homologs of yeast PBP1-LSmAD (FGVKSTFDEHLYTTKINKDDPNYSKRLQEAERIAKEESQGTSGNIHIAE-DRGIIIDDSGLDEEDLYSGVDRR) among single-cell organisms, nothing significant was detected in bacteria and archaea. The LSmAD sequence was also not detected in ciliate/flagellate parasites except *Trypanosoma* and *Plasmodium* (both adapted to oxidative stress environments like blood cells), and already in rhodophytes and chlorophytes. However, among eukaryotic protists, plants, and animals, many orthologs were found with high significance (BlastP score < 1 × 10^-10^) and compiled in Table S1F. Thus, the LSmAD sequence appeared only in species with multi-domain proteins involved in stress responses that evolved to counteract oxidative damage due to the endosymbiosis of mitochondria and chloroplasts and due to exposure to sunlight.

Regarding crucial amino acids within this domain, it was reported that LSmAD contains a clathrin-mediated trans-Golgi signal (YDS, amino acid 414–416 in human ATXN2) and an endoplasmic reticulum exit signal (ERD, amino acid 426–428 in human ATXN2) [49]. However, the experimental evidence for their functional role was never unequivocal, with both not being well conserved in ATXN2, and ERD not being well conserved in ATXN2L either, despite the LSmAD sequence showing astonishing conservation since protists (Table S1F).

In view of this strong homology among LSmAD sequences throughout evolution (see Table S1F), we used them in extensive BlastP searches to define Ataxin-2 orthologs with unusual length (>1000 residues) that contain further protein domains of known function. The findings are reported in the last paragraphs of the Results text. It is relevant to know that many LSmAD-containing factors among chlorophytes were named CTC-interacting domain (CID) proteins, which refers to the presence of a PAM2 motif and its interaction with PAB1-binding protein (the MLLE domain of poly(A)-binding proteins was previously known as CTC domain) [217].

### 2.12. Sequence After LSmAD, Including the PAM2 Motif, Has a Polyampholytic Intrinsically Disordered Structure and Is Modified by Alternatively Spliced Exons Ante-10 and 10 in Human ATXN2

Downstream from the LSmAD border, the Ataxin-2 orthologous proteins are predicted to be mostly intrinsically disordered, with short interruptions at irregular positions. The total length of Ataxin-2 orthologs in protozoa and fungi varies between 650 and 1200 amino acids, with great variability and only a few consistent trends from algae to mammals. Practically all of the length variability occurs among the disordered sequences, much more towards the C-terminus in IDR2 before PAM2 (Table S1G), and IDR3 after PAM2 than at the N-terminus in IDR1. Thus, it is clear that the length and properties of these intrinsically disordered sequences are carefully controlled. The IDR2 clearly shows consensus patterns that differ between vertebrates, insects, fungi, and algae. Size variation and occurrence of primitive repeats in IDR2 are considerable.

RNA surveillance occurs in stress granules, and fat storage occurs in lipid droplets, both of which exist in liquid–liquid phase separation (LLPS). Thus, the quite variable sequences with polyampholytic intrinsically disordered structure [218] immediately from LSmAD to the C-terminus (extending up to 1200 amino acids in orthologs, like XP_061722979.1 in *Cydia pomonella,* where an unusual positive polyelectrolyte repeat follows the LSmAD sequence, or KAJ8263487.1 in *Conger conger*, or KAJ1504482.1 in *Coelomomyces lativittatus)* may play a role in localizing Ataxin-2 in the vicinity of these cytoplasmic compartments.

The fact that the human *ATXN2* gene has the alternatively spliced exons ante-10 and 10, at the end of the LSmAD cluster of exons, represents more evidence that this ATXN2 region has importance and is regulated in dependence on functional context, like stress.

### 2.13. PAM2 Motif in Many Protists and Insects Lies Close to Long polyQ Stretches

PAM2, as a short linear motif, is immersed among sequences with intrinsically disordered structures (Table S1H) at about 3–4 fifths of the total protein length, in contrast to LSm and LSmAD, where InterPro pLDDT scores predict high alpha- (or beta)-fold confidence. The previous literature has named the intrinsically disordered sequence until PAM2 as IDR2, and the intrinsically disordered sequence after PAM2 as IDR3 [62]. PAM2 is crucial for the ability of ATXN2 to be incorporated into stress granules [62,219]. PAM2 sequences changed considerably during evolution, so the InterPro algorithm often does not detect them, particularly in lower species; therefore, such candidate PAM2 motifs are compiled in Table S1H. It was already reported for CID3/CID4 in land plants [217] that a part can be repeated in tandem (sekstLNPNAKEFk-LNPNAKSFtps), but among excavates, amoebozoa, and algae, there is only an imperfect core sequence with considerable variability for Ataxin-2 family members (pksksKLNPNAKpFsLNiNAKeF, see Table S1H).

Within the IDRs on each side of the PAM2 motif, polyQ repeat sequences are frequent from protists until insects, reaching up to 30Q without interruption (downstream from PAM2 for A0A2V3IVX2 in the rhodophyte *Gracilariopsis chorda*, or downstream from LSmAD for Q55DE7 in the amoebozoon *Dictyostelium discoideum*), at the extreme size of 73Q with three Met interruptions (A0A813ABV5 in *Symbiodinium necroappetens*), or later with interruptions by Pro residues (F4X2F6 in *Acromyrmex echinatior*, A0A836FCM7 in *Acromyrmex insinuator*, A0A158NW33 in *Atta cephalotes*, A0A8J2HMF1 in *Cotesia congregate*, A0A834JR87 in *Vespula vulgaris*, A0AA40FF43 in *Melipona bicolor*). The 73Q domain in *Symbiodinium necroappetens* is translated from nucleotides (Met coding triplets in bold) CAG(CAA)_2_(CAG)_2_CAA**ATG**(CAG)_2_CAA(CAG)_2_CAA(CAG)_2_CAA(CAG)_2_CAA(CAG)_4_CAA(CAG)_2_CAA(CAG)_6_CAA(CAG)_3_CAA**ATG**(CAG)_2_CAA(CAG)_2_(CAA)_2_(CAG)_2_CAA(CAG)_2_CAA(CAG)_4_(CAA)_2_(CAG)_3_(CAA)_3_(CAG)_3_CAA(CAG)_2_CAA(CAG)_3_**ATG**CAG (bold letters for triplets encoding the interrupting Met residues), while the 30Q domain in *Gracilariopsis chorda* red algae gets translated from nucleotides (CAA)_3_CAG(CAA)_2_(CAG)_2_(CAA)_2_(CAG)_2_(CAA)_5_(CAG)_6_CAA(CAG)_2_(CAA)_4_, and the 29Q domain in slime mold *Dictyostelium discoideum* is translated from nucleotides (CAA)_29_. An uninterrupted 45Q domain in *Condylostylus longicornis* is translated from (CAA)_2_CAG(CAA)_7_CAG(CAA)_8_(CAG)_3_(CAA)_4_CAG(CAA)_5_CAG(CAA)_3_CAGCAA-(CAG)_3_(CAA)_4_, see NCBI entries XP_055375020.1 and XM_055519045.1. Thus, there seems to be neither a codon preference nor a likely repeat structure underlying very long polyQ domains. 

Downstream from LSmAD, the intrinsically disordered region 2 (IDR2) of human ATXN2 starts in the sequences encoded by exon 8 and extends across PAM2 into exon 17. The PAM2 motif in human ATXN2/ATXN2L is encoded by exons 16/15, respectively, which are genomically clustered with one preceding and two subsequent exons, suggesting joint regulation and function in this area. The subsequent constitutive *ATXN2* exons 22/23 still encode intrinsically disordered sequences (IDR3). Within the *ATXN2* exons 16 and 17, several methionine residues exist as candidate homologs of the yeast C-terminal methionine-rich region where metabolic redox sensing occurs to control phase separation of Ataxin-2 [220,221,222], but none of them is conserved in ATXN2L or in the single-copy Ataxin-2 orthologs, although the two paralogs and these orthologs show similar condensation dynamics.

### 2.14. Sequence Beyond LSmAD Without an Intrinsically Disordered Structure

In the C-terminal half of Ataxin-2 orthologs, the intrinsically disordered sequences are usually interrupted at least once, in primates up to 130 amino acids beyond the PAM2 motif, for some 40–200 residues (around position 500–880 for the typical Ataxin-2 ortholog of 1000 amino acid length) (Table S1I). For human ATXN2, this interrupting ordered sequence is encoded by exons 18/19/20, and for human ATXN2L, by exons 17/18/19. Interestingly, for human ATXN2, the alternatively spliced exon 18B evolved inside the start of this cluster, while the alternatively spliced exon 21 evolved at the end of this cluster, both encoding ordered sequences. This regulation effort is compatible with the notion that the functional relevance of this ordered area has to be modulated upon changes of stress versus stimulus.

### 2.15. C-Terminus Alternatives

The C-terminal end of Ataxin-2 orthologs can contain only an intrinsically disordered sequence, or first an intrinsically disordered part and then an ordered terminal end (Table S1I). Much alternative splicing is documented for human ATXN2 at the C-terminus, with constitutive strongly expressed exons 22/23 encoding intrinsically disordered ATXN2L-homologous sequences where some isoforms stop (e.g., Q99700-4). Furthermore, the alternatively spliced exons 24A/B encode ordered ATXN2L-homologous sequences, including their joint stop (Supplementary Material S5). In *ATXN2* exon 24A, another methionine-rich sequence is encoded as a potential similarity with the yeast PBP1-C-terminal region that acts as a sensor of metabolic redox status to control the phase separation of Ataxin-2 orthologs [220,221,222], but in the human species, these residues are situated in a structured region, and only one of these methionine residues is conserved in human ATXN2L. The translation products of the alternatively spliced exon 23B (encoding a sequence known since primates) and alternatively spliced exon 25 (encoding a Q(P/A/T)(H)_1–3_Q(P/Q)(P/Q)L sequence before an alternative stop, followed by a long non-coding 3′ untranslated region) are predicted to have an ordered structure as well, mediating the modification of the C-terminus in periods of growth versus stress.

This C-terminal sequence, encoded by exons 22–24A in human ATXN2, shows the maximum expression among all regions of Ataxin-2 according to GTEx RNAseq quantifications in nervous tissue, as mentioned above. It shows clearly conserved residues from humans until early chordates (Table S1I). Regarding its function, it was shown that the C-terminal IDR enhances Ataxin-2 target mRNA interaction and influences the long-term habituation, ribonucleoprotein (RNP) granule formation, and neurodegeneration in *Drosophila melanogaster* retina [213,216,223]. Therefore, the maximal expression levels of the C-terminal exons may serve to encode Ataxin-2 isoforms that are crucial for RNP granule formation and motility during neurite transport, while the lower production of longer isoforms, including LSm-LSmAD, might serve the transition from RNP transport to active translation in stimulated synapses [224]. It may also be relevant that this C-terminus has the highest sequence homology (28.7%, *p* = 3.5 × 10^−7^) in chordates with entry A0A8C4PYQ0, a regulator of trafficking from ER to Golgi with predicted localization to ER exit sites, thus being assumed to influence the production of phospholipids and sterols, together with protein biosynthesis and trafficking, as well as calcium homeostasis.

The definition of highly conserved residues in this C-terminal sequence among insects (Table S1I) was employed to search for related proteins among nematodes and cnidaria, finding high homology with entry A7S107 (30.9%, *p* = 3.8 × 10^−13^), again a regulator of trafficking from ER to Golgi with predicted localization to ER exit sites that is recognized as member of the SEC31 family [225,226].

### 2.16. Ataxin-2 Forms Chimeric Proteins with Very-Long-Chain Fatty Acid Synthases in Various Algae, Suggesting an Evolutionary Link and a Potential Role in Wax Biosynthesis in Plants

Several other motifs of known function were integrated with the core features of Ataxin-2 orthologs in chimeric proteins of several protozoa, algae, and plant species (Table S4). Given that plants have no mobility to run from danger and damage, stress response pathways are more prominent and more streamlined in plants than in animals. These additional domains may represent meaningful efforts to optimize the efficiency of Ataxin-2 within its biochemical context, given that the analysis of mice with mutant ATXN2 also documented prominent dysregulations in these processes. The data suggest that Ataxin-2, together with co-translated neighbor genes, has a role in membrane stress responses, with strong conservation across evolution from algae to mammals.

Firstly, always a very-long-chain 3-ketoacyl-CoA synthase domain, and variably a chalcone/stilbene synthase domain, and a 3-oxoacyl-[acyl-carrier-protein] synthase were added to Ataxin-2 in *Chlorella sorokiniana* (A0A2P6U097), *Chlorella ohadii* (A0AAD5DQN1), *Auxenochlorella protothecoides* (V5NG01), and *Micractinium conductrix* (A0A2P6V2R2). The very-long-chain 3-ketoacyl-CoA synthases (abbreviated as KCS), also known as very-long-chain fatty acid (VLCFA)-condensing enzymes, play a crucial role together with very-long-chain fatty acid elongases in the adaptation of lipid membranes to abiotic stress, prominently by the biosynthesis of (epi)cuticular wax [227,228,229].

Secondly, the additional presence of a BRX domain is documented in *Lactuca saligna* (UniProt entries A0AA36E8E6 and A0AA36E8L8). BRX maintains brassinosteroid biosynthesis above a critical threshold [230].

Thirdly, a lecithin:cholesterol/phospholipid:diacylglycerol acyltransferase (LACT/PDAT) domain is present at the N-terminus of the Ataxin-2 ortholog in *Populus alba* (A0A4U5R0B4). Plant PDAT enzymes are involved in seed lipid droplet reserves by triacylglycerol formation via an acyl-CoA-independent pathway and in membrane lipid remodeling [231,232]. Particularly in unicellular microalgae under stress conditions, the accumulation of triacylglycerol is accompanied by gradual chloroplast degradation with re-organization of membrane structures and remodeling of the lipidome [233].

Fourth, a formate/glycerate dehydrogenase catalytic domain with homology to the *Escherichia coli* SerA protein (crucial for serine biosynthesis) is added at the N-terminus of Ataxin-2 in *Symbiodinium necroappetens* (A0A813ABV5). L-serine is a precursor to sphingolipids and phospholipids [234], and formate/glycerate dehydrogenases have a role in the stress responses of plants (*Symbiodinium* carries a chloroplast) [235,236].

### 2.17. Ataxin-2 Is Chimeric with Oxysterol-Binding Proteins in Various Hexapods and with Myelin Biosynthesis Factors in Animals

This evidence from primitive plants is in good agreement with the integration of Ataxin-2 sequences into chimeric proteins among animals (Table S5).

In the arthropod–hexapod class of collembola, the Ataxin-2 domains are joined to oxysterol-binding protein sequences in *Folsomia candida* (A0A226E5R7), *Archesella cincta* (A0A1D2NII5), and *Allacma fusca* (A0A8J2NQ72, A0A8J2J4F7, A0A8J2NIC3). The role of sterols for plasma membrane dynamics is well known; oxysterols have an additional signaling function [237,238].

Furthermore, the addition of an N-terminal apolipophorin-III precursor domain to Ataxin-2 occurred in the wax-producing honey bee *Apis cerana* (A0A2A3ESF8) and in the ant *Temnothorax longispinosus* (A0A4S2KN76). In insects, apolipophorin-III assists in loading diacylglycerol, generated from triacylglycerol stores in fat bodies via the adipokinetic hormone, onto hemolymph lipophorin, and it also modifies the stress responses to bacteria [239,240].

A C-terminal addition of a Ser/Thr hydratase domain to Ataxin-2 is documented in *Paralvinella palmiformis* worms (A0AAD9K567). Such L-serine ammonia-lyases are central to the production of acetyl-CoA and lipids in other worms [241] and mediate stress responses against acid already in bacteria [242].

In *Hermetia illucens* flies (A0A7R8YXR3), a C-terminal C2HC-type zinc-finger transcription factor with homology to myelin transcription factor-1 (MYT1) is appended onto the Ataxin-2 ortholog. Here, it is important to know that animal myelin, sphingolipids, ceramides, and tear film meibum, similar to plant wax (in the Latin language, “*cera*” means wax), are produced from very-long-chain fatty acids [243]. MYT1 regulates, for example, the expression of the myelin protein PLP1 [244,245].

The *Cloeon dipterum* mayfly Ataxin-2 ortholog (A0A8S1C5C0, A0A8S1C908, A0A8S1C9E1) shows an N-terminal addition of a Ser/Thr protein kinase domain with homology to CAMKK. Human CAMKK controls adiposity and cholesterol homeostasis [246,247,248].

Among bony fishes, *Clarias magur* Ataxin-2 (KAF5904804.1) shows an N-terminal RALDH1 retinol dehydrogenase domain combined with a MAPKAPK5 domain. The human RALDH1 ortholog ALDH1A1 protects against lipid peroxidation byproducts such as malonaldehyde, while MAPKAPK5 (also known as PRAK or MK5) controls cytosolic phospholipase A2 (cPLA2) and mTORC1 among other substrates, protecting against oxidative stress in Parkinson’s disease [249,250,251,252]. With special relevance for SCA2, RALDH enzymes govern the metabolism of retinoic acid, its signaling impact, and therefore hindbrain differentiation [112]. Human *ALDH2,* encoding a major biosynthesis enzyme for retinoic acid in liver mitochondria [253], and *MAPKAPK5* are neighbor genes of *ATXN2* on chromosome 12q24.

An Ataxin-2 ortholog in the naked mole rat *Heterocephalus glaber* (G5AKU3) shows C-terminal addition of a sesquipedalian family pleckstrin homology domain. Members of this family, such as yeast Boi1/Boi2 and human FAPP1, are required for the fusion of secretory vesicles with the plasma membrane at sites of polarized growth, acting by the modulation of phosphatidylinositol 4-phosphate (PtdIns4P) levels [254,255]. Again, a sesquipedalian family member is a neighbor gene of *ATXN2*.

Overall, Ataxin-2 orthologs were combined with other sensors and effectors of membrane stress responses into longer multi-domain proteins, both in plants and animals.

### 2.18. Ataxin-2 Is Chimeric with an AMP Kinase Subunit and with a SCYL1 Ortholog in Some Fungi, Where Its Mutations May Impact Metabolic Reserves and Lipid Homeostasis

In fungi, domain additions to Ataxin-2 orthologs include (Table S6):

An N-terminal KA1/Ssp2_C domain in *Neophaeococcomyces mojaviensis* (NCBI Genbank entry KAJ9652119.1). This C-terminal domain in the serine/threonine protein kinase AMPK catalytic (alpha) subunit Ssp2 acts as a sensor to regulate cellular responses to a variety of nutritional and environmental stresses [256].

A longin-like domain exists at the C-terminus in *Saitoella complicata* fungus (UniProt entry A0A0E9NFC8). These sequences are involved in membrane dynamics regulation, occurring e.g., in GDP/GTP exchange factors for Rab7/Rab32/Rab38, such as the MON1/CCZ1-MC1 complex or the HPS1/HPS4-BLOC3 complex [257].

An ARM-like fold together with a CIK-related domain is present at the C-terminus in *Aspergillus felis* fungus (A0A8H6QL38). This combination of armadillo-repeats in CIK (Catalytically Inactive Kinase)-related proteins is preserved in human SCYL1-3, which are involved in the regulation of traffic between the Golgi apparatus and the ER, or play a role between the trans-Golgi network and the endosomal system, but also contribute to tRNA export from the nucleus [258,259]. This observation seems relevant since SCYL1 mutations cause progressive spinocerebellar neurodegeneration in mouse and human (SCAR21 type) and motor neuron disease with mislocalization of TDP-43 in mouse, similar to ALS and to ATXN2 mutation effects in humans [260,261,262,263,264,265]. SCYL1 mediates MTORC1 signals [266].

An N-terminal addition of a phospholipid transporter P-type ATPase occurred in *Physocladia* obscura fungus (A0AAD5T663 and A0AAD5XG15).

A C-terminal addition of a SEC6 domain is documented in *Candida oxycetoniae* fungus (A0AAI9WVV1) and in *Candidozyma haemuli* fungus (A0A2V1AQ46). The exocyst component SEC6 interacts with oxysterol-binding protein to regulate polarized vesicular membrane transport and growth in a sterol-dependent manner [267].

Further, an N-terminal cytochrome c oxidase, subunit Va/VI domain exists in *Racocetra fulgida* (A0A9N9AHJ9). This observation suggests that Ataxin-2 can act on the intra-mitochondrial pathway of oxidative stress generation directly.

A Calcineurin-like phosphoesterase domain was added in *Diversispora eburnea* (A0A9N8VJW7). Such a domain occurs e.g., in nucleotidases and sphingomyelin phosphodiesterases [268].

A Cysteine desulfurase was appended to the N-terminus, and at the same time, a cell cycle-regulated microtubule-associated protein (TPX2) domain was added at the C-terminus in *Bifiguratus adelaidae* (A0A261XY39). The mitochondrial cysteine desulfurase NifS/IscS/NFS1 acts in the core iron–sulfur cluster (ISC) assembly complex, where perturbations lead to the human disorder Friedreich’s ataxia [269,270]. Cellular iron–sulfur (Fe/S) proteins are involved in lipid synthesis and steroid metabolism [271]. The TPX2 protein is an assembly factor for spindles, downstream from RanGTP [272].

Thus, Ataxin-2 orthologs (in diverse species from protists and algae to higher plants, fungi, and animals) are integrated into longer chimeric proteins that enable streamlined adaptation of lipids in membranes and fat bodies upon stress exposure, together with the production of (epi)cuticular wax or myelin from very-long-chain fatty acids, and the adaptation of chloroplast/mitochondrial homeostasis. This may suggest that mutations in Ataxin-2 orthologs play a role not only in RNA quality control but also in lipid adjustments after cell damage. Since LSmAD sequences are always adjacent to LSm domains and seem to function jointly with them, it is worth considering if the sensor of oxidative stress could be the LSm via binding to misfolded or oxidized RNA, while the Ataxin-2-specific effector of stress control could be LSmAD via modifications of lipid homeostasis.

## 3. Discussion

Current knowledge of the Ataxin-2 protein family is focused on the full-length isoforms. In particular, the roles of the LSm domain and the PAM2 motif and their impact on RNA processing have been investigated, although they are not unique to Ataxin-2. In contrast, very little knowledge exists about Ataxin-2 small isoforms and about the LSmAD component, its interactors, and its functions. This focus has persisted, although small isoforms are more strongly expressed than the full-length isoform, and LSmAD exists only in the context of Ataxin-2, constituting its defining feature. The fact that Ataxin-2 family members frequently show chimerisms with added domains at either terminus of the protein was previously unknown.

Here, this manuscript represents a datamining effort to analyze the evolution of Ataxin-2 structural components, their expression regulation, and the functional impact of their mutations. For a systematic evaluation, every effort was made to identify a maximum number of orthologs of Ataxin-2 in the various kingdoms of life and to compile them in Table S1A. Overall, we hope to derive functional insights from lower organisms that can be applied in patients, where Ataxin-2 can be exploited as a crucial switch to mitigate the progression of neurodegenerative diseases [21].

**Figure 6:**
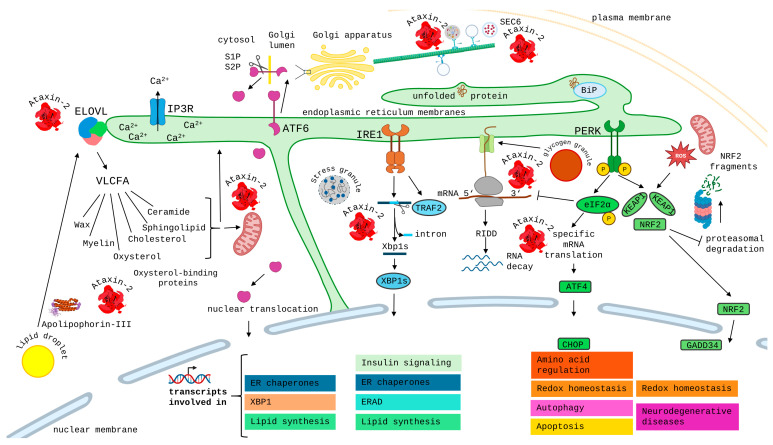
Synopsis of datamining and experimental evidence about the mutation effects of Ataxin-2, confirming its relevance for rRNA and mRNA processing and emphasizing the impact of the Ataxin-2 locus on membrane strengthening during stress periods. Fusion of additional domains onto Ataxin-2 with conservation across multiple species was documented with Apolipophorin-III, oxysterol-binding proteins, elongases (ELOVL) for very-long-chain fatty acids (VLCFA), and SEC6 proteins as exocyst components. Further fusion events clarify its integration into RNA surveillance and translation modulation pathways, in stress granules, ribonucleoprotein granules during microtubular transport, XBP1 isoform splicing, regulated IRE1-dependent RNA decay (RIDD), and specific mRNA translation. A little-expressed isoform of Ataxin-2 with a mitochondrial targeting peptide underlines its function for endosymbiont management. Previous experimental data independently showed Ataxin-2 mutations to impact lipid droplets, VLCFA elongation, plant wax, oligodendrocyte myelin, oxysterols and cholesterol, sphingolipids and ceramide, subcutaneous fat, endoplasmic reticulum calcium homeostasis, chaperones such as BiP, insulin signaling and glycogen granules, amino acid regulation, autophagy, redox homeostasis, Golgi fragmentation, ribonucleoprotein trafficking in neurites, and exo-/endocytosis of vesicles. Although Ataxin-2 mutations therefore modulate multiple pathways in ER stress, it remains unclear if Ataxin-2 acts as a selective or generalized modifier of the three main processes in unfolded protein responses, which are under the control of ATF6/IRE1/PERK. Created in BioRender. Key, J. (https://BioRender.com/8073e0f) (accessed on 8 December 2025) is licensed under CC BY 4.0.

Altogether, the data are compatible with a novel scenario where mutations in the Ataxin-2 protein orthologs since algae affect not so much mRNA but mainly rRNA, serving the biosynthesis of secreted and membrane proteins and strongly impacting membrane resilience in periods of cell stress (main datamining and experimental evidence are summarized in Figure 6). We speculate that strong expression of small isoforms of Ataxin-2 containing C-terminal IDR2 and IDR3 may alleviate the deleterious consequences of mutations in the N-terminal IDR1, so that human exon 1B seems the preferable target for knockdown therapies to prevent neurodegenerative diseases.

### 3.1. Ataxin-2 Chimerisms, Probably Due to Transcriptional Readthrough, Include Domains Enriched in Functions for rRNA Processing and Membrane Resilience

As a completely novel finding, large chimeric forms of Ataxin-2 with extra domains added to the N- or C-terminus were found to occur in approximately 5% of lower species, so this observation will be discussed first, in great detail. The lists of such domains added to Ataxin-2 in diverse species contain an enrichment of rRNA processing enzymes. If the chimerisms do not represent random errors of organismal protein synthesis or technical artifacts, then Ataxin-2 might contribute to defending and adapting the translation apparatus rRNAs in periods of cell stress and to protecting all vulnerable mRNAs with AU-rich elements and rapid turnover from oxidative damage, rather than acting on specific mRNAs. LSm and PAM2 perhaps make contact with RNA in an effort to sense oxidative damage and only then act as modifiers of translation and repair. Under such oxidative stress, lipid peroxidation and membrane damage are bound to occur in parallel, and the associated LSmAD and IDR sequences may become crucial effectors to protect the cell. A crucial question regarding LSmAD as a characteristic Ataxin-2 feature is whether it functions mainly in an auxiliary manner to modify the RNA-binding of the adjacent Lsm domain, RNA stability, and translational control, or if it modulates membrane interactions and composition upon recognition of oxidized RNA by the LSm domain.

This novel concept takes into account the ability of eukaryotic organisms to create multi-domain proteins in order to maximize the efficiency within a metabolic pathway. Given that the order of genes within chromosomes and within genomes is dynamically reshuffled across evolution, there is a continuous sorting process to optimize if neighbor genes are well matched or should be assembled differently. It was recently shown that transcriptional readthrough across neighbor genes is frequent under stress conditions for GC-rich genes (such as *Ataxin-2*) [273,274], in particular upon calcium release from ER stores [275]. Also, healthy cells show this feature in genomic regions with high transcriptional activity [276,277]. Although most genes in a genome appear to have no functional connection to their neighbors, some genes indeed are clustered with functional interactors within the same locus (HLA genes, as a famous example). It is known in humans that the chromosomal locus of Ataxin-2 is part of an exceptionally large linkage disequilibrium block across one megabase [117,278], so there is a selective advantage for these neighbor genes to stay together. It is unclear according to what principles the positioning of new genes or duplicate gene copies into a meaningful genomic context occurs and how chromosomal rearrangements are adapted over time. Given that the genomic surroundings of *Ataxin-2* have changed over time, we cannot know which among the observed chimeric proteins has a physiological added value. However, it is conceivable from the data above that mutations in *Ataxin-2* during cellular stress may also affect the transcription of neighbor genes and the translation of their domains.

Of course, some annotated proteins in databases are based on preliminary documentation, and some might be artifacts due to technical problems. The limitations of our study mainly center on our inability to distinguish artificial protein fragmentation and chimerism from physiological short isoforms and extra-long readthrough multi-domain proteins, respectively. In the following, examples are provided that support transcriptional slippage rather than ribosomal slippage [279] to underlie the observed chimerisms: in the 2577 residues of the UniProt entry A0AAF0YF69 from *Vanrija pseudolonga* fungus, in the 2585 residues of A0AA48I7S8 from *Cutaneotrichosporon cavernicola* fungus, and in the 2553 residues of A0AAD3TXG0 from *Cutaneotrichosporon spelunceum* fungus, Ataxin-2 orthologous sequences have an added cytidine deaminase domain together with immunoglobulin domains that are still enclosed within a chromosome 7 whole genome shotgun sequence, according to InterPro analysis. Such recombined fusion proteins could arise, e.g., during cDNA synthesis via erroneous annealing or may be the product of transcriptional readthrough, where intron retention due to cell stress is a known crucial feature [273,280].

Chimerisms of Ataxin-2 were frequently found among lower organisms, where cell stress is more frequent than in highly developed organisms. Interestingly, a chimerism is documented in the ATXN2 ortholog of the *Pituophis catenifer* snake (NCBI protein entry XP_070807217.1, see Table S3), where genomic information is already available. The N-terminal addition of an extra-domain with BRAP2 can indeed be explained as transcriptional readthrough, given that *BRAP2* is the closest neighbor gene upstream from *ATXN2* according to the UCSC genome browser from humans to *Alligator* reptiles and *Xenopus* amphibians, with the same direction of translation. Given that practically all genomes are unknown for the species with extra-domains added to Ataxin-2, it is probable that many or all the above chimerisms represent transcriptional readthrough events. Thus, the generation of readthrough transcripts and chimeric proteins appears to be a relevant feature of Ataxin-2, which gets activated in periods of cell stress.

Chimerism of ATXN2 among highly developed organisms, where the genome sequence is known for related species, is also documented in the *Heterocephalus glaber* naked mole rat (entry G5AKU3, see Table S5). A sesquipedalian family domain was added to its C-terminus. Again, this can be explained as transcriptional readthrough, given that the Sesquipedalian-1 protein is encoded by the *PHETA1* gene, the immediate neighbor of the *ATXN2* gene at its 3′ end, with the same transcriptional orientation, as a genomic position conserved until the human.

Further examples include the C-terminal addition of a RIO2 kinase domain to the ATXN2 ortholog of the bony fish *Anabarilius graham* (UniProt ROL51429.1). Indeed, the UCSC genome browser of the bony fish *Danio rerio* shows the *RIOK2* gene to be localized at the 3′ end of the *ATXN2* orthologous gene in the same transcriptional direction. A final example is the N-terminal addition of a RALDH1 retinol dehydrogenase domain and a MAPKAPK5 domain to the ATXN2 ortholog in the bony fish *Clarias magur* (UniProt entry KAF5904804.1). Again, the UCSC genome browser of the bony fish *Latimeria chalumnae* confirms the position of the *ALDH1A1* and *MAPKAPK5* genes at the 5′ end of the *ATXN2* gene, but interestingly, in the opposite transcriptional direction. Overall, these examples from species with known genome sequences indicate that the extra domains added onto the Ataxin-2 protein sequence are transcribed and translated from neighbor genes as a general rule.

Of course, it is important to be cautious and to ignore artifacts during datamining efforts, but further formal arguments and a host of experimental evidence led us to consider the above chimerisms as relevant, or at least worthwhile to be assessed further.

Firstly, technical artifacts should be distributed randomly across databases, but the Ataxin-2 orthologs with additional domains are frequent among low organisms but very rare among higher animals and plants.

Secondly, artifacts should implicate all pathways at random in Ataxin-2 biology, but the additional domains are preferentially involved in stress responses, rRNA processing, the mobilization of fat/glycogen stores, and the modulation of lipid membranes.

Thirdly, although aberrant fusion proteins may occur in nature as pathological events that cause pathology, e.g., cancer, their conservation across multiple related species points to a selective advantage of this fusion event in that particular ecological niche.

Fourth, previous experimental characterization of Ataxin-2 mutant organisms has revealed only small, selective rather than striking or broad changes of RNA repair or mRNA translation in normal tissues and growing cells (in summary of many transcriptome profiling efforts from humans to mice and flies [55,56,197,224,281,282,283,284,285,286,287,288,289,290,291,292,293,294]). In contrast, strong anomalies of lipid (and carbohydrate) stores and membrane homeostasis were readily demonstrable upon Ataxin-2 mutation, not only in lower organisms such as flies, but in a conserved manner until mammals. Thus, a host of previously reported experimental evidence supports the concept that Ataxin-2 mutations affect membrane composition.

Irrespective of whether the extra-domains reflect only transcriptional readthrough events that are relevant selectively for stressed cells, or are part of a context of physiological functions of Ataxin-2, the pathways surrounding such extra-functions are affected by Ataxin-2 mutations as a consequence of the existence of chimeric proteins. So, it seems relevant in the following paragraph to provide a summary of current experimental evidence that implicates Ataxin-2 mutations in membrane stress.

### 3.2. Experimental Confirmation That Mutations in Ataxin-2 Alter Membrane Resilience

In particular, the absence of the Ataxin-2 ortholog PBP1 (Pbp1p) in *Saccharomyces cerevisiae* yeast caused the downregulation of several mitochondrial metabolism enzymes, namely citrate synthase, alpha-ketoglutarate dehydrogenase, glyoxylate reductase, succinyl-CoA ligase, and ATP synthase component ATP1 [141]. PBP1 is integrated in the phosphorylation cascade between AMPK and MTORC1 [295], sensing metabolic redox status via the reversible oxidation of multiple methionines that control its condensation state [220,221,222]. Given that these methionine switches are not conserved during evolution, it is conceivable that oxidation damage is also sensed via lipid peroxidation or via oxidative RNA damage.

- In *Caenorhabditis elegans*, the Ataxin-2 ortholog regulates the fat content and size of cells, acting via MTORC1 signaling [296,297].
- In *Drosophila melanogaster* flies, Ataxin-2 accumulates in fat bodies together with Sec23 as a marker of ER exit sites [147]. The depletion of fly Ataxin-2 mitigates glycogen metabolism problems similar to mTORC1 repression rapamycin treatment [298].
- The absence of ATXN2 from *Mus musculus* tissue results in massive accumulation of lipid droplets and glycogen, together with reduced sterol degradation in cerebellum and elevated blood cholesterol [137]. Proteome profiling of these ATXN2-null mouse livers demonstrated significant deficits of enzymes in the fatty acid beta-oxidation and malonyl-CoA/methylmalonyl-CoA pathways [299]. Again, according to unbiased global proteome and metabolome profiling efforts, the ATXN2 polyQ expansion in mouse cerebellum indeed has its main impact on the very-long-chain fatty acid elongases such as ELOVL4 (residing at the ER in a multi-protein complex), the very-long-chain fatty acids VLCFA24-26 with precursors such as acetyl-CoA and N-acetylaspartate [130], and their derivative sphingolipids and ceramides [148]. This is accompanied by changes in inositol-tris-phosphate metabolism and calcium/calmodulin-dependent kinases [300]. Receptors for the inositol 1,4,5-trisphosphate lipids were also implicated in an SCA2 mouse model [301]. In the spinal cords from two different SCA2 mouse models with ATXN2 polyQ expansion, the suppression of enzymes for cholesterol biosynthesis from acetyl-CoA and squalene, together with cholesterol and oxysterol deficits, were the main findings [150,290,302]. The restoration of brain cholesterol turnover was reported to have therapeutic value in an SCA2 mouse model [303].
- For *Homo sapiens*, the brains of SCA2 patients show a reduction in C22/24-sphingomyelin and cholesterol levels [148], and the myelinated white matter of the brain is deficient [24,304,305,306,307,308,309,310], already at presymptomatic disease stages [31,311,312]. The subcutaneous fat deposits, the cheek fat body, the visceral fat, and the body weight of SCA2 patients decrease progressively, as well as the levels of testosterone as a cholesterol derivative, in peripheral tissues [313,314,315]. In an epidemiological genome-wide association study of a Japanese population, variants of ATXN2 were found to underlie the susceptibility for dyslipidemia [316].

### 3.3. What Is the Role of Ataxin-2 for the Unfolded Protein Response and Retinoic Acid Signaling?

In bacteria, the RNA chaperone Hfq, as an ancestor of the LSm proteins, responds to envelope stress via the sigma transcription factor for envelope stress, RpoS. This sigma factor is regulated either at the post-transcriptional level via Hfq or at the proteolysis level via CLPP/CLPX-mediated unfolded protein responses [181]. Membrane stress responses via sigma transcription factors were not conserved in eukaryotes. Instead, stress granules and the Unfolded Protein Response (UPR) with the Sigma-1 Receptor (S1R) at the mitochondria-associated ER membrane (MAM) in interaction with the inositol 1,4,5-trisphosphate receptor (IP3R) and Inositol-Requiring Endoribonuclease 1 (IRE1) mediate the appropriate responses for ER stress in mammals [317,318,319]. For the UPR of bacterial and mitochondrial membranes, CLPP is crucial [320,321,322,323,324,325,326], and mutations in CLPP result in progressive ataxia and leukodystrophy in patients and mice [322,327,328].

The elongation to synthesize very-long-chain fatty acids occurs at the ER and is the prerequisite for the biosynthesis of (glyco-)sphingolipids, ceramides and myelin/wax when membranes need to be strengthened [329,330]. To integrate proteins into plasma membranes/the myelin sheath/the (epi)cuticular wax, their biosynthesis under stress conditions may require internal ribosomal entry site (IRES)-dependent translation from appropriately spliced mRNAs, then post-translational modification like glycosylation in the ER lumen, trafficking via the Golgi apparatus along microtubules to their destiny in a polarized cell, and finally their turn-over by ER-associated degradation (ERAD). Ataxin-2 localizes to all of these compartments and may have evolved to modulate all of these processes during stress periods, including the recruitment of lipids from cytoplasmic fat droplets and carbohydrates from glycogen granules [137], for the modification of membrane composition and complexity.

Ataxin-2 impacts not only ER membranes but also mitochondria. It was shown to control translation preferentially for mitochondrial factors [138], to modulate PINK1 levels [70,331], and to interact with PARK2 and sequestrate it into insoluble aggregates [68,69]. Thus, the crucial cytoplasmic factors that govern mitochondrial membranes regarding stress signaling, damage repair, and autophagic elimination are within the network of Ataxin-2 functions.

Retinoic-acid-triggered cell differentiation in mammals involves ER stress UPR via XBP1 [332,333,334,335,336]. IRE1 selectively acts on the *XBP1* transcript, excising 26 nucleotides [337,338,339]. This generates an *XBP1* splice variant, which encodes a transcriptional activator to promote UPR target gene expression [337,338,339]. Very recently, ATXN2 was also found to selectively target *XBP1* mRNA, stabilizing it and suppressing its translation, until Ataxin-2 is ubiquitinated by FBXO42 in a SKP-A/CULLIN-1 complex and gets degraded [294]. XBP1 protein is not only the central transcription activator in ER stress pathways via UPR, with an important role for neurodegenerative disorders [340,341,342,343], but is also crucial in physiology for secretory cell signaling to regulate lipid and insulin metabolism, as well as autophagy [344,345,346]. IRE1 and XBP1 influence the expression of MYC/MYCN and receive feedback control from them, within the pathway where retinoic acid governs differentiation, e.g., of neuroblastoma cells [347,348,349,350,351,352], with Ataxin-2 levels previously shown to depend on MYCN within this pathway [353]. During severe ER stress, regulated IRE1-dependent decay (RIDD) of mRNAs (to lessen ER load by reducing translation and post-translational ER translocation and secretion of proteins) generates single-strand mRNA fragments that lack 5′-caps or 3′-poly(A) tails, thus activating retinoic inducible gene-I (RIG-I) protein and mitochondria-dependent innate immunity [354]. The LSM domain of Ataxin-2 might recognize such RNA fragments, and Ataxin-2 might contribute to RIDD activity.

In mammals, the mutations in both ATXN2 and CLPP cause ataxia phenotypes [1,21,327,328]. ATXN2 mutations have a profound effect on IP3R1 expression [300,355], and mutations in mammalian IP3R1 also cause a phenotype of ataxia [356]. The relevance of the IRE1-XBP1 pathway among ER stress responses is well studied in ALS but was also reported for Ataxia–Telangiectasia and Friedreich’s Ataxia [357,358,359,360,361,362,363,364]. A crucial role of Ataxin-2 orthologs for vitamin A derivatives, cholesterol, and retinoid homeostasis (as modulators of membrane strength versus stress) was evident in lower organisms and appears conserved until mammals, given that *Atxn2*-KO mice show altered retinoic acid signaling [355]. The receptors for retinoic acid and related compounds, RAR-RXR-RORalpha, are in close interaction [365,366,367,368,369]. RORalpha protects neurons against oxidative stress [370,371]. Abnormal retinoic acid signaling was reported in several forms of ataxia, namely SCA1 [372,373], SCA3 [374], SCA7 [375,376], FRDA [377], A-T [378], and the staggerer mouse mutant [379,380,381,382]. Several of these ataxias show disease course modification by Ataxin-2.

### 3.4. Are Chimerisms, Transcriptional Readthrough, and Neighbor Genes Relevant for Ataxin-2-like?

It remains unclear to what degree the roles and regulation of *ATXN2*, which remained in the ancient genomic context of Ataxin-2, are still conserved for *ATXN2L* after gene duplication into a different genomic context. Within its locus on human chromosome 16p, the same transcriptional orientation is shared among neighbor genes *EIF3C*–*NPIPB9*–*ATXN2L*–*SH2B1*–*ATP2A1*. Chimeric proteins containing ATXN2L with extra domains from its neighbor proteins were not observed in our study, although *ATXN2L* expression is also activated in human neuroblastoma cells during stress periods [128].

The observation in Figure 2 that ATXN2L has acquired a distinct sequence pattern that is clearly separate from each ATXN2 paralog throughout different species, from fish to human, is in good agreement with previous findings that the loss of one paralog does not trigger the compensatory induction of the other paralog [128]. Jointly, the data indicate separate roles in a cell.

Apart from their similar role for stress granules, ATXN2L deficiency in mice was recently shown to trigger multinucleated cells (perhaps due to deficits in nuclear membranes or their stability), selectively affecting nuclear envelope component SYNE2 at microtubule organizing centers (MTOC), the nuclear FMR1 interacting protein 2 (NUFIP1), and the nuclear alternative splicing pathway. It is also interesting to note that the nuclear homeobox transcription factor and pyramidal neuron marker CUX1 is downregulated in ATXN2L-null mouse brain, in view of the *CUX2* gene being localized at the 3′ end of the *ATXN2* gene in mouse and human, suggesting that a *CUX* expression enhancer acting in trans is part of the *ATXN2L* gene duplication segment and was affected by the mutation [59,128,129,383]. ATXN2L protein seems distributed more between the ER and the perinuclear space, while ATXN2 appears associated more with the ER, Golgi, endosomes, and plasma membrane. Therefore, we speculate that ATXN2L is specialized in the formation/maintenance/strengthening of nuclear membranes in eukaryotes, where microtubules are anchored, playing a less complex role that did not need to evolve much. In comparison, ATXN2 would be specialized for Golgi membranes, plasma membranes, and cell protrusions, a role that has become much more sophisticated for mammalian neurons/neurites/synapses than it was in protists.

### 3.5. Ataxin-2 Isoforms

Previous research mostly investigated the full-length isoforms of each species. Although several articles documented allelic splicing of Ataxin-2, and one article emphasized the existence of at least two start codons [124,126,127], any small bands in immunoblots were usually dismissed as apparent cross-reactivity. In particular, it was thought that the functions of Ataxin-2 depend on the joint presence of LSm, LsmAD, and PAM2 sequences. However, this datamining study identified many species where alternative isoforms either contain LSm and LSmAD or lack them. In addition, several database entries were encountered where the PAM2 motif was alternatively spliced out from the Ataxin-2 ortholog. Figure 4 documents six alternative isoform starts before the LSM sequence, another four starts before PAM2, and three additional starts after PAM2, but there is no functional understanding of the different contexts where each isoform is useful. It is clear that database entries may be contaminated by artifacts and fragments, but the vast majority of Ataxin-2 ortholog isoform entries start with a Met start codon, as expected for functional isoforms.

Together with the evidence that the C-terminal IDR3 sequences are most strongly expressed, and that multiple variations of C-terminal but not N-terminal short isoforms exist in spinocerebellar tissue, as seen in Figure S1, it is clear that the old concepts have to be revised and the short isoforms should be systematically investigated.

The smaller isoforms towards the C-terminus may be crucial for SCA2 and ALS patients. It is conceivable that the previous experiments targeting exon 1 of mouse *Atxn2* [137,384] were partially compensated by increased generation of shorter isoforms, some of which would contain all core domains. Such compensation efforts would explain why mice with *Atxn2*-knockout or *Atxn2*-knockin targeting exon 1 have only mild phenotypes of obesity, insulin resistance, and dyslipidemia or age-associated neurodegeneration, respectively [5,52,53,55,69,70,81,124,130,131,137,148,150,285,287,289,299,300,355,384,385]. These observations clearly diverge from *Atxn2l*-knockout mice with disruption at LSm/LSmAD sequences showing mid-gestation embryonic lethality with brain cortex lamination defects [59,128,129], from the knockout of complete fly Ataxin-2, or its LSm or LSmAD domains in *Drosophila melanogaster* that result in lethality [213], and from the knockdown of nematode Ataxin-2 in *C. elegans*, which also causes embryonic lethality and germline defects [60,281,386].

### 3.6. How Are Ataxin-2 LSm and LSmAD Domains Essential for Eukaryotic Life?

The datamining about Ataxin-2 evolution confirmed that it became relevant when eukaryotic cells had already engulfed mitochondria and plastids, apparently when eukaryotic synthesis of organellar membranes started and when organelles were distributed across the cell geometry. Ataxin-2 is not yet documented in glaucophytes, but appears in rhodophyte and chlorophyte algae and in other primitive protists. Upon terrestrialization of plants and animals, gene duplications were conserved, suggesting that more stress responses required higher dosage. However, it is unclear whether higher gravitational forces or increased exposure to sunlight, heat, and drought are responsible. The fact that flying birds (although not flying bats) lost the duplicated gene might argue in favor of the importance of mechanical stress. In consequence, the notion that Ataxin-2 also has a role in the resilience of cellular membranes and cytoskeleton would be strengthened, in addition to its role in rRNA and mRNA quality control.

Overall, the essential parts of Ataxin-2 are evidently the beta-sheet-structured LSm with the alpha-helix-structured LSmAD and perhaps also the linear PAM2 sequences. On the one hand, targeted interference with these domains already causes lethality at the embryonic stage, while interference with the IDR sequences triggers mild phenotypes. On the other hand, only these three sequences and structures are strongly conserved from algae to humans, while IDRs have considerable variability.

### 3.7. How Should Future Investigations Be Re-Focused?

From the conclusions above, it is clear that samples from SCA2 and ALS13 patients, as well as their disease models and treatment interventions, should now be re-investigated. Urgent questions concern (i) the altered expression of neighbor genes due to the mutation or the therapeutic approach; (ii) in particular, the altered expression and stability of the potentially active *U7* snRNA copy at the *ATXN2* gene locus (most U7 copies are encoded on chromosome 12p13), as well as the processing of replication-dependent histone mRNA, together with the dynamics of U-bodies, Cajal bodies and spliceosomes [387,388,389,390]; (iii) the altered expression of individual *ATXN2* exons together with the stability of shorter Ataxin-2 isoforms; (iv) the altered resilience and dynamics of membranes and cytoskeleton of cells; and (v) the oxidative damage and altered methylation of rRNA in these samples.

This should be complemented by the generation of new mice with targeted conditional knockout of the ATXN2 LSm domain and of the ATXN2 LSmAD sequence, to first assess if this intervention causes lethal effects, and secondly to create tools where tissue-specific interactors of LSm and LSmAD can be studied.

Importantly, the interactor molecules of the LSmAD domain, as the characteristic feature of Ataxin-2 family members, should be documented to elucidate their functions. Subsequently, point mutations and deletions should target the LSmAD domain for a precise mapping of such interactions.

Furthermore, the impact of Ataxin-2 and its mutations on the different pathways of the unfolded protein response and the mechanical resilience/composition of membranes should be explored systematically, regarding the endoplasmic reticulum, mitochondria, plasma membrane endo/exocytosis, and nuclear membrane trafficking.

Most importantly, there is a consequence of our datamining effort for the design of preventive therapies against SCA2 and ALS13. In view of the complexity of Ataxin-2 isoforms and chimerisms, it seems crucial to selectively target the poorly expressed polyQ-surrounding IDR1 sequences encoded by exon 1B of human *ATXN2* rather than highly conserved LSm/LSmAD/PAM2 sequences or the strongly expressed C-terminal sequences where interference can trigger cell death. Selective targeting of these sequences before the LSm domain would target only a region of *ATXN2* that is non-essential and has evolved only since bony fish (Figure 4). Exon 1B encodes polyQ (as CAG repeat with CAA interruptions) and polyP (via CCC or CCG triplets), with both repeats jointly enhancing the GC content of the 5′ end of *ATXN2* and thus increasing its mRNA stability but also its propensity for chimerisms in stress periods [273,391], while differentially modulating liquid–liquid phase-separation, given that polyQ forms amyloid while polyP breaks it [156]. Such re-design of knockdown approaches to target exon 1B would avoid any impact on isoforms that are not full-length and would permit a partial compensation of functions by upregulated expression of smaller isoforms.

## 4. Materials and Methods

The publicly available bioinformatics tools employed in this study comprise the BlastP algorithm at UniProt–UniParc and NCBI databases, with the compilation of results in EXCEL as tables and in WORD as text files; additional homology searches via the STRING Heidelberg database; the identification of additional orthologs by automated interrogation of these databases (harnessing the GenAI Claude Sonnet 4.5 Code tool to compile and share data via an iterative LSMAD-bootstrapping strategy, BlastP against NCBI nr database with taxonomy filters, ConservedDomain-Search validation with strict E-value thresholds, quality tier classification, and Python/BioPython (version 1.86) implementation, followed by Suprabase data storage, and GitHub (version 3.5.4) plus Docker software (version 4.50.0) for data sharing); multi-protein sequence alignment via the CLUSTAL Omega 1.2.4 algorithm; protein domain analyses by InterPro and Pfam algorithms, as well as MitoProt and TPpred3 prediction tools of mitochondrial targeting sequences; structure analyses at ebi.ac.uk/rcsb.org databases and via PyMOL, version 3.1.0; expression analyses for mRNA isoforms at ENSEMBL and NCBI databases and for individual exons at GTExportal.org; genomic analyses of exon–intron structure via the UCSC genome browser; visualization of alternatively spliced isoforms with Maya 2025 software; and biomedical publications from the PubMed database. Regarding the protein structure 3D modelling, the AlphaFold predicted structures of human ATXN2 (AF-Q99700-F1-v6), ATXN2L (AF-Q8WWM7-F1-v6), and *G. chorda* ortholog PBPIP4 (AF-A0A2V3IVX2-F1-v6) were used in structural domain alignments. Structured regions corresponding to LSm, LSmAD, and polyQ domains were extracted from the main predictions. Alignment was performed on the extracted molecules using the built-in “align” function with default parameters in Open-Source PyMOL, v3.1.0 (Schrödinger LLC, New York, NY 10036, USA). Commercially licensed graphics software to illustrate results included Powerpoint, Adobe Photoshop, and BioRender.

## 5. Conclusions

Overall, the functions of Ataxin-2 in mRNA processing have been the main focus of attention so far, but its evolutionary data and genomic context suggest that its mutations also impact rRNA stress and membrane stress. Analysis of alternatively spliced exons and documented protein isoforms suggests low expression of N-terminal sequences, which could be targeted in neuroprotective efforts by knockdown strategies and may be compensated for by strong expression of C-terminal isoforms, thus minimizing adverse effects.

## Data Availability

The data supporting the conclusions of this article are available from the authors upon reasonable request.

## Supplementary Materials

The following supporting information can be downloaded at: https://www.mdpi.com/article/10.3390/ijms27031499/s1.

## Abbreviations

The following abbreviations are used in this manuscript:

Ala	Alanine
ALS	Amyotrophic Lateral Sclerosis
AMPK	Protein Kinase AMP-Activated Catalytic Subunit Alpha 1
Arg	Arginine
ARM	Armadillo repeat
A-T	Ataxia–Telangiectasia
ATF6	Activating Transcription Factor 6
ATP	Adenosine triphosphate
ATXN2	Ataxin-2
ATXN2L	Ataxin-2 like
BiP	Immunoglobulin-Binding Protein
BLOC3	Biogenesis of Lysosome-Related Organelles Complex-3
BRAP2	BRCA1 Associated Protein 2
BRCA1	BReast CAncer gene 1
BRX	Brevis radiX
C2HC	Cys2His2 Zinc Finger Protein Family
CAMKK	Calcium/calmodulin-Dependent Protein Kinase Kinase
CCZ1MC1	CCZ1B Vacuolar Protein Trafficking and Biogenesis Associated
CHOP	C/EBP Homologous Protein
CID3	Polyadenylate-binding Protein-interacting Protein 3
CIK	Catalytically Inactive Kinase
CLPP	ATP-dependent Clp Protease Proteolytic Subunit, mitochondrial
CoA	Coenzyme A
CPEB3	Cytoplasmic Polyadenylation Element Binding Protein 3
CTC	Carboxy-Terminal Conserved Domain
CUL-1	CULLIN-1
DDX	DEAD box RNA helicase domain
DEAD	Asp-Glu-Ala-Asp
DNA	Deoxyribonucleic Acid
ELOVL	Elongation of Very-Long-Chain Fatty Acids Protein
EMBL	European Molecular Biology Laboratory
ER	Endoplasmic Reticulum
ERAD	ER-Associated Degradation
eRF3	Eukaryotic Release Factor 3
FAPP1	Four-Phosphate-Adaptor Protein 1
FBXO42	F-box Only Protein 42,
FeS	Iron–Sulfur Cluster
FMN2	Formin-2
FTD	Fronto-Temporal Dementia
GADD34	Growth Arrest and DNA Damage-Inducible Protein
GDP	Guanosine Diphosphate
Glu	Glutamine
GSPT1	G1 to S Phase Transition 1 Protein
GTP	Guanosine Triphosphate
GW182	TNRC6 = Trinucleotide Repeat Containing 6A
HECT	Homologous to E6-AP C-Terminus
His	Histidine
HPS1/4	Heat Shock Protein 1/4
IDR	Intrinsically Disordered Sequence
IP3R1	Inositol 1,4,5-trisphosphate Receptor Type 1
IRE1	Iron-Responsive Element
KAI1	CD82 = Cluster of Differentiation 82
KCS	3-ketoacyl-CoA Synthase
KO	Knockout
LACT	Lecithin:cholesterol acyltransferase
LARP4	La-related Proteins
LLPS	Liquid–Liquid Phase Separation
LRR	Leucine-rich repeat
LSm	Like-Sm
LSM11/12	U7 snRNA-associated Sm-like protein 11/12
LSmAD	Lsm-associated Domain
MAM	Mitochondria-associated ER Membrane
MAPKAPK5	MAP kinase-activated protein kinase 5
MED25	Mediator Complex Subunit 25
Met	Methionine
MK5	MAPK-activated Protein Kinase 5
MLLE	MLLEKITG, from French Mademoiselle
MON1	Vacuolar Fusion Protein Mon1
mRNA	Messenger RNA
MTOC	Microtubule Organizing Centers
MTORC1	Mechanistic Target of Rapamycin Complex 1
MYC	Myc Proto-Oncogene Protein, derived from myelocytomatosis-neuroblastoma
MYCN	N-Myc Proto-Oncogene Protein
MYT1	myelin transcription factor-1
NAADP	Nicotinic Acid Adenine Dinucleotide Phosphate
NRF2	Nuclear Factor Erythroid 2-Related Factor 2
NTH1	Nth-Like DNA Glycosylase 1
Pbp1p	Poly(A)-binding protein (Pab1p) interacting protein
PAIP1/2	Polyadenylate-binding Protein-interacting Protein 1/2
PAM2	PABP-interacting Motif 2
PAN3	Poly(A)-Specific Ribonuclease Subunit 3
PARK2	PRKN = Parkin
PARKIN	Parkin RBR E3 Ubiquitin Protein Ligase
PBP1	Poly(A)-binding protein (Pab1p) interacting protein
PDAT	Phospholipid:diacylglycerol acyltransferase
PERK	Protein Kinase RNA-Like Endoplasmic Reticulum Kinase
PFAM	Protein Families Database
PINK1	PTEN Induced Kinase 1
polyQ	Polyglutamine repeat
PRAK	p38-Regulated/Activated Protein Kinase
PRM	Proline-rich Motifs
Pro	Proline
PRR36	Proline-rich Protein 36
PSP	Progressive Supranuclear Palsy
PtdIns4P	Phosphatidylinositol 4-phosphate
Q	Glutamine
Rab7/32/38	Ras-related Protein
RALDH1	Aldehyde Dehydrogenase 1 Family Member A1
RAN	Ras-related Nuclear Protein
RAR	Retinoid Acid Receptors
rFNA	Ribosomal RNA
RIDD	Regulated IRE1-dependent decay
RIOK2	Right Open Reading Frame Kinase
RNA	Ribonucleic acid
RNAseq	RNA sequencing
RNP	Ribonucleoprotein
ROR	RAR-related Orphan Receptors
ROS	Reactive Oxygen Species
RRM	RNA Recognition Motif
RXR	Retinoid X Receptors
SCA2	Spinocerebellar Ataxia Type 2
SCAR21	Spinocerebellar Ataxia, Autosomal Recessive 21
SCYL1	SCY1-Like Pseudokinase 1
SEC6	SNARE-binding Exocyst Subunit
Ser	Serine
SF3B4	Splicing Factor 3B subunit
SFPQ	Splicing Factor, Proline-and Glutamine-rich
shRNA	Short RNA
SKOR1	SKI Family Transcriptional Corepressor 1
SKP-A	S-phase Kinase Associated A Protein
TAF4	TFIID Subunit 4
TDP-43	TAR DNA-binding Protein 43
Tob1/2	Transducer Of ERBB2, 1
TORC2	Target of Rapamycin Complex 2
TPX2	Targeting Protein for Xklp2
TRAF2	TNF Receptor Associated Factor 2
TTC3	Tetratricopeptide Repeat Domain 3
UBP	Ubiquitin-Specific Protease
UPR	Unfolded Protein Response
USP10	Ubiquitin-Specific Peptidase 10
UV	ultra-violet light
Val	Valine
VLCFA	Very-Long-Chain Fatty Acids
WASF2	WASP Family Member 2
WT	Wildtype
XBP1	X-box Binding Protein 1

## References

[1] Pulst S.M., Nechiporuk A., Nechiporuk T., Gispert S., Chen X.N., Lopes-Cendes I., Pearlman S., Starkman S., Orozco-Diaz G., Lunkes A. (1996). Moderate expansion of a normally biallelic trinucleotide repeat in spinocerebellar ataxia type 2. Nat. Genet..

[2] Sanpei K., Takano H., Igarashi S., Sato T., Oyake M., Sasaki H., Wakisaka A., Tashiro K., Ishida Y., Ikeuchi T. (1996). Identification of the spinocerebellar ataxia type 2 gene using a direct identification of repeat expansion and cloning technique, DIRECT. Nat. Genet..

[3] Imbert G., Saudou F., Yvert G., Devys D., Trottier Y., Garnier J.M., Weber C., Mandel J.L., Cancel G., Abbas N. (1996). Cloning of the gene for spinocerebellar ataxia 2 reveals a locus with high sensitivity to expanded CAG/glutamine repeats. Nat. Genet..

[4] Elden A.C., Kim H.J., Hart M.P., Chen-Plotkin A.S., Johnson B.S., Fang X., Armakola M., Geser F., Greene R., Lu M.M. (2010). Ataxin-2 intermediate-length polyglutamine expansions are associated with increased risk for ALS. Nature.

[5] Becker L.A., Huang B., Bieri G., Ma R., Knowles D.A., Jafar-Nejad P., Messing J., Kim H.J., Soriano A., Auburger G. (2017). Therapeutic reduction of ataxin-2 extends lifespan and reduces pathology in TDP-43 mice. Nature.

[6] Shulman J.M., Feany M.B. (2003). Genetic modifiers of tauopathy in Drosophila. Genetics.

[7] Ross O.A., Rutherford N.J., Baker M., Soto-Ortolaza A.I., Carrasquillo M.M., DeJesus-Hernandez M., Adamson J., Li M., Volkening K., Finger E. (2011). Ataxin-2 repeat-length variation and neurodegeneration. Hum. Mol. Genet..

[8] Thompson A.D., Scaglione K.M., Prensner J., Gillies A.T., Chinnaiyan A., Paulson H.L., Jinwal U.K., Dickey C.A., Gestwicki J.E. (2012). Analysis of the tau-associated proteome reveals that exchange of Hsp70 for Hsp90 is involved in tau degradation. ACS Chem. Biol..

[9] Kim A., Park S.H., Jeon B. (2018). An Autopsy Case of Progressive Supranuclear Palsy with Incidental ATXN2 Expansion. JAMA Neurol..

[10] Auburger G., Diaz G.O., Capote R.F., Sanchez S.G., Perez M.P., del Cueto M.E., Meneses M.G., Farrall M., Williamson R., Chamberlain S. (1990). Autosomal dominant ataxia: Genetic evidence for locus heterogeneity from a Cuban founder-effect population. Am. J. Hum. Genet..

[11] Orozco Diaz G., Nodarse Fleites A., Cordoves Sagaz R., Auburger G. (1990). Autosomal dominant cerebellar ataxia: Clinical analysis of 263 patients from a homogeneous population in Holguin, Cuba. Neurology.

[12] Lastres-Becker I., Rub U., Auburger G. (2008). Spinocerebellar ataxia 2 (SCA2). Cerebellum.

[13] Auburger G.W. (2012). Spinocerebellar ataxia type 2. Handb. Clin. Neurol..

[14] Rub U., Schols L., Paulson H., Auburger G., Kermer P., Jen J.C., Seidel K., Korf H.W., Deller T. (2013). Clinical features, neurogenetics and neuropathology of the polyglutamine spinocerebellar ataxias type 1, 2, 3, 6 and 7. Prog. Neurobiol..

[15] Lorenzetti D., Bohlega S., Zoghbi H.Y. (1997). The expansion of the CAG repeat in ataxin-2 is a frequent cause of autosomal dominant spinocerebellar ataxia. Neurology.

[16] Lessing D., Bonini N.M. (2008). Polyglutamine genes interact to modulate the severity and progression of neurodegeneration in Drosophila. PLoS Biol..

[17] Velazquez Perez L., Cruz G.S., Santos Falcon N., Enrique Almaguer Mederos L., Escalona Batallan K., Rodriguez Labrada R., Paneque Herrera M., Laffita Mesa J.M., Rodriguez Diaz J.C., Rodriguez R.A. (2009). Molecular epidemiology of spinocerebellar ataxias in Cuba: Insights into SCA2 founder effect in Holguin. Neurosci. Lett..

[18] Sequeiros J., Seneca S., Martindale J. (2010). Consensus and controversies in best practices for molecular genetic testing of spinocerebellar ataxias. Eur. J. Hum. Genet..

[19] Lyasota O., Dorohova A., Hernandez-Caceres J.L., Svidlov A., Tekutskaya E., Drobotenko M., Dzhimak S. (2024). Stability of the CAG Tract in the ATXN2 Gene Depends on the Localization of CAA Interruptions. Biomedicines.

[20] Matsuura T., Sasaki H., Yabe I., Hamada K., Hamada T., Shitara M., Tashiro K. (1999). Mosaicism of unstable CAG repeats in the brain of spinocerebellar ataxia type 2. J. Neurol..

[21] Auburger G., Sen N.E., Meierhofer D., Basak A.N., Gitler A.D. (2017). Efficient Prevention of Neurodegenerative Diseases by Depletion of Starvation Response Factor Ataxin-2. Trends Neurosci..

[22] Kacher R., Lejeune F.X., David I., Boluda S., Coarelli G., Leclere-Turbant S., Heinzmann A., Marelli C., Charles P., Goizet C. (2024). CAG repeat mosaicism is gene specific in spinocerebellar ataxias. Am. J. Hum. Genet..

[23] Magana J.J., Velazquez-Perez L., Cisneros B. (2013). Spinocerebellar ataxia type 2: Clinical presentation, molecular mechanisms, and therapeutic perspectives. Mol. Neurobiol..

[24] Estrada R., Galarraga J., Orozco G., Nodarse A., Auburger G. (1999). Spinocerebellar ataxia 2 (SCA2): Morphometric analyses in 11 autopsies. Acta Neuropathol..

[25] Varrone A., Salvatore E., De Michele G., Barone P., Sansone V., Pellecchia M.T., Castaldo I., Coppola G., Brunetti A., Salvatore M. (2004). Reduced striatal [123 I]FP-CIT binding in SCA2 patients without parkinsonism. Ann. Neurol..

[26] van de Warrenburg B.P., Notermans N.C., Schelhaas H.J., van Alfen N., Sinke R.J., Knoers N.V., Zwarts M.J., Kremer B.P. (2004). Peripheral nerve involvement in spinocerebellar ataxias. Arch. Neurol..

[27] Lee T., Li Y.R., Ingre C., Weber M., Grehl T., Gredal O., de Carvalho M., Meyer T., Tysnes O.B., Auburger G. (2011). Ataxin-2 intermediate-length polyglutamine expansions in European ALS patients. Hum. Mol. Genet..

[28] Gispert S., Kurz A., Waibel S., Bauer P., Liepelt I., Geisen C., Gitler A.D., Becker T., Weber M., Berg D. (2012). The modulation of Amyotrophic Lateral Sclerosis risk by ataxin-2 intermediate polyglutamine expansions is a specific effect. Neurobiol. Dis..

[29] Lahut S., Omur O., Uyan O., Agim Z.S., Ozoguz A., Parman Y., Deymeer F., Oflazer P., Koc F., Ozcelik H. (2012). ATXN2 and its neighbouring gene SH2B3 are associated with increased ALS risk in the Turkish population. PLoS ONE.

[30] Velazquez-Perez L., Tunnerhoff J., Rodriguez-Labrada R., Torres-Vega R., Ruiz-Gonzalez Y., Belardinelli P., Medrano-Montero J., Canales-Ochoa N., Gonzalez-Zaldivar Y., Vazquez-Mojena Y. (2017). Early corticospinal tract damage in prodromal SCA2 revealed by EEG-EMG and EMG-EMG coherence. Clin. Neurophysiol..

[31] Velazquez-Perez L., Rodriguez-Labrada R., Torres-Vega R., Ortega-Sanchez R., Medrano-Montero J., Gonzalez-Pina R., Vazquez-Mojena Y., Auburger G., Ziemann U. (2018). Progression of corticospinal tract dysfunction in pre-ataxic spinocerebellar ataxia type 2: A two-years follow-up TMS study. Clin. Neurophysiol..

[32] Velazquez-Perez L., Rodriguez-Labrada R., Canales-Ochoa N., Montero J.M., Sanchez-Cruz G., Aguilera-Rodriguez R., Almaguer-Mederos L.E., Laffita-Mesa J.M. (2014). Progression of early features of spinocerebellar ataxia type 2 in individuals at risk: A longitudinal study. Lancet Neurol..

[33] Jacobi H., du Montcel S.T., Bauer P., Giunti P., Cook A., Labrum R., Parkinson M.H., Durr A., Brice A., Charles P. (2015). Long-term disease progression in spinocerebellar ataxia types 1, 2, 3, and 6: A longitudinal cohort study. Lancet Neurol..

[34] Velazquez-Perez L., Medrano-Montero J., Rodriguez-Labrada R., Canales-Ochoa N., Campins Ali J., Carrillo Rodes F.J., Rodriguez Grana T., Hernandez Oliver M.O., Aguilera Rodriguez R., Dominguez Barrios Y. (2020). Hereditary Ataxias in Cuba: A Nationwide Epidemiological and Clinical Study in 1001 Patients. Cerebellum.

[35] Rive Le Gouard N., Bah M.G., Coarelli G., Heinzmann A., Fauret A.L., de Sainte-Agathe J.M., Cazeneuve C., Gerasimenko A., Gras D., Capri Y. (2025). The Two Faces of Pediatric SCA2. Eur. J. Neurol..

[36] Charles P., Camuzat A., Benammar N., Sellal F., Destee A., Bonnet A.M., Lesage S., Le Ber I., Stevanin G., Durr A. (2007). Are interrupted SCA2 CAG repeat expansions responsible for parkinsonism?. Neurology.

[37] Kim J.M., Hong S., Kim G.P., Choi Y.J., Kim Y.K., Park S.S., Kim S.E., Jeon B.S. (2007). Importance of low-range CAG expansion and CAA interruption in SCA2 Parkinsonism. Arch. Neurol..

[38] Yu Z., Zhu Y., Chen-Plotkin A.S., Clay-Falcone D., McCluskey L., Elman L., Kalb R.G., Trojanowski J.Q., Lee V.M., Van Deerlin V.M. (2011). PolyQ repeat expansions in ATXN2 associated with ALS are CAA interrupted repeats. PLoS ONE.

[39] Corrado L., Mazzini L., Oggioni G.D., Luciano B., Godi M., Brusco A., D’Alfonso S. (2011). ATXN-2 CAG repeat expansions are interrupted in ALS patients. Hum. Genet..

[40] Wang C., Xu Y., Feng X., Ma J., Xie S., Zhang Y., Tang B.S., Chan P. (2015). Linkage analysis and whole-exome sequencing exclude extra mutations responsible for the parkinsonian phenotype of spinocerebellar ataxia-2. Neurobiol. Aging.

[41] Al-Ramahi I., Perez A.M., Lim J., Zhang M., Sorensen R., de Haro M., Branco J., Pulst S.M., Zoghbi H.Y., Botas J. (2007). dAtaxin-2 mediates expanded Ataxin-1-induced neurodegeneration in a Drosophila model of SCA1. PLoS Genet..

[42] Van Langenhove T., van der Zee J., Engelborghs S., Vandenberghe R., Santens P., Van den Broeck M., Mattheijssens M., Peeters K., Nuytten D., Cras P. (2012). Ataxin-2 polyQ expansions in FTLD-ALS spectrum disorders in Flanders-Belgian cohorts. Neurobiol. Aging.

[43] Neuenschwander A.G., Thai K.K., Figueroa K.P., Pulst S.M. (2014). Amyotrophic lateral sclerosis risk for spinocerebellar ataxia type 2 ATXN2 CAG repeat alleles: A meta-analysis. JAMA Neurol..

[44] Nobrega C., Carmo-Silva S., Albuquerque D., Vasconcelos-Ferreira A., Vijayakumar U.G., Mendonca L., Hirai H., de Almeida L.P. (2015). Re-establishing ataxin-2 downregulates translation of mutant ataxin-3 and alleviates Machado-Joseph disease. Brain.

[45] Rubino E., Mancini C., Boschi S., Ferrero P., Ferrone M., Bianca S., Zucca M., Orsi L., Pinessi L., Govone F. (2019). ATXN2 intermediate repeat expansions influence the clinical phenotype in frontotemporal dementia. Neurobiol. Aging.

[46] Glass J.D., Dewan R., Ding J., Gibbs J.R., Dalgard C., Keagle P.J., Shankaracharya, Garcia-Redondo A., Traynor B.J., Chia R. (2022). ATXN2 intermediate expansions in amyotrophic lateral sclerosis. Brain.

[47] Mijajlovic M., Dragasevic N., Stefanova E., Petrovic I., Svetel M., Kostic V.S. (2008). Transcranial sonography in spinocerebellar ataxia type 2. J. Neurol..

[48] Shibata H., Huynh D.P., Pulst S.M. (2000). A novel protein with RNA-binding motifs interacts with ataxin-2. Hum. Mol. Genet..

[49] Huynh D.P., Yang H.T., Vakharia H., Nguyen D., Pulst S.M. (2003). Expansion of the polyQ repeat in ataxin-2 alters its Golgi localization, disrupts the Golgi complex and causes cell death. Hum. Mol. Genet..

[50] Ng H., Pulst S.M., Huynh D.P. (2007). Ataxin-2 mediated cell death is dependent on domains downstream of the polyQ repeat. Exp. Neurol..

[51] Del Castillo U., Gnazzo M.M., Sorensen Turpin C.G., Nguyen K.C.Q., Semaya E., Lam Y., de Cruz M.A., Bembenek J.N., Hall D.H., Riggs B. (2019). Conserved role for Ataxin-2 in mediating endoplasmic reticulum dynamics. Traffic.

[52] Nonis D., Schmidt M.H.H., van de Loo S., Eich F., Dikic I., Nowock J., Auburger G. (2008). Ataxin-2 associates with the endocytosis complex and affects EGF receptor trafficking. Cell. Signal..

[53] Drost J., Nonis D., Eich F., Leske O., Damrath E., Brunt E.R., Lastres-Becker I., Heumann R., Nowock J., Auburger G. (2013). Ataxin-2 modulates the levels of Grb2 and SRC but not ras signaling. J. Mol. Neurosci..

[54] Bian W., Jiang H., Yao L., Hao W., Wu L., Li X. (2023). A spatially defined human Notch receptor interaction network reveals Notch intracellular storage and Ataxin-2-mediated fast recycling. Cell Rep..

[55] van de Loo S., Eich F., Nonis D., Auburger G., Nowock J. (2009). Ataxin-2 associates with rough endoplasmic reticulum. Exp. Neurol..

[56] Satterfield T.F., Pallanck L.J. (2006). Ataxin-2 and its Drosophila homolog, ATX2, physically assemble with polyribosomes. Hum. Mol. Genet..

[57] Satterfield T.F., Jackson S.M., Pallanck L.J. (2002). A Drosophila homolog of the polyglutamine disease gene SCA2 is a dosage-sensitive regulator of actin filament formation. Genetics.

[58] Ralser M., Nonhoff U., Albrecht M., Lengauer T., Wanker E.E., Lehrach H., Krobitsch S. (2005). Ataxin-2 and huntingtin interact with endophilin-A complexes to function in plastin-associated pathways. Hum. Mol. Genet..

[59] Key J., Almaguer-Mederos L.E., Kandi A.R., Sen N.E., Gispert S., Kopf G., Meierhofer D., Auburger G. (2025). ATXN2L primarily interacts with NUFIP2, the absence of ATXN2L results in NUFIP2 depletion, and the ATXN2-polyQ expansion triggers NUFIP2 accumulation. Neurobiol. Dis..

[60] Stubenvoll M.D., Medley J.C., Irwin M., Song M.H. (2016). ATX-2, The *C. elegans* Ortholog of Human Ataxin-2, Regulates Centrosome Size and Microtubule Dynamics. PLoS Genet..

[61] Del Castillo U., Norkett R., Lu W., Serpinskaya A., Gelfand V.I. (2022). Ataxin-2 is essential for cytoskeletal dynamics and neurodevelopment in Drosophila. iScience.

[62] Boeynaems S., Dorone Y., Zhuang Y., Shabardina V., Huang G., Marian A., Kim G., Sanyal A., Sen N.E., Griffith D. (2023). Poly(A)-binding protein is an ataxin-2 chaperone that regulates biomolecular condensates. Mol. Cell.

[63] Kim S.K., Gelfand V.I. (2025). PolyQ-Expansion of Ataxin-2 Disrupts Microtubule Stability and Impairs Axon Outgrowth. J. Neurosci..

[64] Nonhoff U., Ralser M., Welzel F., Piccini I., Balzereit D., Yaspo M.L., Lehrach H., Krobitsch S. (2007). Ataxin-2 interacts with the DEAD/H-box RNA helicase DDX6 and interferes with P-bodies and stress granules. Mol. Biol. Cell.

[65] Swisher K.D., Parker R. (2010). Localization to, and effects of Pbp1, Pbp4, Lsm12, Dhh1, and Pab1 on stress granules in Saccharomyces cerevisiae. PLoS ONE.

[66] Yamagishi R., Inagaki H., Suzuki J., Hosoda N., Sugiyama H., Tomita K., Hotta T., Hoshino S.I. (2024). Concerted action of ataxin-2 and PABPC1-bound mRNA poly(A) tail in the formation of stress granules. Nucleic Acids Res..

[67] Palozzi J.M., Jeedigunta S.P., Minenkova A.V., Monteiro V.L., Thompson Z.S., Lieber T., Hurd T.R. (2022). Mitochondrial DNA quality control in the female germline requires a unique programmed mitophagy. Cell Metab..

[68] Huynh D.P., Nguyen D.T., Pulst-Korenberg J.B., Brice A., Pulst S.M. (2007). Parkin is an E3 ubiquitin-ligase for normal and mutant ataxin-2 and prevents ataxin-2-induced cell death. Exp. Neurol..

[69] Halbach M.V., Stehning T., Damrath E., Jendrach M., Sen N.E., Basak A.N., Auburger G. (2015). Both ubiquitin ligases FBXW8 and PARK2 are sequestrated into insolubility by ATXN2 PolyQ expansions, but only FBXW8 expression is dysregulated. PLoS ONE.

[70] Sen N.E., Drost J., Gispert S., Torres-Odio S., Damrath E., Klinkenberg M., Hamzeiy H., Akdal G., Gulluoglu H., Basak A.N. (2016). Search for SCA2 blood RNA biomarkers highlights Ataxin-2 as strong modifier of the mitochondrial factor PINK1 levels. Neurobiol. Dis..

[71] O’Neill E.C., Trick M., Hill L., Rejzek M., Dusi R.G., Hamilton C.J., Zimba P.V., Henrissat B., Field R.A. (2015). The transcriptome of Euglena gracilis reveals unexpected metabolic capabilities for carbohydrate and natural product biochemistry. Mol. Biosyst..

[72] Luisi P.L. (1979). Why are enzymes macromolecules?. Naturwissenschaften.

[73] Marijuan P.C., del Moral R., Navarro J. (2013). On eukaryotic intelligence: Signaling system’s guidance in the evolution of multicellular organization. Biosystems.

[74] Toro I., Thore S., Mayer C., Basquin J., Seraphin B., Suck D. (2001). RNA binding in an Sm core domain: X-ray structure and functional analysis of an archaeal Sm protein complex. EMBO J..

[75] Ralser M., Albrecht M., Nonhoff U., Lengauer T., Lehrach H., Krobitsch S. (2005). An integrative approach to gain insights into the cellular function of human ataxin-2. J. Mol. Biol..

[76] Jimenez-Lopez D., Guzman P. (2014). Insights into the evolution and domain structure of Ataxin-2 proteins across eukaryotes. BMC Res. Notes.

[77] Zhang S., Zhang Y., Chen T., Hu H.Y., Lu C. (2025). The LSmAD Domain of Ataxin-2 Modulates the Structure and RNA Binding of Its Preceding LSm Domain. Cells.

[78] Kozlov G., De Crescenzo G., Lim N.S., Siddiqui N., Fantus D., Kahvejian A., Trempe J.F., Elias D., Ekiel I., Sonenberg N. (2004). Structural basis of ligand recognition by PABC, a highly specific peptide-binding domain found in poly(A)-binding protein and a HECT ubiquitin ligase. EMBO J..

[79] Huang K.L., Chadee A.B., Chen C.Y., Zhang Y., Shyu A.B. (2013). Phosphorylation at intrinsically disordered regions of PAM2 motif-containing proteins modulates their interactions with PABPC1 and influences mRNA fate. RNA.

[80] Kozlov G., Safaee N., Rosenauer A., Gehring K. (2010). Structural basis of binding of P-body-associated proteins GW182 and ataxin-2 by the Mlle domain of poly(A)-binding protein. J. Biol. Chem..

[81] Damrath E., Heck M.V., Gispert S., Azizov M., Nowock J., Seifried C., Rub U., Walter M., Auburger G. (2012). ATXN2-CAG42 sequesters PABPC1 into insolubility and induces FBXW8 in cerebellum of old ataxic knock-in mice. PLoS Genet..

[82] Albrecht M., Lengauer T. (2004). Survey on the PABC recognition motif PAM2. Biochem. Biophys. Res. Commun..

[83] Petrauskas A., Fortunati D.L., Kandi A.R., Pothapragada S.S., Agrawal K., Singh A., Huelsmeier J., Hillebrand J., Brown G., Chaturvedi D. (2024). Structured and disordered regions of Ataxin-2 contribute differently to the specificity and efficiency of mRNP granule formation. PLoS Genet..

[84] Obornik M., Lukes J. (2013). Cell biology of chromerids: Autotrophic relatives to apicomplexan parasites. Int. Rev. Cell Mol. Biol..

[85] Cavalier-Smith T. (2003). The excavate protozoan phyla Metamonada Grasse emend. (Anaeromonadea, Parabasalia, Carpediemonas, Eopharyngia) and Loukozoa emend. (Jakobea, Malawimonas): Their evolutionary affinities and new higher taxa. Int. J. Syst. Evol. Microbiol..

[86] Hannaert V., Saavedra E., Duffieux F., Szikora J.P., Rigden D.J., Michels P.A., Opperdoes F.R. (2003). Plant-like traits associated with metabolism of Trypanosoma parasites. Proc. Natl. Acad. Sci. USA.

[87] Whitfield J. (2003). Sleeping sickness bug swallowed a plant. Nature.

[88] Karnkowska A., Vacek V., Zubacova Z., Treitli S.C., Petrzelkova R., Eme L., Novak L., Zarsky V., Barlow L.D., Herman E.K. (2016). A Eukaryote without a Mitochondrial Organelle. Curr. Biol..

[89] Burri L., Williams B.A., Bursac D., Lithgow T., Keeling P.J. (2006). Microsporidian mitosomes retain elements of the general mitochondrial targeting system. Proc. Natl. Acad. Sci. USA.

[90] Nowack E.C.M., Weber A.P.M. (2018). Genomics-Informed Insights into Endosymbiotic Organelle Evolution in Photosynthetic Eukaryotes. Annu. Rev. Plant Biol..

[91] Kim E., Graham L.E. (2008). EEF2 analysis challenges the monophyly of Archaeplastida and Chromalveolata. PLoS ONE.

[92] Keeling P.J. (2010). The endosymbiotic origin, diversification and fate of plastids. Philos. Trans. R. Soc. Lond. B Biol. Sci..

[93] Keeling P.J. (2004). Diversity and evolutionary history of plastids and their hosts. Am. J. Bot..

[94] Ponce-Toledo R.I., Deschamps P., Lopez-Garcia P., Zivanovic Y., Benzerara K., Moreira D. (2017). An Early-Branching Freshwater Cyanobacterium at the Origin of Plastids. Curr. Biol..

[95] Figueroa-Martinez F., Jackson C., Reyes-Prieto A. (2019). Plastid Genomes from Diverse Glaucophyte Genera Reveal a Largely Conserved Gene Content and Limited Architectural Diversity. Genome Biol. Evol..

[96] Lemieux C., Otis C., Turmel M. (2000). Ancestral chloroplast genome in Mesostigma viride reveals an early branch of green plant evolution. Nature.

[97] Lee R.E. (2018). Chapter 5: Chlorophyta. Phycology.

[98] Miyagishima S.Y. (2023). Taming the perils of photosynthesis by eukaryotes: Constraints on endosymbiotic evolution in aquatic ecosystems. Commun. Biol..

[99] Woelkerling W.J. (1990). An Introduction. Biology of Red Algae.

[100] Rohmer M., Bouvier P., Ourisson G. (1979). Molecular evolution of biomembranes: Structural equivalents and phylogenetic precursors of sterols. Proc. Natl. Acad. Sci. USA.

[101] Ourisson G., Nakatani Y. (1994). The terpenoid theory of the origin of cellular life: The evolution of terpenoids to cholesterol. Chem. Biol..

[102] Wu F., Janvier P., Zhang C. (2023). The rise of predation in Jurassic lampreys. Nat. Commun..

[103] Meyer A., Schartl M. (1999). Gene and genome duplications in vertebrates: The one-to-four (-to-eight in fish) rule and the evolution of novel gene functions. Curr. Opin. Cell Biol..

[104] Schilling T.F., Knight R.D. (2001). Origins of anteroposterior patterning and Hox gene regulation during chordate evolution. Philos. Trans. R. Soc. Lond. B Biol. Sci..

[105] Moens C.B., Prince V.E. (2002). Constructing the hindbrain: Insights from the zebrafish. Dev. Dyn..

[106] Wilson L., Maden M. (2005). The mechanisms of dorsoventral patterning in the vertebrate neural tube. Dev. Biol..

[107] Lupo G., Harris W.A., Lewis K.E. (2006). Mechanisms of ventral patterning in the vertebrate nervous system. Nat. Rev. Neurosci..

[108] Marletaz F., Holland L.Z., Laudet V., Schubert M. (2006). Retinoic acid signaling and the evolution of chordates. Int. J. Biol. Sci..

[109] Kuraku S. (2010). Palaeophylogenomics of the vertebrate ancestor--impact of hidden paralogy on hagfish and lamprey gene phylogeny. Integr. Comp. Biol..

[110] Campo-Paysaa F., Jandzik D., Takio-Ogawa Y., Cattell M.V., Neef H.C., Langeland J.A., Kuratani S., Medeiros D.M., Mazan S., Kuraku S. (2015). Evolution of retinoic acid receptors in chordates: Insights from three lamprey species, Lampetra fluviatilis, Petromyzon marinus, and Lethenteron japonicum. Evodevo.

[111] Handberg-Thorsager M., Gutierrez-Mazariegos J., Arold S.T., Kumar Nadendla E., Bertucci P.Y., Germain P., Tomancak P., Pierzchalski K., Jones J.W., Albalat R. (2018). The ancestral retinoic acid receptor was a low-affinity sensor triggering neuronal differentiation. Sci. Adv..

[112] Bedois A.M.H., Parker H.J., Krumlauf R. (2021). Retinoic Acid Signaling in Vertebrate Hindbrain Segmentation: Evolution and Diversification. Diversity.

[113] Qian X., Riccio A., Zhang Y., Ginty D.D. (1998). Identification and characterization of novel substrates of Trk receptors in developing neurons. Neuron.

[114] Joe R.M., Flores A., Doche M.E., Cline J.M., Clutter E.S., Vander P.B., Riedel H., Argetsinger L.S., Carter-Su C. (2018). Phosphorylation of the Unique C-Terminal Tail of the Alpha Isoform of the Scaffold Protein SH2B1 Controls the Ability of SH2B1alpha To Enhance Nerve Growth Factor Function. Mol. Cell. Biol..

[115] Wang T.C., Chiu H., Chang Y.J., Hsu T.Y., Chiu I.M., Chen L. (2011). The adaptor protein SH2B3 (Lnk) negatively regulates neurite outgrowth of PC12 cells and cortical neurons. PLoS ONE.

[116] Lin L., Li X., Pan C., Lin W., Shao R., Liu Y., Zhang J., Luo Y., Qian K., Shi M. (2019). ATXN2L upregulated by epidermal growth factor promotes gastric cancer cell invasiveness and oxaliplatin resistance. Cell Death Dis..

[117] Auburger G., Gispert S., Lahut S., Omur O., Damrath E., Heck M., Basak N. (2014). 12q24 locus association with type 1 diabetes: SH2B3 or ATXN2?. World J. Diabetes.

[118] Li L., Wang M., Huang L., Zheng X., Wang L., Miao H. (2024). Ataxin-2: A powerful RNA-binding protein. Discov. Oncol..

[119] Zhang G., Li C., Li Q., Li B., Larkin D.M., Lee C., Storz J.F., Antunes A., Greenwold M.J., Meredith R.W. (2014). Comparative genomics reveals insights into avian genome evolution and adaptation. Science.

[120] Jiao Y., Wickett N.J., Ayyampalayam S., Chanderbali A.S., Landherr L., Ralph P.E., Tomsho L.P., Hu Y., Liang H., Soltis P.S. (2011). Ancestral polyploidy in seed plants and angiosperms. Nature.

[121] Zhang Z., Mistry D., Jetter R. (2024). Micromorphological and Chemical Characterization of Drimys winteri Leaf Surfaces: The Secondary Alcohols Forming Epicuticular Wax Crystals Are Accompanied by Alkanediol, Alkanetriol and Ketol Derivatives. Plant Cell Physiol..

[122] Chang C.Y., Brautigam K., Huner N.P.A., Ensminger I. (2021). Champions of winter survival: Cold acclimation and molecular regulation of cold hardiness in evergreen conifers. New Phytol..

[123] Rice D.W., Alverson A.J., Richardson A.O., Young G.J., Sanchez-Puerta M.V., Munzinger J., Barry K., Boore J.L., Zhang Y., dePamphilis C.W. (2013). Horizontal transfer of entire genomes via mitochondrial fusion in the angiosperm Amborella. Science.

[124] Lastres-Becker I., Nonis D., Nowock J., Auburger G. (2019). New alternative splicing variants of the ATXN2 transcript. Neurol. Res. Pract..

[125] Nechiporuk T., Huynh D.P., Figueroa K., Sahba S., Nechiporuk A., Pulst S.M. (1998). The mouse SCA2 gene: cDNA sequence, alternative splicing and protein expression. Hum. Mol. Genet..

[126] Affaitati A., de Cristofaro T., Feliciello A., Varrone S. (2001). Identification of alternative splicing of spinocerebellar ataxia type 2 gene. Gene.

[127] Scoles D.R., Pflieger L.T., Thai K.K., Hansen S.T., Dansithong W., Pulst S.M. (2012). ETS1 regulates the expression of ATXN2. Hum. Mol. Genet..

[128] Key J., Harter P.N., Sen N.E., Gradhand E., Auburger G., Gispert S. (2020). Mid-Gestation lethality of Atxn2l-Ablated Mice. Int. J. Mol. Sci..

[129] Key J., Almaguer-Mederos L.E., Kandi A.R., Fellenz M., Gispert S., Kopf G., Meierhofer D., Deller T., Auburger G. (2025). Conditional ATXN2L-Null in Adult Frontal Cortex CamK2a+ Neurons Does Not Cause Cell Death but Restricts Spontaneous Mobility and Affects the Alternative Splicing Pathway. Cells.

[130] Sen N.E., Canet-Pons J., Halbach M.V., Arsovic A., Pilatus U., Chae W.H., Kaya Z.E., Seidel K., Rollmann E., Mittelbronn M. (2019). Generation of an Atxn2-CAG100 knock-in mouse reveals N-acetylaspartate production deficit due to early Nat8l dysregulation. Neurobiol. Dis..

[131] Almaguer-Mederos L.E., Kandi A.R., Sen N.E., Canet-Pons J., Berger L.M., Stokes M.P., Abell K., Key J., Gispert S., Auburger G. (2025). Spinal Cord Phosphoproteome of SCA2 Mouse Model Reveals Alteration of ATXN2-N-Term PRM-SH3-Actin Interactome and of Autophagy. Mol. Cell. Proteomics.

[132] Inada R., Hirano M., Oka N., Samukawa M., Saigoh K., Suzuki H., Udaka F., Hashiguchi A., Takashima H., Hamada Y. (2021). Phenotypic and molecular diversities of spinocerebellar ataxia type 2 in Japan. J. Neurol..

[133] Laffita-Mesa J.M., Nennesmo I., Paucar M., Svenningsson P. (2021). A Novel Duplication in ATXN2 as Modifier for Spinocerebellar Ataxia 3 (SCA3) and C9ORF72-ALS. Mov. Disord..

[134] Aguiar J., Santurlidis S., Nowok J., Alexander C., Rudnicki D., Gispert S., Schulz W., Auburger G. (1999). Identification of the physiological promoter for spinocerebellar ataxia 2 gene reveals a CpG island for promoter activity situated into the exon 1 of this gene and provides data about the origin of the nonmethylated state of these types of islands. Biochem. Biophys. Res. Commun..

[135] Morgan A.A., Rubenstein E. (2013). Proline: The distribution, frequency, positioning, and common functional roles of proline and polyproline sequences in the human proteome. PLoS ONE.

[136] Mandal A., Mandal S., Park M.H. (2014). Genome-wide analyses and functional classification of proline repeat-rich proteins: Potential role of eIF5A in eukaryotic evolution. PLoS ONE.

[137] Lastres-Becker I., Brodesser S., Lutjohann D., Azizov M., Buchmann J., Hintermann E., Sandhoff K., Schurmann A., Nowock J., Auburger G. (2008). Insulin receptor and lipid metabolism pathology in ataxin-2 knock-out mice. Hum. Mol. Genet..

[138] van de Poll F., Sutter B.M., Acoba M.G., Caballero D., Jahangiri S., Yang Y.S., Lee C.D., Tu B.P. (2023). Pbp1 associates with Puf3 and promotes translation of its target mRNAs involved in mitochondrial biogenesis. PLoS Genet..

[139] Tuong Vi D.T., Fujii S., Valderrama A.L., Ito A., Matsuura E., Nishihata A., Irie K., Suda Y., Mizuno T., Irie K. (2021). Pbp1, the yeast ortholog of human Ataxin-2, functions in the cell growth on non-fermentable carbon sources. PLoS ONE.

[140] Cornelius N., Wardman J.H., Hargreaves I.P., Neergheen V., Bie A.S., Tumer Z., Nielsen J.E., Nielsen T.T. (2017). Evidence of oxidative stress and mitochondrial dysfunction in spinocerebellar ataxia type 2 (SCA2) patient fibroblasts: Effect of coenzyme Q10 supplementation on these parameters. Mitochondrion.

[141] Seidel G., Meierhofer D., Sen N.E., Guenther A., Krobitsch S., Auburger G. (2017). Quantitative Global Proteomics of Yeast PBP1 Deletion Mutants and Their Stress Responses Identifies Glucose Metabolism, Mitochondrial, and Stress Granule Changes. J. Proteome Res..

[142] Chitre M., Emery P. (2023). ATXN2 is a target of N-terminal proteolysis. PLoS ONE.

[143] Leitgeb B., Kerenyi A., Bogar F., Paragi G., Penke B., Rakhely G. (2007). Studying the structural properties of polyalanine and polyglutamine peptides. J. Mol. Model..

[144] Brown L.Y., Brown S.A. (2004). Alanine tracts: The expanding story of human illness and trinucleotide repeats. Trends Genet..

[145] Kurokawa R., Kurokawa M., Mitsutake A., Nakaya M., Baba A., Nakata Y., Moritani T., Abe O. (2023). Clinical and neuroimaging review of triplet repeat diseases. Jpn. J. Radiol..

[146] Gillon A.D., Latham C.F., Miller E.A. (2012). Vesicle-mediated ER export of proteins and lipids. Biochim. Biophys. Acta.

[147] Vianna M.C., Poleto D.C., Gomes P.F., Valente V., Paco-Larson M.L. (2016). Drosophila ataxin-2 gene encodes two differentially expressed isoforms and its function in larval fat body is crucial for development of peripheral tissues. FEBS Open Bio..

[148] Sen N.E., Arsovic A., Meierhofer D., Brodesser S., Oberschmidt C., Canet-Pons J., Kaya Z.E., Halbach M.V., Gispert S., Sandhoff K. (2019). In Human and Mouse Spino-Cerebellar Tissue, Ataxin-2 Expansion Affects Ceramide-Sphingomyelin Metabolism. Int. J. Mol. Sci..

[149] Watanabe R., Higashi S., Nonaka T., Kawakami I., Oshima K., Niizato K., Akiyama H., Yoshida M., Hasegawa M., Arai T. (2020). Intracellular dynamics of Ataxin-2 in the human brains with normal and frontotemporal lobar degeneration with TDP-43 inclusions. Acta Neuropathol. Commun..

[150] Canet-Pons J., Sen N.E., Arsovic A., Almaguer-Mederos L.E., Halbach M.V., Key J., Doring C., Kerksiek A., Picchiarelli G., Cassel R. (2021). Atxn2-CAG100-KnockIn mouse spinal cord shows progressive TDP43 pathology associated with cholesterol biosynthesis suppression. Neurobiol. Dis..

[151] Torricella F., Tugarinov V., Clore G.M. (2024). Nucleation of Huntingtin Aggregation Proceeds via Conformational Conversion of Pre-Formed, Sparsely-Populated Tetramers. Adv. Sci..

[152] Zhang L., Kang H., Perez-Aguilar J.M., Zhou R. (2022). Possible Co-Evolution of Polyglutamine and Polyproline in Huntingtin Protein: Proline-Rich Domain as Transient Folding Chaperone. J. Phys. Chem. Lett..

[153] Darnell G., Orgel J.P., Pahl R., Meredith S.C. (2007). Flanking polyproline sequences inhibit beta-sheet structure in polyglutamine segments by inducing PPII-like helix structure. J. Mol. Biol..

[154] Ceccon A., Tugarinov V., Clore G.M. (2021). Quantitative Exchange NMR-Based Analysis of Huntingtin-SH3 Interactions Suggests an Allosteric Mechanism of Inhibition of Huntingtin Aggregation. J. Am. Chem. Soc..

[155] Nagarajan A., Jawahery S., Matysiak S. (2014). The effects of flanking sequences in the interaction of polyglutamine peptides with a membrane bilayer. J. Phys. Chem. B.

[156] Ramirez de Mingo D., Pantoja-Uceda D., Hervas R., Carrion-Vazquez M., Laurents D.V. (2022). Conformational dynamics in the disordered region of human CPEB3 linked to memory consolidation. BMC Biol..

[157] Gerber H.P., Seipel K., Georgiev O., Hofferer M., Hug M., Rusconi S., Schaffner W. (1994). Transcriptional activation modulated by homopolymeric glutamine and proline stretches. Science.

[158] Achsel T., Stark H., Luhrmann R. (2001). The Sm domain is an ancient RNA-binding motif with oligo(U) specificity. Proc. Natl. Acad. Sci. USA.

[159] Sobti M., Cubeddu L., Haynes P.A., Mabbutt B.C. (2010). Engineered rings of mixed yeast Lsm proteins show differential interactions with translation factors and U-rich RNA. Biochemistry.

[160] Schumacher M.A., Pearson R.F., Moller T., Valentin-Hansen P., Brennan R.G. (2002). Structures of the pleiotropic translational regulator Hfq and an Hfq-RNA complex: A bacterial Sm-like protein. EMBO J..

[161] Zhang A., Wassarman K.M., Rosenow C., Tjaden B.C., Storz G., Gottesman S. (2003). Global analysis of small RNA and mRNA targets of Hfq. Mol. Microbiol..

[162] Folichon M., Arluison V., Pellegrini O., Huntzinger E., Regnier P., Hajnsdorf E. (2003). The poly(A) binding protein Hfq protects RNA from RNase E and exoribonucleolytic degradation. Nucleic Acids Res..

[163] Brennan R.G., Link T.M. (2007). Hfq structure, function and ligand binding. Curr. Opin. Microbiol..

[164] Sittka A., Lucchini S., Papenfort K., Sharma C.M., Rolle K., Binnewies T.T., Hinton J.C., Vogel J. (2008). Deep sequencing analysis of small noncoding RNA and mRNA targets of the global post-transcriptional regulator, Hfq. PLoS Genet..

[165] Padalon-Brauch G., Hershberg R., Elgrably-Weiss M., Baruch K., Rosenshine I., Margalit H., Altuvia S. (2008). Small RNAs encoded within genetic islands of Salmonella typhimurium show host-induced expression and role in virulence. Nucleic Acids Res..

[166] Link T.M., Valentin-Hansen P., Brennan R.G. (2009). Structure of Escherichia coli Hfq bound to polyriboadenylate RNA. Proc. Natl. Acad. Sci. USA.

[167] Lorenz C., Gesell T., Zimmermann B., Schoeberl U., Bilusic I., Rajkowitsch L., Waldsich C., von Haeseler A., Schroeder R. (2010). Genomic SELEX for Hfq-binding RNAs identifies genomic aptamers predominantly in antisense transcripts. Nucleic Acids Res..

[168] Otaka H., Ishikawa H., Morita T., Aiba H. (2011). PolyU tail of rho-independent terminator of bacterial small RNAs is essential for Hfq action. Proc. Natl. Acad. Sci. USA.

[169] Horstmann N., Orans J., Valentin-Hansen P., Shelburne S.A., Brennan R.G. (2012). Structural mechanism of Staphylococcus aureus Hfq binding to an RNA A-tract. Nucleic Acids Res..

[170] Zeng Q., Sundin G.W. (2014). Genome-wide identification of Hfq-regulated small RNAs in the fire blight pathogen Erwinia amylovora discovered small RNAs with virulence regulatory function. BMC Genom..

[171] Reichelt R., Rothmeier T., Grunberger F., Willkomm S., Bruckmann A., Hausner W., Grohmann D. (2023). The archaeal Lsm protein from Pyrococcus furiosus binds co-transcriptionally to poly(U)-rich target RNAs. Biol. Chem..

[172] Guisbert E., Rhodius V.A., Ahuja N., Witkin E., Gross C.A. (2007). Hfq modulates the sigmaE-mediated envelope stress response and the sigma32-mediated cytoplasmic stress response in Escherichia coli. J. Bacteriol..

[173] Diestra E., Cayrol B., Arluison V., Risco C. (2009). Cellular electron microscopy imaging reveals the localization of the Hfq protein close to the bacterial membrane. PLoS ONE.

[174] Andrade J.M., Dos Santos R.F., Chelysheva I., Ignatova Z., Arraiano C.M. (2018). The RNA-binding protein Hfq is important for ribosome biogenesis and affects translation fidelity. EMBO J..

[175] Sharma I.M., Korman A., Woodson S.A. (2018). The Hfq chaperone helps the ribosome mature. EMBO J..

[176] Figueroa-Bossi N., Lemire S., Maloriol D., Balbontin R., Casadesus J., Bossi L. (2006). Loss of Hfq activates the sigmaE-dependent envelope stress response in Salmonella enterica. Mol. Microbiol..

[177] Johansen J., Rasmussen A.A., Overgaard M., Valentin-Hansen P. (2006). Conserved small non-coding RNAs that belong to the sigmaE regulon: Role in down-regulation of outer membrane proteins. J. Mol. Biol..

[178] Wang M.C., Chien H.F., Tsai Y.L., Liu M.C., Liaw S.J. (2014). The RNA chaperone Hfq is involved in stress tolerance and virulence in uropathogenic Proteus mirabilis. PLoS ONE.

[179] Vogt S.L., Raivio T.L. (2014). Hfq reduces envelope stress by controlling expression of envelope-localized proteins and protein complexes in enteropathogenic Escherichia coli. Mol. Microbiol..

[180] Deng Y., Chen C., Zhao Z., Zhao J., Jacq A., Huang X., Yang Y. (2016). The RNA Chaperone Hfq Is Involved in Colony Morphology, Nutrient Utilization and Oxidative and Envelope Stress Response in Vibrio alginolyticus. PLoS ONE.

[181] Gottesman S. (2019). Trouble is coming: Signaling pathways that regulate general stress responses in bacteria. J. Biol. Chem..

[182] Tobias N.J., Heinrich A.K., Eresmann H., Wright P.R., Neubacher N., Backofen R., Bode H.B. (2017). Photorhabdus-nematode symbiosis is dependent on hfq-mediated regulation of secondary metabolites. Environ. Microbiol..

[183] He W., Parker R. (2000). Functions of Lsm proteins in mRNA degradation and splicing. Curr. Opin. Cell Biol..

[184] Friesen W.J., Dreyfuss G. (2000). Specific sequences of the Sm and Sm-like (Lsm) proteins mediate their interaction with the spinal muscular atrophy disease gene product (SMN). J. Biol. Chem..

[185] Fromont-Racine M., Mayes A.E., Brunet-Simon A., Rain J.C., Colley A., Dix I., Decourty L., Joly N., Ricard F., Beggs J.D. (2000). Genome-wide protein interaction screens reveal functional networks involving Sm-like proteins. Yeast.

[186] Tritschler F., Eulalio A., Helms S., Schmidt S., Coles M., Weichenrieder O., Izaurralde E., Truffault V. (2008). Similar modes of interaction enable Trailer Hitch and EDC3 to associate with DCP1 and Me31B in distinct protein complexes. Mol. Cell. Biol..

[187] Tang W., Kannan R., Blanchette M., Baumann P. (2012). Telomerase RNA biogenesis involves sequential binding by Sm and Lsm complexes. Nature.

[188] Mura C., Randolph P.S., Patterson J., Cozen A.E. (2013). Archaeal and eukaryotic homologs of Hfq: A structural and evolutionary perspective on Sm function. RNA Biol..

[189] Reimer K.A., Stark M.R., Aguilar L.C., Stark S.R., Burke R.D., Moore J., Fahlman R.P., Yip C.K., Kuroiwa H., Oeffinger M. (2017). The sole LSm complex in Cyanidioschyzon merolae associates with pre-mRNA splicing and mRNA degradation factors. RNA.

[190] Brandmann T., Fakim H., Padamsi Z., Youn J.Y., Gingras A.C., Fabian M.R., Jinek M. (2018). Molecular architecture of LSM14 interactions involved in the assembly of mRNA silencing complexes. EMBO J..

[191] Lekontseva N.V., Stolboushkina E.A., Nikulin A.D. (2021). Diversity of LSM Family Proteins: Similarities and Differences. Biochemistry.

[192] Zhang J., Guan X., Shah K., Yan J. (2021). Lsm12 is an NAADP receptor and a two-pore channel regulatory protein required for calcium mobilization from acidic organelles. Nat. Commun..

[193] Paya G., Bautista V., Pastor-Soler S., Camacho M., Esclapez J., Bonete M.J. (2024). Analysis of Lsm Protein-Mediated Regulation in the Haloarchaeon Haloferax mediterranei. Int. J. Mol. Sci..

[194] Chen Q., Chen Y., Zheng Q. (2025). The RNA-binding protein LSM family regulating reproductive development via different RNA metabolism. Biochim. Biophys. Acta Mol. Basis Dis..

[195] Yang X.C., Desotell A., Lin M.H., Paige A.S., Malinowska A., Sun Y., Aik W.S., Dadlez M., Tong L., Dominski Z. (2023). In vitro methylation of the U7 snRNP subunits Lsm11 and SmE by the PRMT5/MEP50/pICln methylosome. RNA.

[196] Albrecht M., Lengauer T. (2004). Novel Sm-like proteins with long C-terminal tails and associated methyltransferases. FEBS Lett..

[197] Yokoshi M., Li Q., Yamamoto M., Okada H., Suzuki Y., Kawahara Y. (2014). Direct binding of Ataxin-2 to distinct elements in 3′ UTRs promotes mRNA stability and protein expression. Mol. Cell.

[198] Wang J.Y., Liu Y.J., Zhang X.L., Liu Y.H., Jiang L.L., Hu H.Y. (2024). PolyQ-expanded ataxin-2 aggregation impairs cellular processing-body homeostasis via sequestering the RNA helicase DDX6. J. Biol. Chem..

[199] Scoles D.R., Ho M.H., Dansithong W., Pflieger L.T., Petersen L.W., Thai K.K., Pulst S.M. (2015). Repeat Associated Non-AUG Translation (RAN Translation) Dependent on Sequence Downstream of the ATXN2 CAG Repeat. PLoS ONE.

[200] Bruner S.D., Norman D.P., Verdine G.L. (2000). Structural basis for recognition and repair of the endogenous mutagen 8-oxoguanine in DNA. Nature.

[201] Nash H.M., Bruner S.D., Scharer O.D., Kawate T., Addona T.A., Spooner E., Lane W.S., Verdine G.L. (1996). Cloning of a yeast 8-oxoguanine DNA glycosylase reveals the existence of a base-excision DNA-repair protein superfamily. Curr. Biol..

[202] Wang Q., Hobbs K., Lynn B., Rymond B.C. (2003). The Clf1p splicing factor promotes spliceosome assembly through N-terminal tetratricopeptide repeat contacts. J. Biol. Chem..

[203] Yan F., LaMarre J.M., Rohrich R., Wiesner J., Jomaa H., Mankin A.S., Fujimori D.G. (2010). RlmN and Cfr are radical SAM enzymes involved in methylation of ribosomal RNA. J. Am. Chem. Soc..

[204] Bohnsack K.E., Hobartner C., Bohnsack M.T. (2019). Eukaryotic 5-methylcytosine (m^5^C) RNA Methyltransferases: Mechanisms, Cellular Functions, and Links to Disease. Genes.

[205] Haigis M.C., Kurten E.L., Raines R.T. (2003). Ribonuclease inhibitor as an intracellular sentry. Nucleic Acids Res..

[206] Kazan K. (2017). The Multitalented MEDIATOR25. Front. Plant Sci..

[207] Maurice F., Perebaskine N., Thore S., Fribourg S. (2019). In vitro dimerization of human RIO2 kinase. RNA Biol..

[208] Nithianandam V., Sarkar S., Feany M.B. (2024). Pathways controlling neurotoxicity and proteostasis in mitochondrial complex I deficiency. Hum. Mol. Genet..

[209] Ghosh S., Nguyen M.T., Choi H.E., Stahl M., Kuhn A.L., Van der Auwera S., Grabe H.J., Volzke H., Homuth G., Myers S.A. (2024). RIOK2 transcriptionally regulates TRiC and dyskerin complexes to prevent telomere shortening. Nat. Commun..

[210] D’Amora D.R., Hu Q., Pizzardi M., Kubiseski T.J. (2018). BRAP-2 promotes DNA damage induced germline apoptosis in *C. elegans* through the regulation of SKN-1 and AKT-1. Cell Death Differ..

[211] Sakai H., Shiina I., Shinomiya T., Nagahara Y. (2021). BRAP2 inhibits the Ras/Raf/MEK and PI3K/Akt pathways in leukemia cells, thereby inducing apoptosis and inhibiting cell growth. Exp. Ther. Med..

[212] Li S., Ku C.Y., Farmer A.A., Cong Y.S., Chen C.F., Lee W.H. (1998). Identification of a novel cytoplasmic protein that specifically binds to nuclear localization signal motifs. J. Biol. Chem..

[213] Bakthavachalu B., Huelsmeier J., Sudhakaran I.P., Hillebrand J., Singh A., Petrauskas A., Thiagarajan D., Sankaranarayanan M., Mizoue L., Anderson E.N. (2018). RNP-Granule Assembly via Ataxin-2 Disordered Domains Is Required for Long-Term Memory and Neurodegeneration. Neuron.

[214] Bravo J., Aguilar-Henonin L., Olmedo G., Guzman P. (2005). Four distinct classes of proteins as interaction partners of the PABC domain of Arabidopsis thaliana Poly(A)-binding proteins. Mol. Genet. Genom..

[215] Xie J., Kozlov G., Gehring K. (2014). The “tale” of poly(A) binding protein: The MLLE domain and PAM2-containing proteins. Biochim. Biophys. Acta.

[216] Singh A., Hulsmeier J., Kandi A.R., Pothapragada S.S., Hillebrand J., Petrauskas A., Agrawal K., Rt K., Thiagarajan D., Jayaprakashappa D. (2021). Antagonistic roles for Ataxin-2 structured and disordered domains in RNP condensation. Elife.

[217] Jimenez-Lopez D., Bravo J., Guzman P. (2015). Evolutionary history exposes radical diversification among classes of interaction partners of the MLLE domain of plant poly(A)-binding proteins. BMC Evol. Biol..

[218] McCarty J., Delaney K.T., Danielsen S.P.O., Fredrickson G.H., Shea J.E. (2019). Complete Phase Diagram for Liquid-Liquid Phase Separation of Intrinsically Disordered Proteins. J. Phys. Chem. Lett..

[219] Scheidt T., Ruan H., Yu M., Lemke E.A. (2023). Stressing the role of a short linear motif in ataxin-2 condensation. Mol. Cell.

[220] Yang Y.S., Kato M., Wu X., Litsios A., Sutter B.M., Wang Y., Hsu C.H., Wood N.E., Lemoff A., Mirzaei H. (2019). Yeast Ataxin-2 Forms an Intracellular Condensate Required for the Inhibition of TORC1 Signaling during Respiratory Growth. Cell.

[221] Kato M., Yang Y.S., Sutter B.M., Wang Y., McKnight S.L., Tu B.P. (2019). Redox State Controls Phase Separation of the Yeast Ataxin-2 Protein via Reversible Oxidation of Its Methionine-Rich Low-Complexity Domain. Cell.

[222] Prouteau M., Loewith R. (2019). TOR Signaling Is Going through a Phase. Cell Metab..

[223] Huelsmeier J., Walker E., Bakthavachalu B., Ramaswami M. (2021). A C-terminal ataxin-2 disordered region promotes Huntingtin protein aggregation and neurodegeneration in Drosophila models of Huntington’s disease. G3.

[224] Wijegunawardana D., Nayak A., Vishal S.S., Venkatesh N., Gopal P.P. (2025). Ataxin-2 polyglutamine expansions aberrantly sequester TDP-43 ribonucleoprotein condensates disrupting mRNA transport and local translation in neurons. Dev. Cell.

[225] Salama N.R., Chuang J.S., Schekman R.W. (1997). Sec31 encodes an essential component of the COPII coat required for transport vesicle budding from the endoplasmic reticulum. Mol. Biol. Cell.

[226] Tang B.L., Zhang T., Low D.Y., Wong E.T., Horstmann H., Hong W. (2000). Mammalian homologues of yeast sec31p. An ubiquitously expressed form is localized to endoplasmic reticulum (ER) exit sites and is essential for ER-Golgi transport. J. Biol. Chem..

[227] De Bigault Du Granrut A., Cacas J.L. (2016). How Very-Long-Chain Fatty Acids Could Signal Stressful Conditions in Plants?. Front. Plant Sci..

[228] Lewandowska M., Keyl A., Feussner I. (2020). Wax biosynthesis in response to danger: Its regulation upon abiotic and biotic stress. New Phytol..

[229] Khan U.M., Rana I.A., Shaheen N., Raza Q., Rehman H.M., Maqbool R., Khan I.A., Atif R.M. (2023). Comparative phylogenomic insights of KCS and ELO gene families in Brassica species indicate their role in seed development and stress responsiveness. Sci. Rep..

[230] Li J., Mo X., Wang J., Chen N., Fan H., Dai C., Wu P. (2009). BREVIS RADIX is involved in cytokinin-mediated inhibition of lateral root initiation in Arabidopsis. Planta.

[231] Xu Y., Caldo K.M.P., Pal-Nath D., Ozga J., Lemieux M.J., Weselake R.J., Chen G. (2018). Properties and Biotechnological Applications of Acyl-CoA:diacylglycerol Acyltransferase and Phospholipid:diacylglycerol Acyltransferase from Terrestrial Plants and Microalgae. Lipids.

[232] Sah S.K., Fan J., Blanford J., Shanklin J., Xu C. (2024). Physiological Functions of Phospholipid:Diacylglycerol Acyltransferases. Plant Cell Physiol..

[233] Zienkiewicz K., Du Z.Y., Ma W., Vollheyde K., Benning C. (2016). Stress-induced neutral lipid biosynthesis in microalgae—Molecular, cellular and physiological insights. Biochim. Biophys. Acta.

[234] Holecek M. (2022). Serine Metabolism in Health and Disease and as a Conditionally Essential Amino Acid. Nutrients.

[235] Suzuki K., Itai R., Suzuki K., Nakanishi H., Nishizawa N.K., Yoshimura E., Mori S. (1998). Formate dehydrogenase, an enzyme of anaerobic metabolism, is induced by iron deficiency in barley roots. Plant Physiol..

[236] Igamberdiev A.U., Kleczkowski L.A. (2018). Corrigendum: The Glycerate and Phosphorylated Pathways of Serine Synthesis in Plants: The Branches of Plant Glycolysis Linking Carbon and Nitrogen Metabolism. Front. Plant Sci..

[237] Fernandez-Bernal A., Mota N., Pamplona R., Area-Gomez E., Portero-Otin M. (2025). Mission cholesterol: Uncovering its hidden role in ALS neurodegeneration. Biochim. Biophys. Acta Mol. Basis Dis..

[238] Griffiths W.J., Wang Y. (2019). Oxysterol research: A brief review. Biochem. Soc. Trans..

[239] Kim B.Y., Jin B.R. (2015). Apolipophorin III from honeybees (Apis cerana) exhibits antibacterial activity. Comp. Biochem. Physiol. B Biochem. Mol. Biol..

[240] Maravilla E., Le D.P., Tran J.J., Chiu M.H., Prenner E.J., Weers P.M.M. (2020). Apolipophorin III interaction with phosphatidylglycerol and lipopolysaccharide: A potential mechanism for antimicrobial activity. Chem. Phys. Lipids.

[241] Alencar M.B., Girard R., Crispim M., Baptista C.G., Biran M., Bringaud F., Silber A.M. (2025). The role of l-serine and l-threonine in the energy metabolism and nutritional stress response of Trypanosoma cruzi. mSphere.

[242] Wiebe M.A., Brannon J.R., Steiner B.D., Bamidele A., Schrimpe-Rutledge A.C., Codreanu S.G., Sherrod S.D., McLean J.A., Hadjifrangiskou M. (2022). Serine Deamination Is a New Acid Tolerance Mechanism Observed in Uropathogenic *Escherichia coli*. mBio.

[243] Sassa T., Kihara A. (2014). Metabolism of very long-chain Fatty acids: Genes and pathophysiology. Biomol. Ther..

[244] Kim J.G., Hudson L.D. (1992). Novel member of the zinc finger superfamily: A C_2_-HC finger that recognizes a glia-specific gene. Mol. Cell. Biol..

[245] Nielsen J.A., Berndt J.A., Hudson L.D., Armstrong R.C. (2004). Myelin transcription factor 1 (Myt1) modulates the proliferation and differentiation of oligodendrocyte lineage cells. Mol. Cell. Neurosci..

[246] Lin F., Ribar T.J., Means A.R. (2011). The Ca^2+^/calmodulin-dependent protein kinase kinase, CaMKK2, inhibits preadipocyte differentiation. Endocrinology.

[247] Kobayashi T., Imachi H., Fukunaga K., Lyu J., Sato S., Saheki T., Ibata T., Matsumoto M., Japar S.B., Murao K. (2022). HDL promotes adiponectin gene expression via the CAMKK/CAMKIV pathway. J. Mol. Endocrinol..

[248] Lin C., Pulliam T.L., Han J.J., Xu J., Recio C.V., Wilkenfeld S.R., Shi Y., Kushwaha M., Bench S., Ruiz E. (2025). Cholesterol metabolism regulated by CAMKK2-CREB signaling promotes castration-resistant prostate cancer. Cell Rep..

[249] Zheng M., Wang Y.H., Wu X.N., Wu S.Q., Lu B.J., Dong M.Q., Zhang H., Sun P., Lin S.C., Guan K.L. (2011). Inactivation of Rheb by PRAK-mediated phosphorylation is essential for energy-depletion-induced suppression of mTORC1. Nat. Cell Biol..

[250] Kostenko S., Dumitriu G., Laegreid K.J., Moens U. (2011). Physiological roles of mitogen-activated-protein-kinase-activated p38-regulated/activated protein kinase. World J. Biol. Chem..

[251] Tang J., Liu J., Li X., Zhong Y., Zhong T., Liu Y., Wang J.H., Jiang Y. (2014). PRAK interacts with DJ-1 and prevents oxidative stress-induced cell death. Oxid. Med. Cell. Longev..

[252] Dancik G.M., Varisli L., Vlahopoulos S.A. (2023). The Molecular Context of Oxidant Stress Response in Cancer Establishes ALDH1A1 as a Critical Target: What This Means for Acute Myeloid Leukemia. Int. J. Mol. Sci..

[253] Hellgren M., Stromberg P., Gallego O., Martras S., Farres J., Persson B., Pares X., Hoog J.O. (2007). Alcohol dehydrogenase 2 is a major hepatic enzyme for human retinol metabolism. Cell Mol. Life Sci..

[254] Godi A., Di Campli A., Konstantakopoulos A., Di Tullio G., Alessi D.R., Kular G.S., Daniele T., Marra P., Lucocq J.M., De Matteis M.A. (2004). FAPPs control Golgi-to-cell-surface membrane traffic by binding to ARF and PtdIns(4)P. Nat. Cell Biol..

[255] Masgrau A., Battola A., Sanmartin T., Pryszcz L.P., Gabaldon T., Mendoza M. (2017). Distinct roles of the polarity factors Boi1 and Boi2 in the control of exocytosis and abscission in budding yeast. Mol. Biol. Cell.

[256] Hardie D.G., Carling D., Carlson M. (1998). The AMP-activated/SNF1 protein kinase subfamily: Metabolic sensors of the eukaryotic cell?. Annu. Rev. Biochem..

[257] Sanchez-Pulido L., Ponting C.P. (2020). Hexa-Longin domain scaffolds for inter-Rab signalling. Bioinformatics.

[258] Chafe S.C., Mangroo D. (2010). Scyl1 facilitates nuclear tRNA export in mammalian cells by acting at the nuclear pore complex. Mol. Biol. Cell.

[259] Pelletier S. (2016). SCYL pseudokinases in neuronal function and survival. Neural Regen. Res..

[260] Schmidt W.M., Kraus C., Hoger H., Hochmeister S., Oberndorfer F., Branka M., Bingemann S., Lassmann H., Muller M., Macedo-Souza L.I. (2007). Mutation in the Scyl1 gene encoding amino-terminal kinase-like protein causes a recessive form of spinocerebellar neurodegeneration. EMBO Rep..

[261] Burman J.L., Bourbonniere L., Philie J., Stroh T., Dejgaard S.Y., Presley J.F., McPherson P.S. (2008). Scyl1, mutated in a recessive form of spinocerebellar neurodegeneration, regulates COPI-mediated retrograde traffic. J. Biol. Chem..

[262] Pelletier S., Gingras S., Howell S., Vogel P., Ihle J.N. (2012). An early onset progressive motor neuron disorder in Scyl1-deficient mice is associated with mislocalization of TDP-43. J. Neurosci..

[263] Schmidt W.M., Rutledge S.L., Schule R., Mayerhofer B., Zuchner S., Boltshauser E., Bittner R.E. (2015). Disruptive SCYL1 Mutations Underlie a Syndrome Characterized by Recurrent Episodes of Liver Failure, Peripheral Neuropathy, Cerebellar Atrophy, and Ataxia. Am. J. Hum. Genet..

[264] Kuliyev E., Gingras S., Guy C.S., Howell S., Vogel P., Pelletier S. (2018). Overlapping Role of SCYL1 and SCYL3 in Maintaining Motor Neuron Viability. J. Neurosci..

[265] Amano G., Matsuzaki S., Mori Y., Miyoshi K., Han S., Shikada S., Takamura H., Yoshimura T., Katayama T. (2020). SCYL1 arginine methylation by PRMT1 is essential for neurite outgrowth via Golgi morphogenesis. Mol. Biol. Cell.

[266] Kaeser-Pebernard S., Vionnet C., Mari M., Sankar D.S., Hu Z., Roubaty C., Martinez-Martinez E., Zhao H., Spuch-Calvar M., Petri-Fink A. (2022). mTORC1 controls Golgi architecture and vesicle secretion by phosphorylation of SCYL1. Nat. Commun..

[267] Alfaro G., Johansen J., Dighe S.A., Duamel G., Kozminski K.G., Beh C.T. (2011). The sterol-binding protein Kes1/Osh4p is a regulator of polarized exocytosis. Traffic.

[268] Aravind L., Koonin E.V. (1998). Phosphoesterase domains associated with DNA polymerases of diverse origins. Nucleic Acids Res..

[269] Gakh O., Smith D.Y.t., Isaya G. (2008). Assembly of the iron-binding protein frataxin in Saccharomyces cerevisiae responds to dynamic changes in mitochondrial iron influx and stress level. J. Biol. Chem..

[270] Gervason S., Larkem D., Mansour A.B., Botzanowski T., Muller C.S., Pecqueur L., Le Pavec G., Delaunay-Moisan A., Brun O., Agramunt J. (2019). Physiologically relevant reconstitution of iron-sulfur cluster biosynthesis uncovers persulfide-processing functions of ferredoxin-2 and frataxin. Nat. Commun..

[271] Lill R., Freibert S.A. (2020). Mechanisms of Mitochondrial Iron-Sulfur Protein Biogenesis. Annu. Rev. Biochem..

[272] Moss D.K., Wilde A., Lane J.D. (2009). Dynamic release of nuclear RanGTP triggers TPX2-dependent microtubule assembly during the apoptotic execution phase. J. Cell Sci..

[273] Hadar S., Meller A., Saida N., Shalgi R. (2022). Stress-induced transcriptional readthrough into neighboring genes is linked to intron retention. iScience.

[274] Vilborg A., Sabath N., Wiesel Y., Nathans J., Levy-Adam F., Yario T.A., Steitz J.A., Shalgi R. (2017). Comparative analysis reveals genomic features of stress-induced transcriptional readthrough. Proc. Natl. Acad. Sci. USA.

[275] Vilborg A., Steitz J.A. (2017). Readthrough transcription: How are DoGs made and what do they do?. RNA Biol..

[276] Caldas P., Luz M., Baseggio S., Andrade R., Sobral D., Grosso A.R. (2024). Transcription readthrough is prevalent in healthy human tissues and associated with inherent genomic features. Commun. Biol..

[277] Mei Y., Cheng Z., Lu Y., Wu S., Chen X. (2025). Comprehensive resource for transcription readthrough events in healthy human tissues. Sci. Data.

[278] Yu F., Sabeti P.C., Hardenbol P., Fu Q., Fry B., Lu X., Ghose S., Vega R., Perez A., Pasternak S. (2005). Positive selection of a pre-expansion CAG repeat of the human SCA2 gene. PLoS Genet..

[279] Chitale G.G., Kulkarni S.R., Bapat S.A. (2025). Chimerism: A whole new perspective in gene regulation. Biochim. Biophys. Acta Gen. Subj..

[280] Shine M., Gordon J., Scharfen L., Zigackova D., Herzel L., Neugebauer K.M. (2024). Co-transcriptional gene regulation in eukaryotes and prokaryotes. Nat. Rev. Mol. Cell Biol..

[281] Ciosk R., DePalma M., Priess J.R. (2004). ATX-2, the *C. elegans* ortholog of ataxin 2, functions in translational regulation in the germline. Development.

[282] Lim C., Allada R. (2013). ATAXIN-2 activates PERIOD translation to sustain circadian rhythms in Drosophila. Science.

[283] Zhang Y., Ling J., Yuan C., Dubruille R., Emery P. (2013). A role for Drosophila ATX2 in activation of PER translation and circadian behavior. Science.

[284] Sudhakaran I.P., Hillebrand J., Dervan A., Das S., Holohan E.E., Hulsmeier J., Sarov M., Parker R., VijayRaghavan K., Ramaswami M. (2014). FMRP and Ataxin-2 function together in long-term olfactory habituation and neuronal translational control. Proc. Natl. Acad. Sci. USA.

[285] Fittschen M., Lastres-Becker I., Halbach M.V., Damrath E., Gispert S., Azizov M., Walter M., Muller S., Auburger G. (2015). Genetic ablation of ataxin-2 increases several global translation factors in their transcript abundance but decreases translation rate. Neurogenetics.

[286] Dansithong W., Paul S., Figueroa K.P., Rinehart M.D., Wiest S., Pflieger L.T., Scoles D.R., Pulst S.M. (2015). Ataxin-2 regulates RGS8 translation in a new BAC-SCA2 transgenic mouse model. PLoS Genet..

[287] Lastres-Becker I., Nonis D., Eich F., Klinkenberg M., Gorospe M., Kotter P., Klein F.A., Kedersha N., Auburger G. (2016). Mammalian ataxin-2 modulates translation control at the pre-initiation complex via PI3K/mTOR and is induced by starvation. Biochim. Biophys. Acta.

[288] Lee J., Yoo E., Lee H., Park K., Hur J.H., Lim C. (2017). LSM12 and ME31B/DDX6 Define Distinct Modes of Posttranscriptional Regulation by ATAXIN-2 Protein Complex in Drosophila Circadian Pacemaker Neurons. Mol. Cell.

[289] Hansen M., Zeddies S., Meinders M., di Summa F., van Alphen F.P.J., Hoogendijk A.J., Moore K.S., Halbach M., Gutierrez L., van den Biggelaar M. (2020). The RNA-Binding Protein ATXN2 is Expressed during Megakaryopoiesis and May Control Timing of Gene Expression. Int. J. Mol. Sci..

[290] Scoles D.R., Dansithong W., Pflieger L.T., Paul S., Gandelman M., Figueroa K.P., Rigo F., Bennett C.F., Pulst S.M. (2020). ALS-associated genes in SCA2 mouse spinal cord transcriptomes. Hum. Mol. Genet..

[291] Inagaki H., Hosoda N., Tsuiji H., Hoshino S.I. (2020). Direct evidence that Ataxin-2 is a translational activator mediating cytoplasmic polyadenylation. J. Biol. Chem..

[292] Rounds J.C., Corgiat E.B., Ye C., Behnke J.A., Kelly S.M., Corbett A.H., Moberg K.H. (2022). The disease-associated proteins Drosophila Nab2 and Ataxin-2 interact with shared RNAs and coregulate neuronal morphology. Genetics.

[293] Vieira de Sa R., Sudria-Lopez E., Canizares Luna M., Harschnitz O., van den Heuvel D.M.A., Kling S., Vonk D., Westeneng H.J., Karst H., Bloemenkamp L. (2024). ATAXIN-2 intermediate-length polyglutamine expansions elicit ALS-associated metabolic and immune phenotypes. Nat. Commun..

[294] Santos C.C., Schweizer N., Cairrao F., Ramirez J., Osinalde N., Yang M., Gaspar C.J., Rasheva V.I., Trigo M.L., Hensel Z. (2025). Fbxo42 promotes the degradation of Ataxin-2 granules to trigger terminal Xbp1 signaling. Nat. Commun..

[295] DeMille D., Badal B.D., Evans J.B., Mathis A.D., Anderson J.F., Grose J.H. (2015). PAS kinase is activated by direct SNF1-dependent phosphorylation and mediates inhibition of TORC1 through the phosphorylation and activation of Pbp1. Mol. Biol. Cell.

[296] Bar D.Z., Charar C., Dorfman J., Yadid T., Tafforeau L., Lafontaine D.L., Gruenbaum Y. (2016). Cell size and fat content of dietary-restricted Caenorhabditis elegans are regulated by ATX-2, an mTOR repressor. Proc. Natl. Acad. Sci. USA.

[297] Bar D.Z., Charar C., Gruenbaum Y. (2018). Small GTPases in *C. elegans* metabolism. Small GTPases.

[298] Perlegos A.E., Durkin J., Belfer S.J., Rodriguez A., Shcherbakova O., Park K., Luong J., Bonini N.M., Kayser M.S. (2024). TDP-43 impairs sleep in Drosophila through Ataxin-2-dependent metabolic disturbance. Sci. Adv..

[299] Meierhofer D., Halbach M., Sen N.E., Gispert S., Auburger G. (2016). Ataxin-2 (Atxn2)-Knock-Out Mice Show Branched Chain Amino Acids and Fatty Acids Pathway Alterations. Mol. Cell. Proteom..

[300] Arsovic A., Halbach M.V., Canet-Pons J., Esen-Sehir D., Doring C., Freudenberg F., Czechowska N., Seidel K., Baader S.L., Gispert S. (2020). Mouse Ataxin-2 Expansion Downregulates CamKII and Other Calcium Signaling Factors, Impairing Granule-Purkinje Neuron Synaptic Strength. Int. J. Mol. Sci..

[301] Bezprozvanny I. (2011). Role of inositol 1,4,5-trisphosphate receptors in pathogenesis of Huntington’s disease and spinocerebellar ataxias. Neurochem. Res..

[302] Sen N.E., Eugenin von Bernhardi J., Olamide Adeyemi J., Arsović A., Canet-Pons J., Miralles A.J., Key J., Bina L., Romano V., Fellenz M. (2025). ATXN2 polyglutamine expansion impairs QKI-dependent alternative splicing and oligodendrocyte maintenance. BioRXiv.

[303] Nobrega C., Mendonca L., Marcelo A., Lamaziere A., Tome S., Despres G., Matos C.A., Mechmet F., Langui D., den Dunnen W. (2019). Restoring brain cholesterol turnover improves autophagy and has therapeutic potential in mouse models of spinocerebellar ataxia. Acta Neuropathol..

[304] Armstrong J., Bonaventura I., Rojo A., Gonzalez G., Corral J., Nadal N., Volpini V., Ferrer I. (2005). Spinocerebellar ataxia type 2 (SCA2) with white matter involvement. Neurosci. Lett..

[305] Rub U., Seidel K., Ozerden I., Gierga K., Brunt E.R., Schols L., de Vos R.A., den Dunnen W., Schultz C., Auburger G. (2007). Consistent affection of the central somatosensory system in spinocerebellar ataxia type 2 and type 3 and its significance for clinical symptoms and rehabilitative therapy. Brain Res. Rev..

[306] Mercadillo R.E., Galvez V., Diaz R., Hernandez-Castillo C.R., Campos-Romo A., Boll M.C., Pasaye E.H., Fernandez-Ruiz J. (2014). Parahippocampal gray matter alterations in Spinocerebellar Ataxia Type 2 identified by voxel based morphometry. J. Neurol. Sci..

[307] Hernandez-Castillo C.R., Galvez V., Mercadillo R., Diaz R., Campos-Romo A., Fernandez-Ruiz J. (2015). Extensive White Matter Alterations and Its Correlations with Ataxia Severity in SCA 2 Patients. PLoS ONE.

[308] Stezin A., Bhardwaj S., Khokhar S., Hegde S., Jain S., Bharath R.D., Saini J., Pal P.K. (2021). In vivo microstructural white matter changes in early spinocerebellar ataxia 2. Acta Neurol. Scand..

[309] Tu Y., Li Z., Xiong F., Gao F. (2024). Progressive white matter degeneration in patients with spinocerebellar ataxia type 2. Neuroradiology.

[310] Sha R., Su S., Hu M., Ma L., Cai H., Wu C., Zhao J. (2024). Global and Regional Brain Grey and White Matter Morphometry Alterations in Type 1, 2, and 3 Spinocerebellar Ataxias (SCAs) Patients. Cerebellum.

[311] Velazquez-Perez L., Rodriguez-Labrada R., Torres-Vega R., Montero J.M., Vazquez-Mojena Y., Auburger G., Ziemann U. (2016). Central motor conduction time as prodromal biomarker in spinocerebellar ataxia type 2. Mov. Disord..

[312] Velazquez-Perez L., Rodriguez-Labrada R., Torres-Vega R., Medrano Montero J., Vazquez-Mojena Y., Auburger G., Ziemann U. (2016). Abnormal corticospinal tract function and motor cortex excitability in non-ataxic SCA2 mutation carriers: A TMS study. Clin. Neurophysiol..

[313] Diallo A., Jacobi H., Schmitz-Hubsch T., Cook A., Labrum R., Durr A., Brice A., Charles P., Marelli C., Mariotti C. (2017). Body Mass Index Decline Is Related to Spinocerebellar Ataxia Disease Progression. Mov. Disord. Clin. Pract..

[314] Rodriguez-Grana T., Rodriguez-Labrada R., Santana-Porben S., Reynaldo-Cejas L., Medrano-Montero J., Canales-Ochoa N., Silva-Ricardo Y., Torres-Vega R., Gonzalez-Zaldivar Y., Almaguer-Gotay D. (2022). Weight loss is correlated with disease severity in Spinocerebellar ataxia type 2: A cross-sectional cohort study. Nutr. Neurosci..

[315] Almaguer-Mederos L.E., Aguilera-Rodriguez R., Almaguer-Gotay D., Hechavarria-Barzaga K., Alvarez-Sosa A., Chapman-Rodriguez Y., Silva-Ricardo Y., Gonzalez-Zaldivar Y., Vazquez-Mojena Y., Cuello-Almarales D. (2020). Testosterone Levels Are Decreased and Associated with Disease Duration in Male Spinocerebellar Ataxia Type 2 Patients. Cerebellum.

[316] Yasukochi Y., Sakuma J., Takeuchi I., Kato K., Oguri M., Fujimaki T., Horibe H., Yamada Y. (2019). Identification of six novel susceptibility loci for dyslipidemia using longitudinal exome-wide association studies in a Japanese population. Genomics.

[317] Sanchez-Alvarez M., Del Pozo M.A., Bakal C. (2018). AKT-mTOR signaling modulates the dynamics of IRE1 RNAse activity by regulating ER-mitochondria contacts. Sci. Rep..

[318] Huang S., Xing Y., Liu Y. (2019). Emerging roles for the ER stress sensor IRE1alpha in metabolic regulation and disease. J. Biol. Chem..

[319] Lachance V., Belanger S.M., Hay C., Le Corvec V., Banouvong V., Lapalme M., Tarmoun K., Beaucaire G., Lussier M.P., Kourrich S. (2023). Overview of Sigma-1R Subcellular Specific Biological Functions and Role in Neuroprotection. Int. J. Mol. Sci..

[320] Zhao Q., Wang J., Levichkin I.V., Stasinopoulos S., Ryan M.T., Hoogenraad N.J. (2002). A mitochondrial specific stress response in mammalian cells. EMBO J..

[321] Haynes C.M., Petrova K., Benedetti C., Yang Y., Ron D. (2007). ClpP mediates activation of a mitochondrial unfolded protein response in *C. elegans*. Dev. Cell.

[322] Gispert S., Parganlija D., Klinkenberg M., Drose S., Wittig I., Mittelbronn M., Grzmil P., Koob S., Hamann A., Walter M. (2013). Loss of mitochondrial peptidase Clpp leads to infertility, hearing loss plus growth retardation via accumulation of CLPX, mtDNA and inflammatory factors. Hum. Mol. Genet..

[323] Quiros P.M., Prado M.A., Zamboni N., D’Amico D., Williams R.W., Finley D., Gygi S.P., Auwerx J. (2017). Multi-omics analysis identifies ATF4 as a key regulator of the mitochondrial stress response in mammals. J. Cell Biol..

[324] Bhaskaran S., Pharaoh G., Ranjit R., Murphy A., Matsuzaki S., Nair B.C., Forbes B., Gispert S., Auburger G., Humphries K.M. (2018). Loss of mitochondrial protease ClpP protects mice from diet-induced obesity and insulin resistance. EMBO Rep..

[325] Maletzko A., Key J., Wittig I., Gispert S., Koepf G., Canet-Pons J., Torres-Odio S., West A.P., Auburger G. (2021). Increased presence of nuclear DNAJA3 and upregulation of cytosolic STAT1 and of nucleic acid sensors trigger innate immunity in the ClpP-null mouse. Neurogenetics.

[326] Liu Z., Qiang Y., Shan S., Wang S., Liu Z., Yang Y., Huang Z., Song M., Zhao X., Song F. (2024). Aberrant mitochondrial aggregation of TDP-43 activated mitochondrial unfolded protein response and contributed to recovery of acetaminophen induced acute liver injury. Toxicol. Res..

[327] Auburger G., Key J., Gispert S. (2022). The Bacterial ClpXP-ClpB Family Is Enriched with RNA-Binding Protein Complexes. Cells.

[328] Key J., Gispert S., Auburger G. (2024). Knockout Mouse Studies Show That Mitochondrial CLPP Peptidase and CLPX Unfoldase Act in Matrix Condensates near IMM, as Fast Stress Response in Protein Assemblies for Transcript Processing, Translation, and Heme Production. Genes.

[329] Batsale M., Bahammou D., Fouillen L., Mongrand S., Joubes J., Domergue F. (2021). Biosynthesis and Functions of Very-Long-Chain Fatty Acids in the Responses of Plants to Abiotic and Biotic Stresses. Cells.

[330] Barnes-Velez J.A., Aksoy Yasar F.B., Hu J. (2023). Myelin lipid metabolism and its role in myelination and myelin maintenance. Innovation.

[331] Sen N.E., Gispert S., Auburger G. (2017). PINK1 and Ataxin-2 as modifiers of growth. Oncotarget.

[332] Liu L., Liu C., Zhong Y., Apostolou A., Fang S. (2012). ER stress response during the differentiation of H9 cells induced by retinoic acid. Biochem. Biophys. Res. Commun..

[333] Li J., Cai X., Xia Q., Yao K., Chen J., Zhang Y., Naranmandura H., Liu X., Wu Y. (2015). Involvement of endoplasmic reticulum stress in all-trans-retinal-induced retinal pigment epithelium degeneration. Toxicol. Sci..

[334] Liu Y., Chen Y., Zhang J., Liu Y., Zhang Y., Su Z. (2017). Retinoic acid receptor-related orphan receptor alpha stimulates adipose tissue inflammation by modulating endoplasmic reticulum stress. J. Biol. Chem..

[335] Chen Y., Pang J., Ye L., Zhang Z., Kang J., Qiu Z., Lin N., Liu H. (2024). Pin1 Downregulation Is Involved in Excess Retinoic Acid-Induced Failure of Neural Tube Closure. Int. J. Mol. Sci..

[336] Pu J., Liu H., Li X., Chen D., Tian G., He J., Zheng P., Yan H., Wu A., Mao X. (2025). All-trans retinoic acid protects piglets from TGEV-induced diarrhea and intestinal epithelial apoptosis by modulating redox status and endoplasmic reticulum stress pathways. J. Anim. Sci..

[337] Yoshida H., Matsui T., Yamamoto A., Okada T., Mori K. (2001). XBP1 mRNA is induced by ATF6 and spliced by IRE1 in response to ER stress to produce a highly active transcription factor. Cell.

[338] Ali M.M., Bagratuni T., Davenport E.L., Nowak P.R., Silva-Santisteban M.C., Hardcastle A., McAndrews C., Rowlands M.G., Morgan G.J., Aherne W. (2011). Structure of the Ire1 autophosphorylation complex and implications for the unfolded protein response. EMBO J..

[339] Eletto D., Eletto D., Dersh D., Gidalevitz T., Argon Y. (2014). Protein disulfide isomerase A6 controls the decay of IRE1alpha signaling via disulfide-dependent association. Mol. Cell.

[340] Lindholm D., Wootz H., Korhonen L. (2006). ER stress and neurodegenerative diseases. Cell Death Differ..

[341] Yoshida H., Nadanaka S., Sato R., Mori K. (2006). XBP1 is critical to protect cells from endoplasmic reticulum stress: Evidence from Site-2 protease-deficient Chinese hamster ovary cells. Cell Struct. Funct..

[342] de Mena L., Lopez-Scarim J., Rincon-Limas D.E. (2021). TDP-43 and ER Stress in Neurodegeneration: Friends or Foes?. Front. Mol. Neurosci..

[343] Wang C., Chang Y., Zhu J., Ma R., Li G. (2022). Dual Role of Inositol-requiring Enzyme 1alpha-X-box Binding protein 1 Signaling in Neurodegenerative Diseases. Neuroscience.

[344] Matus S., Nassif M., Glimcher L.H., Hetz C. (2009). XBP-1 deficiency in the nervous system reveals a homeostatic switch to activate autophagy. Autophagy.

[345] Wu R., Zhang Q.H., Lu Y.J., Ren K., Yi G.H. (2015). Involvement of the IRE1alpha-XBP1 pathway and XBP1s-dependent transcriptional reprogramming in metabolic diseases. DNA Cell Biol..

[346] Luo X., Alfason L., Wei M., Wu S., Kasim V. (2022). Spliced or Unspliced, That Is the Question: The Biological Roles of XBP1 Isoforms in Pathophysiology. Int. J. Mol. Sci..

[347] Wen X.Y., Stewart A.K., Sooknanan R.R., Henderson G., Hawley T.S., Reimold A.M., Glimcher L.H., Baumann H., Malek L.T., Hawley R.G. (1999). Identification of c-myc promoter-binding protein and X-box binding protein 1 as interleukin-6 target genes in human multiple myeloma cells. Int. J. Oncol..

[348] Zhou X., Jiang H., Hou J. (2007). Coordination of upregulated XBP-1 and downregulated c-myc during myeloma cell differentiation induced by 2-methoxyestradiol. Leuk. Res..

[349] Xie H., Tang C.H., Song J.H., Mancuso A., Del Valle J.R., Cao J., Xiang Y., Dang C.V., Lan R., Sanchez D.J. (2018). IRE1alpha RNase-dependent lipid homeostasis promotes survival in Myc-transformed cancers. J. Clin. Investig..

[350] Zhao N., Cao J., Xu L., Tang Q., Dobrolecki L.E., Lv X., Talukdar M., Lu Y., Wang X., Hu D.Z. (2018). Pharmacological targeting of MYC-regulated IRE1/XBP1 pathway suppresses MYC-driven breast cancer. J. Clin. Investig..

[351] Dong H., Adams N.M., Xu Y., Cao J., Allan D.S.J., Carlyle J.R., Chen X., Sun J.C., Glimcher L.H. (2019). The IRE1 endoplasmic reticulum stress sensor activates natural killer cell immunity in part by regulating c-Myc. Nat. Immunol..

[352] Abdullah A., Talwar P., d’Hellencourt C.L., Ravanan P. (2019). IRE1alpha is critical for Kaempferol-induced neuroblastoma differentiation. FEBS J..

[353] Wiedemeyer R., Westermann F., Wittke I., Nowock J., Schwab M. (2003). Ataxin-2 promotes apoptosis of human neuroblastoma cells. Oncogene.

[354] Junjappa R.P., Patil P., Bhattarai K.R., Kim H.R., Chae H.J. (2018). IRE1alpha Implications in Endoplasmic Reticulum Stress-Mediated Development and Pathogenesis of Autoimmune Diseases. Front. Immunol..

[355] Halbach M.V., Gispert S., Stehning T., Damrath E., Walter M., Auburger G. (2017). Atxn2 Knockout and CAG42-Knock-in Cerebellum Shows Similarly Dysregulated Expression in Calcium Homeostasis Pathway. Cerebellum.

[356] Tada M., Nishizawa M., Onodera O. (2016). Roles of inositol 1,4,5-trisphosphate receptors in spinocerebellar ataxias. Neurochem. Int..

[357] He L., Kim S.O., Kwon O., Jeong S.J., Kim M.S., Lee H.G., Osada H., Jung M., Ahn J.S., Kim B.Y. (2009). ATM blocks tunicamycin-induced endoplasmic reticulum stress. FEBS Lett..

[358] Hetz C., Thielen P., Matus S., Nassif M., Court F., Kiffin R., Martinez G., Cuervo A.M., Brown R.H., Glimcher L.H. (2009). XBP-1 deficiency in the nervous system protects against amyotrophic lateral sclerosis by increasing autophagy. Genes Dev..

[359] Reichlmeir M., Duecker R.P., Rohrich H., Key J., Schubert R., Abell K., Possemato A.P., Stokes M.P., Auburger G. (2024). The ataxia-telangiectasia disease protein ATM controls vesicular protein secretion via CHGA and microtubule dynamics via CRMP5. Neurobiol. Dis..

[360] Mishra P., Sivakumar A., Johnson A., Pernaci C., Warden A.S., El-Hachem L.R., Hansen E., Badell-Grau R.A., Khare V., Ramirez G. (2024). Gene editing improves endoplasmic reticulum-mitochondrial contacts and unfolded protein response in Friedreich’s ataxia iPSC-derived neurons. Front. Pharmacol..

[361] Valenzuela V., Becerra D., Astorga J.I., Fuentealba M., Diaz G., Bargsted L., Chacon C., Martinez A., Gozalvo R., Jackson K. (2025). Artificial enforcement of the unfolded protein response reduces disease features in multiple preclinical models of ALS/FTD. Mol. Ther..

[362] Shen D., Vincent A., Udine E., Buhidma Y., Anoar S., Tsintzas E., Maeland M., Xu D., Carcole M., Osumi-Sutherland D. (2025). Differential neuronal vulnerability to C9orf72 repeat expansion driven by Xbp1-induced endoplasmic reticulum-associated degradation. Cell Rep..

[363] Grima N., Smith A.N., Shepherd C.E., Henden L., Zaw T., Carroll L., Rowe D.B., Kiernan M.C., Blair I.P., Williams K.L. (2025). Multi-region brain transcriptomic analysis of amyotrophic lateral sclerosis reveals widespread RNA alterations and substantial cerebellum involvement. Mol. Neurodegener..

[364] Li Y., Liu D., Li S. (2025). IRE1/Xbp1 promotes the clearance of poly(GR) dipeptide repeats in amyotrophic lateral sclerosis. J. Biol. Chem..

[365] Matsui T. (1997). Transcriptional regulation of a Purkinje cell-specific gene through a functional interaction between ROR alpha and RAR. Genes Cells.

[366] Jetten A.M., Kurebayashi S., Ueda E. (2001). The ROR nuclear orphan receptor subfamily: Critical regulators of multiple biological processes. Prog. Nucleic Acid Res. Mol. Biol..

[367] Agudo M., Yip P., Davies M., Bradbury E., Doherty P., McMahon S., Maden M., Corcoran J.P. (2010). A retinoic acid receptor beta agonist (CD2019) overcomes inhibition of axonal outgrowth via phosphoinositide 3-kinase signalling in the injured adult spinal cord. Neurobiol. Dis..

[368] Chen C.T., Schultz J.A., Haven S.E., Wilhite B., Liu C.H., Chen J., Hibbeln J.R. (2020). Loss of RAR-related orphan receptor alpha (RORalpha) selectively lowers docosahexaenoic acid in developing cerebellum. Prostaglandins Leukot. Essent. Fat. Acids.

[369] Petkovich M., Chambon P. (2022). Retinoic acid receptors at 35 years. J. Mol. Endocrinol..

[370] Chauvet C., Bois-Joyeux B., Berra E., Pouyssegur J., Danan J.L. (2004). The gene encoding human retinoic acid-receptor-related orphan receptor alpha is a target for hypoxia-inducible factor 1. Biochem. J..

[371] Boukhtouche F., Vodjdani G., Jarvis C.I., Bakouche J., Staels B., Mallet J., Mariani J., Lemaigre-Dubreuil Y., Brugg B. (2006). Human retinoic acid receptor-related orphan receptor alpha1 overexpression protects neurones against oxidative stress-induced apoptosis. J. Neurochem..

[372] Serra H.G., Duvick L., Zu T., Carlson K., Stevens S., Jorgensen N., Lysholm A., Burright E., Zoghbi H.Y., Clark H.B. (2006). RORalpha-mediated Purkinje cell development determines disease severity in adult SCA1 mice. Cell.

[373] Fujita K., Mao Y., Uchida S., Chen X., Shiwaku H., Tamura T., Ito H., Watase K., Homma H., Tagawa K. (2017). Developmental YAPdeltaC determines adult pathology in a model of spinocerebellar ataxia type 1. Nat. Commun..

[374] Watanave M., Hoshino C., Konno A., Fukuzaki Y., Matsuzaki Y., Ishitani T., Hirai H. (2019). Pharmacological enhancement of retinoid-related orphan receptor alpha function mitigates spinocerebellar ataxia type 3 pathology. Neurobiol. Dis..

[375] Ajayi A., Yu X., Lindberg S., Langel U., Strom A.L. (2012). Expanded ataxin-7 cause toxicity by inducing ROS production from NADPH oxidase complexes in a stable inducible Spinocerebellar ataxia type 7 (SCA7) model. BMC Neurosci..

[376] Sanz A.B., Garcia R., Rodriguez-Pena J.M., Nombela C., Arroyo J. (2016). Cooperation between SAGA and SWI/SNF complexes is required for efficient transcriptional responses regulated by the yeast MAPK Slt2. Nucleic Acids Res..

[377] Napierala J.S., Rajapakshe K., Clark A., Chen Y.Y., Huang S., Mesaros C., Xu P., Blair I.A., Hauser L.A., Farmer J. (2021). Reverse Phase Protein Array Reveals Correlation of Retinoic Acid Metabolism with Cardiomyopathy in Friedreich’s Ataxia. Mol. Cell. Proteom..

[378] Fernandes N.D., Sun Y., Price B.D. (2007). Activation of the kinase activity of ATM by retinoic acid is required for CREB-dependent differentiation of neuroblastoma cells. J. Biol. Chem..

[379] Jarvis C.I., Staels B., Brugg B., Lemaigre-Dubreuil Y., Tedgui A., Mariani J. (2002). Age-related phenotypes in the staggerer mouse expand the RORalpha nuclear receptor’s role beyond the cerebellum. Mol. Cell. Endocrinol..

[380] Boukhtouche F., Mariani J., Tedgui A. (2004). The “CholesteROR” protective pathway in the vascular system. Arterioscler. Thromb. Vasc. Biol..

[381] Guissart C., Latypova X., Rollier P., Khan T.N., Stamberger H., McWalter K., Cho M.T., Kjaergaard S., Weckhuysen S., Lesca G. (2018). Dual Molecular Effects of Dominant RORA Mutations Cause Two Variants of Syndromic Intellectual Disability with Either Autism or Cerebellar Ataxia. Am. J. Hum. Genet..

[382] Talarico M., de Bellescize J., De Wachter M., Le Guillou X., Le Meur G., Egloff M., Isidor B., Cogne B., Beysen D., Rollier P. (2025). RORA-neurodevelopmental disorder: A unique triad of developmental disabilities, cerebellar anomalies, and myoclonic seizures. Genet. Med..

[383] Kaehler C., Isensee J., Nonhoff U., Terrey M., Hucho T., Lehrach H., Krobitsch S. (2012). Ataxin-2-like is a regulator of stress granules and processing bodies. PLoS ONE.

[384] Kiehl T.R., Nechiporuk A., Figueroa K.P., Keating M.T., Huynh D.P., Pulst S.M. (2006). Generation and characterization of Sca2 (ataxin-2) knockout mice. Biochem. Biophys. Res. Commun..

[385] Pfeffer M., Gispert S., Auburger G., Wicht H., Korf H.W. (2017). Impact of Ataxin-2 knock out on circadian locomotor behavior and PER immunoreaction in the SCN of mice. Chronobiol. Int..

[386] Kiehl T.R., Shibata H., Pulst S.M. (2000). The ortholog of human ataxin-2 is essential for early embryonic patterning in *C. elegans*. J. Mol. Neurosci..

[387] Gadgil A., Raczynska K.D. (2021). U7 snRNA: A tool for gene therapy. J. Gene Med..

[388] Liu J.L., Gall J.G. (2007). U bodies are cytoplasmic structures that contain uridine-rich small nuclear ribonucleoproteins and associate with P bodies. Proc. Natl. Acad. Sci. USA.

[389] Jacobs E.Y., Frey M.R., Wu W., Ingledue T.C., Gebuhr T.C., Gao L., Marzluff W.F., Matera A.G. (1999). Coiled bodies preferentially associate with U4, U11, and U12 small nuclear RNA genes in interphase HeLa cells but not with U6 and U7 genes. Mol. Biol. Cell.

[390] Gadgil A., Walczak A., Stepien A., Mechtersheimer J., Nishimura A.L., Shaw C.E., Ruepp M.D., Raczynska K.D. (2021). ALS-linked FUS mutants affect the localization of U7 snRNP and replication-dependent histone gene expression in human cells. Sci. Rep..

[391] Courel M., Clement Y., Bossevain C., Foretek D., Vidal Cruchez O., Yi Z., Benard M., Benassy M.N., Kress M., Vindry C. (2019). GC content shapes mRNA storage and decay in human cells. Elife.

