# Supplementary material for: Bioinformatic Analyses of the Ataxin-2 Family Since Algae Emphasize Its Small Isoforms, Large Chimerisms, and the Importance of Human Exon 1B as Target of Therapies to Prevent Neurodegeneration
Source: Int J Mol Sci. 2026 Feb 3;27(3):1499. doi: 10.3390/ijms27031499 (PMC12898128; doi:10.3390/ijms27031499)
Supplement: Supplementary file 1 [file ijms-27-01499-s001.zip › AuburgerSen_SupplMaterialS1-MultipleSequenceAlignment-ATXN2L-ATXN2.pdf]

Ataxin-2 family <u>LSM</u> alignment in fish species before and after gene duplication

CLUSTAL O(1.2.4) multiple sequence alignment

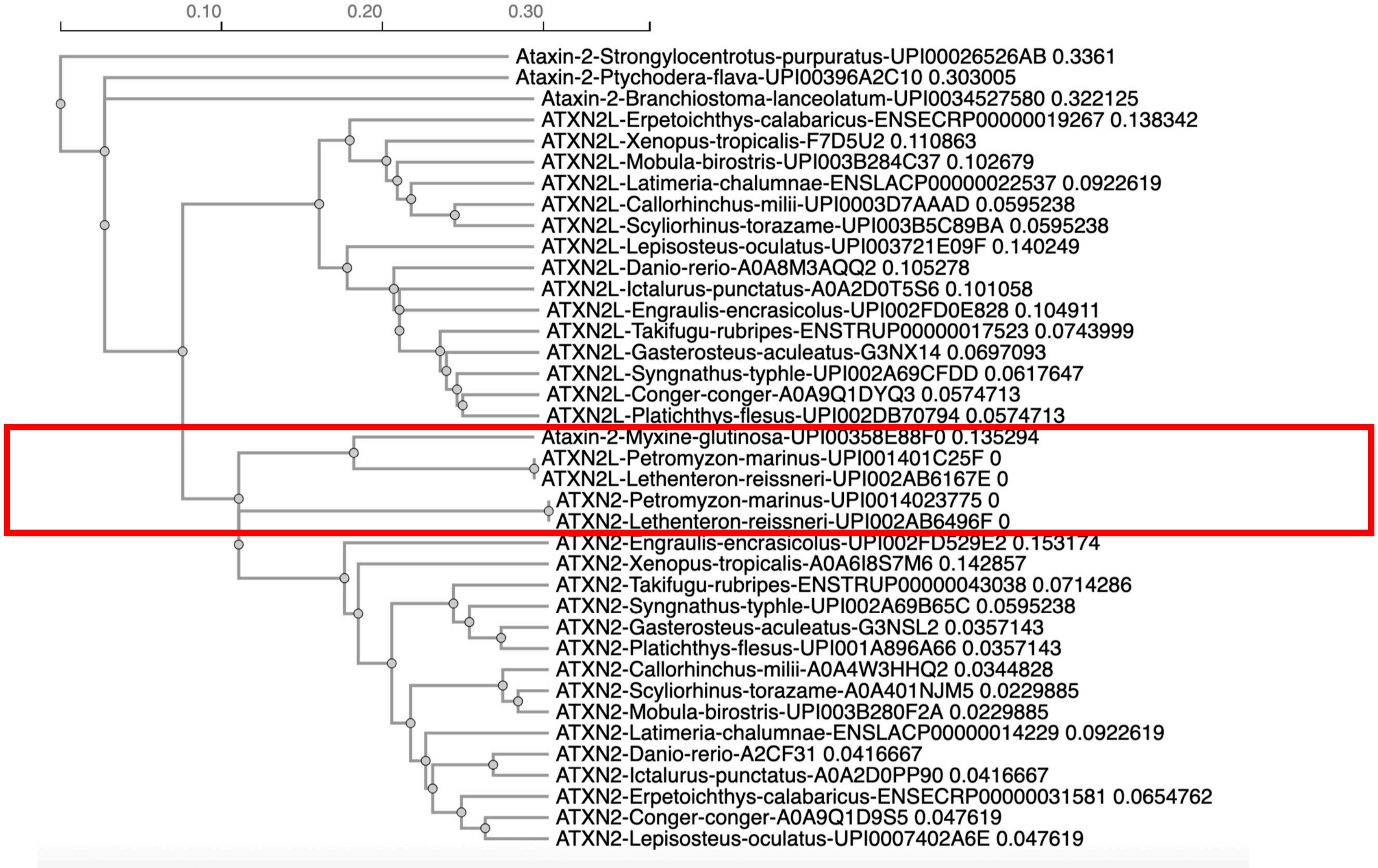

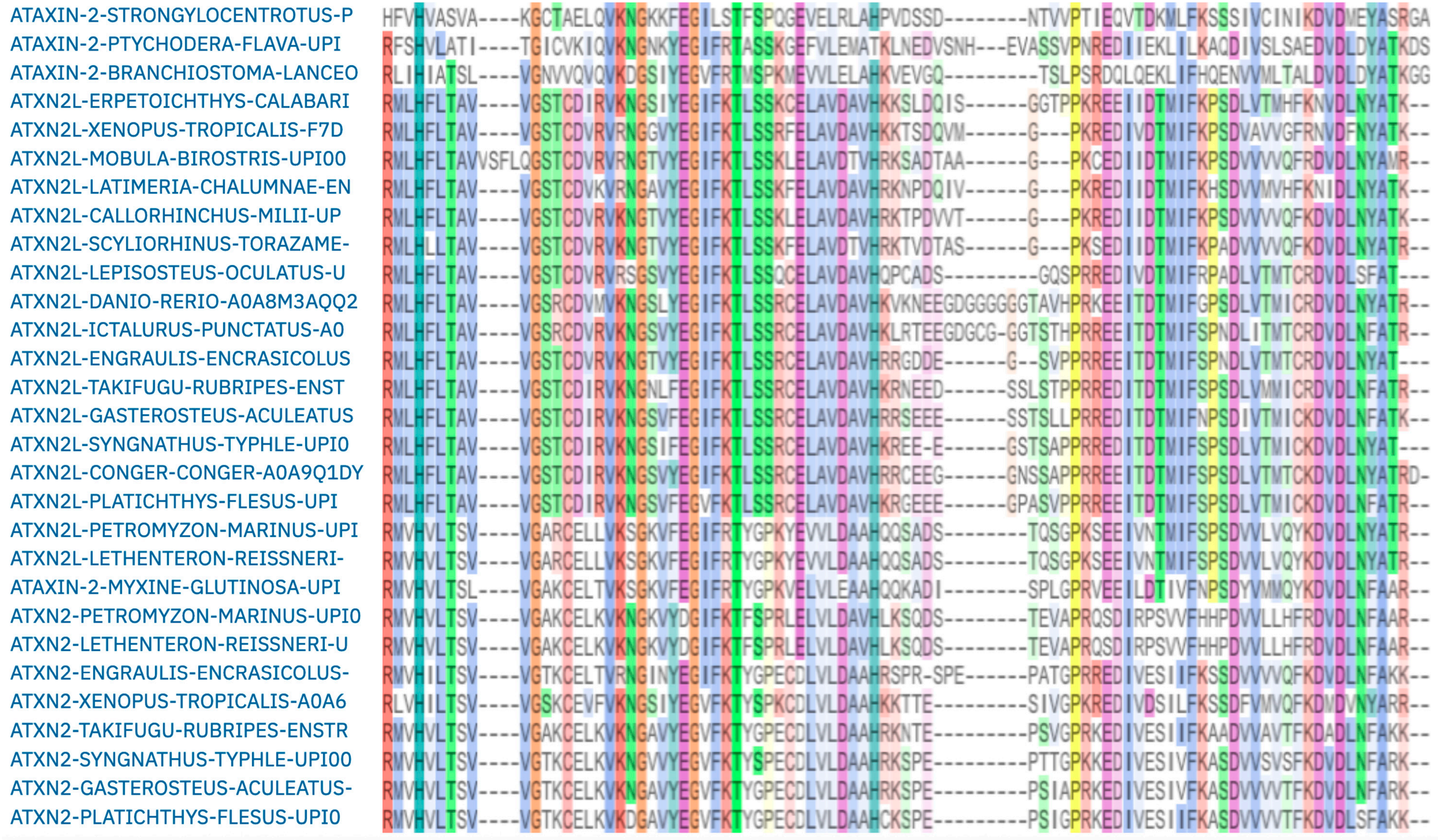
ATAXIN-2-STRONGYLOCENTROTUS-P
ATAXIN-2-PTYCHODERA-FLAVA-UPI
ATAXIN-2-BRANCHIOSTOMA-LANCEO
ATXN2L-ERPETOICHTHYS-CALABARI
ATXN2L-XENOPUS-TROPICALIS-F7D
ATXN2L-MOBULA-BIROSTRIS-UPI00
ATXN2L-LATIMERIA-CHALUMNAE-EN
ATXN2L-CALLORHINCHUS-MILII-UP
ATXN2L-SCYLIORHINUS-TORAZAME-
ATXN2L-LEPISOSTEUS-OCULATUS-U
ATXN2L-DANIO-RERIO-A0A8M3AQQ2
ATXN2L-ICTALURUS-PUNCTATUS-A0
ATXN2L-ENGRAULIS-ENCRASICOLUS
ATXN2L-TAKIFUGU-RUBRIPES-ENST
ATXN2L-GASTEROSTEUS-ACULEATUS
ATXN2L-SYNGNATHUS-TYPHLE-UPI0
ATXN2L-CONGER-CONGER-A0A9Q1DY
ATXN2L-PLATICHTHYS-FLESUS-UPI
ATXN2L-PETROMYZON-MARINUS-UPI
ATXN2L-LETHENTERON-REISSNERI-
ATAXIN-2-MYXINE-GLUTINOSA-UPI
ATXN2-PETROMYZON-MARINUS-UPI0
ATXN2-LETHENTERON-REISSNERI-U
ATXN2-ENGRAULIS-ENCRASICOLUS-
ATXN2-XENOPUS-TROPICALIS-A0A6
ATXN2-TAKIFUGU-RUBRIPES-ENSTR
ATXN2-SYNGNATHUS-TYPHLE-UPI00
ATXN2-GASTEROSTEUS-ACULEATUS-
ATXN2-PLATICHTHYS-FLESUS-UPI0

LSM sequences used:
> ATXN2L-Xenopus-tropicalis-F7D5U2
RMLHFLTAVVGSTCDVRVRNGGVYEGIFKTLSSRFELAVDAVHKKTSDQVMGPKREDIVDTMIFKPSDVAVVGFRNVDFNYATK

> ATXN2L-Danio-rerio-A0A8M3AQQ2
RMLHFLTAVVGSRCDVMVKNGSLYEGIFKTLSSRCELAVDAVHKVKNEEGDGGGGGGTAVHPRKEEITDTMIFGPSDLVTMICRDVDLNYATR

> ATXN2L-Gasterosteus-aculeatus-G3NX14
RMLHFLTAVVGSTCDIRVKNGSVFEGIFKTLSSRCELAVDAVHRRSEEESSTSLLPRREDITDTMIFNPSDIVTMICKDVDLNFATK

> ATXN2L-Takifugu-rubripes-ENSTRUP00000017523
RMLHFLTAVVGSTCDIRVKNGNLFEGIFKTLSSRCELAVDAVHKRNEEDSSLSTPPRREDITDTMIFSPSDLVMMICRDVDLNFATR

> ATXN2L-Ictalurus-punctatus-A0A2D0T5S6
RMLHFLTAVVGSRCDVRVKNGSVYEGIFKTLSSRCELAVDAVHKLRTEEGDGCGGGTSTHPRREEITDTMIFSPNDLITMTCRDVDLNFATR

> ATXN2L-Engraulis-encrasicolus-UPI002FD0E828
RMLHFLTAVVGSTCDVRVKNGTVYEGIFKTLSSRCELAVDAVHRRGDDEGSVPPRREEITDTMIFSPNDLVTMTCRDVDLNYAT

> ATXN2L-Syngnathus-typhle-UPI002A69CFDD
RMLHFLTAVVGSTCDIRVKNGSIFEGIFKTLSSRCELAVDAVHKREEEGSTSAPPRREDITDTMIFSPSDLVTMICKDVDLNYAT

> ATXN2L-Lepisosteus-oculatus-UPI003721E09F
RMLHFLTAVVGSTCDVRVRSGSVYEGIFKTLSSQCELAVDAVHQPCADSGQSPRREDIVDTMIFRPADLVTMTCRDVDLSFAT

> ATXN2L-Conger-conger-A0A9Q1DYQ3
RMLHFLTAVVGSTCDIRVKNGSVYEGIFKTLSSRCELAVDAVHRRCEEGGNSSAPPRREEITDTMIFSPSDLVTMTCKDVDLNYATRD

> ATXN2L-Erpetoichthys-calabaricus-ENSECRP00000019267
RMLHFLTAVVGSTCDIRVKNGSIYEGIFKTLSSKCELAVDAVHKKSLDQISGGTPPKREEIIDTMIFKPSDLVTMHFKNVDLNYATK

> ATXN2L-Platichthys-flesus-UPI002DB70794
RMLHFLTAVVGSTCDIRVKNGSVFEGVFKTLSSRCELAVDAVHKRGEEEGPASVPPRREEITDTMIFSPSDLVTMICKDVDLNFATR

> ATXN2L-Latimeria-chalumnae-ENSLACP00000022537
RMLHFLTAVVGSTCDVKVRNGAVYEGIFKTLSSKFELAVDAVHRKNPDQIVGPKREDIIDTMIFKHSDVVMVHFKNIDLNYATK

> ATXN2L-Callorhinchus-milii-UPI0003D7AAAD
RMLHFLTAVVGSTCDVRVKNGTVYEGIFKTLSSKLELAVDAVHRKTPDVVTGPKREDIIDTMIFKPSDVVVVQFKDVDLNYATK

> ATXN2L-Scyliorhinus-torazame-UPI003B5C89BA
RMLHLLTAVVGSTCDVRVKNGTVYEGIFKTLSSKFELAVDTVHRKTVDTASGPKSEDIIDTMIFKPADVVVVQFKDVDLNYATR

> ATXN2L-Mobula-birostris-UPI003B284C37
RMLHFLTAVVSFLQGSTCDVRVRNGTVYEGIFKTLSSKLELAVDTVHRKSADTAAGPKCEDIIDTMIFKPSDVVVVQFRDVDLNYAMR

> ATXN2L-Petromyzon-marinus-UPI001401C25F
RMVHVLTSVVGARCELLVKSGKVFEGIFRTYGPKYEVVLDAAHQQSADSTQSGPKSEEIVNTMIFSPSDVVLVQYKDVDLNYATR

> ATXN2L-Lethenteron-reissneri-UPI002AB6167E
RMVHVLTSVVGARCELLVKSGKVFEGIFRTYGPKYEVVLDAAHQQSADSTQSGPKSEEIVNTMIFSPSDVVLVQYKDVDLNYATR

> ATXN2-Xenopus-tropicalis-A0A6I8S7M6
RLVHILTSVVGSKCEVFVKNGSIYEGVFKTYSPKCDLVLDAAHKKTTESIVGPKREDIVDSILFKSSDFVMVQFKDMDVNYARR

> ATXN2-Danio-rerio-A2CF31
RMVHVLTSVVGTKCELKVKNGLIYEGVFKTYGPECDIVLDAAHRKSVEPNAGPRREDIVESIIFKSSDVVVVHFKDVDLNYAKK

> ATXN2-Gasterosteus-aculeatus-G3NSL2
RMVHVLTSVVGTKCELKVKNGAVYEGVFKTYGPECDLVLDAAHRKSPEPSIAPRKEDIVESIVFKASDVVVVTFKDVDLNFARK

> ATXN2-Takifugu-rubripes-ENSTRUP00000043038
RMVHVLTSVVGAKCELKVKNGAVYEGVFKTYGPECDLVLDAAHRKNTEPSVGPRKEDIVESIIFKAADVVAVTFKDADLNFAKK

> ATXN2-Ictalurus-punctatus-A0A2D0PP90
RMVHVLTSVVGAKCELKVKNGAIYEGVFKTYGPECDLVLDAAHRKSPEPNVGPRREDIVESIIFKASDVVVVHFKDVDLSYAKK

> ATXN2-Engraulis-encrasicolus-UPI002FD529E2
RMVHILTSVVGTKCELTVRNGINYEGIFKTYGPECDLVLDAAHRSPRSPEPATGPRREDIVESIIFKSSDVVVVQFKDVDLNFAKK

> ATXN2-Syngnathus-typhle-UPI002A69B65C
RMVHVLTSVVGTKCELKVKNGVVYEGVFKTYSPECDLVLDAAHRKSPEPTTGPKKEDIVESIVFKASDVVSVSFKDVDLNFARK

> ATXN2-Conger-conger-A0A9Q1D9S5
RMVHVLTSVVGTKCELKVKNGVVYEGVFKTYGPECDLVLDAAHRKSSDPSLGPRREDIVESIIFKASDVVVVQFKDVDLNYARK

> ATXN2-Erpetoichthys-calabaricus-ENSECRP00000031581
RMVHVLTSVVGTKCELKVKNGIVYEGVFKTYSPKCDLVLDAVHRKCLDSALGPKREDIVESIIFKASDVVVVQFKDVDLNYARK

> ATXN2-Platichthys-flesus-UPI001A896A66
RMVHVLTSVVGTKCELKVKDGAVYEGVFKTYGPECDLVLDAAHCKSPEPSIGPRKEDIVESIIFKASDVVVVTFKDVDLSFAKK

> ATXN2-Latimeria-chalumnae-ENSLACP00000014229
RMVHILTSVVGSKCELQVKNGGIYEGVFKTYSPKCDLVLDAAHRKNAESNPGPKREDIVESIIFKSSDVVLVQFKDVDLNYARK

> ATXN2-Lepisosteus-oculatus-UPI0007402A6E
RMVHVLTSVVGTKCELKVKNGVVFEGVFKTYGPECHLVLDAAHRKSPDNSLGPKREDVVDSMIFKASDVVVVQFKDVDLNYARK

> ATXN2-Callorhinchus-milii-A0A4W3HHQ2
RMVHILTSVVGAKCELKVRNGGIYEGVFKTYSPKCDLVLDAAHRKNTEFSSRDPGPKREDIVESIIFKSADVVMLHFKDVDLNYARR

> ATXN2-Scyliorhinus-torazame-A0A401NJM5
RMVHILTSVVGAKCELKVKNGGIYEGVFKTYSPKCDLVLDAAHRKNTESSPRELGPKREDIVESIIFKSADVVMLHFKDVDLSYARR

> ATXN2-Mobula-birostris-UPI003B280F2A
RMVHILTSVVGAKCELKVKNGGMYEGVFKTYSPKCDLVLDAAHRKNSESSSRELGPKREDIVESIIFKSADVVMLHFKDVDLNYARR

> ATXN2-Petromyzon-marinus-UPI0014023775
RMVHVLTSVVGAKCELKVKNGKVYDGIFKTFSPRLELVLDAVHLKSQDSTEVAPRQSDIRPSVVFHHPDVVLLHFRDVDLNFAAR

> ATXN2-Lethenteron-reissneri-UPI002AB6496F
RMVHVLTSVVGAKCELKVKNGKVYDGIFKTFSPRLELVLDAVHLKSQDSTEVAPRQSDIRPSVVFHHPDVVLLHFRDVDLNFAAR

> Ataxin-2-Branchiostoma-lanceolatum-UPI0034527580
RLIHIATSLVGNVVQVQVKDGSIYEGVFRTMSPKMEVVLELAHKVEVGQTSLPSRDQLQEKLIFHQENVVMLTALDVDLDYATKGG

> Ataxin-2-Ptychodera-flava-UPI00396A2C10
RFSHVLATITGICVKIQVKNGNKYEGIFRTASSKGEFVLEMATKLNEDVSNHEVASSVPNREDIIEKLILKAQDIVSLSAEDVDLDYATKDS

> Ataxin-2-Strongylocentrotus-purpuratus-UPI00026526AB
HFVHVASVAKGCTAELQVKNGKKFEGILSTFSPQGEVELRLAHPVDSSDNTVVPTIEQVTDKMLFKSSSIVCINIKDVDMEYASRGA

> Ataxin-2-Myxine-glutinosa-UPI00358E88F0
RMVHVLTSLVGAKCELTVKSGKVFEGIFRTYGPKVELVLEAAHQQKADISPLGPRVEEILDTIVFNPSDYVMMQYKDVDLNFAAR

Ataxin-2 family LSMAD alignment in fish species before and after gene duplication

CLUSTAL O(1.2.4) multiple sequence alignment

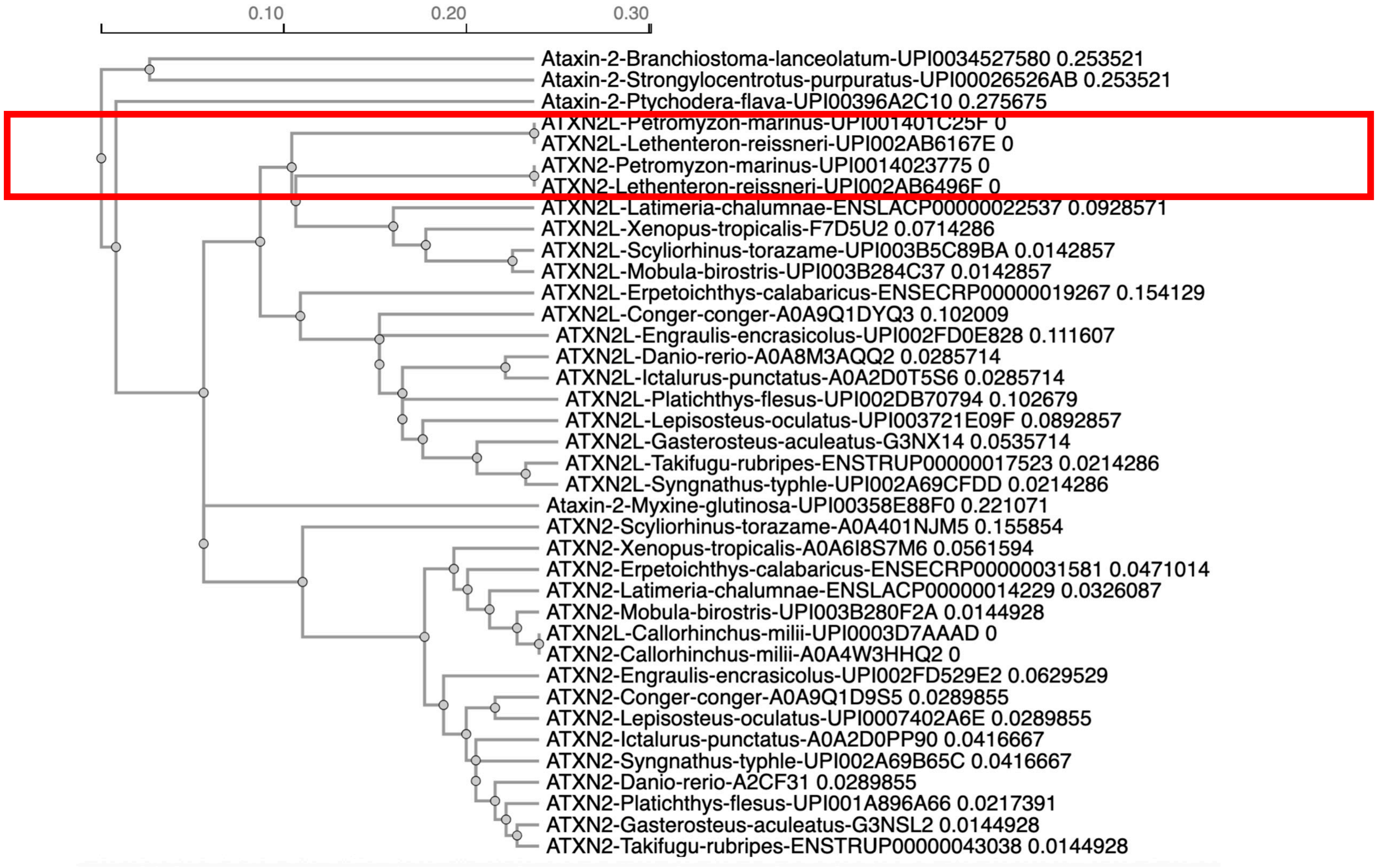

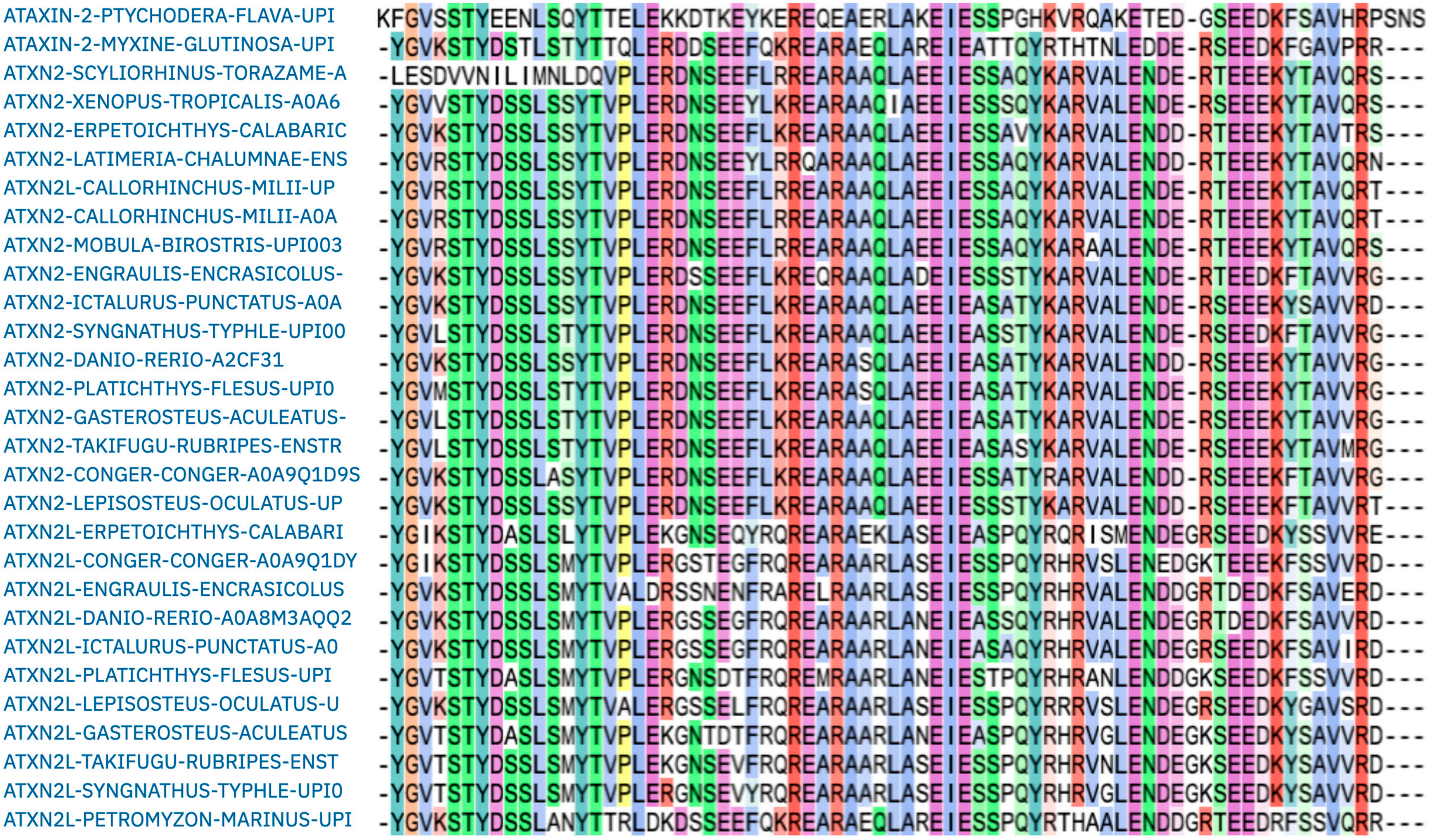
ATAXIN-2-PTYCHODERA-FLAVA-UPI KFGVSSTYEENLSQYTTELEKKDTKEYKEREQEAERLAKEIESSPGHKVRQAKETED-GSEEDKFSAVHRPSNS
ATAXIN-2-MYXINE-GLUTINOSA-UPI -YGVKSTYDSTLSTYTTQLERDDSEEFQKREARAEQLAREIEATTQYRTHTNLEDDE-RSEEDKFGAVPRR---
ATXN2-SCYLIORHINUS-TORAZAME-A -LESDVVNILIMNLDQVPLERDNSEEFLRREARAAQLAEEIESSAQYKARVALENDE-RTEEEKYTAVQRS---
ATXN2-XENOPUS-TROPICALIS-A0A6 -YGVVSTYDSSLSSYTVPLERDNSEEYLKREARAAQIAEEIESSSQYKARVALENDE-RSEEEKYTAVQRS---
ATXN2-ERPETOICHTHYS-CALABARIC -YGVKSTYDSSLSSYTVPLERDNSEEFLKREARAAQLAEEIESSAVYKARVALENDD-RTEEEKYTAVTRS---
ATXN2-LATIMERIA-CHALUMNAE-ENS -YGVRSTYDSSLSSYTVPLERDNSEEYLRRQARAAQLAEEIESSAQYKARVALENDD-RTEEEKYTAVQRN---
ATXN2L-CALLORHINCHUS-MILII-UP -YGVRSTYDSSLSSYTVPLERDNSEEFLRREARAAQLAEEIESSAQYKARVALENDE-RTEEEKYTAVQRT---
ATXN2-CALLORHINCHUS-MILII-A0A -YGVRSTYDSSLSSYTVPLERDNSEEFLRREARAAQLAEEIESSAQYKARVALENDE-RTEEEKYTAVQRT---
ATXN2-MOBULA-BIROSTRIS-UPI003 -YGVRSTYDSSLSSYTVPLERDNSEEFLRREARAAQLAEEIESSAQYKARAALENDE-RTEEEKYTAVQRS---
ATXN2-ENGRAULIS-ENCRASICOLUS- -YGVKSTYDSSLSSYTVPLERDSSEEFLKREQRAAQLADEIESSSTYKARVALENDE-RTEEDKFTAVVRG---
ATXN2-ICTALURUS-PUNCTATUS-A0A -YGVKSTYDSSLSSYTVPLERDNSEEFLKREARAAQLAEEIEASATYKARVALENDE-RSEEEKYSAVVRD---
ATXN2-SYNGNATHUS-TYPHLE-UPI00 -YGVLSTYDSSLSTYTVPLERDNSEEFLKREARAAQLAEEIEASSTYKARVALENDE-RSEEDKFTAVVRG---
ATXN2-DANIO-RERIO-A2CF31 -YGVKSTYDSSLSSYTVPLERDNSEEFLKREARASQLAEEIEASATYKARVALENDD-RSEEEKYTAVVRG---
ATXN2-PLATICHTHYS-FLESUS-UPI0 -YGVMSTYDSSLSTYTVPLERDNSEEFLKREARASQLAEEIEASATYKARVALENDE-RSEEEKYTAVVRG---
ATXN2-GASTEROSTEUS-ACULEATUS- -YGVLSTYDSSLSTYTVPLERDNSEEFLKREARAAQLAEEIEASATYKARVALENDE-RSEEEKYTAVVRG---
ATXN2-TAKIFUGU-RUBRIPES-ENSTR -YGVLSTYDSSLSTYTVPLERDNSEEFLKREARAAQLAEEIEASASYKARVALENDE-RSEEEKYTAVMRG---
ATXN2-CONGER-CONGER-A0A9Q1D9S -YGVKSTYDSSLASYTVPLERDNSEEFLKREARAAQLAEEIESSATYRARVALENDD-RSEEEKFTAVVRG---
ATXN2-LEPISOSTEUS-OCULATUS-UP -YGVKSTYDSSLSSYTVPLERDNSEEFLKREARAAQLAEEIESSSTYKARVALENDD-RSEEEKFTAVVRT---
ATXN2L-ERPETOICHTHYS-CALABARI -YGIKSTYDASLSLYTVPLEKGNSEQYRQREARAEKLASEIEASPQYRQRISMENDEGRSEEDKYSSVVRE---
ATXN2L-CONGER-CONGER-A0A9Q1DY -YGIKSTYDSSLSMYTVPLERGSTEGFRQREARAARLASEIESSPQYRHRVSLENEDGKTEEEKFSSVVRD---
ATXN2L-ENGRAULIS-ENCRASICOLUS -YGVKSTYDSSLSMYTVALDRSSNENFRARELRAARLASEIESSPQYRHRVALENDDGRTDEDKFSAVERD---
ATXN2L-DANIO-RERIO-A0A8M3AQQ2 -YGVKSTYDSSLSMYTVPLERGSSEGFRQREARAARLANEIEASSQYRHRVALENDEGRTDEDKFSAVVRD---
ATXN2L-ICTALURUS-PUNCTATUS-A0 -YGVKSTYDSSLSMYTVPLERGSSEGFRQREARAARLANEIEASAQYRHRVALENDEGRSEEDKFSAVIRD---
ATXN2L-PLATICHTHYS-FLESUS-UPI -YGVTSTYDASLSMYTVPLERGNSDTFRQREMRAARLANEIESTPQYRHRANLENDDGKSEEDKFSSVVRD---
ATXN2L-LEPISOSTEUS-OCULATUS-U -YGVKSTYDSSLSMYTVALERGSSELFRQREARAARLASEIESSPQYRRRVSLENDEGRSEEDKYGAVSRD---
ATXN2L-GASTEROSTEUS-ACULEATUS -YGVTSTYDASLSMYTVPLEKGNTDTFRQREARAARLANEIEASPQYRHRVGLENDEGKSEEDKYSAVVRD---
ATXN2L-TAKIFUGU-RUBRIPES-ENST -YGVTSTYDSSLSMYTVPLEKGNSEVFRQREARAARLASEIESSPQYRHRVNLENDEGKSEEDKYSAVVRD---
ATXN2L-SYNGNATHUS-TYPHLE-UPI0 -YGVTSTYDSSLSMYTVPLERGNSEVYRQREARAARLASEIESSPQYRHRVGLENDEGKSEEDKYSAVVRD---
ATXN2L-PETROMYZON-MARINUS-UPI -YGVKSTYDSSLANYTTRLDKDSSEEFQKREARAEQLAREIESSPQYRTHAALENDDGRTEEDRFSSVQRR---

LSMAD sequences used:

> ATXN2L-Xenopus-tropicalis-F7D5U2
YGVKTTYDSSLSSYTIPLEKDNSEEFRQREMRATQLAREIESSPQYRARIAIENDDCRTEEEKHSAVQRP

> ATXN2L-Danio-rerio-A0A8M3AQQ2
YGVKSTYDSSLSMYTVPLERGSSEGFRQREARAARLANEIEASSQYRHRVALENDEGRTDEDKFSAVVRD

> ATXN2L-Gasterosteus-aculeatus-G3NX14
YGVTSTYDASLSMYTVPLEKGNTDTFRQREARAARLANEIEASPQYRHRVGLENDEGKSEEDKYSAVVRD

> ATXN2L-Takifugu-rubripes-ENSTRUP00000017523
YGVTSTYDSSLSMYTVPLEKGNSEVFRQREARAARLASEIESSPQYRHRVNLENDEGKSEEDKYSAVVRD

> ATXN2L-Ictalurus-punctatus-A0A2D0T5S6
YGVKSTYDSSLSMYTVPLERGSSEGFRQREARAARLANEIEASAQYRHRVALENDEGRSEEDKFSAVIRD

> ATXN2L-Engraulis-encrasicolus-UPI002FD0E828
YGVKSTYDSSLSMYTVALDRSSNENFRARELRAARLASEIESSPQYRHRVALENDDGRTDEDKFSAVERD

> ATXN2L-Syngnathus-typhle-UPI002A69CFDD
YGVTSTYDSSLSMYTVPLERGNSEVYRQREARAARLASEIESSPQYRHRVGLENDEGKSEEDKYSAVVRD

> ATXN2L-Lepisosteus-oculatus-UPI003721E09F
YGVKSTYDSSLSMYTVALERGSSELFRQREARAARLASEIESSPQYRRRVSLENDEGRSEEDKYGAVSRD

> ATXN2L-Conger-conger-A0A9Q1DYQ3
YGIKSTYDSSLSMYTVPLERGSTEGFRQREARAARLASEIESSPQYRHRVSLENEDGKTEEEKFSSVVRD

> ATXN2L-Erpetoichthys-calabaricus-ENSECRP00000019267
YGIKSTYDASLSLYTVPLEKGNSEQYRQREARAEKLASEIEASPQYRQRISMENDEGRSEEDKYSSVVRE

> ATXN2L-Platichthys-flesus-UPI002DB70794
YGVTSTYDASLSMYTVPLERGNSDTFRQREMRAARLANEIESTPQYRHRANLENDDGKSEEDKFSSVVRD

> ATXN2L-Latimeria-chalumnae-ENSLACP00000022537
YGVKTTYDKSLSLYTIPLEKDNSDEFRQREARATQLAREIESNPQYRMRIAMENDDGKTEEEKHSAIVRA

> ATXN2L-Callorhinchus-milii-UPI0003D7AAAD
YGVRSTYDSSLSSYTVPLERDNSEEFLRREARAAQLAEEIESSAQYKARVALENDERTEEEKYTAVQRT

> ATXN2L-Scyliorhinus-torazame-UPI003B5C89BA
YGVKTTYDSSLSSYTTPLEKDNSAEFRQREARASQLAKEIESSPQYRVRISMENDDGRTEEEKFSAVQRP

> ATXN2L-Mobula-birostris-UPI003B284C37
YGVKTTYDSSLSSYTTPLEKDNSAEFRQREARATQLAKEIESSPQYRVRVSMENDDGRTEEEKFSAVQRP

> ATXN2L-Petromyzon-marinus-UPI001401C25F
YGVKSTYDSSLANYTTRLDKDSSEEFQKREARAEQLAREIESSPQYRTHAALENDDGRTEEDRFSSVQRR

> ATXN2L-Lethenteron-reissneri-UPI002AB6167E
YGVKSTYDSSLANYTTRLDKDSSEEFQKREARAEQLAREIESSPQYRTHAALENDDGRTEEDRFSSVQRR

> ATXN2-Xenopus-tropicalis-A0A6I8S7M6
YGVVSTYDSSLSSYTVPLERDNSEEYLKREARAAQIAEEIESSSQYKARVALENDERSEEEKYTAVQRS

> ATXN2-Danio-rerio-A2CF31
YGVKSTYDSSLSSYTVPLERDNSEEFLKREARASQLAEEIEASATYKARVALENDDRSEEEKYTAVVRG

> ATXN2-Gasterosteus-aculeatus-G3NSL2
YGVLSTYDSSLSTYTVPLERDNSEEFLKREARAAQLAEEIEASATYKARVALENDERSEEEKYTAVVRG

> ATXN2-Takifugu-rubripes-ENSTRUP00000043038
YGVLSTYDSSLSTYTVPLERDNSEEFLKREARAAQLAEEIEASASYKARVALENDERSEEEKYTAVMRG

> ATXN2-Ictalurus-punctatus-A0A2D0PP90
YGVKSTYDSSLSSYTVPLERDNSEEFLKREARAAQLAEEIEASATYKARVALENDERSEEEKYSAVVRD

> ATXN2-Engraulis-encrasicolus-UPI002FD529E2
YGVKSTYDSSLSSYTVPLERDSSEEFLKREQRAAQLADEIESSSTYKARVALENDERTEEDKFTAVVRG

> ATXN2-Syngnathus-typhle-UPI002A69B65C
YGVLSTYDSSLSTYTVPLERDNSEEFLKREARAAQLAEEIEASSTYKARVALENDERSEEDKFTAVVRG

> ATXN2-Conger-conger-A0A9Q1D9S5
YGVKSTYDSSLASYTVPLERDNSEEFLKREARAAQLAEEIESSATYRARVALENDDRSEEEKFTAVVRG

> ATXN2-Erpetoichthys-calabaricus-ENSECRP00000031581
YGVKSTYDSSLSSYTVPLERDNSEEFLKREARAAQLAEEIESSAVYKARVALENDDRTEEEKYTAVTRS

> ATXN2-Platichthys-flesus-UPI001A896A66
YGVMSTYDSSLSTYTVPLERDNSEEFLKREARASQLAEEIEASATYKARVALENDERSEEEKYTAVVRG

> ATXN2-Latimeria-chalumnae-ENSLACP00000014229
YGVRSTYDSSLSSYTVPLERDNSEEYLRRQARAAQLAEEIESSAQYKARVALENDDRTEEEKYTAVQRN

> ATXN2-Lepisosteus-oculatus-UPI0007402A6E
YGVKSTYDSSLSSYTVPLERDNSEEFLKREARAAQLAEEIESSSTYKARVALENDDRSEEEKFTAVVRT

> ATXN2-Callorhinchus-milii-A0A4W3HHQ2
YGVRSTYDSSLSSYTVPLERDNSEEFLRREARAAQLAEEIESSAQYKARVALENDERTEEEKYTAVQRT

> ATXN2-Scyliorhinus-torazame-A0A401NJM5
LESDVVNILIMNLDQVPLERDNSEEFLRREARAAQLAEEIESSAQYKARVALENDERTEEEKYTAVQRS

> ATXN2-Mobula-birostris-UPI003B280F2A
YGVRSTYDSSLSSYTVPLERDNSEEFLRREARAAQLAEEIESSAQYKARAALENDERTEEEKYTAVQRS

> ATXN2-Petromyzon-marinus-UPI0014023775
YGVKSTYDHSLSNYTTPLERDESEEFMRRQARATQLAQEIESNPSYRSRVSLENDEGRTEEERFSAVHRN

> ATXN2-Lethenteron-reissneri-UPI002AB6496F
YGVKSTYDHSLSNYTTPLERDESEEFMRRQARATQLAQEIESNPSYRSRVSLENDEGRTEEERFSAVHRN

> Ataxin-2-Branchiostoma-lanceolatum-UPI0034527580
YGVQSSYDSSLAGYTTQLKEEDTKEYREKRDRAQRIADEIERSTDYQRRVALEVHDDEEAAFSAVHRPSRN

> Ataxin-2-Ptychodera-flava-UPI00396A2C10
KFGVSSTYEENLSQYTTELEKKDTKEYKEREQEAERLAKEIESSPGHKVRQAKETEDGSEEDKFSAVHRPSNS

> Ataxin-2-Strongylocentrotus-purpuratus-UPI00026526AB
QMGVKSSYDSNLGDYTTPLDPKDTDDYQRKIIRADRLAKKIEGSSDYMVRTSKEQEHTEEDRFSAVVRPMNS

> Ataxin-2-Myxine-glutinosa-UPI00358E88F0
YGVKSTYDSTLSTYTTQLERDDSEEFQKREARAEQLAREIEATTQYRTHTNLEDDERSEEDKFGAVPRR

Ataxin-2 family PAM2 alignment in fish species before and after gene duplication

CLUSTAL O(1.2.4) multiple sequence alignment

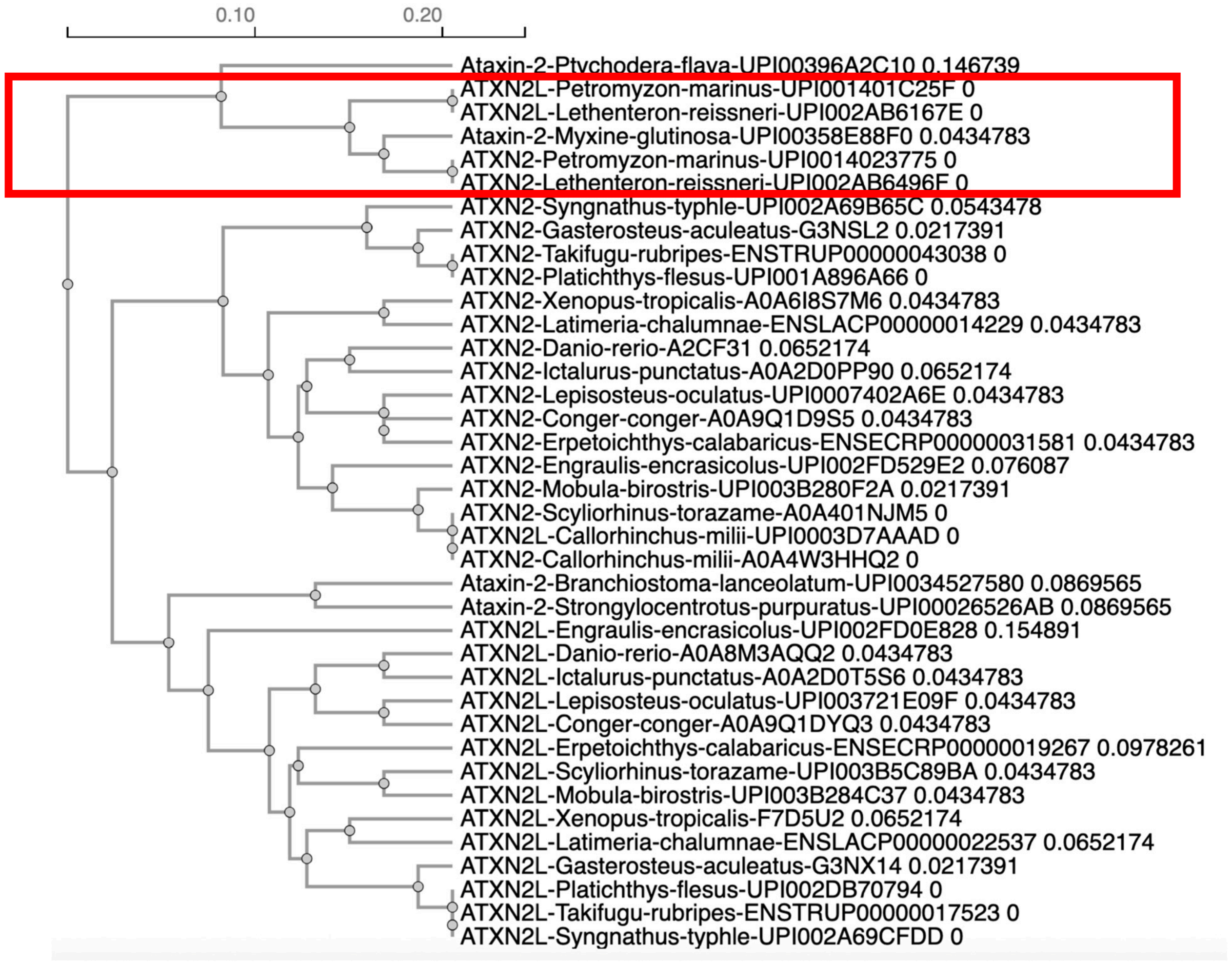

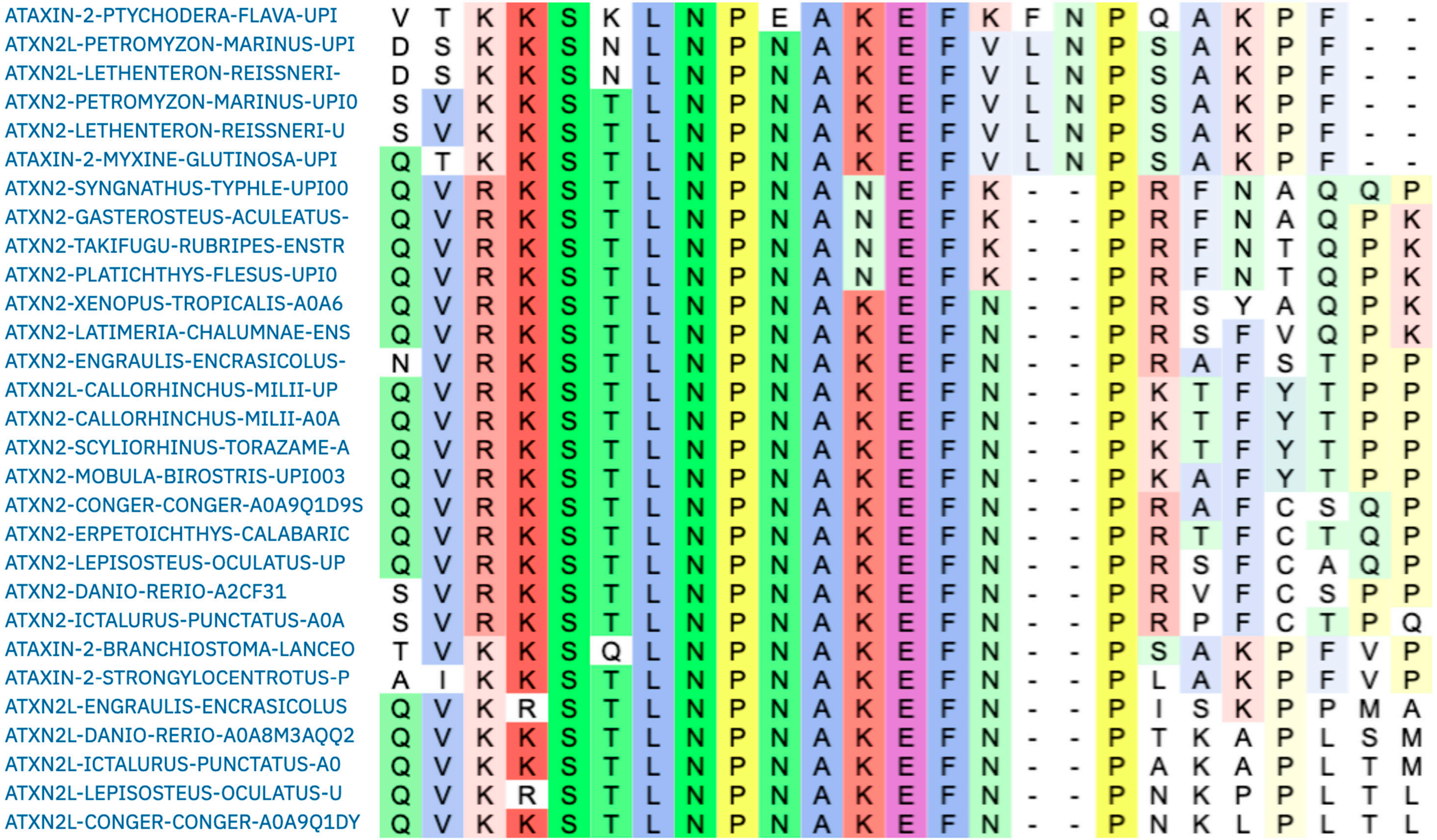
ATAXIN-2-PTYCHODERA-FLAVA-UPI V T K K S K L N P E A K E F K F N P Q A K P F - -
ATXN2L-PETROMYZON-MARINUS-UPI D S K K S N L N P N A K E F V L N P S A K P F - -
ATXN2L-LETHENTERON-REISSNERI- D S K K S N L N P N A K E F V L N P S A K P F - -
ATXN2-PETROMYZON-MARINUS-UPI0 S V K K S T L N P N A K E F V L N P S A K P F - -
ATXN2-LETHENTERON-REISSNERI-U S V K K S T L N P N A K E F V L N P S A K P F - -
ATAXIN-2-MYXINE-GLUTINOSA-UPI Q T K K S T L N P N A K E F V L N P S A K P F - -
ATXN2-SYNGNATHUS-TYPHLE-UPI00 Q V R K S T L N P N A N E F K - - P R F N A Q Q P
ATXN2-GASTEROSTEUS-ACULEATUS- Q V R K S T L N P N A N E F K - - P R F N A Q P K
ATXN2-TAKIFUGU-RUBRIPES-ENSTR Q V R K S T L N P N A N E F K - - P R F N T Q P K
ATXN2-PLATICHTHYS-FLESUS-UPI0 Q V R K S T L N P N A N E F K - - P R F N T Q P K
ATXN2-XENOPUS-TROPICALIS-A0A6 Q V R K S T L N P N A K E F N - - P R S Y A Q P K
ATXN2-LATIMERIA-CHALUMNAE-ENS Q V R K S T L N P N A K E F N - - P R S F V Q P K
ATXN2-ENGRAULIS-ENCRASICOLUS- N V R K S T L N P N A K E F N - - P R A F S T P P
ATXN2L-CALLORHINCHUS-MILII-UP Q V R K S T L N P N A K E F N - - P K T F Y T P P
ATXN2-CALLORHINCHUS-MILII-A0A Q V R K S T L N P N A K E F N - - P K T F Y T P P
ATXN2-SCYLIORHINUS-TORAZAME-A Q V R K S T L N P N A K E F N - - P K T F Y T P P
ATXN2-MOBULA-BIROSTRIS-UPI003 Q V R K S T L N P N A K E F N - - P K A F Y T P P
ATXN2-CONGER-CONGER-A0A9Q1D9S Q V R K S T L N P N A K E F N - - P R A F C S Q P
ATXN2-ERPETOICHTHYS-CALABARIC Q V R K S T L N P N A K E F N - - P R T F C T Q P
ATXN2-LEPISOSTEUS-OCULATUS-UP Q V R K S T L N P N A K E F N - - P R S F C A Q P
ATXN2-DANIO-RERIO-A2CF31 S V R K S T L N P N A K E F N - - P R V F C S P P
ATXN2-ICTALURUS-PUNCTATUS-A0A S V R K S T L N P N A K E F N - - P R P F C T P Q
ATAXIN-2-BRANCHIOSTOMA-LANCEO T V K K S Q L N P N A K E F N - - P S A K P F V P
ATAXIN-2-STRONGYLOCENTROTUS-P A I K K S T L N P N A K E F N - - P L A K P F V P
ATXN2L-ENGRAULIS-ENCRASICOLUS Q V K R S T L N P N A K E F N - - P I S K P P M A
ATXN2L-DANIO-RERIO-A0A8M3AQQ2 Q V K K S T L N P N A K E F N - - P T K A P L S M
ATXN2L-ICTALURUS-PUNCTATUS-A0 Q V K K S T L N P N A K E F N - - P A K A P L T M
ATXN2L-LEPISOSTEUS-OCULATUS-U Q V K R S T L N P N A K E F N - - P N K P P L T L
ATXN2L-CONGER-CONGER-A0A9Q1DY Q V K K S T L N P N A K E F N - - P N K L P L T L

PAM2 sequences used:

> ATXN2L-Xenopus-tropicalis-F7D5U2
QVKKSTLNPNAKEFNPSKPLLSV

> ATXN2L-Danio-rerio-A0A8M3AQQ2
QVKKSTLNPNAKEFNPTKAPLSM

> ATXN2L-Gasterosteus-aculeatus-G3NX14
QVKKSTLNPNAKEFNPIKPLMPM

> ATXN2L-Takifugu-rubripes-ENSTRUP00000017523
QVKKSTLNPNAKEFNPIKPQMPM

> ATXN2L-Ictalurus-punctatus-A0A2D0T5S6
QVKKSTLNPNAKEFNPAKAPLTM

> ATXN2L-Engraulis-encrasicolus-UPI002FD0E828
QVKRSTLNPNAKEFNPISKPPMA

> ATXN2L-Syngnathus-typhle-UPI002A69CFDD
QVKKSTLNPNAKEFNPIKPQMPM

> ATXN2L-Lepisosteus-oculatus-UPI003721E09F
QVKRSTLNPNAKEFNPNKPPLTL

> ATXN2L-Conger-conger-A0A9Q1DYQ3
QVKKSTLNPNAKEFNPNKLPLTL

> ATXN2L-Erpetoichthys-calabaricus-ENSECRP00000019267
QVKKSTLNPNAKEFNPTKPIVTA

> ATXN2L-Platichthys-flesus-UPI002DB70794
QVKKSTLNPNAKEFNPIKPQMPM

> ATXN2L-Latimeria-chalumnae-ENSLACP00000022537
QVKKSTLNPNAKEFNPLKPMLPV

> ATXN2L-Callorhinchus-milii-UPI0003D7AAAD
QVRKSTLNPNAKEFNPKTFYTPP

> ATXN2L-Scyliorhinus-torazame-UPI003B5C89BA
QVKKSTLNPNAKEFNPTKTLVPV

> ATXN2L-Mobula-birostris-UPI003B284C37
QVKKSTLNPNAKEFNPTKTHIPV

> ATXN2L-Petromyzon-marinus-UPI001401C25F
DSKKSNLNPNAKEFVLNPSAKPF

> ATXN2L-Lethenteron-reissneri-UPI002AB6167E
DSKKSNLNPNAKEFVLNPSAKPF

> ATXN2-Xenopus-tropicalis-A0A6I8S7M6
QVRKSTLNPNAKEFNPRSYAQPK

> ATXN2-Danio-rerio-A2CF31
SVRKSTLNPNAKEFNPRVFCSPP

> ATXN2-Gasterosteus-aculeatus-G3NSL2
QVRKSTLNPNANEFKPRFNAQPK

> ATXN2-Takifugu-rubripes-ENSTRUP00000043038
QVRKSTLNPNANEFKPRFNTQPK

> ATXN2-Ictalurus-punctatus-A0A2D0PP90
SVRKSTLNPNAKEFNPRPFCTPQ

> ATXN2-Engraulis-encrasicolus-UPI002FD529E2
NVRKSTLNPNAKEFNPRAFSTPP

> ATXN2-Syngnathus-typhle-UPI002A69B65C
QVRKSTLNPNANEFKPRFNAQQP

> ATXN2-Conger-conger-A0A9Q1D9S5
QVRKSTLNPNAKEFNPRAFCSQP

> ATXN2-Erpetoichthys-calabaricus-ENSECRP00000031581
QVRKSTLNPNAKEFNPRTFCTQP

> ATXN2-Platichthys-flesus-UPI001A896A66
QVRKSTLNPNANEFKPRFNTQPK

> ATXN2-Latimeria-chalumnae-ENSLACP00000014229
QVRKSTLNPNAKEFNPRSFVQPK

> ATXN2-Lepisosteus-oculatus-UPI0007402A6E
QVRKSTLNPNAKEFNPRSFCAQP

> ATXN2-Callorhinchus-milii-A0A4W3HHQ2
QVRKSTLNPNAKEFNPKTFYTPP

> ATXN2-Scyliorhinus-torazame-A0A401NJM5
QVRKSTLNPNAKEFNPKTFYTPP

> ATXN2-Mobula-birostris-UPI003B280F2A
QVRKSTLNPNAKEFNPKAFYTPP

> ATXN2-Petromyzon-marinus-UPI0014023775
SVKKSTLNPNAKEFVLNPSAKPF

> ATXN2-Lethenteron-reissneri-UPI002AB6496F
SVKKSTLNPNAKEFVLNPSAKPF

> Ataxin-2-Branchiostoma-lanceolatum-UPI0034527580
TVKKSQLNPNAKEFNPSAKPFVP

> Ataxin-2-Ptychodera-flava-UPI00396A2C10
VTKKSKLNPEAKEFKFNPQAKPF

> Ataxin-2-Strongylocentrotus-purpuratus-UPI00026526AB
AIKKSTLNPNAKEFNPLAKPFVP

> Ataxin-2-Myxine-glutinosa-UPI00358E88F0
QTKKSTLNPNAKEFVLNPSAKPF
