# Supplementary material for: Bioinformatic Analyses of the Ataxin-2 Family Since Algae Emphasize Its Small Isoforms, Large Chimerisms, and the Importance of Human Exon 1B as Target of Therapies to Prevent Neurodegeneration
Source: Int J Mol Sci. 2026 Feb 3;27(3):1499. doi: 10.3390/ijms27031499 (PMC12898128; doi:10.3390/ijms27031499)
Supplement: Supplementary file 1 [file ijms-27-01499-s001.zip › AuburgerSen_SupplMaterialS2-ArabidopsisThalianaMultipleSequenceAlignment-CID4-CID3.pdf]

Ataxin-2 family LSM alignment in plant species before and after gene duplication

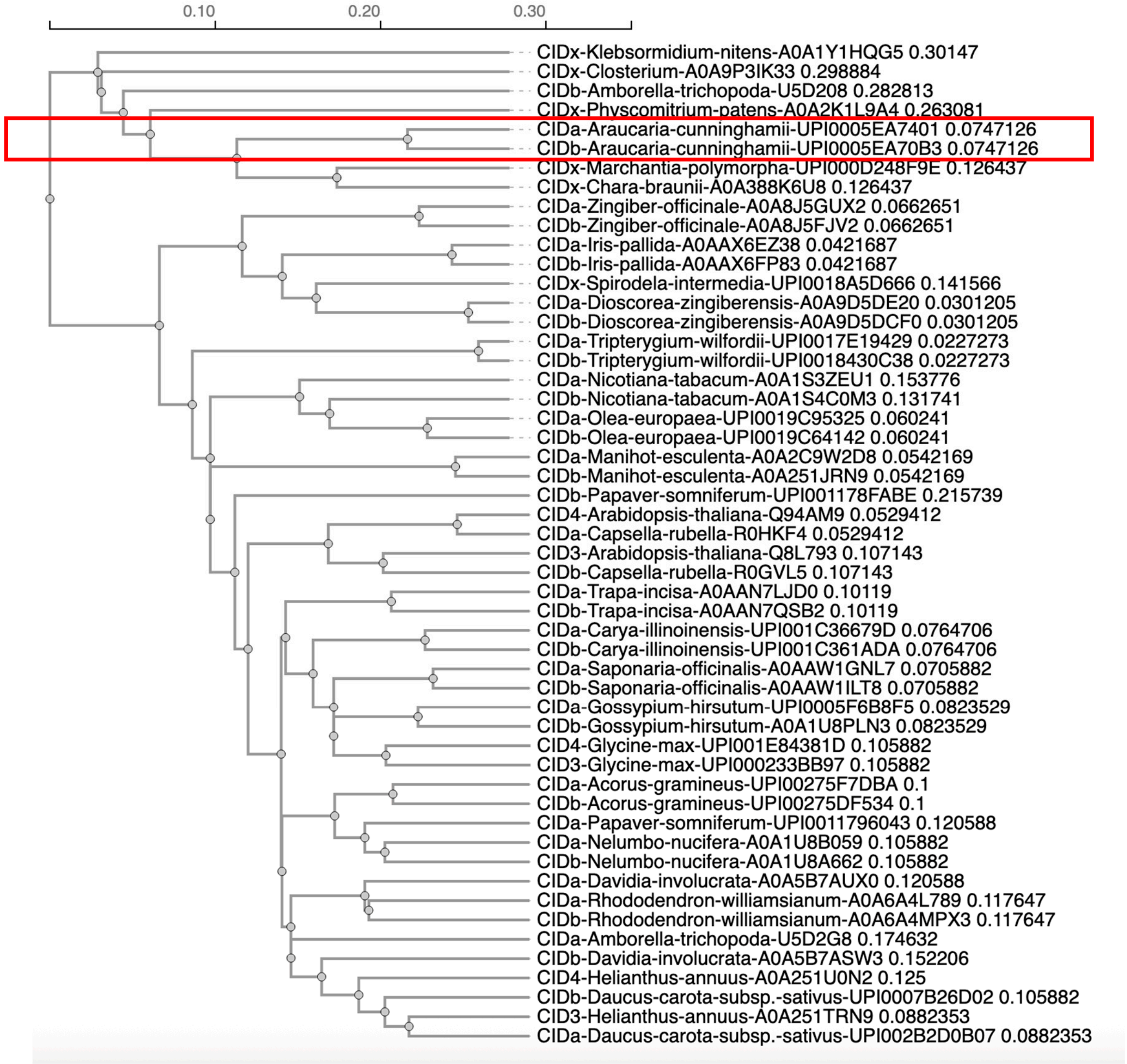

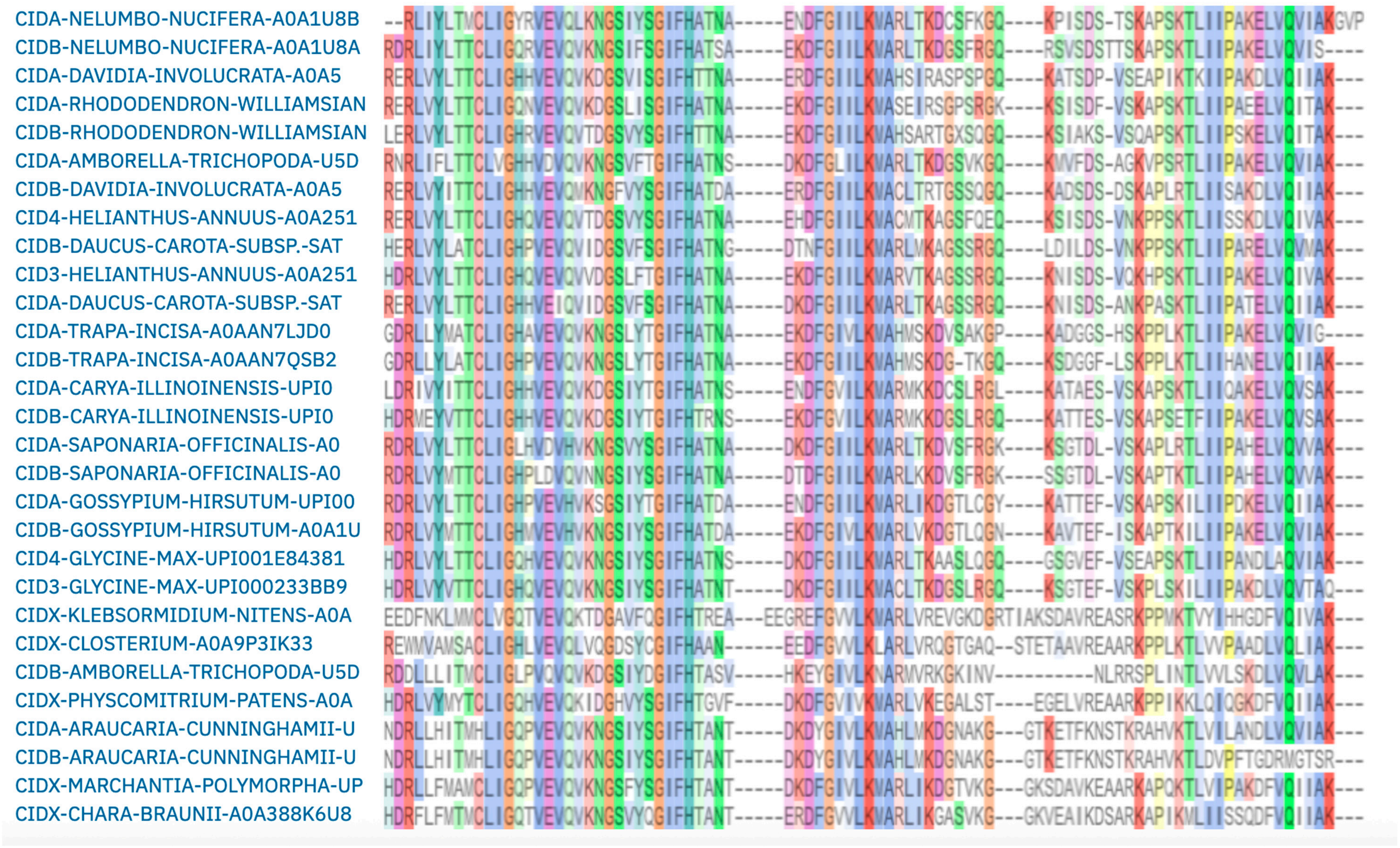
CIDA-NELUMBO-NUCIFERA-A0A1U8B
CIDB-NELUMBO-NUCIFERA-A0A1U8A
CIDA-DAVIDIA-INVOLUCRATA-A0A5
CIDA-RHODODENDRON-WILLIAMSIAN
CIDB-RHODODENDRON-WILLIAMSIAN
CIDA-AMBORELLA-TRICHOPODA-U5D
CIDB-DAVIDIA-INVOLUCRATA-A0A5
CID4-HELIANTHUS-ANNUUS-A0A251
CIDB-DAUCUS-CAROTA-SUBSP.-SAT
CID3-HELIANTHUS-ANNUUS-A0A251
CIDA-DAUCUS-CAROTA-SUBSP.-SAT
CIDA-TRAPA-INCISA-A0AAN7LJD0
CIDB-TRAPA-INCISA-A0AAN7QSB2
CIDA-CARYA-ILLINOINENSIS-UPI0
CIDB-CARYA-ILLINOINENSIS-UPI0
CIDA-SAPONARIA-OFFICINALIS-A0
CIDB-SAPONARIA-OFFICINALIS-A0
CIDA-GOSSYPIUM-HIRSUTUM-UPI00
CIDB-GOSSYPIUM-HIRSUTUM-A0A1U
CID4-GLYCINE-MAX-UPI001E84381
CID3-GLYCINE-MAX-UPI000233BB9
CIDX-KLEBSORMIDIUM-NITENS-A0A
CIDX-CLOSTERIUM-A0A9P3IK33
CIDB-AMBORELLA-TRICHOPODA-U5D
CIDX-PHYSCOMITRIUM-PATENS-A0A
CIDA-ARAUCARIA-CUNNINGHAMII-U
CIDB-ARAUCARIA-CUNNINGHAMII-U
CIDX-MARCHANTIA-POLYMORPHA-UP
CIDX-CHARA-BRAUNII-A0A388K6U8

LSM sequences used:

> CID4-Arabidopsis-thaliana-Q94AM9
LDRLVYFTTCKIGHHVEVHLRNGSVYTGIFHAANVEKDFGIILKMACLIKDGTLRGHKSRSEFVRKPPSKTFIIPADELVQVIAK

> CID3-Arabidopsis-thaliana-Q8L793
QKLLVYFTTCNIGHQVEVHLKNGSVYSGIFHAANVEKDFGIILKMACLIRDSRGTKSRTVSKPSSKLLKIPADELVQVIAKDLP

> CIDa-Capsella-rubella-R0HKF4
RDRLVYLTTCKIGHHVEVHLKNGSMYIGIFHAADVDKDFGIILKMACLIKEGTLRGHKSRSEFVRKPPSKTFIIPADELVQVVAK

> CIDb-Capsella-rubella-R0GVL5
RLLYFTTCKIGHQVEVHLKNGSIYSGIFHAANVDNDFGIILKMACLIKDSRGAKSRSSLVSKPPSKRLIIPAEELVQVIAKDLP

> CIDa-Gossypium-hirsutum-UPI0005F6B8F5
RDRLVYLTTCLIGHPVEVHVKSGSIYTGIFHATDAENDFGIILKMARLIKDGTLCGYKATTEFVSKAPSKILIIPDKELVQIIAK

> CIDb-Gossypium-hirsutum-A0A1U8PLN3
RDRLVYMTTCLIGHMVEVHVKNGSIYSGIFHATDAEKDFGIVLKMARLVKDGTLQGNKAVTEFISKAPTKILIIPAKELVQVIAK

> CIDa-Manihot-esculenta-A0A2C9W2D8
RDRLVYLSTCLIGHPVEVHLKNGCIYSGTCYTTNVEKEFAIVLKMARLIKDVSFRGQKAENLSKPPSKSLIIPGKEVAQVIAK

> CIDb-Manihot-esculenta-A0A251JRN9
RDRLVYLSACLIGHPVEVHLKNGSIYSGTCYTTNVEKEFAIILKMARLTKDVSFRGQKAETLSKAPSKTLIIPGKEVVQVLAK

> CIDa-Nelumbo-nucifera-A0A1U8B059
RLIYLTMCLIGYRVEVQLKNGSIYSGIFHATNAENDFGIILKMARLTKDCSFKGQKPISDSTSKAPSKTLIIPAKELVQVIAKGVP

> CIDb-Nelumbo-nucifera-A0A1U8A662
RDRLIYLTTCLIGQRVEVQVKNGSIFSGIFHATSAEKDFGIILKMARLTKDGSFRGQRSVSDSTTSKAPSKTLIIPAKELVQVIS

> CIDa-Saponaria-officinalis-A0AAW1GNL7
RDRLVYLTTCLIGLHVDVHVKNGSVYSGIFHATNADKDFGIILKMARLTKDVSFRGKKSGTDLVSKAPLRTLIIPAHELVQVVAK

> CIDb-Saponaria-officinalis-A0AAW1ILT8
RDRLVYMTTCLIGHPLDVQVNNGSIYSGIFHATNADTDFGIILKMARLKKDVSFRGKSSGTDLVSKAPTKTLIIPAHELVQVVAK

> CIDa-Rhododendron-williamsianum-A0A6A4L789
RERLVYLTTCLIGQNVEVQVKDGSLISGIFHATNAEKDFGIILKMASEIRSGPSRGKKSISDFVSKAPSKTLIIPAEELVQITAK

> CIDb-Rhododendron-williamsianum-A0A6A4MPX3
LERLVYLTTCLIGHRVEVQVTDGSVYSGIFHTTNAEKDFGIILKMAHSARTGXSQGQKSIAKSVSQAPSKTLIIPSKELVQITAK

> CIDa-Nicotiana-tabacum-A0A1S3ZEU1
RDRLIYLTTCLVGHQVEVQVLDGSVFSGILHAANTEKDSGIILKMAHLIKDCTEGMKSTSETFSKPPSKTLIIPGKEFVQVTAK

> CIDb-Nicotiana-tabacum-A0A1S4C0M3
HDRLVYFSTCLLGHEVEVQILDGSVFSGIFHATNADKDFGIILKMAHLIKDGSQGRKNTPESLIKPPTKTLIIPGKELVQVIAK

> CID4-Helianthus-annuus-A0A251U0N2
RERLVYLTTCLIGHQVEVQVTDGSVYSGIFHATNAEHDFGIILKMACMTKAGSFQEQKSISDSVNKPPSKTLIISSKDLVQIVAK

> CID3-Helianthus-annuus-A0A251TRN9
HDRLVYLTTCLIGHQVEVQVVDGSLFTGIFHATNAEKDFGIILKMARVTKAGSSRGQKNISDSVQKHPSKTLIIPAKELVQIVAK

> CID4-Glycine-max-UPI001E84381D
HDRLVYLTTCLIGQHVEVQVKNGSIYSGIFHATNSDKDFGIILKMARLTKAASLQGQGSGVEFVSEAPSKTLIIPANDLAQVIAK

> CID3-Glycine-max-UPI000233BB97
HDRLVYVTTCLIGHQVEVQVKNGSIYSGIFHATNTDKDFGIILKMACLTKDGSLRGQKSGTEFVSKPLSKILIIPAKDLVQVTAQ

> CIDa-Iris-pallida-A0AAX6EZ38
RDRLIYVMTSLIGLPVDVHIRNGSTISGIFHATNAEKDFGIVLKMAQVIKDGSVRGQKPVSETVKKPRDMFIQSKDIVQVVAK

> CIDb-Iris-pallida-A0AAX6FP83
RDRLIYVMTSLIGLPVDVHIRNGSIISGIFHATNAEKDFGIVLKMAQIIKDGSARGQKSVPDTVKKPRDMFIQSKDLVQVVAK

> CIDa-Araucaria-cunninghamii-UPI0005EA7401
NDRLLHITMHLIGQPVEVQVKNGSIYSGIFHTANTDKDYGIVLKMAHLMKDGNAKGGTKETFKNSTKRAHVKTLVILANDLVQVIAK

> CIDb-Araucaria-cunninghamii-UPI0005EA70B3 / A0A0D6QXC6
NDRLLHITMHLIGQPVEVQVKNGSIYSGIFHTANTDKDYGIVLKMAHLMKDGNAKGGTKETFKNSTKRAHVKTLDVPFTGDRMGTSR

> CIDa-Acorus-gramineus-UPI00275F7DBA
RDRLTYLSTCIIGHHVEVQVQNGCIFSGILHAISAENDFGVILKMARLTKDGSTKGQKSLADSISRAPTKTLIIPAKELVQVIAK

> CIDb-Acorus-gramineus-UPI00275DF534
HDRLTYITTCIIGHRVEVQVKNGSVFSGILHAINTENDSGVILKSARLTKDGSTKGPKSLPDTVTKAPTKTLIIPAKELVQVIAK

> CIDa-Tripterygium-wilfordii-UPI0017E19429
HDRLVYMTTCLIGQPVQVQLKDGSIYSGIFHAGLVHPSTADREFGVILKMACLTKDGSSRWQRAELVNKPPTKTFIIPGKELVQVVAK

> CIDb-Tripterygium-wilfordii-UPI0018430C38
RDRLVYMTTCLIGQPVEVQLKDGSIYSGIFHAGLVHPSTADREFGVILKMACLTKDGSSRWPRAELVNKPPTKTFIIPGKELVQVLAK

> CIDa-Carya-illinoinensis-UPI001C36679D
LDRIVYITTCLIGHHVEVQVKDGSIYTGIFHATNSENDFGVILKMARMKKDCSLRGLKATAESVSKAPSKTLIIQAKELVQVSAK

> CIDb-Carya-illinoinensis-UPI001C361ADA
HDRMEYVTTCLIGHHVEVQVKDGSIYTGIFHTRNSEKDFGVILKMARMKKDGSLRGQKATTESVSKAPSETFIIPAKELVQVSAK

> CIDa-Trapa-incisa-A0AAN7LJD0
GDRLLYMATCLIGHAVEVQVKNGSLYTGIFHATNAEKDFGIVLKMAHMSKDVSAKGPKADGGSHSKPPLKTLIIPAKELVQVIG

> CIDb-Trapa-incisa-A0AAN7QSB2
GDRLLYLATCLIGHPVEVQVKNGSLYTGIFHATNAEKDFGIILKMAHMSKDGTKGQKSDGGFLSKPPLKTLIIHANELVQIIAK

> CIDa-Olea-europaea-UPI0019C95325
RDRLIYLTTCLIGHQVDVQVLDGSVFSGIFHATNADKDFGIILKLAHLIKDGSQGQKNIFDSVNKTPRTLIISAKELVQVIAK

> CIDb-Olea-europaea-UPI0019C64142
RDRMIYLTTCLIGHQVEVQVSDGSVFSGIFHATNADKDFGIVLKMAHLIKDGSQGQKNIFDSVNKTCSRTLIISTKELVQVIAK

> CIDa-Daucus-carota-subsp.-sativus-UPI002B2D0B07
RERLVYLTTCLIGHHVEIQVIDGSVFSGIFHATNADKDFGIILKMARLTKAGSSRGQKNISDSANKPASKTLIIPATELVQIIAK

> CIDb-Daucus-carota-subsp.-sativus-UPI0007B26D02
HERLVYLATCLIGHPVEVQVIDGSVFSGIFHATNGDTNFGIILKMARLMKAGSSRGQLDILDSVNKPPSKTLIIPARELVQVMAK

> CIDa-Davidia-involucrata-A0A5B7AUX0
RERLVYLTTCLIGHHVEVQVKDGSVISGIFHTTNAERDFGIILKMAHSIRASPSPGQKATSDPVSEAPIKTKIIPAKDLVQIIAK

> CIDb-Davidia-involucrata-A0A5B7ASW3
RERLVYITTCLIGHHVEVQMKNGFVYSGIFHATDAERDFGIILKMACLTRTGSSQGQKADSDSDSKAPLRTLIISAKDLVQIIAK

> CIDa-Papaver-somniferum-UPI0011796043
RDRLIYLAACLIGHRVEVQVKNGSIFSGILHAMNAENDFGIVLKMASLTKDGLSKGHKPGSDSISKAPSKTLIVPAKELVQVIAK

> CIDb-Papaver-somniferum-UPI001178FABE
HDRLIYVVTCLIGQQVEVYVKNGSVFSGIFHSTNAASDDKDFGIILKMARLVKDGSCEGQNFDPDIITVPPSKTLIIPATELVQVLAK

> CIDa-Zingiber-officinale-A0A8J5GUX2
RDRLIYVLSFLIGHHVEVHVKNGSIISGIFHATNADRDFEIVLKMAQVVKDASMREQKSSRDNITTPQLMIVPTRELVQLLAK

> CIDb-Zingiber-officinale-A0A8J5FJV2
RDRLIYVLSLLIGRHVEVHVKNGSIISGILHATNADRDFEIVLKMAQVVKDGSVREQKSFRDSITSSRLMIVPARELVQLLAK

> CIDa-Dioscorea-zingiberensis-A0A9D5DE20
HDRLIFVTSCLIGQTVEVHVRNGSIISGIFHTSNAENDFGIILKMARVIKDGSSKGQKTIPDIIKKPQTMIIPGREVVQVLAK

> CIDb-Dioscorea-zingiberensis-A0A9D5DCF0
HDRLIFVTSCLIGQTVEVHVRNGSIISGIFHTSNAENDFGIILKMAQVIKDGSSKGQKPVSDIVKKPQTMIIPGREVVQVLAK

> CIDa-Amborella-trichopoda-U5D2G8
RNRLIFLTTCLVGHHVDVQVKNGSVFTGIFHATNSDKDFGLILKMARLTKDGSVKGQKMVFDSAGKVPSRTLIIPAKELVQVIAK

> CIDb-Amborella-trichopoda-U5D208
RDDLLLITMCLIGLPVQVQVKDGSIYDGIFHTASVHKEYGIVLKNARMVRKGKINVNLRRSPLINTLVVLSKDLVQVLAK

> CIDx-Physcomitrium-patens-A0A2K1L9A4
HDRLVYMYTCLIGQHVEVQKIDGHVYSGIFHTGVFDKDFGVIVKMARLVKEGALSTEGELVREAARKPPIKKLQIQGKDFVQIIAK

> CIDx-Marchantia-polymorpha-UPI000D248F9E
HDRLLFMAMCLIGQPVEVQVKNGSFYSGIFHTANTDKDFGIVLKMARLIKDGTVKGGKSDAVKEAARKAPQKTLVIPAKDFVQIIAK

> CIDx-Chara-braunii-A0A388K6U8
HDRFLFMTMCLIGQTVEVQVKNGSVYQGIFHTANTERDFGVVLKMARLIKGASVKGGKVEAIKDSARKAPIKMLIISSQDFVQIIAK

> CIDx-Closterium-A0A9P3IK33

REWMVAMSACLIGHLVEVQLVQGDSYCGIFHAANEEDFGVVLKLARLVRQGTGAQSTETAAVREAARKPPLKTLVVPAADLVQLIAK

> CIDx-Klebsormidium-nitens-A0A1Y1HQG5

EEDFNKLMMCLVGQTVEVQKTDGAVFQGIFHTREAEEGREFGVVLKMARLVREVGKDGRTIAKSDAVREASRKPPMKTVYIHHGDFVQIVAK

> CIDx-Spirodela-intermedia-UPI0018A5D666

RDRLIFVSTCLIGHSVEVHVKNGSVLSGIFHTINAEKDFGIVLKMARITKDGSVRGPKPVPETVKKPQTMIIPARELVQVIAK

> CIDa-Sorghum-bicolor-A0A1Z5RNJ3

RLIYVLTQLIGHHVDVHVKNGSIISGIFHATNSDKDFGVVLKMAQVIKDGSARGQRYSDDVVKKPETMIIPARELVQVFAKDVA

> CIDb-Sorghum-bicolor-C5YG86

EALLLATVCMVGLPVEVRVRDGSAYAGVLHTACVDAGYGVVLKKAKKIANGKGDANLSLGSFVDTLVVHPDDLVQVIAK

> CID3-Vitis-vinifera-D7T8B6

SQALVFATMCIIGLPVEVHVKDGSVYSGILHTACLGKDYGIILKKARMIKKGKLEANVAHGGMVETLVILTGDLVQVVAK

> CID4-Vitis-vinifera-A0A438K7K9

RDRLVYLTTCFIGLPVEVQVKNGSIISGIFHATNADKDFGIVLKMARLTKDGPVRGQKAISDSVSKAPSKILIIPAKELVQVIAK

> CIDa-Citrus-clementina-V4SRU4

HDKLLYLASCLIGLSVEVQVKSGSVYAGIFHATSDEKDFGVILKMARLIKDGNFRGQKTVAEFVSKPPSKNFIIPSSELVQVIAK

> CIDb-Citrus-clementina-V4WG04

SEALLFATMCIIGLPVDVYIKDGSVYSGIFYTASVEKDYGIVLKKAKMSKKGKSNANVANGTVIETLVILSADLVQVVAK

Ataxin-2 family LSMAD alignment in plant species before and after gene duplication

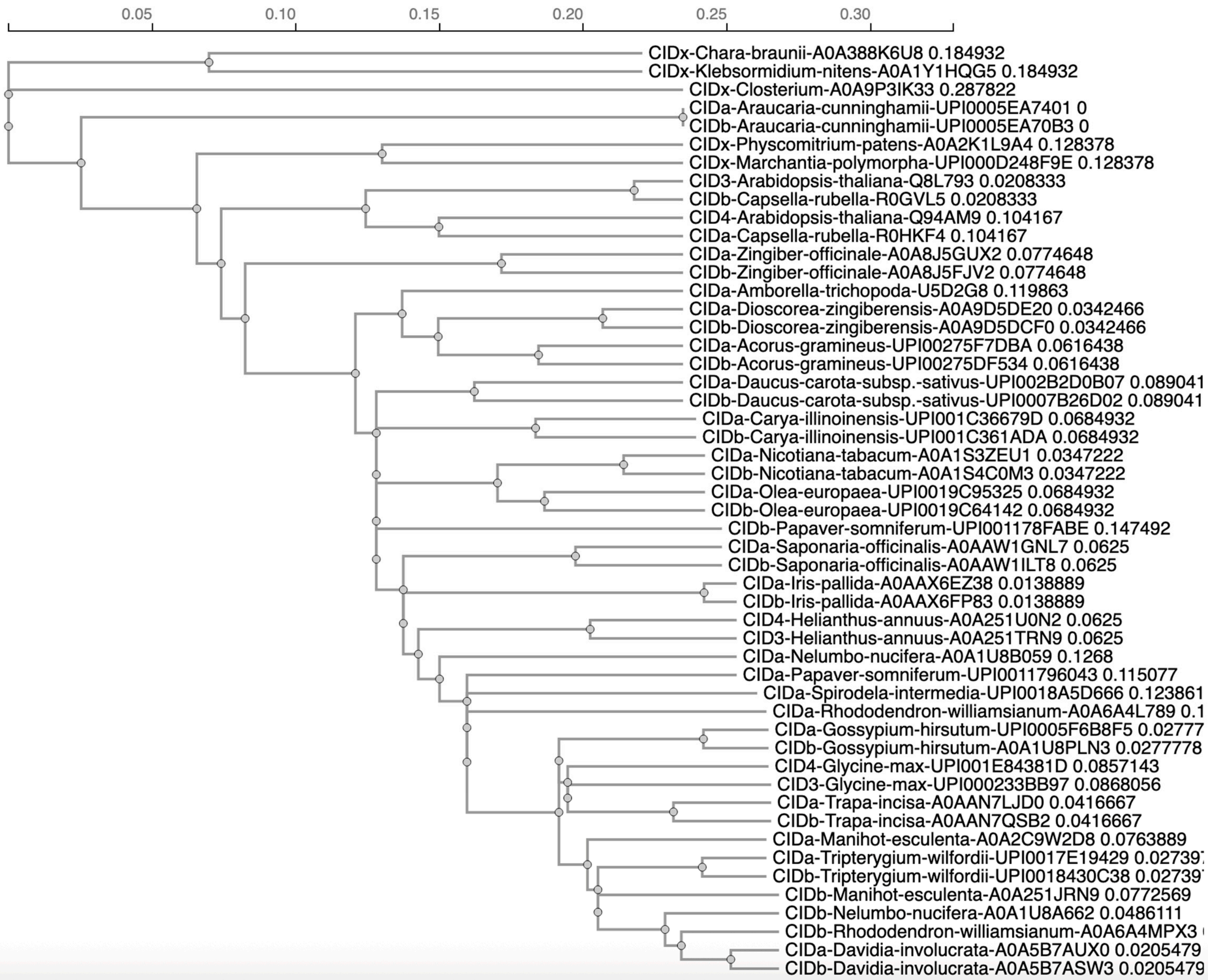
0.05
0.10
0.15
0.20
0.25
0.30
CIDx-Chara-braunii-A0A388K6U8 0.184932
CIDx-Klebsormidium-nitens-A0A1Y1HQG5 0.184932
CIDx-Closterium-A0A9P3IK33 0.287822
CIDa-Araucaria-cunninghamii-UPI0005EA7401 0
CIDb-Araucaria-cunninghamii-UPI0005EA70B3 0
CIDx-Physcomitrium-patens-A0A2K1L9A4 0.128378
CIDx-Marchantia-polymorpha-UPI000D248F9E 0.128378
CID3-Arabidopsis-thaliana-Q8L793 0.0208333
CIDb-Capsella-rubella-R0GVL5 0.0208333
CID4-Arabidopsis-thaliana-Q94AM9 0.104167
CIDa-Capsella-rubella-R0HKF4 0.104167
CIDa-Zingiber-officinale-A0A8J5GUX2 0.0774648
CIDb-Zingiber-officinale-A0A8J5FJV2 0.0774648
CIDa-Amborella-trichopoda-U5D2G8 0.119863
CIDa-Dioscorea-zingiberensis-A0A9D5DE20 0.0342466
CIDb-Dioscorea-zingiberensis-A0A9D5DCF0 0.0342466
CIDa-Acorus-gramineus-UPI00275F7DBA 0.0616438
CIDb-Acorus-gramineus-UPI00275DF534 0.0616438
CIDa-Daucus-carota-subsp.-sativus-UPI002B2D0B07 0.089041
CIDb-Daucus-carota-subsp.-sativus-UPI0007B26D02 0.089041
CIDa-Carya-illinoinensis-UPI001C36679D 0.0684932
CIDb-Carya-illinoinensis-UPI001C361ADA 0.0684932
CIDa-Nicotiana-tabacum-A0A1S3ZEU1 0.0347222
CIDb-Nicotiana-tabacum-A0A1S4C0M3 0.0347222
CIDa-Olea-europaea-UPI0019C95325 0.0684932
CIDb-Olea-europaea-UPI0019C64142 0.0684932
CIDb-Papaver-somniferum-UPI001178FABE 0.147492
CIDa-Saponaria-officinalis-A0AAW1GNL7 0.0625
CIDb-Saponaria-officinalis-A0AAW1ILT8 0.0625
CIDa-Iris-pallida-A0AAX6EZ38 0.0138889
CIDb-Iris-pallida-A0AAX6FP83 0.0138889
CID4-Helianthus-annuus-A0A251U0N2 0.0625
CID3-Helianthus-annuus-A0A251TRN9 0.0625
CIDa-Nelumbo-nucifera-A0A1U8B059 0.1268
CIDa-Papaver-somniferum-UPI0011796043 0.115077
CIDa-Spirodela-intermedia-UPI0018A5D666 0.123861
CIDa-Rhododendron-williamsianum-A0A6A4L789 0.1
CIDa-Gossypium-hirsutum-UPI0005F6B8F5 0.02777
CIDb-Gossypium-hirsutum-A0A1U8PLN3 0.0277778
CID4-Glycine-max-UPI001E84381D 0.0857143
CID3-Glycine-max-UPI000233BB97 0.0868056
CIDa-Trapa-incisa-A0AAN7LJD0 0.0416667
CIDb-Trapa-incisa-A0AAN7QSB2 0.0416667
CIDa-Manihot-esculenta-A0A2C9W2D8 0.0763889
CIDa-Tripterygium-wilfordii-UPI0017E19429 0.02739
CIDb-Tripterygium-wilfordii-UPI0018430C38 0.02739
CIDb-Manihot-esculenta-A0A251JRN9 0.0772569
CIDb-Nelumbo-nucifera-A0A1U8A662 0.0486111
CIDb-Rhododendron-williamsianum-A0A6A4MPX3
CIDa-Davidia-involucrata-A0A5B7AUX0 0.0205479
CIDb-Davidia-involucrata-A0A5B7ASW3 0.0205479

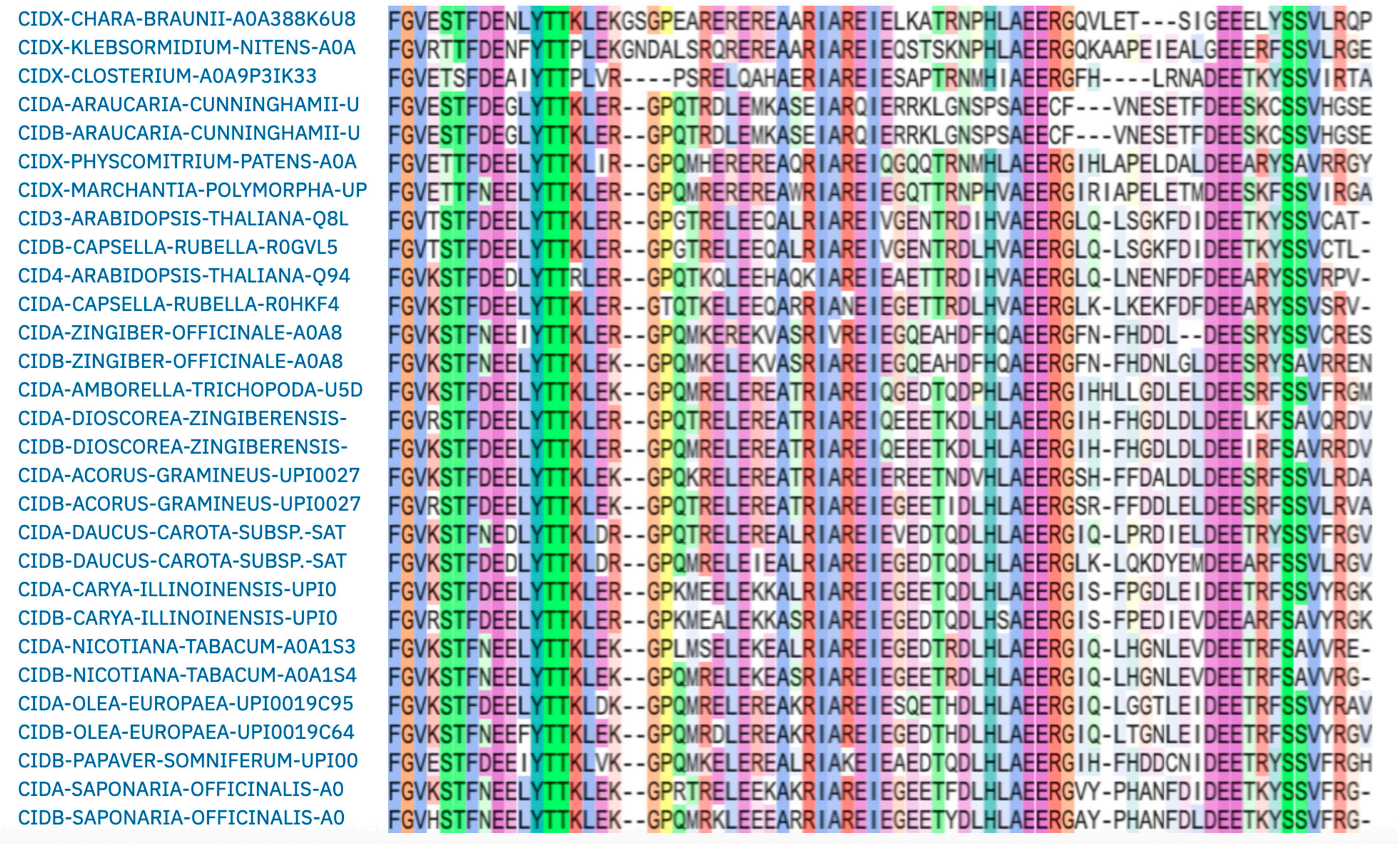
CIDX-CHARA-BRAUNII-A0A388K6U8 FGVESTFDENLYTTKLEKGSGPEAREREREAARIAREIELKATRNPHLAEERGQVLET---SIGEEELYSSVLRQP
CIDX-KLEBSORMIDIUM-NITENS-A0A FGVRTTFDENFYTTPLEKGNDALSRQREREAARIAREIEQSTSKNPHLAEERGQKAAPEIEALGEEERFSSVLRGE
CIDX-CLOSTERIUM-A0A9P3IK33 FGVETSFDEAIYTTPLVR----PSRELQAHAERIAREIESAPTRNMHIAEERGFH----LRNADEETKYSSVIRTA
CIDA-ARAUCARIA-CUNNINGHAMII-U FGVESTFDEGLYTTKLER--GPQTRDLEMKASEIARQIERRKLGNSPSAEECF---VNESETFDEESKCSSVHGSE
CIDB-ARAUCARIA-CUNNINGHAMII-U FGVESTFDEGLYTTKLER--GPQTRDLEMKASEIARQIERRKLGNSPSAEECF---VNESETFDEESKCSSVHGSE
CIDX-PHYSCOMITRIUM-PATENS-A0A FGVETTFDEELYTTKLIR--GPQMHEREREAQRIAREIQGQQTRNMHLAEERGIHLAPELDALDEEARYSAVRRGY
CIDX-MARCHANTIA-POLYMORPHA-UP FGVETTFNEELYTTKLER--GPQMREREREAWRIAREIEGQTTRNPHVAEERGIRIAPELETMDEESKFSSVIRGA
CID3-ARABIDOPSIS-THALIANA-Q8L FGVTSTFDEELYTTKLER--GPGTRELEEQALRIAREIVGENTRDIHVAEERGLQ-LSGKFDIDEETKYSSVCAT-
CIDB-CAPSELLA-RUBELLA-R0GVL5 FGVTSTFDEELYTTKLER--GPGTRELEEQALRIAREIVGENTRDLHVAEERGLQ-LSGKFDIDEETKYSSVCTL-
CID4-ARABIDOPSIS-THALIANA-Q94 FGVKSTFDEDLYTTRLER--GPQTKQLEEHAQKIAREIEAETTRDIHVAEERGLQ-LNENFDFDEEARYSSVRPV-
CIDA-CAPSELLA-RUBELLA-R0HKF4 FGVKSTFDEELYTTKLER--GTQTKELEEQARRIANEIEGETTRDLHVAEERGLK-LKEKFDFDEEARYSSVSRV-
CIDA-ZINGIBER-OFFICINALE-A0A8 FGVKSTFNEEIYTTKLER--GPQMKEREKVASRIVREIEGQEAHDFHQAEERGFN-FHDDL--DEESRYSSVCRES
CIDB-ZINGIBER-OFFICINALE-A0A8 FGVKSTFNEELYTTKLEK--GPQMKELEKVASRIAREIEGQEAHDFHQAEERGFN-FHDNLGLDEESRYSAVRREN
CIDA-AMBORELLA-TRICHOPODA-U5D FGVKSTFDEELYTTKLEK--GPQMRELEREATRIAREIQGEDTQDPHLAEERGIHHLLGDLELDEESRFSSVFRGM
CIDA-DIOSCOREA-ZINGIBERENSIS- FGVRSTFDEELYTTKLER--GPQTRELEREATRIAREIQEEETKDLHLAEERGIH-FHGDLDLDEELKFSAVQRDV
CIDB-DIOSCOREA-ZINGIBERENSIS- FGVKSTFDEELYTTKLER--GPQMRELEREATRIAREIQEEETKDLHLAEERGIH-FHGDLDLDEEIRFSAVRRDV
CIDA-ACORUS-GRAMINEUS-UPI0027 FGVKSTFDEELYTTKLEK--GPQKRELEREATRIAREIEREETNDVHLAEERGSH-FFDALDLDEESRFSSVLRDA
CIDB-ACORUS-GRAMINEUS-UPI0027 FGVRSTFDEELYTTKLEK--GPQTRELEREATRIAREIEGEETIDLHLAEERGSR-FFDDLELDEESRFSSVLRVA
CIDA-DAUCUS-CAROTA-SUBSP.-SAT FGVKSTFNEDLYTTKLDR--GPQTRELEREALRIAREIEVEDTQDLHLAEERGIQ-LPRDIELDEETRYSSVFRGV
CIDB-DAUCUS-CAROTA-SUBSP.-SAT FGVKSTFDEDLYTTKLDR--GPQMRELEIEALRIAREIEGEDTQDLHLAEERGLK-LQKDYEMDEEARFSSVLRGV
CIDA-CARYA-ILLINOINENSIS-UPI0 FGVKSTFDEELYTTKLER--GPKMEELEKKALRIAREIEGEETQDLHLAEERGIS-FPGDLEIDEETRFSSVYRGK
CIDB-CARYA-ILLINOINENSIS-UPI0 FGVRSTFDEELYTTKLER--GPKMEALEKKASRIAREIEGEDTQDLHSAEERGIS-FPEDIEVDEEARFSAVYRGK
CIDA-NICOTIANA-TABACUM-A0A1S3 FGVKSTFNEELYTTKLEK--GPLMSELEKEALRIAREIEGEDTRDLHLAEERGIQ-LHGNLEVDEETRFSAVVRE-
CIDB-NICOTIANA-TABACUM-A0A1S4 FGVKSTFNEELYTTKLEK--GPQMRELEKEASRIAREIEGEETRDLHLAEERGIQ-LHGNLEVDEETRFSAVVRG-
CIDA-OLEA-EUROPAEA-UPI0019C95 FGVKSTFDEELYTTKLDK--GPQMRELEREAKRIAREIESQETHDLHLAEERGIQ-LGGTLEIDEETRFSSVYRAV
CIDB-OLEA-EUROPAEA-UPI0019C64 FGVKSTFNEEFYTTKLEK--GPQMRDLEREAKRIAREIEGEDTHDLHLAEERGIQ-LTGNLEIDEETRFSSVYRGV
CIDB-PAPAVER-SOMNIFERUM-UPI00 FGVESTFDEEIYTTKLVK--GPQMKELEREALRIAKEIEAEDTQDLHLAEERGIH-FHDDCNIDEETRYSSVFRGH
CIDA-SAPONARIA-OFFICINALIS-A0 FGVKSTFNEELYTTKLEK--GPRTRELEEKAKRIAREIEGEETFDLHLAEERGVY-PHANFDIDEETKYSSVFRG-
CIDB-SAPONARIA-OFFICINALIS-A0 FGVHSTFNEELYTTKLEK--GPQMRKLEEEARRIAREIEGEETYDLHLAEERGAY-PHANFDLDEETKYSSVFRG-

LSMAD sequences used:

> CID4-Arabidopsis-thaliana-Q94AM9
FGVKSTFDEDLYTTRLERGPQTKQLEEHAQKIAREIEAETTRDIHVAEERGLQLNENFDFDEEARYSSVRPV

> CID3-Arabidopsis-thaliana-Q8L793
FGVTSTFDEELYTTKLERGPGTRELEEQALRIAREIVGENTRDIHVAEERGLQLSGKFDIDEETKYSSVCAT

> CIDa-Capsella-rubella-R0HKF4
FGVKSTFDEELYTTKLERGTQTKELEEQARRIANEIEGETTRDLHVAEERGLKLKEKFDFDEEARYSSVSRV

> CIDb-Capsella-rubella-R0GVL5
FGVTSTFDEELYTTKLERGPGTRELEEQALRIAREIVGENTRDLHVAEERGLQLSGKFDIDEETKYSSVCTL

> CIDa-Gossypium-hirsutum-UPI0005F6B8F5
FGVKSTFNEELYTTKLERGPQTRELEKEAMRIAREIEGEDTQDLHLAEERGVDLHDDFDIDEEMRYSSVYRG

> CIDb-Gossypium-hirsutum-A0A1U8PLN3
FGVKSTFNEELYTTKLERGPQTRELEKEAMRIAREIEGEETRDLHLAEERGLDLHDNFDIDEEMRYSSVYRG

> CIDa-Manihot-esculenta-A0A2C9W2D8
FGVKSTFDEEIYTTKLERGPQMRELEKEATRIAREIEGEDTQDLHLAEERGIHLDGNFDIDEETRYSSVYRG

> CIDb-Manihot-esculenta-A0A251JRN9
FGVKSTFDEELYTTKLQRGPQMRELEKKAMRMAREIEGEDTQDLHLAEERGIQLHEDFDIDEETRFSSVYRG

> CIDa-Nelumbo-nucifera-A0A1U8B059
FGVKSTFNEEFYTTKLERGPHMRELEREATRIAREIENEETHDLHLAEERGLNFHDDYDEEARFSSVLRG

> CIDb-Nelumbo-nucifera-A0A1U8A662
FGVKSTFDEELYTTKLERGPQMRELEREASRIAREIEGDETHDLHLAEERGLHLHDDFDIDEETRFSSVFRG

> CIDa-Saponaria-officinalis-A0AAW1GNL7
FGVKSTFNEELYTTKLEKGPRTRELEEKAKRIAREIEGEETFDLHLAEERGVYPHANFDIDEETKYSSVFRG

> CIDb-Saponaria-officinalis-A0AAW1ILT8
FGVHSTFNEELYTTKLEKGPQMRKLEEEARRIAREIEGEETYDLHLAEERGAYPHANFDLDEETKYSSVFRG

> CIDa-Rhododendron-williamsianum-A0A6A4L789
FGVKSTFDEELYTTKLDRGGPQMRDLEREASRLAREIEGEDTLDLHLAEERGIQLHGSLDIDEETRFSSVRRG

> CIDb-Rhododendron-williamsianum-A0A6A4MPX3
FGVKSTFDEELYTTKLERGPQMRDLEREALRIAREIEGEETHDIHLAEERGIHLHENFDIDEETRFSSVFRG

> CIDa-Nicotiana-tabacum-A0A1S3ZEU1
FGVKSTFNEELYTTKLEKGPLMSELEKEALRIAREIEGEDTRDLHLAEERGIQLHGNLEVDEETRFSAVVRE

> CIDb-Nicotiana-tabacum-A0A1S4C0M3
FGVKSTFNEELYTTKLEKGPQMRELEKEASRIAREIEGEETRDLHLAEERGIQLHGNLEVDEETRFSAVVRG

> CID4-Helianthus-annuus-A0A251U0N2
FGVTSTFNEELYTTKLDRGPLMKEREKEALRIAREIEGEDTEDLHLAEERGIHFQSGFDLDEETKYSSVFRG

> CID3-Helianthus-annuus-A0A251TRN9
FGVKSTFNEELYTTKLDRGPQMRELEKEALRLAREIEGEDTQDLHLAEERGIHFHDKFDLDEETKYSSVFRG

> CID4-Glycine-max-UPI001E84381D
FGVKSTFNEDFYTTKLEIGPQTRELEKQALRIAREIEGEETQDLHLAEERGLYHNFDIDEETRFSSVYRG

> CID3-Glycine-max-UPI000233BB97
FGVKSTFNEDLYTTKLEKGPQTRELERQALRIAREIEGEETQDLHLAEERGLHLHEDFDIDEETRFSSVYRG

> CIDa-Iris-pallida-A0AAX6EZ38
FGVKSTFDEELYTTKLERGPRMRELELKASRIAREIEGEDTNDLHLAEERGMQFHEDFDFDEEIRYSAVKRE

> CIDb-Iris-pallida-A0AAX6FP83
FGVKSTFDEELYTTKLERGPRTRELELKASRLAREIEGEDTNDLHLAEERGMQFHEDFDFDEEIRYSAVKRE

> CIDa-Araucaria-cunninghamii-UPI0005EA7401
FGVESTFDEGLYTTKLERGPQTRDLEMKASEIARQIERRKLGNSPSAEECFVNESETFDEESKCSSVHGSE

> CIDb-Araucaria-cunninghamii-UPI0005EA70B3
FGVESTFDEGLYTTKLERGPQTRDLEMKASEIARQIERRKLGNSPSAEECFVNESETFDEESKCSSVHGSE

> CIDa-Acorus-gramineus-UPI00275F7DBA
FGVKSTFDEELYTTKLEKGPQKRELEREATRIAREIEREETNDVHLAEERGSHFFDALDLDEESRFSSVLRDA

> CIDb-Acorus-gramineus-UPI00275DF534
FGVRSTFDEELYTTKLEKGPQTRELEREATRIAREIEGEETIDLHLAEERGSRFFDDLELDEESRFSSVLRVA

> CIDa-Tripterygium-wilfordii-UPI0017E19429

FGVKSTFDEELYTTKLERGPQMRELEKEALRIAREIEGEETHDLHLAEERGTSFHDKFDIDEETRFSSVYRGS

> CIDb-Tripterygium-wilfordii-UPI0018430C38

FGVKSTFDEELYTTKLERGPQMRELEKEALRIAREIEDEETHDLHLAEERGISFHDKFDIDEETRFSSVDRGR

> CIDa-Carya-illinoinensis-UPI001C36679D

FGVKSTFDEELYTTKLERGPKMEELEKKALRIAREIEGEETQDLHLAEERGISFPGDLEIDEETRFSSVYRGK

> CIDb-Carya-illinoinensis-UPI001C361ADA

FGVRSTFDEELYTTKLERGPKMEALEKKASRIAREIEGEDTQDLHSAEERGISFPEDIEVDEEARFSAVYRGK

> CIDa-Trapa-incisa-A0AAN7LJD0

FGVKSTFNEELYTTKLERGPQMKELEKEASRIAREIEGDVTQDLHLAEERGLQLHEFDVDEETRFSSVFRGR

> CIDb-Trapa-incisa-A0AAN7QSB2

FGVKSTFNEELYTTKLERGPQTRELEKEASRIAREIEGEVTQDLHLAEERGLQLHEDFDVDEETRFSSVFRGR

> CIDa-Olea-europaea-UPI0019C95325

FGVKSTFDEELYTTKLDKGPQMRELEREAKRIAREIESQETHDLHLAEERGIQLGGTLEIDEETRFSSVYRAV

> CIDb-Olea-europaea-UPI0019C64142

FGVKSTFNEEFYTTKLEKGPQMRDLEREAKRIAREIEGEDTHDLHLAEERGIQLTGNLEIDEETRFSSVYRGV

> CIDa-Daucus-carota-subsp.-sativus-UPI002B2D0B07

FGVKSTFNEDLYTTKLDRGPQTRELEREALRIAREIEVEDTQDLHLAEERGIQLPRDIELDEETRYSSVFRGV

> CIDb-Daucus-carota-subsp.-sativus-UPI0007B26D02

FGVKSTFDEDLYTTKLDRGPQMRELEIEALRIAREIEGEDTQDLHLAEERGLKLQKDYEMDEEARFSSVLRGV

> CIDa-Davidia-involucrata-A0A5B7AUX0

FGVKSTFDEELYTTKLERGPQMRELEREASRLAREIEGEETQDLHLAEERGIHLHENFDIDEETRFSSVFRGI

> CIDb-Davidia-involucrata-A0A5B7ASW3

FGVKSTFDEELYTTKLDRGPQMRELEKEASRLAREIEGEETQDLHLAEERGIHLHENFDIDEETRFSSVFRGF

> CIDa-Papaver-somniferum-UPI0011796043
FGVKSTFDEELYTTKLDRGPQMREREREASRIAREIEGEDTKDMHLAEERGRHFDFDVDEETRFSSVLRTV

> CIDb-Papaver-somniferum-UPI001178FABE
FGVESTFDEEIYTTKLVKGPQMKELEREALRIAKEIEAEDTQDLHLAEERGIHFHDDCNIDEETRYSSVFRGH

> CIDa-Zingiber-officinale-A0A8J5GUX2
FGVKSTFNEEIYTTKLERGPQMKEREKVASRIVREIEGQEAHDFHQAEERGFNFHDDLDEESRYSSVCRES

> CIDb-Zingiber-officinale-A0A8J5FJV2
FGVKSTFNEELYTTKLEKGPQMKELEKVASRIAREIEGQEAHDFHQAEERGFNFHDNLGLDEESRYSAVRREN

> CIDa-Dioscorea-zingiberensis-A0A9D5DE20
FGVRSTFDEELYTTKLERGPQTRELEREATRIAREIQEEETKDLHLAEERGIHFHGDLDLDEELKFSAVQRDV

> CIDb-Dioscorea-zingiberensis-A0A9D5DCF0
FGVKSTFDEELYTTKLERGPQMRELEREATRIAREIQEEETKDLHLAEERGIHFHGDLDLDEEIRFSAVRRDV

> CIDa-Amborella-trichopoda-U5D2G8
FGVKSTFDEELYTTKLEKGPQMRELEREATRIAREIQGEDTQDPHLAEERGIHHLLGDLELDEESRFSSVFRGM

> CIDx-Physcomitrium-patens-A0A2K1L9A4
FGVETTFDEELYTTKLIRGPQMHEREREAQRIAREIQGQQTRNMHLAEERGIHLAPELDALDEEARYSAVRRGY

> CIDx-Marchantia-polymorpha-UPI000D248F9E
FGVETTFNEELYTTKLERGPQMREREREAWRIAREIEGQTTRNPHVAEERGIRIAPELETMDEESKFSSVIRGA

> CIDx-Chara-braunii-A0A388K6U8
FGVESTFDENLYTTKLEKGSGPEARERereAARIAREIELKATRNPHLAEERGQVLETSIGEEELYSSVLRQP

> CIDx-Closterium-A0A9P3IK33
FGVETSFDEAIYTTPLVRPSRELQAHAERIAREIESAPTRNMHIAEERGFHLRNA…DEETKYSSVIRTA

> CIDx-Klebsormidium-nitens-A0A1Y1HQG5
FGVRTTFDENFYTTPLEKGNDALSRQREREAARIAREIEQSTSKNPHLAEERGQKAAPEIEALGEEERFSSVLRGE

> CIDa-Spirodela-intermedia-UPI0018A5D666
FGVKTTFNEELYTTKLERGPQMREREREALRIAREIEGEDTKDLHLAEERGIHFNEDFDFDEEARFSSVYRGI

> CIDb-Amborella-trichopoda-U5D208
-

> CIDa-Citrus-clementina-V4SRU4
FGVKTTFNEELYTTKLLRGPQTEELEKEAARIAREIAGEDTQDLHLAEERGSSIHENLGIDEETRFSSVYRG

> CIDb-Citrus-clementina-V4WG04
QKRISVRNENGYFHGDGPIKAEKEHEEQMLSLKNMRNAMEVEHGKRDRMDVTKIEEASVDSV

> CID3-Vitis-vinifera-D7T8B6

> CID4-Vitis-vinifera-A0A438K7K9
FGVNSTFDEEIYTTKLDRGPQTRELEKEALRLAREIEGEETHDLHLAEERGLHLHANFDIDEEARFSSVLRR

> CIDa-Sorghum-bicolor-A0A1Z5RNJ3
FGVKSTFNEEIYTTKLERGPHMRELEKHALKIAREIEGEDTKDTHLAEERGLFLGDDLDHDEEIKYSAVRRD

> CIDb-Sorghum-bicolor-C5YG86

Ataxin-2 family PAM2 alignment in plant species before and after gene duplication

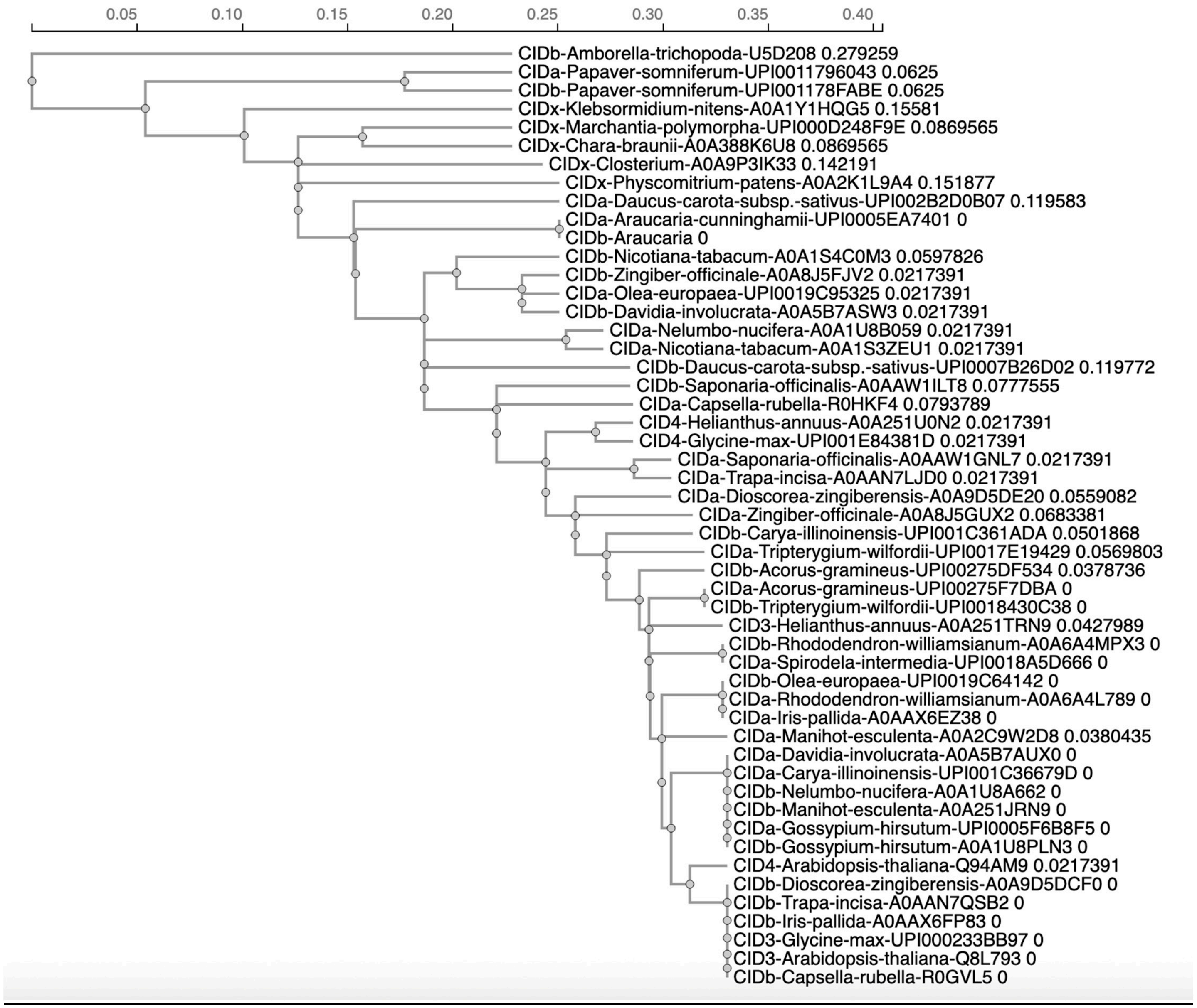
0.05
0.10
0.15
0.20
0.25
0.30
0.35
0.40
CIDb-Amborella-trichopoda-U5D208 0.279259
CIDa-Papaver-somniferum-UPI0011796043 0.0625
CIDb-Papaver-somniferum-UPI001178FABE 0.0625
CIDx-Klebsormidium-nitens-A0A1Y1HQG5 0.15581
CIDx-Marchantia-polymorpha-UPI000D248F9E 0.0869565
CIDx-Chara-braunii-A0A388K6U8 0.0869565
CIDx-Closterium-A0A9P3IK33 0.142191
CIDx-Physcomitrium-patens-A0A2K1L9A4 0.151877
CIDa-Daucus-carota-subsp.-sativus-UPI002B2D0B07 0.119583
CIDa-Araucaria-cunninghamii-UPI0005EA7401 0
CIDb-Araucaria 0
CIDb-Nicotiana-tabacum-A0A1S4C0M3 0.0597826
CIDb-Zingiber-officinale-A0A8J5FJV2 0.0217391
CIDa-Olea-europaea-UPI0019C95325 0.0217391
CIDb-Davidia-involucrata-A0A5B7ASW3 0.0217391
CIDa-Nelumbo-nucifera-A0A1U8B059 0.0217391
CIDa-Nicotiana-tabacum-A0A1S3ZEU1 0.0217391
CIDb-Daucus-carota-subsp.-sativus-UPI0007B26D02 0.119772
CIDb-Saponaria-officinalis-A0AAW1ILT8 0.0777555
CIDa-Capsella-rubella-R0HKF4 0.0793789
CID4-Helianthus-annuus-A0A251U0N2 0.0217391
CID4-Glycine-max-UPI001E84381D 0.0217391
CIDa-Saponaria-officinalis-A0AAW1GNL7 0.0217391
CIDa-Trapa-incisa-A0AAN7LJD0 0.0217391
CIDa-Dioscorea-zingiberensis-A0A9D5DE20 0.0559082
CIDa-Zingiber-officinale-A0A8J5GUX2 0.0683381
CIDb-Carya-illinoinensis-UPI001C361ADA 0.0501868
CIDa-Tripterygium-wilfordii-UPI0017E19429 0.0569803
CIDb-Acorus-gramineus-UPI00275DF534 0.0378736
CIDa-Acorus-gramineus-UPI00275F7DBA 0
CIDb-Tripterygium-wilfordii-UPI0018430C38 0
CID3-Helianthus-annuus-A0A251TRN9 0.0427989
CIDb-Rhododendron-williamsianum-A0A6A4MPX3 0
CIDa-Spirodela-intermedia-UPI0018A5D666 0
CIDb-Olea-europaea-UPI0019C64142 0
CIDa-Rhododendron-williamsianum-A0A6A4L789 0
CIDa-Iris-pallida-A0AAX6EZ38 0
CIDa-Manihot-esculenta-A0A2C9W2D8 0.0380435
CIDa-Davidia-involucrata-A0A5B7AUX0 0
CIDa-Carya-illinoinensis-UPI001C36679D 0
CIDb-Nelumbo-nucifera-A0A1U8A662 0
CIDb-Manihot-esculenta-A0A251JRN9 0
CIDa-Gossypium-hirsutum-UPI0005F6B8F5 0
CIDb-Gossypium-hirsutum-A0A1U8PLN3 0
CID4-Arabidopsis-thaliana-Q94AM9 0.0217391
CIDb-Dioscorea-zingiberensis-A0A9D5DCF0 0
CIDb-Trapa-incisa-A0AAN7QSB2 0
CIDb-Iris-pallida-A0AAX6FP83 0
CID3-Glycine-max-UPI000233BB97 0
CID3-Arabidopsis-thaliana-Q8L793 0
CIDb-Capsella-rubella-R0GVL5 0

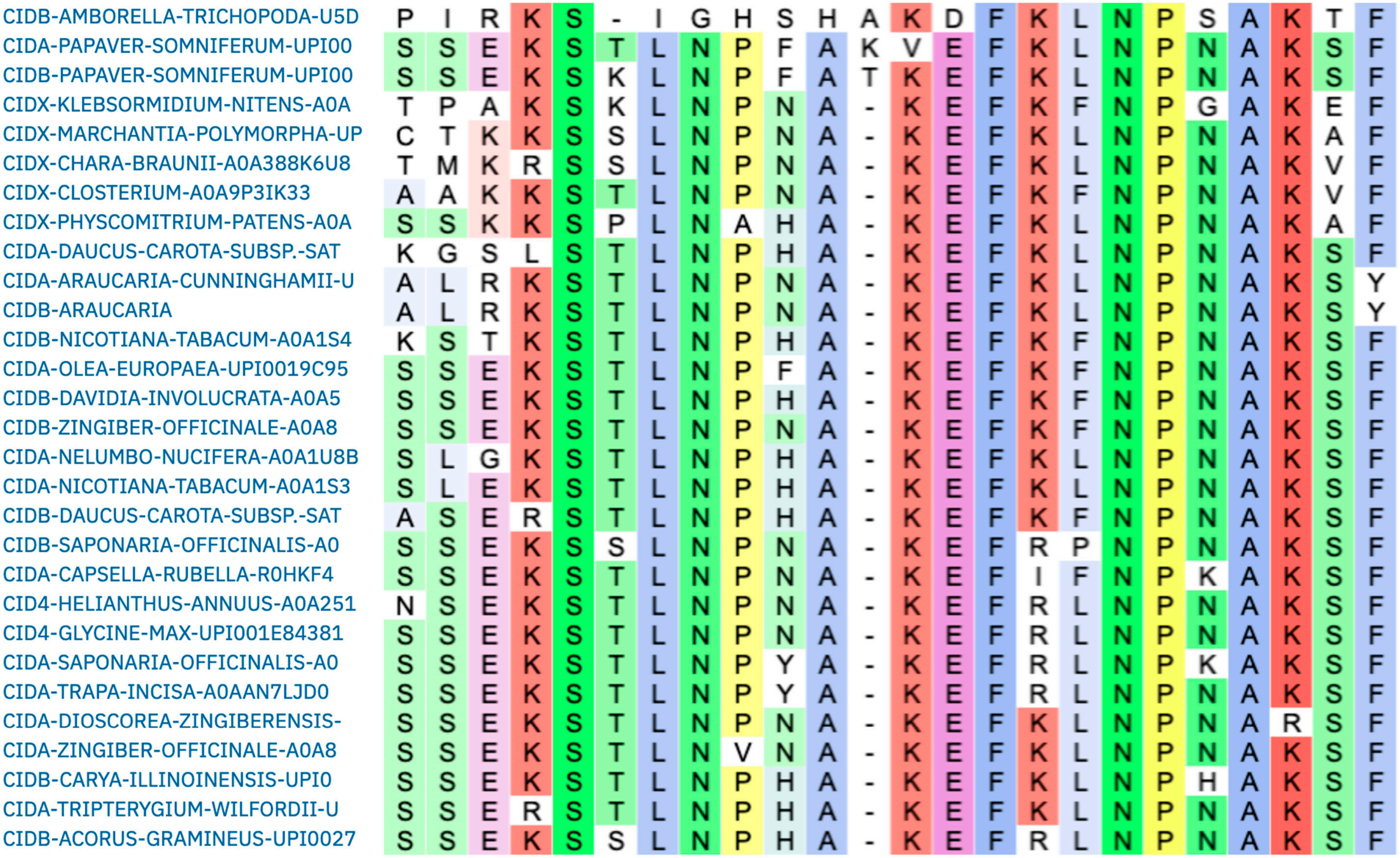
CIDB-AMBORELLA-TRICHOPODA-U5D P I R K S - I G H S H A K D F K L N P S A K T F
CIDA-PAPAVER-SOMNIFERUM-UPI00 S S E K S T L N P F A K V E F K L N P N A K S F
CIDB-PAPAVER-SOMNIFERUM-UPI00 S S E K S K L N P F A T K E F K L N P N A K S F
CIDX-KLEBSORMIDIUM-NITENS-A0A T P A K S K L N P N A - K E F K F N P G A K E F
CIDX-MARCHANTIA-POLYMORPHA-UP C T K K S S L N P N A - K E F K L N P N A K A F
CIDX-CHARA-BRAUNII-A0A388K6U8 T M K R S S L N P N A - K E F K L N P N A K V F
CIDX-CLOSTERIUM-A0A9P3IK33 A A K K S T L N P N A - K E F K F N P N A K V F
CIDX-PHYSCOMITRIUM-PATENS-A0A S S K K S P L N A H A - K E F K L N P N A K A F
CIDA-DAUCUS-CAROTA-SUBSP.-SAT K G S L S T L N P H A - K E F K L N P N A K S F
CIDA-ARAUCARIA-CUNNINGHAMII-U A L R K S T L N P N A - K E F K L N P N A K S Y
CIDB-ARAUCARIA A L R K S T L N P N A - K E F K L N P N A K S Y
CIDB-NICOTIANA-TABACUM-A0A1S4 K S T K S T L N P H A - K E F K F N P N A K S F
CIDA-OLEA-EUROPAEA-UPI0019C95 S S E K S T L N P F A - K E F K F N P N A K S F
CIDB-DAVIDIA-INVOLUCRATA-A0A5 S S E K S T L N P H A - K E F K F N P N A K S F
CIDB-ZINGIBER-OFFICINALE-A0A8 S S E K S T L N P N A - K E F K F N P N A K S F
CIDA-NELUMBO-NUCIFERA-A0A1U8B S L G K S T L N P H A - K E F K L N P N A K S F
CIDA-NICOTIANA-TABACUM-A0A1S3 S L E K S T L N P H A - K E F K L N P N A K S F
CIDB-DAUCUS-CAROTA-SUBSP.-SAT A S E R S T L N P H A - K E F K F N P N A K S F
CIDB-SAPONARIA-OFFICINALIS-A0 S S E K S S L N P N A - K E F R P N P N A K S F
CIDA-CAPSELLA-RUBELLA-R0HKF4 S S E K S T L N P N A - K E F I F N P K A K S F
CID4-HELIANTHUS-ANNUUS-A0A251 N S E K S T L N P N A - K E F R L N P N A K S F
CID4-GLYCINE-MAX-UPI001E84381 S S E K S T L N P N A - K E F R L N P N A K S F
CIDA-SAPONARIA-OFFICINALIS-A0 S S E K S T L N P Y A - K E F R L N P K A K S F
CIDA-TRAPA-INCISA-A0AAN7LJD0 S S E K S T L N P Y A - K E F R L N P N A K S F
CIDA-DIOSCOREA-ZINGIBERENSIS- S S E K S T L N P N A - K E F K L N P N A R S F
CIDA-ZINGIBER-OFFICINALE-A0A8 S S E K S T L N V N A - K E F K L N P N A K S F
CIDB-CARYA-ILLINOINENSIS-UPI0 S S E K S T L N P H A - K E F K L N P H A K S F
CIDA-TRIPTERYGIUM-WILFORDII-U S S E R S T L N P H A - K E F K L N P N A K S F
CIDB-ACORUS-GRAMINEUS-UPI0027 S S E K S S L N P H A - K E F R L N P N A K S F

PAM2 sequences used:

> CID4-Arabidopsis-thaliana-Q94AM9
SSEKSTLNPNAKEFKLNPKAKSF

> CID3-Arabidopsis-thaliana-Q8L793
SSEKSTLNPNAKEFKLNPNAKSF

> CIDa-Capsella-rubella-R0HKF4
SSEKSTLNPNAKEFIFNPKAKSF

> CIDb-Capsella-rubella-R0GVL5
SSEKSTLNPNAKEFKLNPNAKSF

> CIDa-Gossypium-hirsutum-UPI0005F6B8F5
SSEKSTLNPHAKEFKLNPNAKSF

> CIDb-Gossypium-hirsutum-A0A1U8PLN3
SSEKSTLNPHAKEFKLNPNAKSF

> CIDa-Manihot-esculenta-A0A2C9W2D8
SSEKLTLNPHAKEFKLNPNAKSF

> CIDb-Manihot-esculenta-A0A251JRN9
SSEKSTLNPHAKEFKLNPNAKSF

> CIDa-Nelumbo-nucifera-A0A1U8B059
SLGKSTLNPHAKEFKLNPNAKSF

> CIDb-Nelumbo-nucifera-A0A1U8A662
SSEKSTLNPHAKEFKLNPNAKSF

> CIDa-Saponaria-officinalis-A0AAW1GNL7
SSEKSTLNPYAKEFRLNPKAKSF

> CIDb-Saponaria-officinalis-A0AAW1ILT8
SSEKSSLNPNAKEFRPNPNAKSF

> CIDa-Rhododendron-williamsianum-A0A6A4L789
SSEKSTLNPYAKEFKLNPNAKSF

> CIDb-Rhododendron-williamsianum-A0A6A4MPX3
TSEKSTLNPHAKEFKLNPNAKSF

> CIDa-Nicotiana-tabacum-A0A1S3ZEU1
SLEKSTLNPHAKEFKLNPNAKSF

> CIDb-Nicotiana-tabacum-A0A1S4C0M3
KSTKSTLNPHAKEFKFNPNAKSF

> CID4-Helianthus-annuus-A0A251U0N2
NSEKSTLNPNAKEFRLNPNAKSF

> CID3-Helianthus-annuus-A0A251TRN9
TSEKSTLNPHAKEFRLNPNAKSF

> CID4-Glycine-max-UPI001E84381D
SSEKSTLNPNAKEFRLNPNAKSF

> CID3-Glycine-max-UPI000233BB97
SSEKSTLNPNAKEFKLNPNAKSF

> CIDa-Iris-pallida-A0AAX6EZ38
SSEKSTLNPYAKEFKLNPNAKSF

> CIDb-Iris-pallida-A0AAX6FP83
SSEKSTLNPNAKEFKLNPNAKSF

> CIDa-Araucaria-cunninghamii-UPI0005EA7401
ALRKSTLNPNAKEFKLNPNAKSY

> CIDb-Araucaria cunninghamii-UPI0005EA70B3
ALRKSTLNPNAKEFKLNPNAKSY

> CIDa-Acorus-gramineus-UPI00275F7DBA
SSEKSTLNPHAKEFRLNPNAKSF

> CIDb-Acorus-gramineus-UPI00275DF534
SSEKSSLNPHAKEFRLNPNAKSF

> CIDa-Tripterygium-wilfordii-UPI0017E19429
SSERSTLNPHAKEFKLNPNAKSF

> CIDb-Tripterygium-wilfordii-UPI0018430C38
SSEKSTLNPHAKEFRLNPNAKSF

> CIDa-Carya-illinoinensis-UPI001C36679D
SSEKSTLNPHAKEFKLNPNAKSF

> CIDb-Carya-illinoinensis-UPI001C361ADA
SSEKSTLNPHAKEFKLNPHAKSF

> CIDa-Trapa-incisa-A0AAN7LJD0
SSEKSTLNPYAKEFRLNPNAKSF

> CIDb-Trapa-incisa-A0AAN7QSB2
SSEKSTLNPNAKEFKLNPNAKSF

> CIDa-Olea-europaea-UPI0019C95325
SSEKSTLNPFAKEFKFNPNAKSF

> CIDb-Olea-europaea-UPI0019C64142
SSEKSTLNPYAKEFKLNPNAKSF

> CIDa-Daucus-carota-subsp.-sativus-UPI002B2D0B07
KGSLSTLNPHAKEFKLNPNAKSF

> CIDb-Daucus-carota-subsp.-sativus-UPI0007B26D02
ASERSTLNPHAKEFKFNPNAKSF

> CIDa-Davidia-involucrata-A0A5B7AUX0
SSEKSTLNPHAKEFKLNPNAKSF

> CIDb-Davidia-involucrata-A0A5B7ASW3
SSEKSTLNPHAKEFKFNPNAKSF

> CIDa-Papaver-somniferum-UPI0011796043
SSEKSTLNPFAKVEFKLNPNAKSF

> CIDb-Papaver-somniferum-UPI001178FABE
SSEKSKLNPFA…TKEFKLNPNAKSF

> CIDa-Zingiber-officinale-A0A8J5GUX2
SSEKSTLNVNAKEFKLNPNAKSF

> CIDb-Zingiber-officinale-A0A8J5FJV2
SSEKSTLNPNAKEFKFNPNAKSF

> CIDa-Dioscorea-zingiberensis-A0A9D5DE20
SSEKSTLNPNAKEFKLNPNARSF

> CIDb-Dioscorea-zingiberensis-A0A9D5DCF0
SSEKSTLNPNAKEFKLNPNAKSF

> CIDb-Amborella-trichopoda-U5D208
PIRKSIGHSHAKDFKLNPSAKTF

> CIDx-Physcomitrium-patens-A0A2K1L9A4
SSKKSPLNAHAKEFKLNPNAKAF

> CIDx-Marchantia-polymorpha-UPI000D248F9E
CTKKSSLNPNAKEFKLNPNAKAF

> CIDx-Chara-braunii-A0A388K6U8
TMKRSSLNPNAKEFKLNPNAKVF

> CIDx-Closterium-A0A9P3IK33
AAKKSTLNPNAKEFKFNPNAKVF

> CIDx-Klebsormidium-nitens-A0A1Y1HQG5
TPAKSKLNPNAKEFKFNPGAKEF

> CIDa-Spirodela-intermedia-UPI0018A5D666
TSEKSTLNPHAKEFKLNPNAKSF

> CIDa-Amborella-trichopoda-U5D2G8
-

> CIDa-Citrus-clementina-V4SRU4
SSEKSTLNPYAKEFKLNPNAKSF

> CIDb-Citrus-clementina-V4WG04
KSTKEFKLNPGAKIFSPSSVNPVSATSP

> CID3-Vitis-vinifera-D7T8B6
LPRSSISNKSAKESKLNPGAKVF

> CID4-Vitis-vinifera-D7TZU9
SSEKSTLNPHAKEFKLNPNAKSF

> CIDa-Sorghum-bicolor-A0A1Z5RNJ3
TSEKSTLNPNAKEFKLNPNAKSF

> CIDb-Sorghum-bicolor-C5YG86
TKIVTSSKTAAKEFKLNPCAKVF

Arabidopsis thaliana

CLUSTAL O(1.2.4) multiple sequence alignment

```
CID3_ARATH      -------MKPVLHSGSSSNGFSHRRFEKEAWMNNAQPSVDNTENGWDAESVDTPSQKLLV 53
CID4_ARATH      MLEQSKAAMSIPQSKSSSNGFPLKRGETEEVLHKTNTS--NTVFNGEAGSLKRLSLDRLV 58
                          : :* ******  :* *.*  ::::: *  **  . :* *:.  * . **

CID3_ARATH      YFTTCNIGHQVEVHLKNGSVYSGIFHAANVEKDFGIILKMACLIRDSRG----TKSRTVS 109
CID4_ARATH      YFTTCKIGHHVEVHLRNGSVYTGIFHAANVEKDFGIILKMACLIKDGTLRGHKSRSEFVR 118
                *****:***:*****:*****:*********************:*.      ::*. *

CID3_ARATH      KPSSKLLKIPADELVQVIAKDLPLSSDSVSDSVQCEKPLELLTDSLISQFYNVDLERELK 169
CID4_ARATH      KPPSKTFIIPADELVQVIAKDLSVSSNNMSNAVQGEKPSELLTDSSISQSYHVDRERQLQ 178
                ** **  : ************** :**:.:*::** *** ****** *** *:** **:*:

CID3_ARATH      PWVPDEDVPDCSDLENVFDDPWKRGWNQFEVNKTLFGVTSTFDEELYTTKLERGPGTREL 229
CID4_ARATH      RWVPDETIPHGADLENVFDNPWNRKWNQFEVNKSLFGVKSTFDEDLYTTRLERGPQTKQL 238
                 ***** :*. :*******:**:* ********:****.*****:****:***** *::*

CID3_ARATH      EEQALRIAREIVGENTRDIHVAEERGLQLSGKFDIDEETKYSSVCATNRFDDTCYEDDEE 289
CID4_ARATH      EEHAQKIAREIEAETTRDIHVAEERGLQLNENFDFDEEARYSSVRPVTGFGDSGFDL--- 295
                **:* :*****  .*.*************. :**:***::****   .. *.*: ::

CID3_ARATH      EEEDILLDCCNNLTFGDSSASDGKEPASTGKFYEDS--WGDSLHLRSNKMVDQSWSNSNK 347
CID4_ARATH      -EDNALLDTCNDLTFGGSSTSDGQKPASSGKGCEELRVSGDSQSSRKNKNVDQSCST--- 351
                 *:: *** **:****.**:***::***:**  *:     ***    *.** **** *.

CID3_ARATH      HTRQLMSELPSKDFPVAGNNIRNESQLGEQRKSKFLGASLFKKPSEESVSGFEDAPPPVK 407
CID4_ARATH      ------SKQQSKDFPAAGSNISE-SQLDEQRRKNNEEVSHNNRSAEESTSGHGDIKEGAK 404
                      *:   *****.**.** : ***.***:.:   .*  :: :***.**. *    .*

CID3_ARATH      PS---------FI--DGRLGLLSDRAKSENSSGWPGSSISRNSENSAASSASNLPILSPS 456
CID4_ARATH      SGGGASSVSKAVTEREREASQVSSKTKSESS---FGQSASRSSESRPGPSTSSRPGLSPS 461
                 .         .   :  .  . :*.::***.*    *.* **.**.  . *:*. * ****

CID3_ARATH      SS--GSLSSEKSTLNPNAKEFKLNPNAKSFKPSPSATRPQSPQSPVFDGSFYYPPV---- 510
CID4_ARATH      SSIGSMASSEKSTLNPNAKEFKLNPKAKSFKPLQSAAA--PPQSPIADASFYYPGPSHVP 519
                **   .  *****************:******  **:     ****: *.*****
```

```
CID3_ARATH      -PPMPGLHIRYGTGAAFPGQQHPMMYNNTTQLSPNQTYYSPNSPQYPQPMMVT------- 562
CID4_ARATH      VQQMPGMPPVNYGLPPYPGNQPQMMYHPQAYYH------PNGQPQYPQQQMIPGQQQQQM 573
                   ***:         :**:*  ***:  :            ..*****  *:

CID3_ARATH      -----QQRPILFMPPTPYQPEMPYKGRDSY        587
CID4_ARATH      IPGQQHPRPVYYMHPPPYPQDMPYHNKGRE        603
                     : **: :* * **  :***:.:.
```

Chara braunii

DRFL

FMTMCLIGQTVEVQVKNGSVYQGIFHTANTERDFGVVLKMARLIKGASVKGGKVEAIKDS

ARKAPIKMLIISSQDFVQIIAK
