# Supplementary material for: Bioinformatic Analyses of the Ataxin-2 Family Since Algae Emphasize Its Small Isoforms, Large Chimerisms, and the Importance of Human Exon 1B as Target of Therapies to Prevent Neurodegeneration
Source: Int J Mol Sci. 2026 Feb 3;27(3):1499. doi: 10.3390/ijms27031499 (PMC12898128; doi:10.3390/ijms27031499)
Supplement: Supplementary file 1 [file ijms-27-01499-s001.zip › AuburgerSen_SupplMaterialS3-HomoSapiensATXN2Lgenomic.pdf]

Homo sapiens chromosome 16, GRCh38.p14 Primary Assembly

NCBI Reference Sequence: NC_000016.10

GenBank Graphics

```
>NC_000016.10:28821575-28838654 Homo sapiens chromosome 16, GRCh38.p14 Primary Assembly
AATTTTTGCATTTTTAGTAGAGACAGGGTTTCACCATGTTGGCCAGGCTGATCTCAAACTCCTGACCTCA
AACCTCCCAAAGTACTGGGATTACAGACCATGCCCAGCCTCTTTTCACTTTCTTAAATAAGCTCCCCACT
GCTTTGAGGATAATGTCCGTGTCTCTTACAAGACTTGGGAGCCTTTAAAACCTGATCCCAGATTAGCTAC
CTCTTCCCCCCTTTAATTTTATGCTTAGGACAAGCTGAGTTTTTCTTAGTTTGTCATATCCTATTTCCCT
GCCTAGAAACCCATCTCCCCCTGCCCTCTTATCAGTTTTTTTTTATAATTTAAATTTTTTTTTTTTTGTA
GAGACAAAGTCTCACTTTGTTGCCTAGGCTGGTCTCAAACTCCTGGCCTTAAACGATCCTTCTGCCTCGG
ACTCCCAAAATGTTAGGATTACAGGTATGAGCCACTGCGCCCGGCCCTCCTGTCCCTTATCTGGGTCTCC
CAAAACTGGATTAAGATTAGGTGTCACTCCCATTTGCTCTTGTAGTCCTCTGTGCTTCACCTACTAGATC
TCGTCAAATTCTCTGAATTCACCACTAGCATGAACTCCTCCAGAGCAGGAAGCAGTTTAATTATCCTTGT
GTCCAAAACAACTAACTGAATACTCCAATGCCCAACTCATTTTGAATGAATGAAGCTGCCTTCATTGCAG
GGTCGGGTGGAGGATTTTGAACTCGGAACTTTGGCCTTCAGTCTTTGTTATTACAGTTTAAAGATTCATT
CTCGGGCATGGAGGAACAATTTAGGTCAAAATCACAAAAATCTGAAATAATTTAGGACGAGGCGTAAATT
TATCTGCAAAGATCAACACGCTCAAACGAATCACTAATGCTCATCAGTCTTTTACTTAAAGCAAACAAGG
TGGAGGGTAGCTCCTTGCGAAACCTACCACCTGCCTAAAAGCGCAGTGGCTCGGGTGGTCCAGTCCCGGA
GACTTTCTTGGCACTTAAGGCTCTGCCCACGGGCCGCCAAGTGGCGGAAGAGCCTCACGCACGACGTGCC
GCCTCACACTCCCGCGGAGGCATCGCGACGCAGCGACGGCGTGGGCGGAGAGGCCGGCCCTTCCTCTGGC
GCCTTCGGCTCCTCCCCCTGCGCCTCACACGCTCATTGTGAAGCGACAGCGGCCGCCGGCTTCTAAGGCG
GTTTGAGACCTTAAGACCCTAAATCCTTGGCGCGCGTGAGCGCCCGCGCCGCTCCTTACCCACTCCCAGG
GCTAGGGGTGCGAGGTTCACCGACTTGATTCTCTCCCACTTTCCCGCCGGGAGCTAACCGCGCCGGTGTG
CGCGGCACCTCGCGCCACTGCTCGCTCCTGGCCCCCCCCCCCCCCCCCCCCCCCCCGGGAGCTCCCACCCG
CGCGCTCCTCCCTCTCCGCTCCCCGGCGACGCGCACGCGCGCCAGCCCGGCTCGCGCCCTCTCGCTTTCC
TCCAGCCGCGAGACCCCCTCCCCTTCCGCCTCGCGGCGCTTCCTCGCGCCGCGGTCTTCTCTCTCCACCC
CCGACACCGCGGGGCTCCCCCCGCCCGCCCACGGCGGGCCCCGGCTGCCCGATCCCCCTCGCTTCCCGCG
CTCTCCAGCGGGGCCCCAGCCCCGGCCCCCTCTCTCCCTCCCTTCTCTCTAATTCCCCTTCCGGACGCTG
CCATCATGTTGAAGCCTCAGCCGCTACAACAGCCCTCCCAGCCCCAGCAGCCGCCCCCCACGCAACAGGC
CGTGGCCCGTCGGCCCCCCGGGGGCACCAGCCCTCCCAACGGCGGCCTCCCGGGGCCGCTGGCCACCTCT
GCGGCTCCTCCCGGGCCTCCAGCGGCCGCCTCCCCCTGCCTGGGGCCTGTGGCCGCTGCCGGGAGCGGGC
TCCGCCGGGGAGCCGAAGGCATCTTGGCGCCGCAGCCGCCGCCGCCGCAGCAACACCAGGAGAGGCCGGG
GGCAGCCGCCATCGGCAGCGCCAGGTGAGAAGGGTGGGCTCCGGGCGAGGGAGCCGCGGCCACCCAGAGG
```

Exon for 5'UTR

Exon 1 encoding
MLKPQPLQQPSQPQQPPPTQQAVARRP
PGGTSPPNGGLPGPLATSAAPPGPPAAA
SPCLGPVAAAGSGLRRGAEGILAPQPPP
PQQHQERPGAAAIGSAR

CTGTGGCTCGGTTCCGGTGGGGCGGACCCCGACCCGGCACCGTCAGGGGCACCGGCTGGGTGGGGAGTCC
CCGTGAAGCTGGGGGAGGGCCCCCTCAGGTCCGAACAGGTCGGACGGAAAGGGTCCCCGGGCGGCCACCG
GAGCACTGAGGGGCCGCGCTCGGCTCTCGGGGCCTAGTCAGGGCTCGCAGCCCCGGCCTTCAGGGGAGGC
GGGGCGCATCCCGCCGGCGCCGTCGGAACGGGGAGTTGGGGGGGGCAAGGAGCTGATCGGGGTCCCCGG
TTCCATTACCACGAAGGGTCTCGCGGTCCCCGGCTTCAGCCAATGGGGAGTCTGCTGCGGGAAGGGGTCC
CCGGCCGTAGCCAATGGAGGGGGGCGCGCGGCCCACAGTTTGGCCAATGGGGAGGGAGGGACTTGGGAGC
GGGTTGCTGCCTCCCCCTTCCCGGTCTGGCCAGTAGGAGGGGAGCGAGGTGGGCGGGGGACGGAAGGAG
GGATGACTGGGAGGACTGCGGGCCGGGAGCCTCTGGCGCGCGCGCCCCCTTCCCGTACGTGCGCGTGGAT
GCTCGGTCTCATGACCCGGGCATTCGCGCGCCCCGGGAACACCTAGGGCAGGGACTAGTTCGTGGAGGGG
CTCGTCTGGTGGCAGTGCATGAGTAGTGGAGGCCCTGCTCAGTTTTTCCTGTGCGAGTGTGTGTGTGTTA
ATGGAATTAAGAGTGGCAGCACACGCAGGCGCAGTGGGCTCTGATCGCTGGAGGTGGGGGTTCGGAAAGT
CCCGTGGGTTTTAGTGCGCAGGCGCAGACCGGGCGAGGCCTCCCGGTGGATGGCTTTTGCGGCTGCGCTG
TCCCCCAGCCCCGCCAGCGGCCCCCTCTTCGCCCTCAACCGCCGGTTACATCAGCCAGCGACGAGCAGGG
TTACCTGGCGATTGGTGATCCCCGCAGAGTGGGTAACGGGCTGAATGAGTCATGAGGTCGAGGGGATGGG
GTGTGGAGGGGATACCCCTCCTCCCACAGTTTTGAGGCGTCAGGCTGCTGAAAATGATTGTCTTTTCTGT
TTGGGAGGTAATGTACCTGAGCTAGGTAGTTCCAAAGCTGCACTCCTGGAGCTTTTGCCCTCACAGCTGG
CGTGGCTTTTTGGTTAATACTGTAGCATTGATCTGTTGGTAGCCTGCAGCCCTAGCCCCTTTCTCGTTGG
TTCTTAGAGCAATTGACAACCATTGTTGATTGAGATCTTTTTGCTGGCATTTGGAGGAAATGTGTTTGCT
GGCTTATTAATGTCTATTTTTTTCTTCCTCTACAAGGTGACAGTTTGAAAATCTTATTTATTGGCCGGGC
GCAGTGGCTCACGCCTGTAATGCCAGCACTTTAGGAGGCAAAGGTGGGAGGATCCCTTGAGCCCAAGGAG
ATCGATACCAGCCTGGGCAACACAGATGGGAAATCCCATCTCTACTAAAAATTAAAAAAAAAAAAAGCCA
GGAGTGGTGGTTCGTACCTATAGTCTCAGCCACTCGGGAGGCTGAGGCAGGAAGATCACTTGAGCCTTGG
AGGTTGAGGCTACAGTGAACTGAGATCACGCCACTGCACCCCAGCCTGGGCAACAGAGGGAGACCCTGTC
TCAAAAAAAATAGAAAAATCTTACTGATTAACAATTTTGTAGTCTAAATCAGCATCATCAGGTGGTATAT
ACTTCTAGGATTTAACATTAATTTCATAGTGGTCTAAGAGGGCTTGAACAGACTTAAGTAATTTCTTGTT
TTCTTTTATAGGGGACAGAGCACAGGAAAGGACCCCCACAGTCACCTGTGAGTGTCTTCTCCCACCCTG
TTTAAGATACATAGACCTAAAAGATGCATAATGTGGGAATATAGGGCACATTAGGTCTAGATAGAGTCTA
ATGTACTCAGGAGGGCTGTGGGCCCGGCACACACAAGGCAGAATGAGTAGAGTGCGGCGGGCATTTAGAA
AAGAAAATGAGGTGTTGACTGATTACTCCTTTAATTCTCCCTCTTATGTTAACTGACAGGTGTTTGAAGG
CGTCTACAACAATTCCAGAATGCTGCATTTCCTTACAGCTGTTGTGGTAAGTTGGTACTTAACCCCCGGG
TTGTTTAAGGAACGTAATGCATCTACTTTCTGGAGACACTCTTCTTATTTTTCCCACTCTGCCAGGGCTC
CACTTGTGATGTAAAGGTGAAAAATGGTACCACTTATGAGGGTATCTTCAAGACGCTAAGCTCAAAGGTC
AGTGTACTCAAATTTAATTATTTTTGGAGTTGCAGAGTAGGAGGAGAATGAAATAGGCTCATTGAAGGTG
TCAATTGGGTGATGTCAGAGTATGCCTTTAGTTGTTTCTGTAGGCCTGTGCTGAATAGTGCTGCAGGGAA
AAAAGACAAATTTGAGGGCTGGGTACTTTAATGTTAAAATATTTAAGTTTAAATTTTTGTGAGACTTTTG
CTAAGTCCTGTGGCTGATGTTGAGAAAACAATGCACTTGGTTCCAAGCATGTTGAGGATGTAGTGTTGTG
AAAAGTTTGGGAAGGGTAAGAGAAATCCAGTTCTATTTAAGAGAAATCCAGTTCTATTTTTGCCTTCACT
TTTCTTGAAACTGACCCATGGGTGTGGGGAATGGGGTGTTTGTAGTTTGAACTAGCCGTGGATGCTGTGC
ACCGGAAAGCATCTGAGCCAGCAGGTGGCCCTCGTCGGGAGGACATTGTGGACACCATGGTGTTTAAGCC

Exon 2, encoding
GQSTGKGPPQSP

Exon 3 with LSM domain first part, encoding
VFEGVYNNS**RMLHFLTAVV**

Exon 4 with LSM domain second part, encoding
GSTCDVKVKNGTTYEGIFKTLSSK

Exon 5 with LSM domain third part, encoding
FELAVDAVHRKASEPAGGPRREDIVDTMV
FKPSDVMLVHFRNVDFNYATK

AAGTGATGTCATGCTTGTTCACTTCCGAAATGTTGACTTCAACTATGCTACTAAAGGTATTGTCCTAGGC
TGTTACCTCAGACCTGCTCTGTGTGCATAGAGGACAGAGGGTAGTTTGTGTGCAGGTGGAACATGGTGAT
GTGTTTGGTTTGTTTTTTTGTTTTTGTTTGTTTGTTTTGCTTTAATGCCTTTTTTTTCCTGAGCGAAGTG
GGTGGATTTTTCTTCTTAAAAATATGTCTTGATGTCTAATATATAATGCGATGAATTCCTGTCTGTGTTG
TGGTTCTTCATATTTTCTTTGCTTGGTTTTTAACTTCTTTTTTCTTTGGAATCATAGTACTGATAGACTT
TTTATACTCTTCTTCTCTTGCCTCCCCACCCCCTGGCCATCCTAACACACGGGCACACGTACTCTGGACT
TCTTAAACTTTGTTCCTGAACTACTTACGGAGAGTGGGGTTGGGGGATATTGGAAAGAAGTCTGTGAAAT
ATAGCCTGACTCCTGATCTTCACCTCTGCCCCCACAGACAAGTTCACCGATTCAGCCATTGCCATGAACT
CGAAAGTGAATGGGGAACACAAAGAGAAGGTGCTTCAGCGCTGGGAGGGGGGTGACAGCAACAGCGACGA
CTATGACCTCGAGTCTGACATGGTATAGCCTCCTTCCCTGAGAACTTGGGAGCTGGACAGACAGAATGGG
TTGTTGACAGTGAGGTTCATTTGAGTGGGGAGGGAAATATTTACTGTTGCCCATTGATGGTAGTCAGATA
ACAAATAGAAAGGTATAGAAAATAGCAAAGTAAAAATATCTATTCTTAGCTGGGTGCAGTGGCTCATGTT
TGTAATCCCAGCACTTTGGGAGGCCAAGGCCGGTGGGTCACTTGAGGCAAGGATTTCGAGACCAGTCTGG
GCAACATGGTGAAACCCTGTCTCAACAAGATATATAAAAGCTATCAAGGTTTGGTGGCATGTACCTGTAG
TCCCAGCTACTTGGGAGGCTGAAGTGGAAGGATCACTTGAGCCCAGGGAGGTCGAGGCTACAGTGAGCTG
TGATCATACCACTGCACTCCAGCCTGGGTGGACCGTGTCACACGCAACAAAACCAAAACATCAACTTACT
CTCTTACTATCTATAATTAACCACTCCTAGCATTTGCATCACGTTATTTTTCAACATAACAAAAAGTATA
GATTAATGTGTAGGCCCATTTCTCTTCCCAAAATCAGCTACTCTGAGGCATTTAAAATCTCATGAGGCTG
AGCGCAGTGGCTCATGCCTGTAGTCCCAGCTACTTGGGAGGCCAAGGCAGGAGCTCCGATCACTTGAGCC
CAGGAGTTTGAGGCTGCTGTAAGCTGAGATTGTGCCACTGGACTCCAGTCTGAGCGACAGTGAGGATGTC
TCAAAAAAATAAATAAAAACCGCTCATGAATAGAGCTGAGCGTGGTGGTCCCACACCTGTAGTCCCAGCT
ACTCAGGAGGCTGAGGTAGGAGAATCATTTGAAGCTTTGGTGCACTATGACTCAACTTGTGAATGGCCAC
TGTGCTCCAGCCTGGGCATCATAGCGAGACTATCTGTTGAGAAACAAATGTCTTGTGAATGAATATATTA
TTGTTTTGTTATGTTTAGTTTGTACAAGAATTTGCTTTTCTTGCCTTTTCGAACTTCTCATGGTTGTACG
TAGATCTAGTTCTTTGCTTCTATTGTTGTGTGCTGTTTTCAAATATACACACACACGTGTACACAGCAAA
TTGTATTTGTCCTTTTCCTTAATCATCTGCATTTGGTTGCCATTATTTTGCTACTAAAAATAGTACCTTG
CTGGACTTTTAGAACATATATTCCCCACGCACATGTGCCAGAATTTTTCTGGGATGTCTTGGAAATAGAA
TTGTTAGGTCTTATGACTTTTCAGGTTTACTAGTTATTGATAATTTCTGCTGCAGTTGTTAGTAATCATT
AACAGTTGCAGGCCGGGCGCGGTGGCTCACACCAGTAATCCCAGCACTTTGGGAGGCCAAGGCGGGCAGA
TCACCTGAGGTTGGGAGTTTGGAACTAGCCTGACCAACGTGGAGAAGCCCCATCTCTACTAAAAATACAA
AATTAGCTGGGCGTGGTGGCACATGCCCGTAATCCCAGCTACTCGTGAGGTTGAGGCAGGAGAATCGCTT
GAACCCAAGGAGACGGAGGTTGCGGTGAGCCAAGATCGCACCATTGCACTCCAGCCTGGGCAACAAGAGT
GAAACTCTTTGTCTCCAAAAAAAAAAAAAAAAATTGCAGTTATTAATTACAATTATTAGTTGATTACTTTT
GCCAGCCACGTTCCAATTTTTTTTCCACTCTTGTTTTGTCGTTTATTTTTGAGACGAGCTTGCTCTGTTG
CCCGGGTTTTTGTTGTAGTTGTTTGTTTTTGTTTTTGTTTTTGTTTTTTTTGGAGACAGGGTCTCACTCT
GTCACTCAGGCTGGAGGCTGGAGTGCAGTGGCTCGATCTCAGCTCACTGCAACCTCTGCCTCCCAGGTTC
AAGCGATTCTTCTGCCTCAGCCAGGACTGCAGGCATGCGCCACCACGGATGGCTAATTTTTTATTTTTAG
TAGAGACGGGGTTTCACCATGTTGGTCAGGTTGATCTCCTGACCTCAGGTGATCCACCCACCTCCCAAAG
TGCTGGGATTACAGGCATGAGCCACCACGCCCAGCTAGTCGCCCAGGCCAGAGCGCAGTGGTGCCATAAT

Exon 6, encoding
DKFTDSAIAMNSKVNGEHKEKVLQRW
EGGDSNSDDYDLESDM

```
AATTCACTGCAGCCTCAAACTCCTGGGCTCAAGTGATCCTCTCTCCTCAATCTCCTGAGTAGCGAGTAGC
TAGGACCCCAGGCATGCACCACCACACCCGGCTGTTTCATTATTTTTTTCTAGAGATGAGCTCTTGCTGT
GTTGCCCAGTCTGGCCTTAAACTCCTGGCCTCAGGAGAGCCTCCTGCCTCAGCTTCCCAAAGTACTGGGA
TTACAGGTTTCAGCCACTGTGCCCAGCCAATTTGTTCACATCTTGGAGTTTTTAACCTGATGGAAGGGAA
GTGACTTGTCGTTGCTTTTCCTGCTGGGTTTTAAAACCACCTTCCTCCCTCCCCAGTCCAATGGATGGGA
CCCCAATGAAATGTTCAAGTTCAATGAGGAGAACTACGGTGTGAAGACTACCTATGATAGCAGTCTTTCT
TCTTATACGTGAGTATCTTGGTGCTCTCCAGGTGATGTGTTGGTGATATGGGGTCACTAAGTGAAGACAG
GTTTCCAGGTAGAACATAGTTTTTGCTCATTTTTCTCTGGGTGTCCAGGGTCGCCATCCCTACTCCTACT
CTGCCTTGTGGAATTCTTCCCTCAAAGGTTTTAAGCGTCTTAAGTGCTTCTCACATTCCCAGATAAGCCT
TGGTGCTCTACCTGGGATGCAGTCGGTGCCCGTTACCCAGATGTTGAAGGGATTAAATACTTCCATGCCT
GAACTGGTGATTGGACTTGTTGAAATGTTTTTCCTTTTTTCCTCTTTTGTCCCCTGGCACTGGGATGGTG
GTGGTCTGTGGGTGCTGTCTCAGGGTGCCCTTAGAAAAGGACAACTCAGAAGAGTTTCGTCAGCGAGAGC
TGCGTGCGGCCCAGTTGGCTCGAGAGATTGAATCAAGCCCCCAGTACCGCCTACGGATCGCCATGGAGAA
CGACGATGGGCGCACTGAAGAGGAGAAGCACAGTGCAGTCCAGCGGCAGGGCTCAGGGCGGGAGAGCCCC
AGCTTGGCATCCAGGTGACTGTTGCAAACAGCCAGGACTACTTGGGGCTTCTGGGAATATCCTGGGGCCT
GGGCCCAGAGTTGATGAGTGCTGTGGTTTAAGCCTTGTGACCATGTAAAATGTCATACACAGGTTTAGGA
GGTTGAGGTAAAGCGAGATTATGTAATTTGCCTAAAGTAACAAAGCTGGAGCTTGTAGATCTAAAAAAAA
AAAATTCAAAATTTATGTGCTATTTCTTTTTATAAAAGTGAAATATAACTACATTATTTGTGTCTTTTAA
AAACTATCCCTGGAACTACGGTGTGATCCTACTTTTGTCATAGGTCAGGAGTTGTAGAATTGTGGAGCAC
TGGCCAGGTTGGTTGGCTCAGAGTTGATGTCACATGAGGTCATAAGTCCGTAGCCACAGAACGTTAAACT
AGGGCAGATCTTCAAGTGGGATGGTGCTGGAGCCAGGCAGTGTGTGTATGTTTGGGGGCAGAAGGGGGAG
ACTTTAGAAGAAGAAATGGCTTCATCTTTCCAAAACGTGGGTTTTGTGGCTACCTGGCCTTATTTGAACT
CTTTCCTCAGGGAGGGGAAGTATATCCCTCTGCCTCAACGAGTCCGGGAAGGTCCCCGGGGAGGAGTTCG
ATGCAGCAGCTCTCGGGGCGGTCGGCCTGGCCTTAGCTCTTTGCCACCTCGTGGCCCTCACCATCTGGAC
AACAGCAGCCCTGGCCCAGGTTCTGAGGCCCGTGGTATCAATGGAGGTGAGTTATGAGGTGACTTTGAGG
AAGAGGGCAGGGAAGGGGATGCCAAGGAGGTGGGAGGGTAATTGGAGGGGTTTGGGTGTGTTGGAATGAC
GCTGCATCGGTGGGAATGTAATAGGTCGTCTGCCTCCTGACATTTTCTTCTCAAAAAAAAAAAAAAAAAC
CAAACAGGCCCTTCCCGCATGTCCCCAAAGGCACAACGGCCTCTGAGAGGTGCCAAGACTCTGTCTTCGC
CCAGTAATAGGCCTTCTGGAGAAACTTCTGTTCCACCTCCTCCTGCAGGTAAAGCTTTAGTAGTGTTGGA
TGAAGAAATGGATGGAAATTTGTAAAGAGATGTAAATATGTCCATTTGTTACAGTGCTGGAATGGGAGAT
AATTTTAAGATAGATGTTTTGGGCATTTGGGATTTAGTCATATTGGGAAATAGTTCTTCGATTTCCCTAG
TGCAGAAATCTCAACCTAGCAATCCATGGGTTGTGTCTGCTTGGCAGACATCTTTTGTTTAGATTTGAAT
CAGTTGCTAAGTTTTGTTTTGTTTTGTTTTTTTCTTTTTTTGAAACGGAGTCTTGCACTGTTGCCTGGGC
CAGAGTACGGTGGCGCCATCTCAGCTCACTGCAGCCTCCACCCCTCAGATTCAAGCCATTCTCCTGTCTC
AGCCTCCCAAGTAGCTGGGATTACAGGCACCCACCACCACGGCTGGCTAATTTTTTTGTATTTTTAGTAG
AAGCGGGGTTTTATTATGTTGGCCAGGCTGATCTCGAACTCCTGACCTTGTGATCCTCCCACCTTGGTCT
CCCAAAGTGCTGGGATTACAGGCGTGAGCCACCACACCCTGCCAGTTGCTAACTTTTTAAAACAGGCCGA
GGTAGGCAGATTGCTTGAGCCCAGGAGTTTAAGACCAGCCTGGGCAACACGGCAAAACCCTTCTGCAAAA
AATAAAAAGATTAGCTAGGCTTGATGGCGTGCACCTGTACTCCCAGCTACTTGCGAGGCTGAGGTGGGGG
```

Exon 7 with LSMAD first part, encoding SNGWDPNEMFKFNEEN**YGVKTTYDSS LSSYT**

Exon 8 with LSMAD second part, encoding **VPLEKDNSEEFRQRELRAAQLAREIES SPQYRLRIAMENDDGRTEEEKHSAVQ RQ**GSGRESPSLASR

Exon 9, encoding EGKYIPLPQRVREGPRGGVRCSSSRGGRPG LSSLPPRGPHHLDNSSPGPGSEARGING, with the second part badly conserved since *Danio rerio*.

Exon 10, encoding GPSRMSPKAQRPLRGAKTLSSPSNRPSG ETSVPPPPA, badly conserved.

GAATCGCTTGAGCCCAGGAGGCTGAGGTTGCAGTGAGATGGCATCACACCATTGCACTCCAGTCTGGGGA
ACAGAGCGATACCCTGTCTCTAAATAAATAAATCAATAGAAGCCATAGAAAATTTTTAGTTTCTTTTGAA
TTACAGGAAATTCTGTCAACTGAGCTTGCCTTGCTGCGTAGCTTGAGTTGACTGGAACAGAATCCCTGCC
ACTCCTTTAAGCATTCCAGTTGTCGATGATGCCCATGAGGCCATTTACCTGCTTGTGTTACCTGCCTTGA
GCTTCTAGACATTTAAGTTGGTGACTCTGGTTGTATAGTGTGTAAACTTTCTTGCTGTTTTGAGTAAGGC
CCTTGTACTCACTGCTGTTGACCAGCAGTAACCATCCTACAGCTCCCCCTTTTCTTCCAGTGGGCCGGAT
GTATCCCCCGCGTTCTCCCAAGTCTGCTGCCCCTGCCCCAATCTCAGCTTCCTGTCCAGAGCCTCCCATC
GGCTCGGCAGTGCCAACCTCTTCAGCCTCCATCCCTGTGACCTCATCAGTCTCAGATCCTGGAGTGGGCT
CCATTTCTCCAGCTTCTCCAAAGATCTCCCTGGCCCCCACAGATGGTAAGAGCTAGGTGTTTGAGTGCTG
TGAATGCATATTTAGTGTGATTTGTGGTTCTGGACAGAAGGACCTTTAGGCATTTCTCTTTACTTGAACA
GTAAAAGAACTCTCTACCAAGGAACCTGGGAGAACTCTGGAGCCCCAGGAGCTGGCTCGGATAGCTGGGA
AAGGTGAGGGTGGTTTTTTTTCTGCTGAGGATTAATGCTCCTTTGTCTGGGGGAGAGTATTTCAGTTAGG
AAGTTTGTTGGAATTCAGAGGCAAACAATGTCTATCAGTCCTTGGATTATAATTTCACTTCTGTGATCCT
CAAGAGTTTCTCTTTTTTCTTTGCTTTCTTGTCCTCTGTTCTTTTGGCACTGTGTAGCCACCTTAGAGAA
AGAATGTTTTGTATTTTCTTCTTTTTGACTGTTTTCTCATAGTCCCTGGTCTTCAGAATGAACAGAAACG
ATTCCAACTGGAAGAACTGAGAAAGTTTGGGGCCCAGTTTAAGGTGAGAGAAGAGTGAGCTGGGATATTA
GCAGGGTAAAGGGGTTGGGAGTGGTTCGTAGATGAGGCAAAGGACTAGATAGGAGTCAAAGAGATGAGAT
CAAAAAAGGATGAAAGAAGAAAGCCAGGACTAGGGTCTGGGTCAGACTGGACTGTGTGTGTTTCTCTCTT
CCAGCTTCAGCCCAGTAGCTCCCCTGAGAACAGCCTGGATCCTTTTCCTCCCCGGATCTTAAAGGAGGAG
CCCAAAGGAAAGGAGAAAGAGGTTGATGGTCTGTTGACTTCAGAGCCCATGGGGTCTCCCGTCTCCTCCA
AGACAGAGTCCGTATCGGATAAGGAGGACAAACCACCCCTGGCACCATCAGGAGGCACTGAGGGGCCAGA
GCAGCCCCCACCACCTTGTCCAAGCCAAACTGGCAGCCCCCCGGTGGGCCTCATCAAGGGAGAAGACAAA
GATGAGGGCCCTGTTGCTGAGTGAGTGGAGCGGGGTGGGGCTCTGGGAGGATGGCAGGAGGGATGAGAGC
AAGCCGTGAAGATTTACTGTACTTTCTCTCACAGACAAGTAAAGAAATCAACGTTGAACCCTAATGCTAA
GGAGTTCAATCCTACAAAGCCTCTGCTGTCTGTGGTGAGCTGGGACAGGAGAATGTGGACTTTGGTTTCT
GTGGGGAGACTTGGGCAGTGCTTATAGATGAATAGGGGGAGGAACACTTCACTTCCAGGACCACTTGCCT
GGCAGGCAGTGTGAGGAGATGTCATAAAAATGTAAGGATGGCACCTTTGGGGCTGGCTTGGGGAAAATGG
ACTCAGGTCTCTAGGTTTGGGTGATCAGGGGATCAGGGATCCCAGTGAGTCTGATGAGGGGTTAACAGGC
TTTTCTTTGGTTCTTGGCATTTGGCTGAGGGAGTATTGGAGTGGGGTAGTCATGAACAGAGGCCAGCTGA
CTTGGCTTGAGCCCCTGTATTTGGAAGGTTTGTGATCTTGGACGTCACTTACATTCTCTGCCTCACCTGT
AACTTTAGAATACTCATCTCCTCATAGGGTTTAATGTGAGGCTTTATCAAGATAAGTGCAGAATATGTTT
GGTATGCAGCATTTCACGAATGTTAATTATTACCACTCTAGGCATGGCCAGGAGTAGAGGGGAAAATACA
AAATAAAATTGTCCTCCCTTGTTTTTGCAGAATAAATCCACCAGTACCCCAACTTCTCCGGGGCCCCGGA
CTCATTCAACTCCCTCCATCCCGGTGCTGACAGCAGGCCAGAGTGGGCTATACAGCCCCCAGTACATCTC
CTACATACCTCAGATCCACATGGGACCAGCTGTGCAGGTATGCAGAGAGACTGGCCGGGCCCAGGGTTAG
CGGGGTGGGATTTGGTTGCGCTGGTTGAGGGACCAGGTCAGGCCTGTCTGGGCATTCGTGAGCGAGTCAT
TCAGCCTCATCTGTGTCCTCATCCCCAGGCACCTCAGATGTATCCATATCCTGTATCCAATTCAGTGCCT
GGGCAGCAGGGCAAGTACCGGGGAGCAAAAGGTGAGCAGGGCTGGGAGGGGCAGGCGGCGAGGCTGCCAA
GGGCCTACTGGCAGGTGGAGCTTGAGCTCTCCTCTCCTCCTCTTCCAGGCTCCCTTCCTCCGCAGCG

Exon 11, from (appflp)VGRMYPPRSPKSAAPAPISASCP EPPIGSAVPTSSASIPVTSSVSDPGVGSISP ASPKISLAPTD,

Exon 12, encoding VKELSTKEPGRTLEPQELARIAGK, badly conserved.

Exon 13, encoding VPGLQNEQKRFQLEELRKFGAQFK, well conserved

Exon 14, encoding LQPSSSPENSLDPFPPRILKEEPKGKEKE VDGLLTSEPMGSPVSSKTESVSDKEDKP PLAPSGGTEGPEQPPPPCPSQTGSPPVG

Exon 15 with PAM2 motif, encoding QVK**KSTLNPNAKEFNPTKP**LLSV

Exon 16, encoding NKSTSTPTSPGPRTHSTPSIPVLTAGQSG LYSPQYISYIPQIHMGPAVQ

Exon 17, encoding APQMYPYPVSNSVPGQQGKYRGAK

Exon 18, encoding GSLPPQRSDQHQPASAPPMMQAAAAA GPPLVAATPYSSYIPYNPQQFPGQPAM MQPMAHYPSQ

CTCGGACCAACACCAGCCAGCCTCAGCCCCGCCGATGATGCAGGCCGCCGCGGCTGCTGGCCCGCCTCTG
GTGGCTGCCACGCCCTATTCTTCCTACATCCCCTACAACCCTCAGCAGTTCCCAGGCCAGCCAGCCATGA
TGCAGCCCATGGCCCACTACCCCTCACAGGTGACTGCGGCCCAGGAGGGCAGTGAGGATCCAGGGCCCCT
GCTAGGGATCCCATCTTCTCCAGAGACTTGGGAGCTGGCTAGGGGTGGCAGGCAGTGTTGTAGGTGGGAT
CGGCCCTCTGTGGTATTGGCGGTGTCAGACTTGGGCTTGAGCCCTGGCTCTGGTGGTACCTGTAACAAGG
CATTGGACATCTGTATCTCTGAAGTGTAGAGAAAATAGTGTCTGCTGGGTGGGATCGTTATGAATGTTGA
ATCAATAGGGTGATTGTGAGGAGGCCCAAGCGGTGCTGTGCACGCAGTGACTGGCAGGAGGACACCTTCC
CAGCTGGCGGCTGTGCCAACCACTCCTCTCTCTGTCCCGCCAGCCGGTGTTTGCCCCCATGCTTCAGAGC
AACCCACGCATGCTGACGTCGGGCAGCCATCCCCAGGCCATCGTGTCATCCTCTACCCCTCAGTACCCTT
CTGCAGAGCAGCCTACCCCCCAAGCCCTTTATGGTGAGTCCTGCGCCTGGTCCCTCTGCTCTGGGCTGTG
TGCCAGCCCCCTCTGGTGTGCTCAGCACTGGTTCTCCCTCTTTCCTGCTGCAGCCACTGTTCACCAGTCC
TACCCACACCATGCCACACAGCTCCATGCCCACCAGCCGCAGCCGGCTACCACGCCTACTGGAAGCCAGC
CGCAGTCCCAGCATGCGGCCCCCAGTCCTGTCCAGGTGCCTGCCATGGGGGGTGCTGAGTGGTCCTGGTG
CAGGAATGGGTGGCCAGAAGAAGGGATAGAGCTAGGGGTCATTTCTGAGTGGCGAGGACTGGGGGCCAGC
GAGTTGCTGGCCTGTGTGGCACTCAACCTTCCCCTCCCCAGCAGCATCAGGCGGGGCAGGCCCCACACTT
GGGCAGTGGACAGCCACAGCAGAATCTGTACCACCCAGGGGCCCTGACAGGCACGCCGCCCTCTCTGCCA
CCGGGACCTTCTGCCCAGTCCCCTCAGAGCAGCTTCCCCCAGCCAGCCGCTGTGTATGCCATCCACCACC
AGCAGCTGCCCCACGGCTTCACCAACATGGCCCATGTTACCCAGGTAAGAGCCCAGCTGTCCCACTTCTG
GGTCTGTTTGCCAGGGCCCGTCTGCCATGGGGACCATCCCATTGCCAAGTCCCTGGTGCCACCCTTGCCA
TAGTGCTCCCTAACTCTGGCTCTCAGAGTCTGTTTCAGGATTCTGTGGTCTTCCCGGCTACTTTTTTGTT
TTCCACAGGCCCATGTCCAAACTGGAATCACAGCAGCCCCGCCCCCTCACCCTGGGGCTCCCCACCCGCC
CCAGGTGATGCTGCTGCACCCACCCCAGAGTCATGGGGGGCCCCCCCAAGGCGCGGTGCCCCAGAGTGGG
GTGCCTGCACTCTCAGCTTCCACACCCTCACCCTACCCCTACATCGGACACCCCCAAGGTGAGCAGCCTG
GCCAGGCGCCTGGATTTCCAGGAGGAGCCGATGACAGGATTCGTGAGTTCTCATTAGCTGGGGAATTTG
GCATGGAAGAGCTGAGGGGCTGCAGGTGGGGCAGGATGCACGGGTTCTGGGTGGGGAGTGAGGGGTCTTG
GAGGCAGGGCTGTCCCACAGGGCGCCCGCCGACCTGCACCTGTCTGTGAAGTATGTAGGGTGGGCAGAAG
CCACAGTCGCCGCCGCCAGGGGCTTGCTCCTGGCTCTGTCCTTTGCTTCCCTCCGTCCTCGCTCAGTTGT
GATCCAGCAGCCCCCCTCCCCACTGCCTCCCCAGCTCTCAGTGACCCCGACTGTCTCCTGACTTAGCCGA
GGTAAGGTCAGTGCAGCAGACAGGGCCAGACTGGGGTGTGGGGGGCTGAGCTGGGCACATGAGTGAGGGC
TCTGGCTTACTGGGAAACAGCGATTGACCTGTGCTTCTGACAGCCCCCGAGACACCTTGAGGAGGCCGCT
CCTTCCCAGACACACCCCCACGCCCCCACTGGACGGCATTGGAGGAAGGGACAGCTGCTTGGGTTCTAAT
GCTCCTGCTCTCTTCTCTTTCCCCTCCAACCAGTTCAATCTCATCCCTCCCAGCAGCTCCCCTTCCACCC
CCCGGGGAACTGAAGATTGTCCTGGCCGCGACCTGAGACCTCCATGAGTGGAGGGAAGAGTGATCTATGT
CTCTTCCCCCAGCAGCTCGGACCACTCCCAGCCCCCCATCCCCCCGTTCCCCAGGGGAGCTGGGGAATTC
CTGCCAAGCACCTTGAATGGGAGGGGCCTCACAGAGGGCAGGGCCAGGGTCCAGCAGGGGTGGGGGGTTC
CTGCTCTGCCCCTGCCCGTCCCCACCCAGTCTTGCCCTCCCATCCTCTCATCTATTCCCCCGCTGGAGAC
GGAAGATCTTTTATTTTCTATTATTTATAACTTCAGACTTGGGCCCCCTGTTCTTTCTTTCCCATTAACT
TGAGTGACCTGTGTGAGAGACAGACAGATGCCCCACGAGGATGGCTGGACAAGGACTTTTACTTTTTATT
ACATAAAAATATTAAAAAATAAATAAAAAAAATAAAATTTTAAACTAACTTAACCTGCCTGGAGTTTCCT

Exon 19, encoding PVFAPMLQSNPRMLTSGSHPQAIVSSST PQYPSAEQPTPQALY

Exon 20, encoding ATVHQSYPHHATQLHAHQPQPATTPT GSQPQSQHAAPSPVQ

Exon 21, encoding HQAGQAPHLGSGQPQQNLYHPGALTG TPPSLPPGPSAQSPQSSFPQPAAVYAIHH QQLPHGFTNMAHVTQ

Exon 22, encoding AHVQTGITAAPPPHPGAPHPPQVMLLHPPQSHGGP PQGAVPQSGVPALSASTPSPYPYIGHPQ as exon 22A, or including GEQPGQAPGFPGGADDRI as eon 22B, or including REFSLAGGIWHGRAEGLQVGQDARVLGGE as exon 22C

Exon 22B, alternatively spliced, encoding GEQPGQAPGFPGGADDRI

Exon 23A, alternatively spliced, encoding LCRVGRSHSRRRQGLAPGSVLCPPPSSL SCDPAAPLPTASP ALSDPDCLLT

Exon 24A, alternatively spliced, encoding VQSHPSQQLPFHPPGH

Exon 24B, alternatively spliced, encoding PPLPPPGELKIVLAAT

CCTTGGAGACTAGAGTGGGGTGACAGCTGTCAGTCTTTTGTGGCATTGATAGGTGCTGCATTGTGACCTG
TTCCTTCACCTCCATGTAAAAATCGTGTCCAGTGGCCAGCCATGGTGGCTCACGCCTGTAATCCCAGCAC
TTTGGGAGGCTGAGGTGGGCAGATCACCAGGTCAAGAGATGGAGACCATCCTGGCCAACTTGGTGAAACC
CCATCTGTACTAAAAATACAAAAATTAGCCAGATGTGGTGGGACATGCCTGTAATTCCAGCTACTTGGGA
GGCTGAGGCAGGAGAATCACTTGAACTCGGGTGAAACCCTGTCTCTACTAAAAATACAAAAATTAGCCTG
GCGTGGTGGGACATGCCTGTAATCCCAGCTACCTGGGAGGCTGAGGCAGGAGAATCACTTAAACCCAGGA
AGCGGAGGTTGCAGTGAGCTGAGATCACTCCATTGCACACCAATGGTGGCCAGTTTTTGAAATCTAGGAT
GATGGGGAGCCAGATCGGGATCCTGTGCCATGCATTTGAAGGTGGGTTCCAGACTTCATGTGAAGTTGTG
TAGTTGATGACTGAGGTTCTGGATGACCGGATGGCTAAAAGCACGTGAGATGGGAGTCCGCTTCCCAGGA
TGGGACAGCAGTGGCACAGTTCCCAAGGATGCTGTCCCTGACTGGCTCTGGAACTGCTACATAGATAGCT
CACCCAGCTGTCTAGTGACTATTTTCTCATCTTGAACTTGGCTTCATCTCAAGGTGTGATTCAAAGTTTC
ATTTCTAATATGAGTATCTCATGAATTACATACACCTCTTTATGTTGAAGGGTGAATGTTTATGGTCATT
GGAATTAAAATCCTGATGGATGTGCAGCTGACTGTGGATGAGAGCTGTAAAGGTGAAGCTGTTAGTGGGA
GAAGAGGGGAGAGGATTGGTTGGGAACTGGGCTTACTGGGGAGCAAGAGCAAAATTAGGCATTATGGGTG
GGACAAGAAGATTGAAGCATGGGATGTGGAAATGACAGCAGGCTCCAGAGGAGGTAGTTTTTCAAGGAGG
TTGAGAATTCTCTCGGAGACTTGACCTTGGTTTCCTTGCCTAAAATGGAGGTGATTTCACCTTTCCAGGA
TTTTAAGGAAGTGGCTGGTGCTCAGTGGTAGACGGGTGTTTCAGCTCTCACCAAAGCCAGAGAAAGCTGG
CTGAGACCAGGACAGGAAGAGTGAAGGCTACAGTATTGGGGAAGAATCCCAGCATATGCAGATTTGGAAC
TTGGTAATAGGTGTACATGGTGGGGTGGAGAGAAGGGGTTATTAAAGAGGTTAAAAAAAAAAAAAAAAAA
AAGGACTTTATGTGGTAGCCACTTGGATGCTAAAAATGGGCGGGGTTGGAGTCCCAAGAGCACTCAGCAG
