# Supplementary material for: Bioinformatic Analyses of the Ataxin-2 Family Since Algae Emphasize Its Small Isoforms, Large Chimerisms, and the Importance of Human Exon 1B as Target of Therapies to Prevent Neurodegeneration
Source: Int J Mol Sci. 2026 Feb 3;27(3):1499. doi: 10.3390/ijms27031499 (PMC12898128; doi:10.3390/ijms27031499)
Supplement: Supplementary file 1 [file ijms-27-01499-s001.zip › AuburgerSen_SupplMaterialS4-HomoSapiensATXN2genomic.pdf]

Homo sapiens ataxin 2 (ATXN2), RefSeqGene (LRG_864) on chromosome 12

NCBI Reference Sequence: NG_011572.3

GenBank Graphics

```
>NG_011572.3:5362-152463 Homo sapiens ataxin 2 (ATXN2), RefSeqGene (LRG_864) on chromosome 12
CCCGAGAA
AGCAACCCAGCGCGCCGCCCGCTCCTCACGTGTCCCTCCCGGCCCCGGGGCCACCTCACGTTCTGCTTCC
GTCTGACCCCTCCGACTTCCGGTAAAGAGTCCCTATCCGCACCTCCGCTCCCACCCGGCGCCTCGGCGCG
CCCGCCCTCCGATGCGCTCAGCGGCCGCAGCTCCTCGGAGTCCCGCGGTGGCCACCGAGTCTCGCCGCTT
CGCCGCAGCCAGGTGGCCCGGGTGGCGCTCGCTCCAGCGGCCGGCGCGGCGGAGCGGGCGGGGCGGCGGT
GGCGCGGCCCCGGGACCGTATCCCTCCGCCGCCCCTCCCCCGCCCGGCCCCGGCCCCCCTCCCTCCCGGC
AGAGCTCGCCTCCCTCCGCCTCAGACTGTTTTGGTAGCAACGGCAACGGCGGCGGCGCGTTTCGGCCCGG
CTCCCGGCGGCTCCTTGGTCTCGGCGGGCCTCCCCGCCCCTTCGTCGTCCTCCTTCTCCCCCTCGCCAGC
CCGGGCGCCCCTCCGGCCGCGCCAACCCGCGCCTCCCCGCTCGGCGCCCGCGCGTCCCCGCCGCGTTCCG
GCGTCTCCTTGGCGCGCCCGGCTCCCGGCTGTCCCCGCCCGGCGTGCGAGCCGGTGTATGGCCCCTCAC
CATGTCGCTGAAGCCCCAGCAGCAGCAGCAGCAGCAGCAGCAGCAGCAGCAGCAGCAACAGCAGCAGCAG
CAGCAGCAGCAGCAGCCGCCGCCCGCGGCTGCCAATGTCCGCAAGCCCGGCGGCAGCGGCCTTCTAGCGT
CGCCCGCCGCCGCGCCTTCGCCGTCCTCGTCCTCGGTCTCCTCGTCCTCGGCCACGGCTCCCTCCTCGGT
GGTCGCGGCGACCTCCGGCGGCGGGAGGCCCGGCCTGGGCAGGTGGGTGTCGGCACCCCAGCCCCCTCCG
CTCCGGGCCCGGCGTCCCCTCCCCCGCGGCCCGCGCCGCCGTCCCCGCCCCGTGACCCGCCGGGCTACCC
GGGGTGGGCTGGGGGCCGGCAGCGCGGGGGAGACTCGCTCGGGCCTGAGCCCCGAGGCTCGGCCGGTGGG
CGCAGCCGGGGTCCTCTGGGATTGTCAGGCCTGTCCAGCCTCCCGCAGCATCCCCGCCCCCTCCCCCGGC
GGTCAAGATGGAGGGAGCGGGCGGCCTCCCCTCCCCACGCGTGTTGGGAGGGGTTCTCGGGTAGCGGCGA
TGGTCAGCCCCGGCTCCCCCTTCCGCACGATCCTCCGCCCGCAGCGTGGGGATGCTCGGGCAGCTCCTCC
ACTCCCGGTTTAGGTGTGAACGTTGGAGGGGTCTGGAGGCTGTGGTGGCGTTTTCCGGAACATGTCCCCC
TCCATGGGGGACATCTCTGGAGGGGAGAAGTTAGGGCCGCGTCCCCCGTGCCGGTTAAAGGGGTAGGCAC
CGGGCTCCTCCGGAATCATCAGGGTCTGTCGGGGCTCTCTCCCCGCCCCCTCCGAGTCCTGGGAAAGATC
GGAGGACGGGGTGGAGACAAGTGGGCCTTGGCCCCCGCACCCCTCTGCGTTCGTGTCCGAGGCGGCGGCG
GGGGCTCCCGAACTCCCCTGAAATCGTGGGGCTCCATGTGGCCTCCGGCAGCGTTCCACCCTCCCCCACC
TGGGGAAGGGAAGGGGTGGGGAGTGCCCGGCCCCGTCCCGGCCTTCCTCCTTCCCCCGCCAGACCTCTCC
GGCGCGCGGGTGGTGGCCGATCCGCATTGCTGTTCGAGGCCGCAGTGGAGAAGGCGCCTGTGGAACATCG
GTGGGTGAGGGCTGGACCCAGGCTGGACCCTGGAGATCCGGGGTGGCGGTGCTGGTGGCAGGGGGCGGGC
```

Exon 1A with very rarely used start codon encodes a sequence with mitochondrial targeting peptide and proline-rich motifs:
MRSAAAA**P**RS**P**AVATESRRFAAARW**P**GWRS
LQR**P**ARRSGRGGGGAA**P**G**P**Y**P**SAA**PPPP**G**P**
G**PPP**S**R**QSS**PP**SASDCFGSNGNGGGAFR**P**GS
RRLLGLGG**PPRP**FVVLLL**P**LAS**P**GA**PP**AA**P**T**R**
AS**P**LGA**R**AS**PPR**SGVSLA**RP**A**P**GC**PRP**ACE**P**

Exon 1B with rarely used translation start MSLKPQQQQ, undergoing evolution with long and expansive polyQ, polyP, polyA, polyS and polyG sequences a spart of intrinsically disordered domain 1 (IDR1), and containing two glycine-arginine motifs. Encodes
MSLK**P**QQQQQQQQQQQQQQQQQQQQQQ
QPPPAAANVRK PGGSGLLASPAAAPSPSSS
SVSSSSATAPSSVVAATSGGGRPGLGR

ACCCTGCGCACTTATCCCAACCCCCGCCCCAATTTCGGAAATGCTAGGAGAGAGAGATTGCAGCAGGGGA
CGTGGTCGGGTTCCTGAAGGCAGAAAGGCGGGTGTTTACTAGCGTCTTTTTCCCTCCTAAGCCGGGGTTG
TAGTAGGGGCTGGGGGCTCAGTGTTGTCCCGGCTAACTGGGTTTGACTCGAGGGTGTGTTTGTGCAGGAG
GGCCTGTTGGGGGTGGCGGGCGGTTGTCAGTTCGTATTTCACGAACTAAGAAAATGCTTAGTGTTCAAAG
GGAGAAGGAAACGTCAATAGACTCCATTCCATTGTGGCCGGTGTCCTTAACTTCGGGAGTGCCGCCAGAG
CTTACCAAGGGCACGCAAGTCCATTTCCCTTGTGCCTCAAGTCCATCCGTGTTGTAGGCACTACTGTGCC
TTCTTTAGGCCTAGGCCGCCGGCTTGACGGCGGGTGACCGGCGTCCTCCTTAAATAGGCATCTTGGGCTT
TGGAAGGTGGAATAAGAGGATTTTTCATTCACCCGAGTTTTCTTTTTGAAAACACATTTTCAGCAACCCA
TTTCCAAAGAATTTTTATTTACAGCAGAAATTCCCCATCAAGAGGAATCAGCTGGTTTTTAAGGAATTCT
GCTGCCTTCAAAGGGGGCGGAAACAGTCGGTTATTTGACTTTACACGCCCCGCCCCCCCTTCCCCTTCTC
TGAGTCTGAAGCATCCCAAACACTACTTAGCCAAACTAGTTCAGATGAAGTGATCGTTTCCCCAAGTAGG
GTAACTTCAGTTTCCCTTTTTCGTTGGCATCTAGCGAAAAATGAAAAAATTTAAAATACAACTTTTATAG
AAAAGGATGTATTCTGTTTTTACTTTCTTAGGTATTAGGAAGAGATTTGGCAGATAATTCAACATGTTCA
AATATATAAACATTAAAACTAAGGTTATTAAGTTGCATTGACTACTAGGCTTAAAAATTAGATTATAAGA
GAATTTGCTCCTGAGTAGTTTGAGTGATCAAAGATATTTGGAATGTTTTAGTACCACAAGGTCTTTTTTC
TGTTCCTTGAGGCTTTACAACAATTTAAGGTTAATTTAGATTTTTCCTTGCTTTAAGTTCTTTTACTTGA
GACCTAAATGGCAGCCCTTATTCTTTCTGATGAATAGGTGAAATTTTGTTTACTGTGTTGGATTTGTGTA
ATGTGAAGTTTTATTCTTGAACAGATCGTTAATGTACTTGTAGAATTACTTTGAATTTGAATCACTTTCC
TGCATTCCTTGTAAATAAGTTTCAGCTTCTAGAATCTCCTCACTTAGGTTTGTGCGTATCAACAGTGAAA
ATAAGTCTCTGAGAGCAAGGGTGAAAAAAAATGCAGCATTCGGTTTGACAAGTTTCGAGATAGCAAAATA
TGCTTGAAAGTCTGGAAATTCACATCTGCTTTAAGAAACATTTCATAATTTGACTTTGTGTGTGTGTGTG
TGAATAGTTTTTCATGACTTTCAGAAGTGATTTATTTTGTTCTTTGTTATATATATATTTTTGAAGGTGGCT
GTTTTAGGAAAGATAATGTAATCACAATATTAGAACATAATTTTACTGTAATCTAATTTTGTGTGTGTGT
GTGTGTGTGTGTGTGTGTGTGTGTGTGTGTTTTGGATGGAATCTCACTCTGTCGCCCAGGCTGGAGTGCA
GTGGCCTGATCTCAGCTTACTGCAGTCTCTGTCTCCTGGGTTCATTTAAGTGATTCTCCAGCCTCAGCCT
CCCCAGTAGCTGGGATTACAGGTTCGTGCTACCACACCTGGCTAATTTTTTTGTATTTTTAGTGAGGACG
GGATTTTGCCATGTTGGCCAGGCTGGTCTCGAACTCCTGACCTCAAGTGATCCGCCTGCCTTGGCCTCCC
AAAGTGCTGGGATTACAGGCGTGAGCTACTGCCCCTGGCCAATTTTTGTATTTTTAGTAGAGATGGGGTT
TCACCATGTTGGCCAGGCTGGTCTCGAACTCCTCCTGACCTCAAGTGATTCGCCAGCCTCGGCCTCCCAA
AGTGCCAGGATTACAGGCAGGAATGAGCCACTGCCCCCAACCATCAGTCTAATTCTTATTTTTGCTTTTT
ACCTTTTCATTTTTATGTAGTAGAGGTGATTGTGTATGTTATTTTGTAGTTAGCTTTTTTCCCCTGAACG
TTGTATTGTAAATGTAAATTTTTTTTTTTTTTTTTGAGACAGAGTCTCGGTGTTTGCCCAGTCTGAAGTG
CAGTGGTACGATCTCAGCTCACTGCAGCCTCTGACTCCTGGGTTCAAGCGATTCTCCCACCTCAGCCTCT
TGAGTAGCTGGGGCTACAGGAATGTTCCACCACGCTTGGCTAATTTTTGTATTTTTGGTAGAGACAAGGT
TTCACCATGTTGGCCAGTTTGGTCTCGTACTACCGACCTCAGGTGATGCGCCCGCCTCGGCCTCCCAAAG
TGCTGGGATTGCAGGCGTGAGCCACTGCGCCCGGCTGTAAGGTTTTTACTTAACCATTCTATTGTTGGGA
ATTGGGTTTCCACTTTTTTGTTATAGATAGTGGTGCAGTGAACATTTTTAAATAGCTTTTTGCTTCAGTG
TAATTATTTCCTTAGAGAAAGTTACCAAGAGTGGTTTTACTAGTTCAGAGGGCTTCAGGATTTTTATGGC
TCTTGCTAGCGGTGCTCTATTATTCTTTAGAAGACTTGTATTACTTCCAGTGTCAAGAAGGTTGCTCTTC

CATGGAATGGTTTCTTTGTAGTTTGTCAAATATTGTGGGGAATTTTTAAAGGAAAAATTGCATTTTTACT
GTCAAGTGCATATATTATTAAGTGCTTTTGTTAGTTACTGGATTATTGATATTTGAGTTTAATTTGGTTC
CTCTGAGGATTTAATAAGGTAATATATGTGAAGATGTTTTGAAACCTGTAACCATTATTATTAATGAGGG
TACTTGGTTTATCTGTCGTGCTGATAGTACTGAGTAAAGTGCAGGAATGAAATTCCTGAGGAACTGTTCT
AAAGCTTTGTTGTTGTTGTTAACCTTTCTTTTTCATCTGAAAGTGTTTTTTATTAGCTGCTAGCCTATGA
CCAAGTTATTTTTGGTAACTTTTTTGTAATTTCATGGCACTATTGGGAATTTTCGCTGGTTGACTCTTCT
TCTTCTACATTCCCTTCCCCATTAAAAATAAAAATATGGATTTACAATTGTTACTCTATTCCTAAACCTA
AATAATATGACATTAGAATTGCTTGGGATACAGGATTCAGTCTGAATAAAATATTTTTCTTTTAGTGATT
TTCAGCTTAGTATTTTTACTGCTTCTTTCTCTTGAGGCATTGCAACTTAAAAATTGTGCTGTTTAGCCAG
GCGCCTGTAATCCCAGCTACTTGGGAGGCTGAGGCAGGAGCATCACTTGAGCCCAGGAGGCGGAGGTTGC
AGTCAGCTGAGATTGTGCCACTGCACTCCAGCCTGGGAGACAGAGTGAGACTCTATCTCAAAAAAAAAAA
AAATGTGCTGTGATTTAATGTAGTTGTTCATCATGCTTCCATTTAAATTTCAGTGAGACTGTTCATCTTT
TGCAGTTAAATATCTTGTAGAAGGGCCTAAAATATCTACGTTGAATACAGCTTTATTGAAGCATCTATGT
ACATGGGGTTTTTGGGATGAATCAGTGAATAAAGCAAACATATTGTCCTTTTGGAGTTTACATTCTAATG
TGACTAGGCAGACAATGAGACATTAAATTACCAGCCTATGTATAATAGTGTATAAGAGCTATGGAATTAG
AAGAAAGCAGATTAAAGGTATAGGGAGTGTGGGGAGGGGAATGAGTTACAATTTTAAATGGATTGGGGGA
ACTTAATTGAGGAGCTAACATTTGAGCAAAGATTTGAAGGTTGGGTATTTAGCCGTTTGCTTTTTATCTA
GGTTAATTAGTCATGTGGCTTCATTAGTAATTTATAAGGTTTAAATGGCATCATCCTTTGTTATTCTTTT
ATGTGCACATTGATACTAACCATCTCTGAAGTTAGACCAAAAAAGTTAATTGACATTGAGGGTCATTAGA
GGTAAATTGTAGATGGCTATTACTAACCAAAGAGACATGTTTTGTTTTTCTTTTGGGCTTACGTATTTTA
CCTAATTAGTTTAGTTTTTGTTTCAAGTATGTGGAGAAAATAAACTTTTTAAGTTTGGGCCAAAACTTGC
TTTGGTTTTCTTTTTCTTTTTCTTTTTTTTTTTTTAAGAGAAAAATGTAAGCCTGTAGTTGCTTAAAGAT
TCCACATTCTGAAACAGTGAAAACATGGGATCAGTCATGGTGTTCCTTTTTTTGGTTAAATGTAAACTTG
TATTTTCAGTGTTACTCTAATTAGCAATGGTTTATACTTCTACATAAGGGATGTTAACTCATATTGTAGC
TATTTAATAGCCATATATTTTGACTTAAAGGAGGATCTCAAGGCCAGGCGCGGTGGCTCATACCTGTAAT
CCCAGCACTTTAGGAGGCTGAGGCGGGTGGATCACCTGGGGTCAGGAGTTTGAGACTAGTCTGGCCAACA
TGGTGAAACCCCCATCTCTACTAAAAATACAAAAATTAGCCGGGCATGGTGGTGGGCGCCTGTAATCCCA
GCTTCTTGGGAGGCTGAGGGAAGAGAATTGCTTGATCCCGGAGGTTGCAATGAGTGCGGAGGTTGCAGTG
AGCTGAGATCATGCCATTACACTCCAGCCTGGGCAACAGAGCGAGACTCTGTCTCAAAACAAACAAACAA
ACAAAAAAAGGAGGATCTCATTTTTTTGTCCTAAATAGCTACAGCCGTGTTAGAACTGTCACCTTAGCAA
AGTATTGTTTTTTTACTTTGAAACGAATTTTAAGGTTTTAGAAGATTGTTCTCTAGAATTACAATTTTCT
GTTTTGACTAGTGATAGTATTTTGATGTTGTGTAAATAGTTGAGCATGAACAAAACCCTATTTTTTTTTT
TAGCTATTTCAAGTGATTGTGACAACTTCAACGGAGATGTAAACAGTTTATTAACAGTCACACCTATTAT
CTTTTTTTTTTTTTTTTTTTTTTGAGACGGAGTCTTGCTCTGTCGCCCAGGCTGGAGTGCAGTGGCACGA
TCTCTGCTTACTGCAACCTTTGCCTCCCGGGTTCAAGTGATTCTCCTGCCTCAGCCTCCTGAGTAGCTGG
GTCTACATGCGCACACCACCACGCCTGGCTAATTTTTGTATTTTTAGTAGAGACAGGGTTTCACCATGTT
GGCTAGAATGGTCTCAAACTCCTGACCTCAGGTGATCCACCTGCCTCAGCCTCCCAAAGTTCTGGGATTA
CAGGCATGAGCCACCGTGCTTGGCCGCTGCCGTATCTTTTTAAATGAAAGTACTTGTGTTTTTTTTGTTT
TTTTCCAAAGGATATCTGGGTCATCTATGATGTTACTGTTACCATCTAAGGGTTTTTTTGTTTGTTTTTG

AGACAGAGTCTCTGTCGCCCAGGCTGGAGTGCAGTGGCGTGATCTTGGCTCACTGCAACCTCCGCCTCCC
AGGTTCAAGCAATTCTCCTGCCTTAGCCCTCCCGAATAGCTGGGATTACAGGCACCCGCCACCATGCCTG
GCTAAGTTTTGCATTTTTAGTAGATATGGAGTTTCACCATGTTGGCCAGGCTGCTCTTGAACTCCTGACC
TCAGGTGATTCGCTTGCCTCGGCCTCCCAAAGTGCTGGGATTACAGGCGTGAGCCACCCCCGCCCAGCCT
CATGAGCTAAGGTGTTTTTTTTTTTTTTGAGACAGTTTTGCTCTTTCCCAGGCTGGAGTGCAGTGGTGCA
ATCTCAGCTCACTGCAACCTCTGTTTCCCGGGTTCAAGCGATTCTCCTGCCTCAGCCTTCTGAGTAGCTG
AGATTACAGGTGCCTGCTACCCCACTCAGCTAATTTTTGTATTTTTAGCAGAGACAGGGTTTCACCATGT
TGGTTAGGCTCATCTCGAACTCCTGACCTTAAGCGATCCACCTGCCTTGGCCTCCCAAAGTGCTGGGATT
ATAGGCATGAGCCACCGTGCGCAGCCTACCCTGTCTCTTAAAAAACAGTAACAACAACAACAACAACAAA
AAATCCTAAATCTTAAAAATGGAAGGCAAAAACTCTAAGCTTTGAGAGATTAGGGGACTTGCCCAAAGCA
ATATTTGTAGGATTTTATTACACCTCTCCCTTTATTTATTTTTTTAGAGTCAAGGTCTCCCTCTGTCACC
CAGGCTGGAGTGCAGCCTCAATCTATGGGGCCAAGCATTTCTCCTGTCTTAGCCTCCTGAGTAGCTGGAA
CTACAGGTGTACACCAGCTGGCTAACATTTAAATTTTTTGTAGAGACAGGGTCCTGCCATGTTGCCCAGA
TTGGTCTCAAACTCCTGGGCTCAAGTGATCCTCCTGCCTCAGCTTCCCAAAGTGCTGAGATTACAGGTGT
GAGCCACTGCACCGAGCCCCCTCCCTTTATTTTTATTTTTAAATTTTAAGTTCTGGGGCCCCTCCCTTGA
AATAAATAGAAACGTAATATATACACAAGATCATGCTGTGTATTTTAAGGCAATGGTCCTCAACCTTTTT
AACACTAGGGACCGGTTTTGTGGAAGATGGTTTTTCCATAGGGGCAGGGGATGATTTTGAGATGAAACTG
TTCCACCGGCCGGGCACGGTGGCTCACGCCTGTAATCCCAGCACTTTGGGAGGCCGAGGCGGGCAGATCA
CGAGGTCAGGAGATCGAGACCATCCTGGCTAACATGGTGAAACCCCCCTCTACTAAAAATACAAAAAAAT
TAGCTGGGCGCGGTGGAGGGCGCCTGTAGTCACAGCTACTCCGGAGGCCGAGGCAAGAGAATGGCATGAA
ACCCGGGAGGCAGAGCTTGCAGTGAGCTGAGATAGCACCACTGTACTTCAGCCTGGGGGACAAAGTGAGA
CTCCGTCTAAAAAAAAAAAAATTGTTCCACCTCAGATCATTATGCATTTGTTAGATTCTCATAAAGAGCA
TACAACCTACATCTCTTGCTATATGCAGTTCCCAGTAGGGTTTGTGCTTCTATAAGAACCTAATGCTGCA
CCTGATCTAACAGGTGGGGCTCAGGTGCTAATGCTCACACAGCTCCTGTTGTGCAGTCTGGTTCCTAACA
GGCCTGTTTTTTTTTTTTTAATTAGATGGAGTCTCGCTCTGTCACCAGGCTGGAGTGCAGTGGCACGATC
TCAGCTCACTGCAACCTCTGCCTCCCGGGTTCAAGCGATTCTCCTGCCTTAGCCTCCCATGTAGTTGGTA
CTACAGGCGCACACTGTGATGCCCAGCTAATTTTTGTATTTTTAGTAGAGACGGGGTTTCACCATGTTGG
CCAGGATGGTGTCGATCTCCTGACCTTGTGATCCGCCCAACAGCCTCCCAAAGTGCTGGAATTACAGGCG
TGAGCTGCTGCGTCCGGCCCCCTAACAGGCTTGTTTTATGGAATACAGTCACGGACAGTACTTGCCCTTC
AGGATATCTTTTTGTAACCTTGATTTTGGCTTGCTAAAATAGGAGGTCTATTTTCTTTTCTTTGTTTTTA
ATGTATGTGGTTCTGTACTTACGTGGTGTGAAATCTACATAAATGTTAAATCCTTGGTTATTTATTTATT
TTGAGACAGAGTCTCACTCTGTCACCCAGTCTGGAAAGCAGTGGCATAATCTCGGCTCACTGTAACCTCC
ACTTCCCAGGTTCCAGTGATTCTCCTGCCTCAGCCTCCTGAGTAGCTGGGATTACAGGCATGCACCACTA
CACCTGGCAAATTTTTGTATTTTTTTTTAGTAGAGATGGGGTTTCACCATGTTGGCCAGGCTCGTCTTGA
ACTCCTGACCTTAGGTGATCTGCCTGTCTTGGCCTCCCAAAGTTTTGGGATTACAGCATGAGCCACTGCG
CCTCGCCTTATTTTTTTGAGACAGGTTCTAGCTCTGTCACCCAGGCGGGAGTGCAGTGGTGCCATCATGG
CTCATTGCAACCTCGAGTTCTCAGGCCCAAGTGATCCTCCTATCTCAGCCTCCTGAGTAGCTGGGACCAC
AGGCATGCGCCACTATGCCCAGCAAAATTTTTGTTTCACTCTGTTGCCTAGGGTGGGGTGCAGTGGCAGG
ATATCGGCTCAGTGCAACCTCTGCCTCTTGCGTTCAAATGATTCTCATGCCTCAGCCTCCCGAGTAGCTG

GGATTATAGGCATGCGCCACTACACCTGGCTAATTTTTGTATTATTGGTAGAGATGGGGTTTTATCATGT
TGGCCAGGCTGGTCTCGAACTCCCGACCTCAGGTGATCCATATACCTTGGCCTCCTGAAGTGCTGGAATT
ACAGGCATAAGCCACTGCGCCTAGCTTTTTTGTTTGTTTTTATTTTGTAGGGACAGAGATTTTACCTGTT
GCCCAGGATGGCCTTGAATTCCTGACCTCAAACAATTTGCCCTCCTTGGCCTCCCAAGGTGCTGGGATTA
CAGGTGTGAGCCACTATGCCTGGCTGGTTTTTTAAATTATTATTATTGTTTGTGTGTGTGTGTTGCAGGA
TCTTACCCTGTCACCCAGGCTGGAATGCAGTGATGTGATCTCGGCTTACTGCAACCTCCGCCTCCTAGGT
TCAAGTGATTGTCCTGCCTCAGCCTCCTGAGTAGCTGGGATAACAGCTGTGTGCCACCATGCCTGGCTAA
TTTTTGTATTTTTAGTAGAGATGGGGTTTCATCATGTTGGCCAGGCTGGTCTCGAACTCCTGACCTTAGA
TGATCCACCCGCCTCGTCCTCCCAAAGTGCTGGGATTACAGGTGTGAGCCACCGTGACCAGTTTGGTTTA
GTTTTTTTTTTTTTTTTTTTTTTTTTTTTTTTTTGAGAAATCTCGCTCTGTCGCCCAGGCTAGAGTGCGG
TGACACAATCTCAGCTCACTGCAAGCTCCACCTCCCAGGTTCATGCCATTCTCCTGCCTCAGCCTCCCGA
GTAGCTGGGACTACATGCGCCCGCCACCATGCCCGGCTAATTTTTTTTATGCATTTTAAGTAGAGATGGG
GTTTCACTGTGTTAGCCAGGATTGTCTCAATCTCCCGACCTCTTGATCTGCCCGCCTCGGCTTCCCAAAG
TGCTGGGATTATAGGCATGAGCCACCGCGTCCGGCCTGGTTTGGTATTTTTTTTATGAGTCTGGGTTGTT
TATGAAAACTTGTCACAGCTGTTAACCTTAACTTTTTTTTTTTCTTTTTTTTCCGAGACGGAGTCTCGCT
CTGTCACCTAGGCTGGAGTGCAGTGGTGCGATCTCGGCTCATTGCAACCTCTGCCTCCCAGGTTAAAGCG
ATTTTTCTGCCTCAGCCTCCTGAGTAGCTGGGACTGCAGGCACGCACCATCTCGCCTGGCTAATTTTTGT
ATTTTAGTAGAGATGGGGTTTCACCATATTGGCCAGGCTGGTCTGGAACTTCTGGCCTCAAGTGATCCAC
CTGCCTTGGCCTCCCATGCCTGGCAACCTTAACTTTTTATTTGCTGGTAATTATTTGTGTTTGCATTCAT
GTGAAAATTTGAAATTCTCATTAACATTTAAAGATTCTTACATAGATTGCTTGTAATTTTAACCCTGAAG
TTGTGTCAAGTGACTTTACAATGTCAATTTGTTTTATTTATTTATTTATTTATTTATTTATTTATTTTTG
TGATAGGATCTGGCTCTGTTGCTAAGGCTGGAGTGCAGTGTTGCAAATACGGCTCACTGCAACCTCTGTC
TCCCGGGTTCAAGCCATCCTCCCACCTCAGCCTCCCAAGTAGTTGGAACTACTGGTGCGCCCCACAGTGC
CTGCCTAGTTTTTTTGTATTTTCAGTAGATGTGGAGTTTTGCCATGTTGATCTTGAACTCATGGCCTCGA
GTGATCCACCCCACTTAGGCCTCCTAACATGCTGGTGTTACAGGTGTGAGCCACTGTGTCCAGCCCGAAA
ATGTCAGTTTCGTGCCATGATTAATAGCTAACTACATTTTGGGAATGTAATAAAATTTCATTCTATAATG
AAGTCTTTGTAAAACTCATTAGTTGTGGTATGAGGCTTGTCGGCAATATAAGTGAACGTGGTTTATTTTT
ATTAACTGTATCAGAACTTTAGAATGTTGGTCTCCTGAAACCATTGCCTTGAGAGGCTTTATTGAACAGT
GTTGCCAATGATCAGTTTTTTTTAAATTTCCTTTTTTTTTGAGACTGAGTCTTACCCTGTTGGCCAGGTT
GGAGTATAGTGGTATGGTCATGGCTCACTGCAGCCTCAACATCCTGGGCTCAAGCAGTCCTCCTACCTCA
GTCTCCCGAGTAGCTGGAACTACAGGTGTATGCCACCATGCCTGGCTTTTGTATATTTTGTAGAGACAGG
GTTTAGCCATGTTGCCCAGGCTGGTCTCAAACTCTTAAATTCAAATGATCCACCCACCTAGTTTTCCCAA
AGTGCTTTAATTACATGTGTGAGGCACCGTGGCTGGCCAGGTCAAATATTTTTCATTGACGTTTTTCATA
TTGCTTTTTAAAGTCATGTTAAAATATTCTTAATAATTTTTCTAAGTGGAATTAATCTTGATTATAATTT
TAGTTTTTTATAAAGGGCGGGTTTTGAAACAAGTACTGCATTTTTCTTTTCGGGTTTATAAACATTTGCT
GTGGACTTTGTGCAGTTAACTATTTTCATTCCTGAAACACATTTCGAAATCAGGAATTGAAGACTAAATG
TCTTTTCACTGAAGCTTGAGCAGATTTTAGAAAGGGGAGTTCTTTTTTTTTTTTTTTTTTTTGGTAGAAA
TGGGGGTCTTGTTATGTTGCCCAGGCTGGCCTCCAACTTCTGGGCTTAAACTGTCCTCCTGCTTTAGCCT
CTGGTCTGGAGAGTTCTTTATGGCCTCTTTGAGAACTTTTACTTTACACATGATTCTATCTAGCTTTCTT

TTCTGATGTACATATTGGCAGCAAGTAGAAAAGCAATGTTTTCAGAGGCAGATATATTAACAGCAATGAG
AAATAACAGTAGCGTGATAGAAAGTTGAAAGACTTAGCTGGGTGCGGTGGCTCACGCTTGTAATCCCAGC
ACTTTGGGAGGCCAAGGAGGGTGGATCACTTGAGGTCAGGAGTTCGAGACCAGTCTGGCCAACATGGTGA
AACCCTGTCTCTACTGAAAAACAGAAAAAGGGCCGGGCGTGGTGGCTCACCCCTGTAATCCCAGCACTTT
GGGAGGTTGAGGAGGGCGGATTACAAGGTCAAGAGATTGAGACCATTCTGGCCAACAGGGTGAAACCCCA
TCTCTACTAAAAATACAAAAAAATTAAATGGGCGTGGTGATGTGTGCCTGTAGTCCCAGCTACTCGGGAG
GCTGAGGCAGGAGAATTGCTTGAACCCGGGAGGCAGAGGTTGCAGTGAGCCGAGATCGGGCCACTGCACT
GACGACAGAGGGAGACTCCGTCTAAAAAAAAAAAAAAAAAAAAAAAACCAGACTTGGGGCTGGGCGGGCG
CCTGTAATCCCAGCTACTTGGGAGGCTGAGGCAGGAGAATCGCTTGAACCCGGGAGGTGAAGGTTGCAGT
GAGCTCAGATTGTGCCACTGTGCCCCAGCCTGGGCCACAGAGCAGAGTGAGACTCTGTCTCAAAAAAAAA
AAAAAAGTTTGGAAGACTGGTGGCTGGGCATGGTGGCTCACACCTGTAATCCCAACACTTTGGGAGGCTG
AAGCAGGCAGATTACCTGAGCCCAGGAGTTCAAGTCCAGCCTGGGCAACACAGGGAAACCCCATCTCAAC
AAAAAATATTAATACAAAAAATTTAGCCAGTCATGGTCGTGCACTTCTGTAGTCTCAGCTACTTGGGAGG
CTGAGGCAGGTGGTTCACTTAAGTCTGGATGTCGAGGTGAGCCATGATTGCACCACTGCACTCCAGCCTG
GGCGTTAAAATGAGACCTTATCTCAAAAAAACAAAGCAAAGAGCCTGGGAACTACTAAAATGGGAACTAC
TAAAAAACAGACACAAGAGCTCAACAAGTATACCATTCTGGGAGGTTTTTTTTTTTTTTTTTTTTTTTTT
TTTTGAGATGGAGTTTTGCTCTTGTCACCCAGGCTGGAGTGCAATGGCGCCATCTCTGCTCACTGTAGTT
CCGCCTCCCAGGTTCAAGCAGTTCTCCTGCCTGACTCCTGAGTAGCTGGGAGTACAGATATTGGTCACAC
ACCGGGTTAATTTTTGTATTTTTAGTAGAGACGGGGTTTCCCCATTTTGGCCAGGCTGGTCTCGAACTCC
TGACCTCAGGTGATCCGCCTGCTTCAGCCTCCCAAAGTGCCGGGACCACAGGCGTGAGCCACCGCACCTG
GCTTTTTTTTTTTGACATAGAATCTTGTTCTGTTGCCCAGGCTGGAGTGCAATGGTACAATCTTGGCCCA
CTGCAACCTCTGCCTCCCAGCTTCTAGCGATTTTCCTGCCTCTGACTCCTGAGTAGCTGGGATTACGGGT
GCCCGCCACCACACCCGGATAATTTTTGTATTTTTAGTAGAGATGGGGTTTTGCCATATTGGCCAGGCCG
GTCTTGAACTCCTGACCTCAGATGATCCACCTGCCTAGGCCTCCCAAAGTGCCGGGATTACAGGCGTGAG
CCACCACTCCCGGCCTGGGAGTTTTGACTGTAAGTTTATAGCTGTATATCTTAGGCCCTAAGGGCATTAC
TGTTTTATAGCACAGTGTAGTTAGTTAATGTGCTCATAATGGTGACTCATAACACCAGGTTAAATGATTT
TTTATATCTCCCAAAGAAGTATTTTTCAATCTGCAGATCATGACCCCTTAGTAGATTGTGAAACACATTA
GTGGATTATGACAAGCATTTTTAGAAAAATGAAAAAGAATAAGAAGTGTTAGGATGCATTGCATTATTGA
AATAATTGTTTTTGAGATGGAGTTTCGCTCTTAGTTGCCGAGGCTGGAGTGCAATGGCCCGATCTGCCTC
CCGGGTTCAAGTGATTCTCCTACCTCAGCCTCCTGAGTAGCTGGGATTACAGACATGCTCCACCATGCCT
GGCTAATTTTGTATTTAGTTTTAGTAGAGATGGGGTTTCTCCATGTTGGTCAGGCTGGTCTTGAACTCCT
GACCTCAGGTGATCCACTTGCCTCGGCCTCCCAAAGTGCTGGGGATACAGGCATGAACCCCTGTGCCCGG
CCTAATTTTTGTATTTTTAGTAGAGATGGGGTTTCACCATGTTGGCCAGGATAGTCTTGATCTCTTGACC
TCGTAATCTGCCCACCTCGACTCCCAAAGTGCTGGGATTACAGGTGTGAGCCACTGCACCCAGCTGCCAA
GAATTGTTTTAAGCTTTGGTTTGAGTTAATGTATATATACCGCATTGTAATTCAAAATGTAATTTTTGGC
CAACTCTGGGCACATTGCCTATGGACTAGTCCTGCTCTGCCACGAGCAGCAACAGTTCAATGAATTTTTT
TTTTTTTTTTTTTTTTTTTTTTTTTTTTGAGACAGGGTCTCTGTCACCAAGGCTAGAATGTAGTGGTGCA
GTCTCGGCTCACTGCAACCTCTGTTTCCTGGGCTCAAGCGATCCTCCCACCTCAGCCTCCTGAGTAGCTG
GGAGTACAGGAGCACGCTACCATGCCTGGCTAATTTTTGTATTTTTTGAAGAGATGAGGTTTTGCCATGT

TGTTCAGGCTAGTCTTGAACTCTGGAGCTCAGATGATCCACCCACCTTGGTGTCCAGAAATGCTGGGATT
ACAGGGATGAGCCACCGTGCCTAGCCAAAAATTTTTTTTAAGTAATTTTTTATTGATATAGTCAAAAAA
GTTACTGCTTTAGAGCCAGAGAAACGCAGTAAAAGGATTGAGAAAGAGTTTTGAGGTTATATCTAAGCTA
GGGTTGTCAGATTTGGCAAATAGAAATACAGGACACTCAGTTAAATTTGAATTTTTGATGAACATTGACC
AGTTTTTTAGTATAATTGTGTATTAAATTGCATAGAAAAAAGTTATTTATCTAAAGTTGAAATTTAACTG
AGCATCTTGTATTTTATCTGGCAACTCCAGTCTAAGCTGGAATCATGGTTCACTGTTTTTTTTTTTTTTT
TTTTTTTTTGAGTCGGAGTCTTGCTGTGTTGCCCAGGCTGGAGTGCAATGGTGCGATCTTGGCTCACTGC
AACCTCCACCTCCTGTGTTCAAGTGATTCTCCTGCCTCAGCCTCCTGAATAGTTGGGATTACAGGCACCC
ACCACCATGCCCAGCTAATTTTTATATTTTTAGTAGAGACGGGGTTTTCGCCATGTTGTTCAGGCTGGTC
TTGAACTCCTGACCTCAGGTGGTCCGCCCACCTGGGCCTCCCAACGTGCTGGGATTACAGGCATGATCTA
CCGTGCCTGGCCATGGTTCACTCTTCAGTAACTAAAATTTAAGCTCTATGAAAGCAGGAACTTTGTTTTG
TTCACTATTGATTGTATCCCTATTTCTTGAATGGTTGGCACTTAACTGCTTGGTCACATGTTTGAATGGG
CAAGTTACTCAGCCACTCTCAGGCTTAGTTTATTTACCTATTAAAAGAGAAAGAATATCTTCCTTGGCTG
GGCGCGGTGGCTCACGCCTATAATCCCAGCACTTTGGGAGGCTGAGGCGGGTGGATCACGAGGTCAGGAG
ATCGAGACCAACCTGGGCAACATTGTGAAACCTCATCTCTACTAAAATAGAAAAAATTAGCTGGGCATGG
TGGTGCGCATCTGTAGTCCCAGCTACTCGAGAGGCTGAGGCAGGGGAATCGCTTGAACCCAGGAGGTGGA
GGTTGCAGTGAGCCAAGATTGTGCCACTGCACTCCAGCCTGGGCGACAGAACGAGACTCTGTCTCCAAAA
AAAAAAAAAAAACAAACAAAAAAAAAAAACTGAGATACTGGCCGGGCGCGGTGGCTCGTGCCTGTAATCCC
AGCACTTTGGAAGGCCGAGGCGGGTGGATCACGAGGTCAGGAGATCGAGACCGTCCTGCCTAACATGGGG
AAACCCTGTCTCTACTAAAAATACAAAAAATTAGCCAGGCGTGGTGGCGGGCGCCTGTAATCCCAGCTAC
TTGGGAGGCTGAGGCAGGAGAATGGCGTGAACCCGGGAGGCAGAGCTTGCAGTGAGCGGAGATGGTGCCA
CTGCACTCCAGCCTGCTGGGCGACAGAGCGAGACTCCGTCTCAAACAAACAAACAAACAAACAAAAAAAC
TGAGATACTAAAGTCTTAATATTTTCTGTTTTTATGTATTTATTTTTTGAGATGGGATCTTGCTGTATTG
CCCAGGTTGGAGTACAGTATTGTGATCATGGCTTATTGCAGCCTTTAACTCCTGGGTTCAAGTGATCCTC
CCACCTCAGCCTCCTGAGTAGCTGGGACCACAGGCACATGCAACATCACACCCTGCAGTTCTTTTTTTTT
TTTTGAGACACCGTCTCGCTTTGTCACCCAGGCTGCAGTGCGTGGTGCAATTTCTGCTCACTCTAACCTC
CACCTCCCGAGTTCAAGCAGTTCTGCCTCAGCCTCCTGAGTAGCTTGGGACCACATGTGTGTGCCATCAT
GCCTGGTTAATTTTTTGTATTTTTAGTAGTGACAGGGTCTTACCATGTTGCCCAGGTTGGTCTCAAACTC
CTGAGCTCAAGTGATCTGCCCGCCTTCGCCTCCCAAAGTGTCTGCGCCCTACAATTTAAAAAAATTTTTG
TAGAGACAGTCTCACTGTTACCCGGGCTGGTTTTGAACTTCTGCCCTCAAGTACTCCTCTTGCCTTGGCC
TCCCAAAGTATTGAAATTAAGGCCATGAGGCAGCACACCCAGCCTAAATTCTTCTTATGTTCTGTTCTTG
GCACATAGTAGATGTTCAACAATGTAGAGTCAAACGCATTTGGAGTTGGAATGGCTCTGGTGTTTTTTTT
TTTTTTTTAAACCAGAAACACGTGCAGTTTATTGAATGCCATTGTAGAAAAGTGTGTGAGGATAAACGGC
TGATAGAGAACTTGGCTCTGGGGGCAGGGCGAGGAATGGAGGGTGGATGGAGTACATGGGAATCAGATCA
CGGGCAGAGCTCCTGGCCTAGATAATGCCTCCTGATCTGTTGATAGACTTGAAAGATCAACACTGGGATG
ATGCTGAGCAGAATGGTCGTAATGATGCGCACAATCAGGGCCCAGATGTTCAGGCACTTGGCGGTAAAGG
CATAGGCCTGGGCCCTGATCAGGTCGCCAACCATCTTCTTGTCCCTAGACTTCACGGAGTAGGCCAATGC
TATGAAGCCCAGGCAGCAGGAGTTCATGAAGTGGGTGTTGAACAGGGACCAGACGACATGGTCGGGCACG
GAGTTCTCGCTGTGGATGGGGATCACGGTGGACATTGGGGGAGCAGGGTTGTGGGGTGCCCCCAGCACAG

CCACCTCTTGCTCCTCCTTGAGCATCTCATAGTTAGGGGGATGGCCGATGTTGGCAGGAGTGAAGAGGTT
TGGACATTGTGGTTCATGGTGTCCAGGGAAGACCAGCTGTGGTCGGGTTGCTGGGGTGGTTCTCAGTGGG
CCCCTCCCTTTCCCTGGTAGTTTGGATTTCTCTGGCTCTGGTGGTTTTTTAGTACTCATTCTATTTACGG
GTGAAGAAATTGAGACCAAGAGGGTTATTTACCAGAGTATCTCATCATTGGCTGCATAACTGGCATTAGA
ATCTGATGTACTTTTATTTCTAATACATTTCTTTTTTTTTTTTTTTTTTTGAGATGGAGTCTCGCTCTGT
TGCCGAGCCTAGAGTGCAGTGGGGCAATCTTGGCTCCTTGCAACCTCCACCTCCTGGGTTCAAGCTATTC
CTGTCTCAGCCTCCCAAGTAGCTGGGACTACAGGCACCTGCCACCACAGCCGGCTAGGTTTTGTATTTTA
GTAGAGATGGGGTAGCACCATGTTGGCCAGGCTGGTCTCGAACTCATGACCTCAGGTGATCCACCTGCCT
CGGCCTCCCAGTGCTGGGATTATAGGCATGAGCCACCATGCCTGGCCTTTCTTTGTCGTTTCCTTTCTTT
CTCTTCATCCCTCCTCTCCTTTTTTCCCCTCCCCGCTGCCTCCTCCTGTCTTCCCTTCTTTCCTTCCTTT
CTCTCCTTTTTATTTTTTCCTTTCTTTTTCTTTCTCTGTCTCTCCCAACCCTTCCTCTCTCCCTCCCTCC
CTCCCCTTCTCTCTCCCCCCCTCCCTCCCCTTCTCTCTCCCCCTCCCCTTTTGTTCCTAAGAGACAGGGT
CTCCTTATGTTGCTGAGGCTGACCTTGAACTCCTGAGCCCAGATGATTCTGCCTCCTTAGTAGCTGGGAC
TACACCCACCTCCCGTTCCGTTGTCATCTTTTTTTTTTTTTTCTTTTTTGGAGACAGAATCTTCCTCTGT
TGCTCAGGGTGGAGTGTAGTGGCACGATCATAGCTTACTGTAACTGTGTAACCTCGAATTCTTGGGCTCA
AGCAATCATCCCATCATCCCACCTCAGCTTGCTGAGTACCTGGGGCTACAGGTGTGTACCACCATGTCCG
GCTAATTACTTTTCTTATTTTTAATTTTTCGGAGATAGGATCTTGCTCTGTTGCCCAGGCTGGTGTCAAA
CTCCTGGGCTCAAGTGAAACTCTTGCCTTGGCCTCCCAAAGTGTTGGGAGGGATTACAGGCATGAGCCAC
TGCACCCAGCCTCCTCTTTCTTCCCATTTAACTCCTAACCACACCGAACTTTCTGTCTGCAGAGAGGAGC
ATTGGTCAGCAGTTCACAAAATGGCTAGGTGTGATGGCGTGCACCCATAGTCCCAGCTACTTGGGGAGCT
GAGGTGGGAGGATCGCTGGAGCCCAGGAGTTCAAGGCCCTGGGCAACACAGCAAGACCTTATCTCTGGCT
GGGCCCAGTGGCTCACGCCTGTAATCCCAGCACTTTGGGAGGCTGAGGTGGGTGGATCACCTGAGGTCAG
GAGTTCGAGACCAGCCTGGCCAACATGGTGAGACCCTGTGTCTACTAAAAGTACAAAAATTAGCCAGGCA
CGGTGGCGCGCTCCTGTAATCCCAGCTACTCGGGGGGGCTGAGACAGGAGAATCACTTGAACCCAGGAGG
AGGAGGTTGCAGTGAACCAAGAACACGCCACTGCACTCCAGCCTGGGTGACATAGTGAGACTCTTATCTC
AAAAAAAAAAAAAAAAGGTCGTCTGTACTATTGCATGTTAGTAGTTTCTTTCTGCTTATTGTTGAGTAGT
AGTCTATTGTATGCATGTACCAGTTTGTTCATCTAGTGGTGGACATTGAGTTAGCAGGTTTTGGCTATTA
AAAATAAAGCTGGAGGCCGGGTGCGATGTCTCACGCCTGTAATCCCAGCATTTTGGAAGGCCGAGGCAGG
CGGATCACCTAAGGTTGGGAGTTTGAGACCAGCCTGACCAACATGGAGAAACCCCATCTCTACTAAAAAT
ACAAAATTAGCCAGGCGTGGTGGCGCATGCCTGTAATCCTAGCTACTCAGGAGGCTGAGGCAGGAGAATC
GCTTGAACCCGGGAGGCAGAGGTTGTGGTGAGCCAAGATTGCACCATTGCACTCCAGCCTGGGCATCAAG
AGTGAAACTCCGTCTCAAAAAAATAAATAAATAAAGCTGGTATGAATATTTATGTACAGGTTTTGTGTGA
ACATATGATTTTATTTCTCTTGGTTGGAATGCATAGAAATGAGATTGCTGGGTTTTGTGGCAAGTGTTTA
TTTTTCCAGGGTACATATAATCCTGTGAGTGTTTATTTAATTTTAAAAGTAATTGCTAAACTGTTTGCTA
AAGTGACTGCTATATTTTCTTTCCCTAGCAGTGTATGAATTTTTTTTTGAGGCAGGGTCTTGCTCTGTCA
CCCAGGGTGGAGTGCAGTGGTGCGATATTGTCTGACTGCAACATTGACCTCCTGGGCTCAAGTGATCCTC
CTGCCTCAGCCTCCTGGCTGGGACCACAGGCATGTACCACCACACCTGGTAGTTTGCTTTGATTTTTAGT
AGAGAAGAGGTCTCACTATGTTGCCCTGGGTGGTGTTGAACTCCTGGGCTCAAGTGATTCATCTACCTCA
GCCTCCCAAAGTGCTGGGATTATAGATATGAGCCCCTGTGCCTGGCCTCATTGTGGTTTTAATTTGCATT

TCCCTAATGCCCAGTGATATTGAGCATTTTTTCATGTGTTTATTTGACATTCATACCATCTTTGGTGATG
AGAAACTATGTTTATGCATTGCTTAATGATGGGGATGTGTTTTGAGAAATTTTTTCGGTGATCTTATCAT
TGTACAAATATAGAGTTTACTTACACAAGCCTAGATGGTATACCTACTAGACACATAGGCTGTCGTACAG
AGTATTACTCTTAGGCTACAAATCTGTATAGCATGTTGCGGTACTGAACACTGTTGGCAGATGTAACATA
ATGTTAAGTATTTGTGAATCTAAACATATCTAAACATAGAAAAGGTGAGTAAAAATACAGCGTAAAAGAT
AAAAGTGGTATATCTGAATAGGTCACTTACCATGAATGGAGCTTGCAGGACAGGAAGTTGCTTGGGATGA
GTCATTTATCAGTGGTGTGTGAATGTGCAGGCCTAGGACATTACTGTATGCTACTGTAGACAAACACTGA
ACAGTTAGGATACACTAAATTGATAAATATCTTTCTTATTTTGTTTTTTGAGATGGAGTCTCGCTCTATC
GCCCAGGCTGGAGTGTAGTGGCGTGATGTTGGCTCACTGCAGTCTCTGCCTTCTGGGTTCAAGCGATTCT
CCTGCCTCAACCTCCTGAATAGCTGGGATTACAGGTGCGTGCCACCACACCTGGCTAATTTTTGTATTTT
TAGTAGAGACGGGGGTTTCACCATGTTGGCCAGGCTGGTCTCGAACTCCTGACCTCAGGTGATCCACCCG
CCGTGGCCTCCCAAAGTGCTGGGATTACAGATGTGAGCCACCGCACCTGGCCAGAGATGAGGTCTTGCTG
TATTGCCCAGGGCGTTGAACTCCTGGGCTCCAGCAATCCTCCCACCTCAGCTTCCCACGTAGCTGGGACT
GTGGGTGCACGCCATCATGCCTAGCCGTTTTGTGAACTGTTGACCAATGCTCTTTTCTGCAGACAGAAAG
TTCACTGTGGTTAGGAGTTAAGACTTTTAACCTCTGACCTCAAGTGATCTGCCCACCTTGACCTCCCAAA
GTGCTGGGATTACAGGTGTGAGCCATCACGCCTGGTCAAAAATATCTTTCTTTAAGAGTAAATTTACCTT
AACTTACTGGTTGATCATTGTATATAGGTCTGTTGTTAATTGAAACATGCGGGCCGGGCCCGGTGGCTCA
TGCCTGTAATCCCAGCACTTTGGGAGGCCGAGGCGGGTGGATCACAAGGTCAGGAGATCGAGACCATCCT
GGCTAACACGGTGAAACCCCGTCTCTACTAAAAATACTAAAAATTAACCGGGTGTGGTGGCGGGCGCCTG
TAATCCCAGCTACTCGGGAGGCTGAGGCAGGAGAATGGCGTGAACCCGGGAGGCGGAGCTTGCAGTGAGC
CGAGATCGTGCCACTGCACTCCAGCCTGGGCAACAGAGCGAGACTCTGTCTCAAAATAAATAAATAAATA
AATAAATAATTGAAACATGCGGTGCATGTGTTTATTTGCGATCTGACTTGTTTGGAAATATTTGCATTAT
CTTCCTTCTAGATTTAGAGCATCTTGACAGTAGGAACAAGTGTTTTGTACAACTTTGTATGCTTAGTAAG
TTATCAATTAACTTGTCGTGGCCAGGCGCAGTGGCTCACGACTGTAATCCCAGCACTTTGGGAGGCCGAG
GCGGGCAGATCACCTGAGGTCAGGAGTTCGAGACCAGCGTGGCCAACGTGGTGAAACCCTGGGTTTGTTT
GTTTGTTTATTTATTTATTTATTTTTTGGAGACGGAGTCTCGCTCTGTCGCCCAGGCTGGAGTGCAGTGG
CGTGATCTCGGCTCACTGCAACCTCCGACTCCCAGGTTCATGCCATTCTCCTGCCTCAGCCTCCCAAGTA
GCTGGGACTACAGGAGCCCGCCACCATGCCTGGCTAATTTTTTTATTTTTAGTAGAGATGGGGTTTCGCC
GTGTTATCTGGGATGGTCTCGAACTCCTGACTTTGTGATCCGCCCGCCTCGGCCTCCCAAAGTTCTGGGA
TTACAGGCGTGAGCCACCACACCTGGCCTACCCTGTGTTTATTACAAATACACAAATTGGCCATTTGTGC
GTGGCTCATCTACAGTCTCAGTGACTCAGAAGGCTGAGGCAGGAGAATCTCTTGAACCCGGGAGGCAGAG
GTTGCAGTGAGCAGAGATCGTGCCACTGTACTTCAGCCTGGGTGACAGAGTGAGACTGTGTCTCAAAATA
ATAATAATAATTTGTTGAATATGTGACTGTTGGTTTAATTTTTATTTTTATGAGATGGAGTCTCACTCTG
TTGCCCAGGTTGGAGTACAGTGGCGTGCAGTGGCGCAATCTTAGCTCACTGCAACCTCCGCCTCCTGTGT
TCAGGTGATTCAGCCTCCCAAGTACCTGAGACTACAGACGTGCACTACCGTGCCTGACTAATTTTTGTAT
TTTTAGTAGAAATGGGGTTTCACCATGTTGGTCAGCCTGGTCTCAAACTCCTATTCTCAAGTGATCCGCC
TACCTCGACCTTCCAAAGTGGCGGAATTATAGGTGTGAGCCGTGGTGCCCGGCCAGACTATTGGTTTGGT
TTGGTGTGATGTTATGTTATGTTATGTTATGTTATGTTATGTTATGTTATGTTATGTTATTTTAAGACAG
AGTTTGTCTCTTGTCGCCCAGGCTGGAGTGCAGCGGCATGATCTCGGCTTACTGCAACCTCCGCCTCCCA

GGTTCAAGTGATTCTCCTGTCTTAGCCTCCCAAGTAGCTGGGATTACAGGCGCCCACCACCGTGCCTGGC
TAATTTTTGTATTTTTAGTAGAGACAGGGTTTCACCATCTTGGCCAGGCTGTTCTGGAACTCCTGACCTC
ATGATCCACCCGCCTTGGCCTCCCAAAGTGCTGGGATTACAGGCGTGAGCCACTGCGCCTGGCTGACTAT
TGGTTTTATTATTAAGCAGTAGTAGTTGACCCTGTCATGTAGAAAGCATGGCATTTATAGGCATACCACG
TTTAATTTCCTCCCCTTTTTTTATTTTTGGAGTACCTCCTGCTTGTGAGGCTTGGGAATACAGTAGTGAA
TAAGCCAGATGAGGTCTCTCTCTTTTTGGAGCTTATGTGGTAGTATAGACTAGGCAGAAAGTTCTCATTG
CCCCTGCCACCTTATGGCATTGAGGTGTTTGAGATGCTGATGTTTACTTCTGTCTCATAAAATCTTGAAA
GGAGTTCTTTTAGATGAAGAGGAAAACAAAATCAGAAGAATGGGCCTGGGTCATGTCTGTAAACCTCCCC
ACGTCATGGGGAGGCTGAAATGGGAAGGGCCAGGAGTTCAAGACCAGGCTGAGAAACATAACAAGACCCC
ATCTCTACAAAAAATATTTTTTAATTAATGGGGGATGGCAGCACACACCTGTAGTCGCAGCTACTACGAG
GCTGAAGCGAGAGGATTGCTTGAGCTCAGGAGTTAAAGATTGCAGGAGCTATGATCACAGCACTGCGCTC
CAGCCCCTCTTATCAGCAGTCTGGTATGTTGCTAAGGGTCTTGTTCTTTTTAGTGCTTCAGGGACAGCCA
CTGGCTATGCCCAGAAATAAGTATGTTTGAGAAGCTTTCTGACCTCAGCTTGAAAAATTGATTAGGGTCA
TAATTAAAAAGGGAGGGAAACAGGATTGAGTGAACCGGACGCTACCGTGAGTTTATTCTCCCAGGGCATA
CATAATCTCATGTGATTACCACATAGCCCTGTTAGATAATCTGTTATCCTGTCCTCATTTTACCCATGAG
GAAATGAAGGCCCAGAGAGGTTAAATGACCTATTCAAATTCACTCAGAAGGTGGCAGAGATGAGTTACTA
TCATTGTATTTTGGATCTCTGGAAAGAAAGAAAACTAGTGATGGTATTAAAAAATGTTATTAATAGTTTC
TTTTAATCAACCAGGAACTTGAGTCACTAGCTTCTCTGGGTGAAGGACTATACTTCAACAGTATGAAAAA
CGGAAAAGAAAATGAGGAATTTTGGCTGGGCACAGTGGCTCACACCTGTAATTCTAGCACTTTGGGAAGC
CAAGGGAGGAGGGTCGCTTGAGCTCAGGAATTCAAGATCAGCCTAGGCAACATAGTGAGGCCCCATCTCT
ACAAAAATAAATTAGCTGGGCATGGTGGTGCATGCGTATAGTCTCAGCTACTTGGGAGGCTGACTCAGGA
GGGTCACTTAAACCCAGGAATTGGAGGTTGCAGTGAGCTATGATTGCGCCACTGTATACCATCCCAGGCG
ACAGAGTGAGACCCTATCCCCCCACCGCCAAAAAAAAGAAAAGAAAATGAGGAATTTACATTTGTGACAG
ATACGGAATTCAGGGAATTTAGTTGTTCATAGTCTATAAATGCTATAAGAAGTCTCCATACCTTTTTTTT
TTTTTTTTTTTTTTTTTGGAGACAGAGTCTTGCTCTGTCGCCCAGGCTGGAGTGCAGTGGTGCGATCTTG
GCTCACTACAAGCTCTGCCTCTCGGGTTCACGCCATTCTCCTGCCTCCACCTCCCGAGTAGCTGGGACTA
CAGGTGCCCGCCACCACGCCCGGCTAATTTTTTTGTATTTTTGGTAGAGATGAGGTTTCACTGTGTTAGC
CACAGATCCCGACCTCATGATCTGTCTGCCTCAGCCTCCCAAAGTGCTGGGATTGCAGGCTTGAATCACC
GCACCCGGCCGGAAGTCTCCATACTTTTTAACCCAATCTAAAATGGTAAGGAAATATATAAGAATGTCTA
TTTATTATTAAATTTTTTCTATATAAAACATTTCAGAAAATAAAGACTAGCATTTCTGAGCCAAGTGGTA
GTAGTGGCCATTTTTTCTGGAAAAAAAAAAAAAAAAGAAAGAAAAAACACATTTAGCTATCTATGATGTGA
AAAGATGAACATTTTATTTAGGTAATAAATGTTATGTCATAAAATACCATTTATTGTGTGCCTATTAGGT
TTCAGGAGAGCTGTGCCAAGAGCATTACTTGTATATCTTTTAAGCCTTACAACAGCCCAGCCTGTCAGGC
TGGTAGTGCCATATCTGTTTTACAGATGAGGAAGTGATGGATTGGAGAAATTAAGGAAATTGCCTTTAGG
TCAAAGAGATAGGAAGTGACAAAGCTGAGATTTTTAACCTTGTGAGATTTCAAAGTCTTTGCTTTTTAAT
AACTGTTCCATTGCTTCTAATATAGAGATATGACAAAAACAAGTAAAAATCAGTGAAGAAGGCTGGGAGC
AGTCGCTTATGCCTGTAATCCCAGCAGTTTGGGAGGCCGAGGCGTGTGGATCGCCTGAGGTCAGGAATTT
GAGACCAGCCTGGCCAACATGACAAAACTCCGTCTCTACTAAAAATACAAAAAAGTTAGCCAGGCGTGGT
GACAAGCACCTGTAATCCCAGCTACTCAGTAGGCTGAGGCAAGGAGAATCGCTTGAACCTGGGAGGTGGA

GGTTGCAGTGAGCTGAGATCGCTCCATTGCACTCCAGCGTAGGCAACAAAGCAAGACTCCGTCTCAAAAA
ATAAATAAATAAATAAATAAAAATAATAACAATAATGAAGAAAACAATCCGGTGATTATTGTCAGCAATA
AAATTTCTTCAATCAACCATGCTTTAGTCCTGGCAGTTCTCTATCAGTGAGTTTCAATCAAAAAGTTTGT
TTATAATTTTTTTTTTTTTAAAATTTTGAAATTTGGAAACAACATCATAAATGATGGTTAGTTTTCTGCA
GCTCCCTATTTTGGCAGATAGTCTGTTGTTACTCATAATTAATTTGAACTAAAAAGTAGTGTTGTACGAT
ATCATGGGCTGTGAATGTGTTTGTGACTTGATCTGAGAACCCACACACCACTTAGGATGCTTCTGTAGGA
AAATTAGAGTATGGAACTCACTTGCCCACGCTTTCCCTGTCTCAGTCCATGTTGGTAGGCTGCAAAGTCT
GGGGCTAGAAGGACACTGAACAAGACTTCAGCAGTACATGTTAGTCTTCCAGAGGGAAGGAATATAATAG
TTGAGAGAATAATTCCTTTCCTCTGTGACTTTAGGCAAATTCTTGGCTATGCTGTTATTTATTTGGGCCA
AACAATATCAGGAGGTTGTACATTTTATTCTTAATTACTGCGATACATTAATTTTATCCATGGGTTTAAC
CTAGCCTACCTTTTGCTGTTAGACTTCAACTCTACTTGTGTTGGGTTACCCCTCTGCTTAAAAATCACCC
TATTCCCAAGCCTGAGGGAGTCTACCTTCAAAGCTTTCTATGACCTAATCCAAGGCCTGTCAAACTTCGT
AAAGGGCCAGATAGTAAATTTGTTTTTTTTTTTTGAGATGGAGTTTTGCTCTTGTCACCCAGGCTGGAGT
GCAATGGTGCCATCTTGGCTCACTGCAACCTCTGCCTCCCAGGTTCAAGTGATTCTCCTGCCTCAGCCTC
TCAAGTAGCTGGGGTTATAGGCATGTGCCACCACGCTCGGCTAATTTCTTTGTATTTAGTAGAGATGGGG
GTTTCACCATTTTGGTCAGGCTGGTCTCGAACTCCTGACCTCAGGTGATCCACCTGCTGCGGCCTCCCAA
AGTGCTGGGATTACCAGTGTGAACCACCGTGCCCAGCCCGATAGTAAATATTTTAGGCTTTGCAGTCCAT
ATACAGTCCCATTTTTTTGTGTATGTTTGCACGTTTTCTTTACATATTTTAAAAGCCCCTTTTTTTTTTT
TTTTGAGACAGAGTCTTGCTGTGTTGCTCAGGCTGGAGTGCAGTGGTGCAATCTTGGCTCACTGCAACCT
CTGCCTCCTGGGTTCAGGCGATTCTCCTGTCTCAGCTTCCCGAGTAACTGGGATTACAGGCACATGCTGC
CACGCCCAGCTAATTTTTGTATTTTTAGTAAAGATGGGGTTTCGCCACATTGGCCAGGCTGGTCTCCTGA
TCTCAGGTGATCTGCCCACCTCTGCCTCCCAAAGTGCTGGGATTACAGGTGTGAGCCACCGTGCCTGACC
TAAAAGCTCTTTACAGTGTAAAAAATATTCTGAGCTTTAAGCCATGTGAAAATAGGCCATGGGCATTTGC
TGACCCCTAATAGAACTCCATTTTACCTTTCTGATCATGTTTCCCATTAACTCTTCAAAAATATGACCTC
CATTTAAATCAAGATGGTCTCCTTCCTCACTGCTTGTGGAGGTCCAGTGCCCAGTGTCTGCCTCTTGCTT
GCTCCTCCATCATTGTTCTGCCATTCGAGATCCTCATACTTACCCTTTAAGATCTAGCCCAAATTTTCCA
TGAAACTAATTCTAATAATTAAAAACTTCCTGTAGAACTTAACTTTGTCTAGTACAAGTTAGCTTTCTTA
TTCAGTAGTAGCTTACTATAAATTACAAGAATAAAAAGATTACCATTTTCCCTCACACTGTTTTGTGGAG
AATGCCTAAAGTTACTTTTTCTTTTTACAGGTCAGTATTCCTATTTGGCATCCTAATCCCCTTTCCCAAA
TCTGAATTTTGGGATTTGAAGCTTGCATTTGAGATTATGATTTGTCTTCCTTGTTGTACACAGGAGCAGG
GACTTTACAATTAGTATTCGCATCCCTGCTCCTTCATACTTCGTGATGTAAGGCAAGTTATTTTCACTTA
TGCTTAAGTTTCTTCCCCTGTAAAAAGGGGATGGAAGAGGATTAAATGAATTAAACATGTAACACGCTTA
AAGCAATGCCTGGCAAGTAATAAGTGCTCAGTAACTTTTAGCTGTTCTTATTAGCATGTTTGGAAACCAG
TAGAAACTACACCAGCAAGTTAAGGTTGAAAAGTGGTATTGATGGGCTTGGGGTAGTACAGTATGAATGG
CTACAGTTTAGCGTTTCATTAAGTTTGTATATTCATTAATTCATTACACATTTGATGCTGTCAGACTAGG
ACAGAGACAAAGATGAATGAAACATTATCTCTGCTTCCAGGTTACCCAGTGTAGTAGAGAAGGCAGGCAT
GCAGATAGTTTAAATTGGTAGCACTGGGAGGGGACTGCCATGGGTGGGCAGTGAAGAAAAAGGGCTTCAA
AATAATGAGAGTTGAGATGGATCTTCAAGGAAGATAAGCAGTTTTCAGTAAGGCCATGAAGAGAGGAGGA
AGTTCCAGGCGGGAAGAGTTTGTGCTAAAGTACAGGGATTGCTATACACATGGTGTATGTAGAAAAAATT

TGGTTCACAGTGTGATAGAAGAATTGGAGGGGGTCCTCACTGAAAGTAAGGAAACACATTTGGAAGAATA
TGTTTCAGTTAGAAAATGAAATGAGCTTAAAGTAAACGCTAATAAGGTTTTTAAAATGTAAAATTTCAAC
GTATTTAGAAAGAGAACAGCTGGATGAATCTTATGTACCTGTCACTCAGCTTTAGCAGTTATCAGTAAAT
GGCCAACGTTGTTTCAGCTATACTCCCCTCTCCTCCACTGATAGTCTTTTGAAGGGGAATACAATTGTTT
TGTGGCCTCCAGAAAGGGATAAGTTTATGAGCAACGGGTAGATCGTTGGGAGAGACTTGAGTTTCCTGTC
AGGAAGCATTCTTGGTGCATAAGTCAGAGGTGATATGAATGCCGTGGAAGGGGGTGGCTTACTGTCTGGA
GAACTCGAGAAGATGGGAATGGGCACTGTCCAGTATTGTGGCTACTTCCACACATGGTTCTTTAAATTTA
AAATTATGTTGATTAAAATTTAAATATTTCAGTTCCTCAGCCATACTAATCGTATTTCAAGTGCTTAGCT
GCCACATGTGCCTAATGGCTGCAATATTGGACAGCATGACATAGGACATCTTCATCATTGTACAAAGTTC
TCTTGGACAGCATGGGACTAGAGCCCTAAGATCCTTTTCTACCTGAGTTGTTTGGATTTTTTGGTGTGTC
TAGGTTGGATCTAGTTGTTCATGGCTTCATGACCAAGCCTTTTATCCCTTTCTCTAGAGGGACTCAAGGG
GTAAAGGCACTGAAGGGGTAAAACTTCATATGAAGAGTGTGGTGGTGGTGGTGGTGTTTTAAGACAGGGT
CTCGCTCTGTCACTCAGGCTGGAGTCCAGTGGCATGATCCTGGCTCACTGCAGCCTCGACTTCCTGGGCT
TAAGTGATCCTCCCACCTCAGCTCCCAAGTAACTGGAACTGTAGGCATGAGCCACCACACCTGCCTAATT
TTTAAAACTTTCTGTAGAGACGAGATTTCGCCATGTTGTCCAGGCTGGTCTCAAATTCCTGGACTCACCT
TGGCCTCCCAGAGTGCTGGGATTGCAGGTGTGGGCCACTGCGACTGGCCTTTTTTTTCTCCTTTACTACT
CTAGTGTATGCTGGAATATGAGGAAATAATTATATTAGCTAGCAGTTATTAAACACTTAATAACATACCA
GGCACTGTTTTAAGCTATGCGATCTGTATGGAATATTACTTAATTTCCACAACCTTATGAAAAGATACTA
TTTTTTTTCTTTTGAGAAGGTACTATTTTCATCTTCATTTCATAGATGTTGAAATTGAAACACAGAGAGC
TGAAGTCACAGGATTAAGGCCACAGAGCTGAGAAGTGATGGAGCCGGAATTTGAACCCAAGCAATTAATG
CTGATATTAGTTCTTGTGTGAATGGTAATTGTTTTGAAACAATGATCCTAGATGATTATATGACCGGATT
AATCTGGCAGTTGTTCTGTGTGAATTTAGAGTTGCCTTCCCACCTCAGTTTCCTAAAAACAAAACAAAAC
AAAACAAAACAAAAAAAACTCTAGCTTCACTGTGTTTGGGTTGTCATGGCCTACCCCCTCTTGCCACCTC
ATTTGACTCAACTTTTTAGGGAGAAAATATTCAATACGTGGTATAGGATTTCCCTTTCTAATAATAATGT
AAACAACAACAAGAAGTCTGAAATTGGAAGAACAAAATGACTCACCTAAGTGAGTTAACCTTAAGAGGTG
GAACTTGATTTCTAGATTTTAGTTAATTGTCTAACTGATGTACTAAATATTAGTTACTTAAGTATTAAAA
CGGGTAGACATAATAGTTGGGGAGCTGCTGTAGAGGGGGTAGTTTGAGAAGGCTTCTTTCAGGAGGTGAC
ATTTAAGTTGGTAACTAACAAGAAAGGGGCAGCCATGTGAATAGCTGGAGGGAAGAGCATTCTTACAGTT
TTACTGGAAGGGGGTTAGAGGTATGTGGTACCCTTATGCCAAAGAAAATTAGTTACTTCTATACAACCAG
TCTGATTCTAGAAACCTGGATCAATGAAATATTTTGATTATATAAAAAAATCTGTTACCCAGGTCTTGTT
GAAATAGCATTAGAAACTACTGAAGGACATATAGAGGAGGAGTGTTGAAAAATGGTGATGGATGAGCAGA
ATGGTGAAAAATAAAAAGACATGAAGCTCTATAATTATATTGTATGGTGACAGTACCAATAGAGATTGCA
TGTTTTTTCTCCCCAGTTTTTTTTTTTTGTTTTGTTTTTTGTTTTTGAGACAGAGTCTCACTGTGTCACTC
AGGCTGGAGTGCATTGTCGTGATATTGGCTTACTGCAACCTCTGCTTCCTGGGTTCAAGCGATTCTCCTG
CCTCAGCCTCCTGAGTAGCTGGGATTACAGGCATGTGCCACCACGCCCGGCTAATTTTTGTATTTTTATT
TGAGAGGGGATTTCACCATGTTGGCAAGGCTGGTCTTGAGTTCCTGACCTCAGATAATCCACCTGCCTCA
GCCTCCCAAAGTGCCGGGATTACAGGTGTGAGCCACTGCGCCCGGCCTCCCCCAGTTGTTGAAACAATAA
TGGAAGGTAATTTTATTCTTAGATTATTTAATGTTTTTCAGTTATCAGGATGTGTTAGATTGTTTGTGTA
TATTGTTTTGCTTGTTAATTAAGTAACACAGTGAATAAGACAGACAAACATACGAAAATGTACATTTATT

TTATTTTTTTGAGACAGTCTGTTGCCCAGGCTGGAGTGCAGTGGCCCAATCTCGGCCCACTGCAACCTCT
GCCTCCTGAGTTGAAGCGATTCTCTTGTGTCAGCCTCATGAGTAGCTGGGGCCATGGGTGCACGCCACCA
TACCCGGCTAATTTTTATATTTTTAGTAGAGATGGGGTTTCACCATATTGGCCAGGCTGGTCTCGAATTC
CTGACCTCAGGTGATCTGCCCGCCTTGGTCTCCCAAAGTGCTGGGACTACAGGCATGAGCCACTGTGCCA
GGCCATTTCATTTTTGGAACGTTCTTTTTTTTTTTTGAAATGGGGTCTCGCTCTGTCTCCCAGGCTGGAG
TGCAGTGGCTCAATCTCAGCTTACTGCAACCTCTGCCTTCCGGGTTCAAGTGATTCTCCTGCCTCAGCCT
CCTGAGTATCTGGGACTACAGGTGCATGCCACCACGCCAGGCTAATTTTTGTATTTTTAGTAGAGACGGG
GTTTCACCATATTGGTGAGGCTGGTCTTGAACTCCTGGCTTCGTGATCTGCCCGCCTCAACTTCGCAAAG
TGCTGGGATTACAAGTGTGAGCCACCACGCCCGGCCTGTTTCTGGAATATTCATAATCTTTTGTTGTCAT
TTCAACAGTGCTCACAGCAGCTTCACCAGGTGTAGATTCCATCTTAAGAAACCACTTTCTTTGCTTATCC
ATGAGAAGCAACACCTCATCTATTCAAGTTTTATCATGAGATTGCAGCAATTCAGTTACATCTTCTGACC
CCACTTCTAATTTTAGTTCTCTTGCTTTTTTACCACATCTGCAGTTACTTGCTCTACTGAAGTCCTGAAC
CCCTCAAAATCATTCATGAGTATTAGAAGCAATTTCCTGGTTGGGCACGGTGGCTCATGCCTGTAATCCC
AGTACTTTGGGAGGCCAAGGAGGGCGGATCACCTGAAGTCAGGAGTTCAAGACCAGTCTGGCAAACGTGG
TGAAACCCCGTTTCTACTAAAAATACAAAAATTAGCGGGGATGTGGTGGCGGGCGCCTATAATCCCAGCT
ACTTGGGAGACTGAGGCAGGAGAATCGCTTGAACCTGGGAGGTGGAGGTTGCAGTGAGTTGAGATTGTGC
CCTTGCACTCCAGCCTGGGCAACAGGAGCGAAACTCTATCTTAAAAAAAAAAAAAAAGAAAAGCAATTTC
CTCTAAAACTCCTGTTAATGTTGATGTTTTAACCTCCTCCCATGCTCATGGATGGCATTCTCAGTGGCAT
CTAGAATGGTGAATACTTTTTAGAAAGTTTTCAATTTATTTTGCCATCAGAGAATGGCTATGAATGGCAG
TAGTAGCCTTACAGAATGTATTTCTTTTTTTTTTTTTCTTTTTTTTTGAGATGGAGTTTTTTTTGCTCTT
GTCACCCAGGCTGGAGTGCAGTGGCATGCTATCTCGGCTCACCGCAACCTCCGCCTCCCGGGTTCAAGCA
ATTCTCCTGCCTCAGCCTCCTGAGTAGCTGGGATTACAGGCATGCACCACCATGCCCACCTAATTTTGTA
TTTTTAGTAGAGGCGGGGTTTCTCCATGTTGGTCAGGCTGGTCTTGAACTCCCGATCTCAGGTGATCTGC
CTGCCTCGGCCTTCCGAAGTGTTGAGATTACAGGCGTGAGCCACCGCGCCCGGCCGTATTTCTTAAATAA
AATGGCTTAAACGTCAAAATTATCCCTTGATCCCTGGGCTATGGACTGATTCTTGTGTTAGCAGTTATGA
AAACATTTATGTCCTTGTACATTCCCATCATAGCTTTTTGTCAATGAGAAGTAATTTTTTTTTTTTTTTT
GAGACAGAATCTCACTCTGTTTCCCAGCGTGGAGTGCAGTGGCATGATCTCAGCTCAGTGCATCCTACAA
CTCTGAGGTTCAAGCAATTCTCGTGCCTCAGCTTACTGAGTAGCTGGGATTACAGGCGCCCACCACCACG
TCTGGCTAATTTTTGTATTTTTAGTAGAGATGGGGTTTCACGATGTTGGCCAGGCTGGGCTCGAACTCCT
GGCTTCAAGTGATCCACCTGCCTTGGCCTCCCAAAGTGCTGGGATTGTAGGTGTGAGCCACTATGCCTGG
CCTAATTGGCCTAATTTCAATATTGTTATATCTCAGGGAATAGAGAGGCACGAGGAGAAAGAGAGACAAG
CTGACTGCTGGTTCGTGGAGTAGTCATAACACACAACATTTATTAAGATTGCTGTCTTATATGGACCGTT
TGTGGTGCCTTAAAAGAAATCAGGGTAACATCAACGATTACTGATTACAGATTACTATAACAGATACAAT
AATAATTGTAAATTATTATTTACAATTGTAAAATACAATCTTTTCTTTATTATTTACAATTATTGTAAAA
TACAATCTGATTACAGATTACTATAACGTATACAATAATAGTGGAAAAGTTTGAAAATATTGTGAGATTT
ATGAGAATGTGACACAGGCGCAAAGAGAGCACATGTTACTGGAAATACGGCACTAATGGACTTGCCCGAC
TCGGGGTTTCCACAGACGGTCAGCTTGTCAAAAATGCAGCATCTGTGAATTTCAATAAAGCAAAGCAGAA
TAAAATGAGGTATGCATGTATTGCCATCACATGTACACTAGTAAAATACGTTTTTTTTTTCAGTAGGTGG
ATCAACCTCAAATTTTAATATAAAGCATTACTTAAAGGAGAATATGGGGACATTCATGACATTTCTTATA

TGTACATAAAACTTCATGAAAATAATTTAATGCTATCCAGCAGTTTATTTTAGAAGTACTGGAGGCTAGG
CATGGTGTCTTATGCCTGTAATCCCAGCACTTTGGGAGGCTGAGGTAGGAGGATCACTTGAGTTCAGGAG
CTGGAGACCAGCTTGGGCAATATAGTGCGACCCCATCTCTACAAAAGAGAAAAGAAGTACTGGAGTGTTG
CAGCTCTTACAGAATTTGTCTAGCAGGTTTTCCAGTCTTTACCAGAAATGCCCCCATGCAGAAGTAGTAA
ATACTGATTCATGTAAAATAATAAACAACTTTATCTTTCAGTTTTTAAAAGACAGGGTCTTGTAACGTTG
CCCAGACTGGCCTTTAATTCCTGGGCTCAAGCGATCCTCTCACCTGAGCCTTTTGAGTAGCTGAGACTAC
AGGCTGCACCTCTGCACCTGGCTCTGCTTGATTTTTAATTGTTGTATTGCTGTTGCAGCTATGTTTTTTT
TTTTCTTCAGTGTGAGGATGGGCAAACTTTTTATGTAAAGTCTCAGGTAATAAGTATTTTAGGCTCTAGG
GCCATATAGCTTCTCTGTTGCATATCCTTTTTTTTTTTTTCCATTTCCCCTCAAATTCCTTTTACCATAA
GCAACTCTTGAGGAACATAAAAATCATTCTTAGCCCAGAAGCCAGACCAAAACAGGTTGTGGGCTGTAGT
GTCCTGACCCCTGATTTAAAGATTGATAGCTTTGAAATGGAAAGTTTTAACTTTCTTTTTTTTTCTTTCC
CTTGTTCTGATTGGGCTGTTAATTCATTAGGTATTTACTCAGTGTGTATCATATGAGGCATGATTCCTCT
GCTAATTTTGGTAGTGGTAGAAAGATACTTTTGCCAAGCTTGGTTGTTAGGTTTTCATTTGTCCAAGAGT
TCCTGACCAAGTGTGAATGGATGTTGAAATCAAGGTGTTTCTTTGGCCACACAATGTGCCTTTGGGGGCT
ATATCTATGTGCTTCTGGTACCTTCTTTTAATTTTCACAAAGACACTGCTTGCCGACCACACTGTTTTGT
CTAATGTGGGGCTATGACCCCCTGGAAGAGGCATCATTTTCTGATTTTCACAGAAGCATAATATGGTCAG
GTGATGGTCCTGAGTAGTGGGTATATGACAGATACACTAGTAATTATAATACAGATCTAAACTGGAGAGT
TGAAAACAGCATCGTATATTTGATTGAGATAATCGAAGGAAGACTTCCTGAAAAGATGGCATTTGAGTTT
CAAGGCTGAGTAGGATTAAGTATTATTATTTAAAAAATGCCTTGGACAATGCATTAAATAGAGTTAACAA
ATCACATCACTTATAGTCTCCAATTAAAAACATTTTACTTAAACATAATTTTAGACTTTTAGAAAAATTG
CAAAGATATTATAAAGAATTCTCCTATATATCTCACCTGTATTCTTCAAGTAACATTTTACCATATTCAC
CTTAACATTTTCTCTGTATTGGTAATTGTATATGTAAGATTTAATATAAAATAAAAATTCTTATTAAACA
TATGAGAGACATGATGCCTCTTTAGCCCTAAATACTTCAACTTGTATGTACTAATAACAAGGGCATTCTA
TTTCAAAACCACAGTACAGTTGTCAAAATAAGGAAATTAATAATTGTGTCAAACTGTTATTCTGTTTATA
GACCTTCTAATGTCCTTTAAAACAATCAACAAATCAACATTTTTCTGGTCAAGAACCAGTAAATATGTAT
ATTCTACATATATATATACACATATATATACACACATATATTCTACATATATATGTGGAATATACGTATT
TACTCCCTCTGTCCAAGAACCAATCCAGGATTGTTACCTTCGGTTATCATGTATCTTTGGTCTCCTTTAA
TCCAAAGCAGTTTCTTTGTCTTTTATGACTTGACACTTTTGAAGATTACAGGTTATTTTGTAGACTGTCC
CTCAACTAGGGTTTATCTGAGGTTTCCTTATGATTAGATTCAGATATTTATTTTTGGCAGGAATACAACA
GAAATGATTTGTGTGTTTTTCTCATTGCATGATATCAGAAAGTGCATTGTATATATTTATCCCATTACTG
GGGTTGTTAACTTTGATCACTTGGTTAGAGTTGTGTCTACTAAGTTTCTTCACTATAAAGTTATTTTTCA
CTTGGTCATTTCATCAGTATCTTGTGGGGAGTTACTTTGTGGTTATATAAATACTCTGTTTCTACTTTCC
CTTACTATATTTAGCTTCTGTGGACACTTTTGCCTGAAACAGTTATTTACTATGGTGTTACCAAGTAGTG
ATGCCCTTTTCTTCCATCATTCTGTCTACATTTTTTTTTTTTTTTTTTTTTTTTTGAGATGGAGTTTCGC
TCTTATTGCCCAGGCTGGAGTGCGGTGGCCTGATCTTGGCTCACTGCAACCTCTGCCTCCCGGGTTCAAG
CAGTTCTCCTGCGTCAGCCTCCCGAGTAGCTGGGATTACAGACATGCGCCACCACTCCTGGCTAATTTTG
TATGTTCAGTAGAGACAGGATTTTTCCATGTTGGTCAGGCTGGTCTCCAACTCCCGACCTCAGGTGATCC
ACCCACCTCAGCCTCCCAGAGTGCTAGGATTACAGGCGTGAGCTGCCACACCAGGCCTTCTTTTTCTCTT
TTAAGAGATAGAGTCCTGCTTTGTCACCAAGGCTGGAGTGCAGTGGCATGATGATAGTTCACTGCAGCCT

CAAACTCCTGGGCTCAAGTGAACCTCCCATCTGTAGCTGGGACTACAGGCACCTGCATAACACCTGACTG
TTTTTTAAAACTATTTTAGAGATGGGGTCTTGCGAAGTTGCTCAGGATGGTCTTGAACTCCGGGTCTTAA
GTGGTCCTTCTGCCTCAGCCTCTGGATTAGTTGGCATTACAGGCATGAGCCATTGTACCTGGCAAGTGCA
TATTTTCTTTTTTTTTTTTTAAGGTGGAGTCTCGAGGCCGGGCGCAGTGGCTCACACCTGTAATCCCAGC
ACTTTGGAAGGCCGAGGTGGGTGGATCAAGAGGTCAGGAGATCGAGACCATCCTGGCTAACATGGTGAAA
CCCTGTCTCTACTAAAAATACAAAAAATTAACTGGGCATGGTGGCACACGCCTGTAGTCCCAGCTACTCG
GGAGGCTGAGGCAGGAGAATTGCTTGAACCCAGGAGGTGGAGGTTGCAGTGAGTCAAGATCATGCCACTG
CACTCCAGCCTGAGCGACAGAGGTAGACTCTGTCTCAAAAAAAACAGAAAGACGGAGTCTTGCTCTGTCA
CCCAGGCTGCATTGCAGTGGCATGAACTCCGCCTCCTGAGTTCAAGCAATTCTTGTGCCTCAGCCTCCCA
AGTAGCTGGGATTACAGACATGTGCCACCACACGTGGCTAATTTTTATAGTTTTAGTAGAGGTGGAGTTT
CACCATGTTGGCTAGGCTGGTCTTGAACTCCTGACTTCAGGTGATCCACCCGCCTTGGCCTCTTGAAGTG
GTGGGATTATGAGTGTGAGCCACTGTGCCCAGCCAAGTGAGTATTTGCTTATGTAGTATTTTAATTTTAT
GATTTTTTTTTCTTTGAGACGGAGGTTTGCTCTTGTTGCCCAAGCTGGAGTACAGTGGTGCCATCTCGGC
TCACTGCAGCCTCCACCTCCTGGGTTCAAGCCGTTCTCCTCCCTCAGCCACCTCCTCCTGAATAGTTGGG
ATTATAGGCGCCTGCCACCATGCCTGGCTAATTTTTTGTATATCTAGTAGTGATGGAGTTTGAGCATGTT
GCCAGGCTGGTCTTGAACCTCTGACCTCAGGTGATCCACCTGCCTTGGCCTCCCAAAGTGCTGGGATTAA
GGCATGAGCCACCATGCCCGGCCAGAGACTGTTCATTTATTTTTTTTTTTTGAGGCGGAGTCTCGCTGTA
TTGCCCAGGCTGGAGTGCAGTGGCACAATCTCGGCTCACTGCAAGCTCCGCCTCCCAAGTTCACACCATT
GTCCTGCCTTAGCCTCCTGAGTAGCTGGGACTACAGGTGCCTGCCACCACGCCTGGCTAATTTTGTTTTT
GTATTTTTAGTAGAGATGGGGTTTCAGCCCGCCTTGGCCTCCTGGAGTGCTGGGATTACAGGCGTGAGTC
AGGGCGCCTGGCCAATCATACCTTCTTTTACTGCATTAATTATGGTTTTCTTTCGTTCTTAAAACATGTT
TATAGTGACCACTTTTGAAATTCTTATTAAGTCAGACATCTGGTTATACAAGCAATTTCTATTGCCTACT
TCTTTTTCCAGTGGGTGGGGTTATACTTTCCTGTGTCTTAGCTTGTCGTTTTTTTTTTTGTTGTTGAAAA
CTGGACATTTTAAGTAATGTAGTAACTCTGGATACCTCATTAGCCTATGGTTGGGGGTGGTGGTTGTTAC
TGTTATTTGCTTATTTGTCTAATGACTGGCTGAATGATTTTAGTGTTCTATCCTTCTTCCCTCCCTGTAC
AGTGTGACACGTCTGATGCTAGTTTTCTTGGGATGCAGCCTTGGGTATGCCTACCATCACTCTAGAATCA
CAGTGATTTTGGCATGGCTTTGTCTCTTTTCCTGACTGTACCCAGCTGTTAAGCTACACTAATTACTAGG
TGATGCTGTGTAGTCATTTCTTGGTGTCCTTGGGGGATTGGTCCCAGGACCCCCCCGTTGGATATAAAAA
TTTATGGATGCTCTAGTCCCTCATAAAATGGCACAGTATTTGCATATACCGGTGCACATCCTCCTGTATG
CTTTGTCATTTCTAGATTACTTATAATACCTAATATGGTGTAAACACTAGGTAAATAGTTGTTATATATT
TTTTATTTGTCTTATTTTTATTGTATTTATTTTTAAGTGTTTTTAATCTCGAGTGATTGAATCTGAGGAT
GTGAAATCTGCAGATATGGAGGGCCTGCATTGTTTTCCGTGGAGCTTTGGGCCTAAACTGCTCCACAGAC
TGATCTGATCAAATTTGCGCTTCTTTGAAGGGATAGTTTCTGAGATCAGTGTTTGAAATTTGTTCCAATC
CACAGAGGAGTCCTCCCAGCTCTCTTTCCCTAGTTCTGGCCACCAAACTAGACAACTACAATTTAGCACT
TATCTCCAATGATTCTCCTCCTACCAAGTGCCTTTGAAAGCATCATTAACTCTTTCATACCTTGTTGCAA
ATGAAATTTCTTTGGGAAGAGATTGTGAGTTTTTTTTCTCCTAAATTATGGTGCAATATAAGTAATATAC
CATTTTAACAATTTTAAGTGTATTAAGTGTTTTTTTTTTTTTGTAGTTTTTTTTTTTTTTGTTTTTTGAGAT
AGTCTTGCTCTGTCGCCCAGGCTGGAGTGCAGTGGCACGATCTCGGCTCACTGGAACCTCCACTTCCCGG
GTTCAAGTGATTCTCTGGTCTCAGCCTCCCCAAATATCTGGGATTACAGGTGTGCACCACCACGCCTGGC

TAATTTTTCTATTTTTAGTAGAAACGGGGTTTCACCATATTGGTCAGGCTGGTCTTGAACTTCTGAGCTC
GTGATCCACCCACCTCGGCCTCCCAAAGTGCTGGGATTACAGGCCTTAGCCACCACACCTGGCCTATGCA
TTGCTTTTATATGTATTTTAAAATTCATAAGTTCTCCTCCTATGATGTTTTTGTCCCATGTGATTTATTT
GTTAAACCGTCATCTTTGGCCGGGCGTGGTAGCTCACGCCTGTAATCCCAGCACTTTGGGAGGCTGAGGT
GGGTGGATCACAAGGTTAAGAGATCAAGACCATCCTGGCCAACATGGTGAAACCCCGTCTCTACTAAGAA
TACAAAAATTATCTGGGCATGGTGACGCGTACCTGTAGTCCTAGCTACCTGGGAGGCGGAGGTTGCAGTG
AGCCAAGATCGTGCCACTGCACTCCAGCCTGGCGACAGAGTGAGACTCTGTCTCAGAAAAAAAAAAAAAC
AAAAAACTGTCATTTTTTATGTTGCATTTACTGCATTCTGGATTTAAACTGTGAGGAACCTCATGGTATC
AGTTAATATATTCTTCCATCTTAATGTTTCTCGTAAACTGGTAGATCTGTAAACTTGATTAGGTCTATCC
TATTGTATCACATCAGAAGCAGAAGGTGCTTTTTTTTTTTTTTAAGGGAAATTGTGTGAAAGTAGACAGA
ATGGTAAAGTGAACCCCTGCACACCTATCACCCAGCTTTAATAGTTATCAGCTCATACCATTCTTGTTTG
ATTTACAACCCCATTCATTTCTCCCTTCTGTATTATTATTATTTAGTTAATTATTTTTTGAGACAGGGTT
TTGCTCTGTCACCAATGCTGGAGTGCAGTGGCATAATCACAGCTCACTGCTGTCTTGACCTCCTGGGCTC
AAGTGATCCTCCCACCTCACCCTACCAAGTAGCGGGGACCACAGGCGTGTGCCACCATGCCTGGCTAGTT
TTTTATTTTTTGTAGAAACAGGGTTTTGCTTTGTTGCCCAGACTGATCTCAAACTCCGGCACTCAAGTGA
TCCTCCTGCCTCAGCCTCCTAAAGTGCTGGGATTACAAGCATGAGCTACCACATTCAGCATGTAAATTTC
TTTATATTAATTTGACTGGCATTTTAAGTCACACTTGAATTTCATATTTGGCAACTATTAAAAGCATAGA
GTCCTGGATATTAGTGTTTTGTTAAACCTGATCTATCTAATCATAAATATACTTAGGTCTAAAATATGCT
CTTGGCCTTTGTTTATTGCGGTTCAGTATTTGTTACTATATTAAATAGTAAAATATTTGGTTTGAGATAC
TAATGAAAAGATTAAAAGTAAAGCATAACTTGAATGGATACAAAAAGAAACAAGAATTTAGACTTCAGTG
GATTTCAGAGAATACTGCTTCGATATGCTAACATTCCTGTTGGGTGTCCAACCGTGTCATAGATCAGTGG
AAATTAGTGGTTTCTGCACTTTACTGTACTGTTTTTTTATATGATAATATTTTCCTGGTTGAATGATTCG
TTCTTTTGAGTAAACTCCATGGTCAAACAATTACTTTTTATTAGTCAAAGATGTAACCACATAATCACTA
AAAAGAACAGTGTGACTTATTTAAAGGGGATTATGTTTTTAAGTCTTTTATATAGCTTTGTAGGGAGGCC
ATATGAGTTTAAGGACAGTTCGTGGCATTTGTTCAAGGTTTTGTAACTTGGCATCTCAGCAGCCACCAGG
ATACCAGATCATCGTTCTAAGTAAGATTTAGGCATTTTAGCCTTCATGTACAGACTATAAGTACACCCCC
CCACACCCCTACCAAAACTGTAAATTCAAATGATGTTTGAAAAAGCATAGAATTTTTGTTAGGCGAGGTA
GTTTATTCCTTGTGATACAGTTCCAGAGAGGCAGCATAACCTAGGAATGAAAAACTTAGACGTGGAATCA
GATACACCTGGTTTAAATACCAGCTCTACTGCTCATGAACTGGATGATTTTGGTCAAGATACTTGACTGC
TGAGGTTCAGTTTCCTCACCTGTAAAGTAGAGGTGATAGATTAGACATGTTGCATGTGAAGTACTTAGTA
TGGTGTCTGGTTTTGTAGTAAGATCTATAAAAGATAAATTATTAGTCATATTCCTTAGACTTCAGGAATT
TATCTCTGTGCCATGTTTGAGGCAAACAGTTACAGAATTAGAATGTTAGAAATGAAAGGAATCCTAGATG
TCATTTAATTCAAGTCCATTGTTTTCTGGATGAGAGAAGAAAGTGAGGAAAAGTGACAGAGTTGGAGACC
AAGCTAGGACTGGCCTCAGAATGTTAAGAGTACTCTTCTAGGGATCGACCAGTCGTGTTACTAGACTTTT
TGGATCTGAATTGTGCTTTTCCTTGAATGTTTTGAATTTTGGCTTGAGTGTTGTGATTATTTTATTAAAA
TGAGATTCCAGTCCTATTGTCATGACTAATGTTTATGAGAAATATAACATTTCACTTTAATGATGTTTTT
TAATTATTCTAAGGGGCCTAATCTTTTTCAGTGGAATAAGCTTTAGGTTGTATTATATTCTATAATTCAC
TTGAAAATAGAATTCATCTTTACTTGACAGCCAAATTTGTGTACTGCATCTTTTCTGAGGGAGAGAGTT
GGCAAGGAAAGGCACTTGTTACAACGATCCACACATATAGACGCATATTATTTAGAAATGAAAGTGCTTT

GAATGATTTAGCTTATTTTCAGTTTTTTTTTTTCTGCAGTTGTAATCATATGACCTGTTTTTCTTTCTTT
TTTTTTTTTTGAGACAGAGTCTTGCTCTGTCACCCCGGCTGGAGTACAATGGGGCGGTCTCAGCTCACTG
CAACCTCCACCTCCCAGGTTCAGGCGATTCTTCTGCCTCAGCCTCCCTAGTAGCTGGGACTACAGGCGCA
TGCCACCACACCTGGCTAATTTTTTTATTCTTAGTAGAGATGGGGTTTCACTGTGTTAGCCAGGATGGTC
TCGAACTCCTGACCTTGTGATCTGCCCACCTCTGCCTCCCAAAGTGCTGGGATTACAGGCATGAGCCACT
GCGCCCGGCCCATATGACCTGTTTTTCTTTTATAGATGGGGGAGAAATATGGGAAGTGACTTGGTGTCAG
TCATCTGTGTTGGTTAAATCAAGAATATAATCCGTGTTTTGCTTCTGAATAGCTCTTTATAACAGTGATT
GGTTACTTTGGGAGTAAAGATTATTATTTAGAGACAGAGTCTTGCTTTGTCGCCCAGGCTAGACTGCAGT
GGAATGATCGTAGCCTACTGCAGCCTCAGACTCCTGGACTCTGGTGATCCTGCCTCAGCCTCCTGAGTAG
CTAGGACTAGAGGTGCATGCCACATGCCTGGCTATAATTATTATTAATTTACGTTTAGCATTAGTTTTTT
TCTTCCAGTAGGCTATTTTACTTTATTTATTTGATTTTGATGAAGTTTGATTATTTCTAGTTTGCTTCCT
TCTATGACCCCTACCTGTTGTGGGTCTCCAGGCAAGCAGTGCATAGGTAGAGCCATCCTTAGGTAGCCTT
TAGACTTAATATTAGGTGAGCTCTCCCCACAGATAGCCTCTCCTTTATTTGAATGGAATTATATTTTAAG
TTTGGAAATATTTTTCAGCTTATTTAGCCTGTTGAATTTAATAAAAATAATATTTAATCTTTTCAGAGGT
CGAAACAGTAACAAAGGACTGCCTCAGTCTACGGTGAGTAACTTTAATGTTACTTATTGGGGAAAATTAG
TAGCTAAAACATGATCTCTAACCACAGACCAAATGCCAAGGCAAAAGATTCCCTTCTTTTGAATTTTGTC
ATAGATAACTTGACTGTTTAAGTATGTTATTAGCCTATATGTGTTTTTTTAATGACTCTGTATAAAATGT
ACAATTACTTGTTGTATTAGTCCATTCTTACACTGCTAATAAAGATATACCTAAGACTGGGTAATTTATA
AAGGAAAGAGGTTTAATTGACTCATGCTCTGCATTGCTGGGGAGGCCTCAGGAAACTTACAATCATGGTG
GAAGGGGAAGCAAACACATCCTTCTTCACATAGCGACAGGAGAGAGAAGTGCTGAGCAAAGCAGGGAAAG
CCCCTTATAAAACCATCAGATCTCCTGAGAACTCACTCACTATCATGAGAGCAGCGTAGGGGAAACTGCC
CCCATGATTCAGTTATCTCCACCTGGTCTTGCCCTTGACACACGAGAATTATTATAATTAAAGATAAGAT
TTGGGTGGGGACACAGAACCAAACCATATCATTTGTAAATAGTATTTTTGTCACGTGTAATAACAAGAAC
AAGTCGCTTGTTCTTTTCTAAATGACTAAGTGCAAATCTAAGTGAAAAACCTCCAAAAGATACGTAGAAC
ACCAAGAGTGGAGTCTGCAGAGTTCTTTATGCTTTTTATTTTGAATTAATGTGCTTTTTTTCTGCTGCTT
TCATTTTTCTCCTTTGGCTTTCTGGTCTTAAATTTTGGAATGTTATCAATGAAATTGAACCGGACATGAA
GGGCAGAAACTATAAGTCCCACATGATGGAAGAAATAAATGAGAAGCTATCACAAATTTTTGAGACTTTG
CCTTTATTAGATTGTTTTACAAGAATCAGGAAGATATACACGTATATGGTAGTAATATGGAGTAGTGTGG
TTGATCAGACTTAAGCACTGTCACTGATGCTGATATGCTGGGAGAACCTAGTCAGGGTTCTTCTATGAAG
GTATGACCTGGCTTCCTACCCCATTTATTTATACTTCACCCTTCTTAGGGTACATTTCTGTGAGTTTTAA
CAATTGCATACAATCAGTGTAACTACCACCACAATCAAGTTAATAGAACAGTTTCATTGCCCACCAAAAT
CCCTCAAATCACTTTTCAGTGAACCCTCCTCTCTCTCCAACCATTGATTTGTCTTCCATCCTTACGGTTT
GTGTCCTTCCTCCTCTATGGAAGTTTACTCTTGCTTTTTTATGTCATGTTTAGTCAAAACACCATTAGTT
GGTTTGACTGATAACACTTGAAAACCTGACCTTCTGTTCCTTCTGTTCTCTATGGAAGCAAAATATTAAA
TAAACAAAATCTTCCCTTAATACATGTAAGATATCATAAACCTAACTAAACATTTTGCAACAAATAATAA
ACGTTAGCTTTATATGCAAATGTAAATACAGGCTGAGCATCCCTAATCGGAAATGCTCCAAAATTTCATA
TTTTGAATTAGGGATGTTCAAGCACTAAGTATAATGCAAATATCCCCAAATCCGAAAAAAATCCGCAGTC
TAAAATACTTCTGGTCCCAAGCATTTTAGATGAGGAAGATTCAGTTTGTACTAATTTCTAATAGTTTTTT
TTTTTTTTAATATTCCAGATTTCTTTTGATGGAATCTATGCAAATATGAGGATGGTTCATATACTTACAT

Usually constitutive exon 2, encoding a part of IDR1, with residues GRNSNKGLPQST.
The glycine-arginine motif with lysine and proline is conserved until *Oryzias latipes*.

Usually constitutive exon 3A, encoding IDR1 before LSM domain, with residues ISFDGIYAN

CAGTTGTTGTAAGTTATTAGATTATTGGGGATAAACTGCCTTGGGGGTAGAATAAAGTAATTCCATGAAG
TTAAAATGTGGATAAATGATTGTCAAAGTAACATTGCTTAGATCATGTTTAGTCAGGATGATTTAGAGAA
ATAGATTAGAACTCCTTTTATCCAGTCTAATATAATTCATTGTAAAAGTACAGTTGGTCCTCTGCATCTG
TGGGTTCCATATTCATGGATTCAGCCAACCTTGGATCAAAAATATTTGTTAAAAAGGCCAGGCACAGTGA
CTCACGCCTGTAATCCCAGCACTTTGGGAGTTTGAGGTGGGCAGATGGCTTGAGCTCACAAGTTTAAGAC
CAGCCTGGGCAACATGGCAGAACTCCGTCTCTACAAAAAGTAAAAAAACTAGCCGAACGTGGTGGTACGT
GCCTGTAGTCCTAGTGACTTGGGAGGCTGACGTGGGAGGATTGTTTGAGCCTGGGAGGTGGAGGTTTCAC
TGAGCTGAGATAATGCCCCTGCACTCAGCCTGGTCAACAGTGCCAGACAGACCCCTTCTCAAAAAAAAAA
ATTTTTTTTTTTTTTTTTTTTTTTTTTTTTTGAGAAAAAAGAGGCATGGTTGCGTCTGAACCAAAGAT
GTACGGACGTTTTTCTTGTCATTATTCCTAAAACAATACAGTATGACAATTTACATAGCATTTACATTAT
ATTAGGTATTACAAGTAATTTAGGGATAGTTTAAAGTATTTGGGAGAATGTGCTTAGTTATATGCAAATA
CTATTACATTTTATGTAAGTGACTTAAGTATTATGTAATTCGGTATCTGAAGGAGGTCCTGGAACCAGTC
CCCTACCAATAACAACAGATAGCTGTATTCTTGTTAACCCTGCTGTGTGTGTAAAATAATGTTAGTAGTT
GATTGTCTTTTGTACATTATTTTGTCACTTAAAATAGCTGGGGTCAGAAATGTTTGACTTCAGTATTAAA
ATTCGTACTGCAAACTCTGAGTAGAGCCTCCTGAAGAATTTCAAGAGTTCAGTGTATTGTTAATGTTTTG
AAATTTTTTTATTGTTTTGTTAGTGAATACCTAATATTGAATGAAGCCTGATGAGGTATAAAAAGTAAAA
TGAAAACAAATATCCCTGGTGACCGGGTAGTATACTGTTTCTTTGATAAATAAATTATATGTTTTTAGGG
CTCCAAATGTGAAGTACAAGTGAAAAATGGAGGTATATATGAAGGAGTTTTTAAAACTTACAGTCCGAAG
GTAATTTTTACTTTTTTTCTTTTTCTTACAAAGTAAAAGAACATTTTCATAGTCAGTGTTTTACCTAGTT
TTTAAAGCCACTTTGAATGATTTTACTTCTCAGTTTCAAATACTGATTATTTTATAGACTGGTTTGTGTA
ATCAGAGAGGCTTCTTGATGTGTGTGCTTATTAAAATATTTCAACCATTTTTAAGCATTGTGAGCTAATA
GAGGGATGTGGTGGTTTGTTTTTTCCTCTTAAAAATTATTATTAATGTACTTAAGACAAACCATAGAAAC
AAAAAACATTTAGATATGAGGATTTTTAAATGATGGAATGGATAATAGATCATATGCCTGGGAAAAAGGG
TATGATTCTCTTGAGATTATTTTTGTCAAAGGCATATAAGAACTGGTACCTTGATGAGCTAAAGAATTCC
TAACAAATTTTATTTTGTAAAGGTTTGGAGTACTTACTTGTGTTTTTCATTTTAGTGTGATTTGGTACTT
GATGCCGCACATGAGAAAAGTACAGAATCCAGTTCGGGGCCGAAACGTGAAGAAATAATGGAGAGTATTT
TGTTCAAATGTTCAGACTTTGTTGTGGTACAGTTTAAAGATATGGACTCCAGTTATGCAAAAAGAGGTGG
GTTTTGATTTCCTAAATATGCCTCATGGTTTATTAGATTTATTCAAGCAAAGATTTTCACAGTGATCTTA
CAAACTTTTTTTAAAGAAATATCTGGGCTGGGTATGGCGGCTCATTCCTGTAATCTTAGCACTTAGGGAG
GCTGAGGCGGGTGGATCACCTGAGGTCAGGAGTTCGAGACCAGCCTGGCCAACATGGCGAAACCCCGTCT
CTACTAAAAATACAAAAATTTATTTTTGTGTGTGGTGGCGTGCGCCTATAGTCCTAGCTACTAGGGAGGC
TGAGACAGAATTGCTTGAACCCAGGAGGCAGAGGTTGCAGTGAGCTGATACCGCACCACTGCACTCCAGC
CTGGGTGACAGAGCAAGACTCCGTTTCAAAAAAAAAAAGAAAGAAAAAAGAAATATCTACTTTCTAGAAT
AGCCCAAGTAAGGTAATTTTTTAGAAAAATGAGAATGTTAATGCATTTTTGTTGGAAAACAATTAGAACT
TTAGAGAAAAATTAAATAGAGTTTTTGTGATCTCTTAAAAAATTAGTTTGTAAAGCATTTTCTACAGTTT
TGTGGTCAAGAATGCTACTGATTATATTCAACTGAAAATTTCTTGTCCCATTTGGCCTACAATGCTTTAG
TTTATAAGTGGGCATGTGGCAAATCTGGAAAGAAATCAAAGTATAAGGCTAAGGAAGAAAGGTAGAGAAC
GGTTGGTAGAAAACAATTGTCTAATGAAAATGAAAAAGGGTGAAGAAGTAGAACATACGTATTTTAAAAA
TATTCAGAGTATGAGACAAGGTTTTGAGAATTTAAAAGCGATTATGTAGTTATATTAAAAATTTAGTCTC

Usually constitutive exon 4 encodes the middle part of the LSM domain, with residues GSKCEVQVKNGGIYEGVFKTYSPK
It is spliced out for example in human NP_001297052.1,
chimpanzee XM_016924245.4, XM_054663550.2, XM_054663569.2, XM_063784958.1

Usually constitutive exon 5 encodes the final part of the SM domain, with residues CD**LVL**DAAHEKSTESSSGPKREEIMESILFKCSDF VVVQFKDMDSSYAKR.
LVL is important in the beta3 fold of the LSM domain
(see Figure 2).

TTTTTAAGTGTCCATTGATGAACAAAGTGGGAATTCCTGTTACTCATTTGCAAGGCATTATTGAGTGTTC
AGTAACACGTTGCAAGGCACTTCTGGGCAATCCTGAACTTGGTTCTCAAATTCTTTTTTTTTTTTTTTTT
GAGACGGAGTCTTGTTCTGTCCCCTGGGTGGAGTGCAGTGGCACGATCTCGGCTCACTGCAGCCTCTGCC
TCCCAGGTTCAAGCGATTCTCCTGCCTCAGCCTCCTGAGTAGCTGGGACTACAGGCGTGTGCCACCACAC
CAAGCTAATTTTTGTATTTTTTGTAGAGACAGGGTTTCACCATGTTGGCCAGGATGGTCTCGATTGTTTG
ACCTCGTGATCCGCCCGCCTCGGCCTCCCAAAGTGCTGGAATTACAGGCATGAGCCACTGCACCCAGCCG
GTTCTAAAATTCTTTTATTTATTTGTATATGCCAAATTCTGTAGTGAAATACGTAATTCTGTTGTAAATT
GTAGTTCAGTACAATTTGATTTTCACTATTCAAATCTATACCAAAAGCTGTTTTTATTGTTGGGCTGATT
CTTCTACACTGTTACTTGGAAATAATAATATACCAGGATTCTTTCTCTTAGACTTAGGAGTCTTTCTCTT
TGCTTGCTTTTTCAGAGGCTAACAGTACTGGGTATTCTTTAACTGTCTTGATATGCTGATGAAAGCACAG
TGTTCTGTTTTTGAATCTTCTCAAATGTCCTTGTCTTTGATTCACAACTTTTTGTCTTAAGAGGCCTTCA
GCATCCCATACAAGGAAACAAGTCTTTTTTTAGCTGCTACCTTTGGAGTTGATTTTGTTTATGTCTAGGA
GCACTAAATTATTTATACTTATACTATTGAAATATTCCTCTGTTATAAATTCAAAAATTGACTTTGGAAG
ATAAAATTTAGTTGAATTTAATACATAGCACTCTGGAAAGAGTATTGGCCACAACAAAAAAAAAGGTTC
CCTACTCTATTGGATACCAGGTCATTTAACAGCCATTTACGGTATGCATTGTCTTTTTGTTTTTATGATG
AATTGATATTTCCCAAATGTGGAAGAGTGAATATTACTTTGAGATGTTTGTGATAGTCCATTCCTTGCTC
CTCTTCAAAATTAATGTCATTAAATTTTTATTACTTTATTAGATCTTCATTTCTCAGATAATTTTAGTTC
ATTATAGAAAGGCAAGAAAATACAGATCAGAGTGACAACTTTGAAAATCTCACTCTACTCATAAGGGGAT
GGGTGTATTTTGCTATATATTACAAAATTAGTTTTCTTGATGAGGACATCCACTATTGGAGTAATTTCAG
GTATCTTATTTTTTCTTTTCTCTCTCTTTTTTTTTTTTTTTTTTGGAGACGGAGTTTCGCTCTGTTGCCCA
GGCTGGAGTGCAGTGGCCTGATCTCGGCTCACCGCAACCTCTGCCTCCTGGGTTCAAGCGATTCTCTTGC
CTCAGCCTCCCGAGTAGCTGGTTACTGAGGCATGTGCCACCATGCCCGGCTAATTTTTGTATTTTTAGTA
GAGACGGGGTTTCACTATGTTGGCCAGGCTGGTCTTGAACTCCTGACCTTGTGATCCTCCTGCCTTGGCC
TCCCAGAGTGCTGGGATTATAGGCGTGAGCCACCACGCCTGGGCAGGTATCTTATTTCAAAACTTACAGT
GGTTTAGTGAATTATACAATTGCGTCCAGTGCGTAGTATCCTGAAAATAGTATTAAGTCATGTGTTTAGG
ACATCAGGTCTCTTAAGCTAAGACTATCCAGGCAGAAATTGCCCTCTTCTATAAAAGAAGAAAAGTATTA
ATTAGGAAGTACTATCAGTATGGAGAAAACCATTTTAGAATTATTAATTGGCATGGTTTCCTTCTTTTTT
TTTTTATTTCGAGATGGAGTCTCACTCTATTTCCCAGGCTGGAGTGCAGTGGTGCGATCTCGGCTCACTG
CAACCTCTGCCTCCTGGGTTTAAGCGATTCTCCTGCCTCAGCCTCCCGAGTAGCTGGGATTATAGGCACA
TACCACCATGCCCTGCTAATTTTTTTTTTTGTTTGTATTCTTAGTACAGACTGGGTTTCACCATGTTGGC
CAGGCCGCATGGTTTTCCTTAATAACAAAATTAAGGCATTTATTACTGCATCTAGATTTTTTTTATTTTT
TATTAGAGACTTACTCAGATTACTCCCAAAGTAAAGGAAGGTATGGTTTAATCAATGCTTCTTAATGCTG
GGTTCACGTTTAGTCACCTGGGGAGTTTTTAAAAATGTTCTCACTTCTAGGGATCCTGGTTTAATTATAA
TTAGCCTGGGTGAGGCTCTGGACAGTCAGGGTGTGAGCTATGGGTTTCATGTGATGAGATCCCAGGAGTG
GCTCTGTTCTGTGGCCTTGAGAATTTGTGCTTTCTAGGCCAGGTGCGGTGGCTCACTCCTGTAATCTCAC
TTTGGGAGACCAAGGTGGGCAGATCATTTGAGGTCAGGAGTTCGAGACCAGCCTGGCCAACATGTTGAAA
CCCCGTCTTTACTAAAAAAGTAAAAAATTAGCGGGACGTGATGGCACATGTCTATAATCCCAGCTACTTG
GGGAGAGGCTGAGGCAGAAGAATCGCTTGAACCCGGGAGGCAGAGATTGCGAGATCATGCCACTGCACTC
CAGCCTGGGCAACAGAATAAAAAAAGAATTTGTGCTTTATTTTCTTGCCTCACAGTCCCCTTTCTGTCTC

AGAATTGGCAACTGCCTGAAATAGTCTCTGCTGTTATCATTTGATAGTACTTTTCCACATCTTGAATGGA
TAGATAGAGTGTTTTTTATAATAGAAGTGGATGAATGATTAGAGTATACTAATATGACATTGTATTTTCC
TAAAAGATATGAATTGATTTCATTTCTGAGCTTTTATAATTCTCTTCTGTAATAGTCTGTCAAATTATTA
AGGTTGATAATATTAACTAAAATTTGAGTGCATATTCTATGTGCCAGACTCTGTGCTAACAGATTTACCT
ACATTTGTTCACATAATCATCACAAGTTGTTTCTGTAGTAGATACAGCTATTATCCACGTCATAGATGAG
GAAACAGGCATATTTAGGAAACTTGCTAAAGTGAGGACACAAATCTAGCTTTTCTACTCTAACTCATGTT
CTTAACATTATACTGCAGTGACATAAATTATGTGGTTTGGTTTGTTGTTTATCTCAGTTGTCATAAGTCG
AATTAATGTTTGTTTGTTTGTTTTGAGACAGAGTCTTGCTCTGTCGCCCAGGCTGGGTACAGTGGCGTGA
TCTTGGCGCACTGCAACCTCCACCTCCTGGGTTCAAGCAGTTATCTTGCTTCAGCCTCCCTAATAACTGG
GATTACAGGCACGTACCACCACACCCGGGTAATTTTTGTATTTTTAGTAGAGATGGGGTTTTACCATGTT
GGCCAGGCTGATTTCAAGCTCCTGACCTTAGGTGATCCACCCACCTGGGCCTCCCAAATTGCTGGGATTG
TAGGCATGAACCACTGTGCCCAGCCAGTAAGTTCCATGGTTGTTAAAGGATTTCTCCACAAATAAAGCTA
AAAGTAAAAAAAAAAAAAAAAAAAAAAAATTCTCAAGCAATATAAGATGCAGACTATTATGTTGTTCAAG
TTTTTTTTTTTTTTTTTAATCTTTTGGCTTTATTTTTGGGGAAACCTTTTTTTTCTTTTTTGTTTTCCTT
GGGACGGAGTTTTGCTCTTGTCGCCCAGGCTGGAGTGCAATGGTGCAATCTTCGCTCACTGCAACCTCCG
CCTTCTGGGTTCAAGCGATTCTCCTATCTCAGCCTCCCGAGTAGCTGGGATTACAGGCATGTGCCACCAT
GCCCGGCTAACTTTGTATTTTTAGTAGAGACTGGGTTTCTCCACGTTGGTCAGGCTGGTCTTGAACTCCT
GACCTCAGGTGATCCACCTGCCTAGGCCTCCCAAAGTGCTGGGATCACAAGCGTGAGCCACCGCGCCCAG
CCAGGGAAACCTTTATTTTGAGGCGGAGTCTCGCTCTGTCACCCAGGCTGGAGTGCAGTGGCGTGATCTC
AGCTCACTGCAACCTCTGCTTCCTAGGTTCAAGCAATTCTTCTGCCTAAGCCTCCCGAGGAGCTGGGATT
ATAGGCGTCTGCCACCATGCCCAGCTAATTTTTATATTTTTAGTAGAGACGGGGTTTCACCATATTGGCC
AGGCTCTTCTCAAATTCCTGACCTCATGATCCACCCACCTTGGCCTCCCAAAGTGCTAGGATTACAGGCG
TGAGCCACCACACTCGGCTGCTGGGGAAACCTTTTAACATGAGTAAGGTCAGTGTGACTTTTAAGTTCTT
GATGCTAACATCATTGATTTCAATAAAGTTTAAAAGTTATATTCATGCATATATGCAAATGAATAAAAGG
CTTTGAAATAGTGACTTCTTACGGTACAGTGAATAAGTTTCCTTTGGTCTCTTGAATGTTATACATGTTC
CAGTTTGATTTACTGAGAAACTGAAAGTACCTTTACGTCATATGAGCTGTGAGTCACCTTGGCACATTCA
TAATTAGAAGAGACCATCAGATTATCATTGGAAAATCAGTTTGTATTTATCCTTTATTTGAATTCCAGTG
CAGACAGATCTGAGGTTCTCTTCATTTTGCTAAAACTTCTTAGGGCCTTCAGTCGCTTTTGGCTCTGTAT
TCGTGTATCTTTGGAATTGTCCTGTTATCTCTGCTTGTTTTTTACTTGATTTTCCATCCATTTCCAGTAT
TCCTTTCTCCTCTATTTTTTTCCTTCATTTTCTTTCTGCTCTTCCTGTTGCGCCATTATTCATGTTTTCC
TCTTTACTCCAACTCAACTATGGCTTTACTTCTGTTTCCTTATTCCATTGTTCCTCATACTTTTTCCTAC
TGCTTCATTTTCTTTGCAGTATTCTCAGCCTAGATGATAGGGGTCAGCAAATCTGCTCATCAGTAAATAA
ATTTTATTGTAGCATAGCTATGCCCATGCGTTTGTGCATTGTCTATGGCTGTTTTGATGGCTGTAGCCAT
AGAGTTGAGTAGTTGTAGCTGACTGTAGGACTTGCAAAGCCAGAAAATTTGACTGTCTCTTTACAGAAAA
GTTTGCCAGCTCTTGGCCTAAATCATATTTTCCGCTGCATTTAGGGCTTTTTAGGACTGATCAAAAATAC
ATGCTATACTGGCTTTGGTGAAGTAACAGAATGTGCTCTGTCCTTTAAACTTACAACTAATTGCATGCTT
TGATTCTAATACTGTATAATATCCTGCGATTCTTATTCATGACCATTCTAATTGGATTTAGTCTGAAGAA
TTACTTTTGCTTAACAGATTCTTTGTCACATTTAGTGAAAAATCATAAAAGGGGAAGGTTGGTTAATGGA
AAAGATCTCCATCAACTAACCACTACCTTCCTTATCTACAAATTTATCTTCTTCCTCCGTGCCATCTTTT

TTTTTTTTTTTTTCAGATGATCTTGCTCTGTTGCCCAGGCTGGAGTGCAGTGATGCAATCACAGCTCACT
GCAGCCTCGACTTCCCAGGCTCAGGTGATCCTCTCACCTCAACCTCCTACATAACTGGGACTGTATGTGC
ACATCACTATGCCTGACTAATTTTTTATATTTATATTTTTTGTAGAGATGGGGTTTCCCTGTATTGCACA
GGCTGGTCTCAAACTGCTGGGCCTAAGAGTCTTCCCACCTTGGCCTCCCAAAGTCCTGGGATTACATGAG
TCACCGCACCCGGCCTCATTATTATTTTTCCTCTGGTTTTAGTAGAGAGGATTTTTAAGCCAACTTCAAT
CATGCCCTTGACTCTCTCCCTTCTACTTACCTCCTTGTTCTCTTTTTCTTTTTCTTTTTTTTTAGATGGA
GTCTCGGTCTGTCACCCAGGCTGAAGTGCAGTGGCGTGATTTCAGCTCACTGCAGCCTCAGCCTCCTGAG
TAGCTGGGGCTATAGGTGCCTGCCACCACGCCCGGCTAATTTTTGTATTTTTAGTAGAGATGGGGTTTCA
CCATGTTGGCCAGGCTGGTCTCGAACTCCTGACCTCAAGTGATCACCTGCCTCAGCCTCCCAAAGTGCTG
GGATTACAGGCGTGAGCCACCACGCCTGGCCATCTTTTTTTTTCTCCTTGCTCTTTTATACCACTTCTCT
GTTTCTGGGCTCTTCAACATCTGCCTTTCTAGTTAATCTTTCCCTTTAGCATGAAAACCTATTCACTTCC
TGCTCATCCTAAAAAGGATTCTTTTTTGTTTTGTTTTGTTTTTGTTTTTGAGACAGAGTCTCGCTCTTGC
CCAGGCTGGAGTGCAGTGGCACTATCTTGGCTCACTGCAAGCTCCGCCTCCCGGGTTCACGCCATTCTCC
TGCCTCAGCCTCCCGAGTAGCTGGGACTACAGGCACCTGCCACCACGCCCAGCTAAATTTTTGTATTTTT
AGTAGAGATGGGGTTTCACCGTGTTAGCTAGGATGGTCTCGATCTCCTGACCTTGTGATCCATCTGCCTC
GGCCTCCCAAAGTGCTGGGATTACAGGCATGAGCCACCGCACTGGGCCCAAAAGGATTCTTTTTAATCCT
GAATTCTTCTAGCCATTATCCTGCCTAAGGCTACGATTAACCTCTAACTGCCAGGTCCTTTGGAATCTTT
TTCTGTCTTTATTGCTGCACTTGAATGTTGGTTTCACCCTCCTTCAGAATTTCCTCTTCTGTATTTTTTA
TGTTTATTGATCATTCCTTCCCTGCCTCATTCCTGGGCTTCTTTTCCTTCACACACCCCTTAGATGTGTG
TCCCCAGTGTTTGTTTCTTTGCCTGCTGCTCTTGCCACATGACACACACTGCCAGCTACCACACACAAGT
TCCCTCCTATCATGTGTGTATCATTGCCCTTATACCATGTTGTATTAAAATTATATGCTTGTCTCCCCTG
TTACAGTTTGAGCTCTTTGTGCTCCAAGTAAAGACAGTGATACTGTCTTTATTATTTATTCTCATGGTCT
AGTATAGTGCTTTGGCACATAGTACAGGCTCAATATAAATGTGTTTGAATAAATGAAATTCAGTGCCTTA
ATACACTTTTGTAGAAGCATTATTTTATGGAAAGAATGAAAAAGCTGTAAGTGGTCTTACATATATAGTC
ATCCAGCAGATACTTAGAGAGCTCTGGGATGTGTTCCTTGCTGTGCTTGTTGCTATGGACAGTACGGAGA
AATACAAGAATCTATTTTGGGTCCCTTTTGAGAACCTAGTGAAACTGTGTACCTAGTGAAACTGTATACC
CTCACCCTAGAAAAATTTACACACATGTAGATTTTACATGTAATTCTTTTAAAAATTAATTTTTTTTCTT
TTTTTTAAAGAAACAGGGTCATGCTCTGTCACTCAGGCTGGAATGCAGTGGTGTGATCATGGCTTACTGT
AGCCTCGACCTCCTGGCTCAAGCGACTCTCCCACCTCAGCCTCCCAAGTAGCTGGGGCTACAGGTGCACG
CCGCTATGCCCGGCTAATTTTTAAAAATATTTTATAGACACTGGTTCTCACTATGTTTCCCAGGCTGGCC
TTTACCTCCTGGGTTCAAGCAATCCTCTACCTTGGCCTTCAAAAGTGATGGGATTATAGGTGCAAGCCAC
TGTGCCCACGCTAATGTAATTTCATGGTGTTCACAGTTTCTTCAGGGAGTTCATATACGCCATGTACTCT
ATTCTAAGCATTTTTAGAGTTAGAGATAGCAAAGCACGTGAATAAATTCAAGAAAAAATGGAATGTTGTAC
TGCATGACATTGAATATCAAATGGAGTCAGCGATGCAAATAATTGTCTAGATTTTACAAAAAAAAATTAGC
CTGGTGTGCTGGTGTGCGCCTCTAATCCCAGCTACTCGGGAGGCTGAGACAGGAGAATCATTTGAACCCA
GAAGGTGGAGGTTGCAATGAGCTGAGATCGTACCACTGCACTCCAGCCTGAGTGACAGAGCGAGACTCCA
TCTCAAAAATAAAAAATAAAAGAATTGTGTAGATTTTAGTAGTTGGAAGAAGTTGGAGTGTTAATGTGTA
ATTAGAGAACAGTGAGAAATAAAATTCTACAGATTGTTTTATTCTGGTGTGCTGTTGTGTTCTCATATGG
TTGTCTTTTTGGTCTTGATAGTGTATCAGTAACAGAGTACGAGTAACAAACAGGGATCTCTTCTGAACGG

CGTGACATTAGAAAAGCTGTTTACGGCCTCAACTTTGCTGTGGTTTATTAAGACACAGATATGTGTTCAT
TCTGGGGCCAAGCAGTAACTGGAGAGTGGCACTTATTGAGGCCAGTATGGAGGCAGTACAGAGATTATTG
AGATTAAAAGAAAGAAACAGGTGGAACGGATCTATGTAATGGAAAGCTAAACAGAATAGTTCGTGGTACA
CAGTAGAAAAGCATTACATGTTTATTAAGATATGGTCATCTTCCATTTATTAAAGTTACATGTTTTATAA
TTTTTAGAGTATATAGAAATTCTCTACCCTATCATGTTTGCCAAAGTCAGAACAATAACTTCATTTATTA
AATATAAAAAAAATAAAAACCTCTAGCATAAAATAGAATTTTATTTGGACAAACGATAAAAAAATACTGT
GTGGTACTAGTAAGAGTAAGGTTGATTCAAGATACATGGGAGCAGAATCCAAAGTGTAGAAATAGGCCAG
GTGCAGTGGCTCATGCCTGTAATTTCAACACTTTTGGAGGCTGAGGCGGGAGGATGAGTTCAGGAGTTCA
AGACTCGCCTTGGCAACTTGGCAAAACCCCATCTCTACAAAAAGTACAAAAATTAGCCGGGTGTGGTGGT
GTACTCCTGTAAACCCAGCTACTTGGTGGGCTGAGGTGAGAGGTTCACTTGCAGCCAGTAAGTCAAGGCT
GCAGTGAGCTGTGGTTATGCCACGGCACTCCAGCTGGGTGACAAGCAAGACCTTGTCTCAAAAACAAACC
AGCCAGGCGTGGCGGATCACCTGAGGTAAGGAGTTGGAGACCAGCCTGGCCGACATGGCTCTACTAAAAA
TACAAAAATTAGCTGGGCGAGGTGACGGGCACCTGTAATCCCAGCTACTTGGGAGGCTGAGGCAGGAGAA
TCGCTTGAATCCAGGAGACGGAGTTTGCAATGAGCCGAGATGGTGGTGCTGCACTCCAGCCTGGGTGACA
GAGCCAGACTCTGTCTCAAAAACAAAAATAAGCATAGGACATGGGGATAAATTGAAGATTTATGAAGACA
CAGCTGAAGGAGACATAAAAGTAGATTTGGCTAAATGGAAACATGCCATACTTTGAATGGAATTATTTAA
TACTACAACGTTGTCAATTTTCCTCAAATAAATCTCTAAAGATAATATATTCAGTTTTGGCCGGGCACGT
TGGCTCACGCCTGTAATCCCAGCACTTTGGAAGGCTGAGGTGGGCCGATCACTTGAGGACGGGAGTTTGA
GACCAGCCTGGCCAACATGGTGAAACCCTGTCTCTACTAAAAATACAAAAATCATCTGGACATGGTGGCA
GGTACCAGCTACTTGGGAAGCTGAGGCAGGAGAATTACTCGAACCCCGTAGGTGGAGGTTGCAGTGAGCT
GAGATTGCACTCCAGCCGGGTGACTCCATCTCAAAAAAAAAAAAAATTTTTATAATATATATATATATATC
CGTTTTTGTAGAAATTGACAAAATGATTCTAAAGCTTATTAGATTATGTGTATTAACAGAAGAACTTTGG
AAATTTTTTTCCACAAGAGTCATAAAGGAGGACTTGCCCTACAAAATATGTCAGAATTAAAACATAACTT
GTCAGCTGGGTGCGGTGGCTCACGCCTATAATTCCAGCACTTTGGGAGGCTGAGGCAGGCAGATCATGAC
CAGCCTGACCAACATGGAGAAACCCCGTCTCTACTAAAAATACAAAATTAGCCGGTCATGGTGGCGCATA
CCTGTAGTCCCAGCTACTCGGGAGGCTGAGGCAGGAGAATCGCTTGAACTCGGGAGGTGGAGGTTGCAGT
GAGCCGAGATCGCGCCATTGCACTCCAGCCTGGGCAACAAGAGTAAAACTCTGTTTCAAAAAAAAAAAAA
AAAAAAAAAGAATTATAACTGTCACAGTGGCTACGTATGGAGCATCCAAAACTGAATTTATGTGGGTATT
TTATTAATATGCAATATAGCACTTTAATTCTGGAGGAAAGGTGGATTATTCAGTAAATGATTCTGGGACA
TTGGGGACAAATTAGATACCTACTTCACACTGATAAATAAAACCAAATAGATTAATGAGAAAACTGTGAT
TAAACAAAACAACACCCAGACTACACTGGAGCAAATCTGTGAATTTGTTTAATTTTGAGTGGAGAAGGAC
TTTATAAGCATGACTACCAGAGCAAAAAAATCATGAAGTAAAAGATCGATACCTTTGATTATAAAGAGAT
TAAAGATTTAGGCCGGGTGTGGTGCTCACGCTTGTAATCCCAGCACTTTGGGAGGCCAAAGCGGGTGGAT
CACTTGAGGTCAGGAGTTTGAGACCAACCTGGTCAACCTGGTGAAACCCCATCTCTACTAAAAATACAAA
AAAATTAGTCAGGCATGGTAGCACATGCCTGTAATCCCAGCTACTCAGGAGGCTAAGGCAGGAGAATTGC
TTGAATTTGGGAAGTGGAGGTTGCAGTGAGCCGAGATTGTGCCACATCACTCCAGCTTGGGCGACAGAGT
GACTCCATCTCAAAAAAAAAAAAAAAAAAAGACTTAGACGTGTCCAAAAGTACCATACATTTAAAAAGACA
TGCCACAAACTGGGAAAAGTAGAAAAATAGTTTTAAAAATGACCAGTGAATGTATGAAAAGGTGGCCCTC
CTCACTTGTAATGATTTAAGAAATGCAGTTTATTTTTATTTTATTGTATTTTTAAAGAAATTCAGTTTTA

AAGCAGTGGAATATGATTGTCTATCAGCTTGCGCTGAATGGTAAATGTGAGAAAGATTACTACTACTTAG
TGGTACTGAGGGAGTTGCAAAACACTTAACACTGCTAGTGGGATGGTTTAAGTAAAACAAGTAGCATTCT
TAAACTCTCTATTAGGTAAAGAATAGGTAAGTAATGCATATGTTTCCAGGACATTTTCAGTAAGACTGTT
TACTGATAGGGTTGTGTAATGCTAATATACTTACTATCTAGTTTTAGTATTATTTTTTTCTCTTGTCTTG
GATGGTTTCAATGGAGTCTTATGCATGCAGATATATTAAAACTAGTAATAAAGCAAGAGAAGGAATGTGG
ATAAATTATCTCTAATTTCTATTTTGTTCTATTTCTATTTCATACTCCTGGGAAAGAATATTAAGTGGGC
ATGTGTACTTGAACAGTTGTTCTGTTTTTTATTAGAAAAGAATCCGAATCTATAAAATGTTTTACATATT
TGCCAGGGAAACAGAAAAGATATTTGTACAGCTGTAAGAATTGGAATTAATTTCATTTTACTGACTTTTC
CTTAACCTAATTCTGAACACTTTTGCCATAGGTTTGAGAATAAGTTGTTATAAAATGACTACTATTCTTC
ACTAATAGTATTGGCATTTCAATTCCTAAATTCTGTTTTTTGATTCTTGAACATTTCTGAATTTACTTTT
TTTGTCTTAGTTCTTCTACAGAATCATTTTCTTCTTTTTTCTTTTTTTATTTTTATTTTTTTATTTTTGAG
ACAGAGTCTTGCTCTGTTGCCCAGGCTGGAGTGCAGTAGCGCGATCTCGGCTCACTGCAAGCTCCGCCTC
CCGGGTTCATGCCATTTTCTCCTGCCTCAGCCTCCCGGGTAGCTGGGACTAGAGGTACCCGCCACAGCGC
CCGGCTAATTTTTTGTATTTTTAGTAGAGACGGGGTTTCACCGTGTTAGCCAAGGTGGTCTCAATCTCCT
GACCTCGTGATCCATCCGCCTCGGCCTCCCAAAGTGCTGGGATTACAGGCATGAGCCATCGCACCCGGCC
TTCTTTTTTTCTTTCTCTTTAACTTCTGAGCTGAAAATAGTACCTTTTATAAAGAAGTGCTCAAACGATG
ATTGGACTGATTTCTCCTTATTTCTCTCTTTCTCTCTGTCTCTTTCACTCTCTTTTTAGAATTTTTCTTT
TTTAAGTAGAGACGAGGTCCCACTATGTTGCCCAGGCTGTCTTGAACTCCTGAGCCCAAGCAATCCTCTT
TGCCTCAGCCTCCCAAAGTGCTCGGATTACAGGCTTAAGCTATCACACCAGGCCTAGGCTAATTTCATAT
TTTGAGATGGCACAAATTTCTTTCAGGTAGCTAGCTTTTCCTCCTCCTCCCCACTTAAAATAGATCCTGA
TCCAGAAGCCTAATGGAGAAAATGAAAACAGAATGTTCACCCATAAACAGTATCTTTGTATTGGAATCTT
TTCTAAAACTTCTTTTGATCTTTTTAGGAGATAGTGTGGGAATCAGCAATCTAGTATTACGTACGTGGAA
TCTGTCACCTTGTTTTTTTAAATACAGCAAACCTCATGAAGTGAATTTCCATATTTTTTCTTGTTCTTGT
TAGTTTTGCACCACTCAGGCTTTGCTGTAGAATTTGATGTATATTTGATTCTGTAGAGCATGGGCTATTG
ATCTTCACTCAGCTTTCAGAGGAATCTGATTAGTAAGTTTGAGTTTTTTATTATTTTTTAGTTGATTTTG
AAGTAAAATACAGCACCATTTTAACTGATACCATTTCTAAACAATTTTCAGTTCAAATTTTAAGTTAGCT
AATTTAGAGCTTAAGAAAATTGCTTTAAAAACATAAAATTACTGGCTGGGTACAGTGGCTCATTCCTGTA
ATCTCAGCACTTTGGGAGGCCAAGGCAGATGAATTGCTTGAGCCCAGTAGTTCAAGACCAGCCTGGGCAA
TATGGTGGAACCCCGTTTCTACAAAAAAAATACAAAAAGTAGCCAGACACGGTGGTATGTACCTGTAGTC
CCAGCTATTCGGGTGGCAGAGGTGAGAGGATCATCTGAGCGCAGGGAGATTGAGGCTGCAGTGAGCCAAG
TGAGACCCTGGTTTCAAAAAAAAAAAAGGTTACTAATTGCAGTGCCTTTTATCTTATTTAATGGGCTTAGT
CAAACTAAGATGATGTATTTTATCTTATAAATGTTTTCCCTTGAATTTTAACTGAAGAATCCAATTTGTA
CCTCTCACAAACAGAATGTATTAGTAAGGAAAATAAATACTGCTTTTTATTACTTAAATAGGATATATTT
TTCTCTTAGGGATTTTTTTTCTATTTTATCTCACTTTATCGTAGTGCTAGAAAATTTAATCATTCATTTG
AGATAGGGAGAAAATTAGGTTTTTTTTTTTCTTCTATTTTGAGACAGGGTCTCATTTTGTTGTCCAGGCT
GGAGTGCAGTGGCGCCATCGTAGCTCACCATAACCTCAAACTCATGGGTTCAGGTGATTCACCTTAGCCT
CCTGATTAAGCTGGGACTGCAGATGTGTATCACCACTCCTGGCTAATTTTTGTTGTTATTTTTTGTTTGA
TGAGGTCTCATTATGTTGCCCAGGCTGGTCTCAAACTCTGGGCCTCAAATGATCCTCCTGCCCCAGCCTC
CCAAAGTGCTGGGATTACAGGCATGAACCTCTGCTCCCAGCCCATTTTTTAAAATATATTCACAGCATTG

TGCAACCATCACTACAATCAATTTACATTTTCATCACCCTGAAAAGAAACTCTGAACCCCTTAGCAGTTC
CTCTCTGTTTGTTTCAATTTTCCCCAGCTCCAGGCAACTATTGATTTATTGTCTTCATAGGTTTGCCCAT
TCTGGACATTGCGTATTAATGGAATCATATAATATATAGCCTTTTTTTTTCTTTTTTTTTTTTGAAACAG
AGTCTCACTGTGTCGCCCAGGCTGGAGCGCAGTGGCATGATTGCAGCTCACTGCATCCTCTGCCTCCCAG
GTTGAAGCGATTCTCCTGCCTCAGCCTCTTGAGTAGCTGGGACTATAGGCGCCTGCCACCACACCTACTA
ATTTTATATTTTTAGTAAAGACGGGGTTGCACCATGTTGGCCAGGCTGGTCTCGAATTCCTGACCTCAAG
TGATCTGCCCACCTCGGACTCCCAAAGTGCTGGGATTGCAGCCATGAGCCACCGCATCTGGCCATATATA
TTATGATAGGCTTGTTTCACTTAGTATGTTTCTTCCATGCTGTAGCATGTATTAGTACTTCTTTCTTTTT
CATGGCCAAATATTCCATTATACAGTTACACAGGTACACTACATTTTGTTTATTCATCAGTTGGTGGACA
TTTTCATTGTTTCCACCTTTTGATTTATACATAATCCTGCTGCGAACAGTGACTTTTAAAGTTTTTGTGT
GGGCCGGGTGTGGTGGCTCATGCCTCTGTAATCCCAGCACTTTGGGAGGCTGGGGCTGGCAGATCATTTG
AGGCCGGGAGTTCGAGACCAGCCTGCCCAACATGGTGAAACCCTGTCTCTACTAAAAATACAAAAATGAG
CTGGGTGTGGTGGCGTGCACCTGTAATCTCAGCTACTAGGGAGGCTGAGGCAGAGAATCACTTGAAGCTG
GGAAGCCGAGGCTACAGTGAGCCGAGATCACGCCACTGCACTCCAGCCTGGGTGACAGAGTGAAACTTCA
TCTCAAAAAAAAAAAAAAAAAAAAAAAAACTGCGTGTGGACATAGGTTTTCAATTCTCATGGGGGTGTGTG
TGTATGCATACTCATACATACATACACATACCTGCAAGATAATTGCTGGCTCGTATGCTAAATCTATGTT
GAACCTTTTACATAACTGTTGGGCTGTTTTGTTTTCTTTTTATTATTTTTTGAAAATAGAGTTGGGGTCT
CACTGTTGCACAGGCTGATTTCCTGGGCATAGTGGCTGTATCATTTTACAATCCTACATAGCTGTTTCCA
ACGTAGCTGTATCATTTTACAATCCTACTAGCAGTGTCTGAGGTTTCTTATGTTTTTCACATCCTCACCA
GCATTTGTTATTGTCTGTCTCTTTGATTATACCCATCCTAGTGGGAGAGTAAGAAGTAGTATCTCACTGT
AGATTTTTTTTTTCTGTTTACAACTTTACTTTAAAAATTATATATGCACACATGGTAAAAAGTTCAAAAC
GTGTGTACCAAAAGATTTAACAGTGAAAATAGAAAATAAGTGTGGTCCTTGTTTTCTTCCACCAAGGCAA
ATATTGTTATAATCTCCTAAACAACTTGTCTTCCAGATTTCTCATTTTCAGTCAATCTTGGGCATTGACA
TAAAGAAATTCTTAGACATTGCTTTTATTAGATCATCTCATCCCTTGCTCAAAATCTTCAGTGGCCACTG
TTGTTTACAGAATAAAGTTGGGATGCTATACAGGGCCCTTCCCAGTGGAACTTCTCTTTTTCAACCTTAT
CTCTCATTATTTCCCAATGTTTTTTTTTTTTTTTTTGAGACGGAGTCTCGCTCTGTCGCCCAGGCTGGAG
TGCAGTGGCGGGATCTCGGCTCACTGCAAGCTCCGCCTCCTGGGTTCACGCCATTCTCCTGCCTCAGCCT
CCCAAGTAGCTGGGACTACAGGCGCCCGCCACTACGCCCGGCTAATTTTTTGTATTTTTAGTAGAGACGG
GGTTTCACCGTTTTAGCCGGGATGGTCTCGATCTCCTGACCTCGTGATCCGCCCACCTCGGCCTCCCAAA
GTGCTGGGATTACAGGCGTGAGCCACCGCGCCCGGCCTATTTCCCAATGTTAATCTACTTATTGACCTAC
TAAGCTGGCATGTTCTGTGTGTTAGACATCACCAACTTTGTGCCTTCTTTTTTTGTTTGTTTTTGAGTTG
GAGTCTCACTCTGTTGCCCAGGTTGGAGTGCAGTGGCGCGATCTTGGCTCACCACAACCTCTGCCTCCCG
GGTTCCAGTGATTCTCCTGCCTGAGCCTCCCGAGAAGCTGAGACGACAGGCGCGCGCCACCATGCCCTGC
TAACTTTTGTATTTTTAGTAGAGATGGGTTTCACTGTGTTTCCCAGGCTGGTCTCGAACTCCTGACCTTG
TGATCCACCTGCCTTGGGCTCCCAAATTGCTGGGATTACAGGCGTGAGCCACCGCGGCCCCCTGTGCCTT
CTTCTTTTACTCCTGGATTTAATCCCAACGTGAAGAATCTACCTTACTAACTAGAGTTTTAGATACTTTT
TCAAAACCAAGCCCACATCTGTCCTTTTTAGAGTCTTCTCTGACCTTCCCTGCTCATTGTGGTTTGTTTT
TATTGCCTGTAACAATGGCTGTTAAACTTTACATTTTAAATTAATTTATGTTTGTATGTATTTATTTGTT
GAGAAAGGGTCTCTCTCTGTCACCCCTACTAGAATGCAGTGGCGCCATCATGGCTTACTGCTTCCTGGGC

TCAAGCTGTTCTCCCATTTCAGCCTCCCCATGCACCACCCTACCTGGCTAATTTTTTTGTTTGTTTTTTT
TAGTTTAGTTTTTGTAGAGACAGATGTCTCACTGTGTTGCACAGGCTGATCTTGAACTCCTGGGCTCACT
TGATCCTCCCATCTCAGCCTCCCCAAGTGCTGGGATTACAGGTGTGAGTCACCATGCCCAGACTTTAACA
TTTTCTTTTTAGTATAGAATAGGTCAGTTTTTTTCCCTCTGATGAGATCCCATGCTGACTCTTAGTTAAA
ACAAGGCTTTGGTTGGAAGAAGAGCTAGTGATGTCCTAGCTCCCTACTTACTCCACTTTCCCTTGCCTTC
TGGGGTGTCCTGAAGACATCATAGGGTGTCATGAAGTACAGTTGGAGAACCAGTGGTCTCCATCATGTAC
CAAACACTCATCTTCACGAAGCAGTATGTAGTGTCTTTTTTACCGGTATATTTTCTCTCTCCCAATGCAT
TAAACTTTTCTGGAGTTCAGAAAACAAATTTATAGAATTAAGGAAATGCGTCCCCCCCAACCATGGTGTC
TAGTATATATACAGTGACTTACAGATAACAGGTGTTCAACATATATATATTCCTTTGATTGATTTTTGAA
AAGTTTACATGTATATATTTTTTATATACGGGGTCTCACTCTATCACTGAGGTTGGAGTGTGGTGATGCA
GATCTTGGCTCACCGCAACCTCCTCCTCCCAGGCTCAAGTGATTCTCCCACCTCAGCCTCCCGAGTACCT
GGGACCACAGGTGCGCATCACCATGCCTGGCTAATTTTTTATATTTTTGGTAGAGACAGGATTTTGCCGT
GTTGCCCAGGTTGGTTTCGAACTCCTGAGCTCAGGCAGTCCACCTGCCTTGGCTTCCCAAGTGTGAGCCA
CCACTGAAATACTTATATTTTTAAACTTAATTTATTTATATTTATTATATTTTTATGTTTTTATATTTTA
AAAAATATTTTTATACTCACTAGACCCAATTTTATACTCCTAAACCAGGGAATAACTGTTTTTTTTTCTC
TTACATAGGCATGATACCATAGACAATGATTAAAATTGTAATTACCATTCATTTCTTAGTTTTGTGGCTG
GGACACTGATGTCTTCAAATGTTAGTTTGCAAATACAGTCAGCCCTCTCTATCCATGGGTTACACAGCTG
TGAATTCAACCAACCATGGATCCAAAATATATGGGAAATACGCTGGGGCTGTGGGTCACACCTGTAATTC
CAGCACTTAGGGAGGCTGAGGCAGATGGATCACCTGAGGTCAGGAGTTCAAGACCAGCCTGGCCAACATG
GCAAAACCCTAGCTCTACTATAAGTACAAAAAATTAGCTGGCCATGGTAGTGCACATGTGTAATCCCAGC
TACTCGAGAGGTTGAGACAAGCAATTTGCTTGAACCTGAGAAGTAGAGGTTTCCATGAGCTGAGATTGTG
TCACTGCACTCCAGCCTGCGCAACAGAGTGTGAGAAGAAAAGAAAAAAAACTGTCTGAAAAGAAAAAAAA
AAATTATATGGGAAATCAAAAGCATCTATACTGAACATGTACAGACTTTTTTTCTTGTCATTATTCCTTA
AGCAGTACCACAACTATTTCCGTAGCATTTACTTTGTATTAGGTATTATAGGTAACCTAGAGGTTTAAAG
TATGCGAGAGTATGCAAATACTACACCACTTTGTATCAGGGACTTAAGCATCCCTGGATTTTGGTATCCC
TAGGGGGTATTAGAACCAATCCCCCATAGATGCTGAAGGACAACTGTAGTGTGTGTTGGAATAATTTATT
TTCAAATGGATCATTTGGAGAACACTATTCTTTAGGAAACATAGCCTCCTAAGTTCTGTTCCATACATCC
CTTTCACCTCCACGGCGTTGTAGCATCCTGCTTTCATGACTGTGTCATCACTCGGAAGGAACTGCTTCTC
TTCCAGAATGCTTTTCAAGATCTACTCTGACCACAGCTATAAACTTTACACTTCTATTCTCTTCTTGCCC
CTCACAGTGTTCTCTGTTCCTCTAAGATCTTAAACTCTGTCTACTCCTAATCCAGCCTGCTGGGTGTGGC
TGGAGAAAGTCCCACTGGGGGGCTGATTAGTTAGGAATGTAGGGTTTCCAGCTCTTGCTGGAGCCTCAGA
AGAGTTCAGCAGACTTTTTTTTTTTTTTTTTCCTTAAACCTATTTCTGCAGCCTTGATGACCACTCCTT
CCAGTCCCTCACCTATTTGCTTTATTCATGGCAGAGGCTCTTTCTTCCTGCTTGTCAGTACAAAGAGGCA
GGATTCTTCACCTGGATCTGTGGATTCTCAAAGAATTTGTGGAGAGAATTCAGGGCATTGATGACCTTGG
ATGAAGAGAAATTTACATCTTTATTTACACTAACCTTCAAGTGAAATTTAGCATTTTTTGCCATTTAAAA
ATATGGGCAACAAACAACTAGTAGTATTAGCAGTATTTATGACTTAAGCACCTATAGAACTCAGTTAATT
TCATATCGCTTGATGTTATGGGTATCTCAAATTATTATTTTATGTATATATATTTTTGAGATGGAGTCTC
GCTCTGTCTCCCAGGCTGAGTGCAGTGGTGCAGTCTCAGCCCATTGCAACCTCTGCCTCCTGGGTTCAAA
CGATTCTCCTGCCTCAGCCTCCTGAGTAGCTGGGATTACAGGCGCACACCACCACGCCTAGCTAATGTTT

GTATTTTCAGTAGAGAAGGGGTTTCACCATATTGGCCAGGCTGGTCACCAACTCCTGACCTCAAGTGATC
CGCCTGCCTTGGCTTCCAAAGTGCTGGGATTACAGGTGTGAGCCACCGCACCCGGCCTCAAATTATTTTT
AGAAACAGAATCTTGATATGGTATCCGCTCTGGCCTTGAACTTGTGGGCTCAGGCAGTCCTCCCACCTCA
GCCTCCTGAGTAGCTGGGATTATAGGCATGTGCCACTGCACCAGGCTTCAAATTATTATGTATGTTCATC
ACCTCTTTAAATTTATAATAGTTATTAAACCTGTTACTGGATCTTAATATTTAATGCTTTAATTAAGAAC
ATGTATGTTACTATGCCAACAGATTTTTTTAGTTTTTGATAACTGCATTTCATTGTTACTTGTTCTCATT
TGATTTCCTGTGTATTTTACGAATTTAAGTACATTCTGAATACGGTTTCATAGGCTTCCCTAAAATATTG
AAGGGGCCCATGGATTAAGAAAAAGGCTAAGAATCCCTAATCTAGAGGCTCCCCACAGTCCTCTTTTGTC
ATCATACCCCTACCCCATTCTAGCCTGAGGAGCGTGGCTCCACCTGTGCCCTTGGTTTTGTTGTTCCAGT
CCATACATCCTGCACCCTTAACTGTGTTTCTTATCCCCAACTTGTTTCTTTGTGTTATTCTTCAGTATTA
TAGTCTTTAATATAATCTGTATAATACATGGTGTAGTAGTATATGCTCGTAGTATACAATTCAGTTAGAA
CAGATGAGTATTCAATGAAAAGATAATCTCCTCTCTAACCCCCAGTCCCACTTCCCTGGGGAAGCCTGTG
TTCTTGTGTACAATTCAGAAAATGTTTATACACATATTTTTTATTTATTTATTTTTTGAGACGGAGTCTC
GCTCTCGCCAGGTTGGAGTGCAGTGGCGCAATCTTGGCTCACTACAACCTCCGCCTCCCTAGTAGTTCAA
GCAATTCAAGGTTCAAGCAATTCGCCTGCCTCAGCCTCCCGAGTAGCTGGGACTATAGGCGTGTACCACC
ACGCCTACCTAATTTTTGTATTTTTAGTAGAGACAGGGTTTCACCATGTTGGCCAGGATGGTCTCGATCT
CTTGACCTCATGATCCACCCGCCTCAGCCTCCCAAAGTGCTGGGATTACAGATGTGAGCCACTGTGCCCA
GCCTGTTGATTTAATTTTAAACAGAGTTTCGCTCTTGTTACCCAGGCTGGAGTGCAATGGTGCGATCTCG
GCTCACCGCAGCCTCTGCCTCCCAGGTTCAAGTGATTCTCCTGCTTCAGCCTCCCGAGCAGCTGGGATTA
CAGGCATGCACCACCATGCACAGCTATATTTAGTAGAGATGGGGGTTTCTCCATGTTGGTCAGGCTGGTC
TCGAACTCCGGACCTCAGGTGATCCGCCCGCCTCGGCCTCCCAAAGTGATGGGATTACAGGCGTCAGCCA
CTGCACCCCGCCTATACACATTTTTTTGTTTTTTGTTTTTTTGAGATGGAGTCTCGCTCTGTTGTCCAGG
CTGGAGTGCAGTGGCGCGATCTCTGCTCACTGCAAGCTCTGCCTCCCTGGTTCACACCATTCTCCTGCCT
CAGCCTCCCGAGTAGCTGGGATTACAGGCGCCGGCCACTACGCCCATCTAACTTTTTGTATTTTTAGTAG
AGATGGGGTTTCACCGTGTTAACCAGGATGGTCTTGATCTCCTGACCTCGTGATCTGCCTGACTGGGCCT
CCCAAAATGCTGAGATTACAGGCGTGAGCCACCGCTCCCAGCTATACACGTATTTTTAATGCCACTCCAG
TCTATGTTGGAACCATTTTACTTCCCCTTTCTTATTTTCTTCTTGTGTTCTTGAAGGCCTAGATCAGCTG
TTGCTGATAGGCTGTCACTGTCACTTTAGAAAGCCCAGAGCCTTTTGTTCCTTAGAACTTTGTTTTTAAT
TGTATTGTAGCACTCATTGTATTCGATTCTAAAAGATTTGCTTCATTTCTGTAACTAGTCTCTTACACCC
AGGAGCTCCTAGTTCCTACAGGAAATGCTGGGAATTGTATCAGTCAAATGTGAATCCCCACCTCGTCCAG
ACTTATGAGTGCATTGTAGGTACTCAGTAAGTGCTAAAAATGACTAAATAGTCCCACTGATACCAATCTA
TATACTGATACTTTATATAGTATATAGATTGGTCCACATATAACGATGACACATAATGAGAAACTGTCTT
AAAAAGTTGTTGAAAGTGCCGCAGGAATAGGAATTGATCAAAACAATATGATTTTTTAGGTTTATATGGA
ACTTTGATGTTTGAGAAAAGGCTGATTTAGTTGAGAAGAAATGGTTAGCTGAGGATTTTGATGACTTCTC
TGGAAGCACATTTGAGGGTTTGTGATGTTAAATCTGATGTTAATGATTATTTCATCCAGTTTTATGTCAT
TTTATAGTTTTTATACATTTAAGTATATTTATTTCTAATGTTTAACACTACCATTTTAGTTATTTGACCA
TTATTCTGGCCCTTTAAAAAATGCTCAGACAAGTTTGAATGATTTTTCAGAGGCATTATTGGCTCAGAGG
TAAAAGAGGAAAGATTGAGAAGCTGAATATGTACTCTGTTTCCTGGGTATGGGGCTGGGGATACCCAGAA
GAGGTTCACACGTTGGTCGAGACATTTCTTTATGACCACCAGCAGGTGGCATCACCGGCCCAAAATGACT

AAGTTTCTGCCCAGAATCAGAAGAGAAGGTGTTGAGAGCCCACTGCTGTGGGGGTAGCATGGAGGTGGGA
TACAGGGGCTGGAGGTGATACAATTTTGTTTCTTCCTCCAACATCGCCTGCTAGTCTAGAGGCTTTTATA
AATTGAAAAACTAATTCTTTATCATCTCATCTGATGGTTTTTATGTTTTTCCTTTTTTCTCTCTATACCT
GTAGTTCCTTCAGAAACAGGTAACACTTTTCTAATAGTCACGTTGTATTCTTGCATCTTGTTGTTACAAT
GCTTTTGTTTCTCACCATAGGGGATGATGGAAAATTAATATTCTTTGACTTATGGCATTGGTAAAATCTG
CATGCAAATTCCCACAGTTGCCTGTAGATTAGAGCCAGTTGTTTTTTTCTCAACTTTGCAGGAATCCTGG
TTACAACATTGTACTATTTACTACCAACAGTGTTTTTTTTTTTAAAATCCAGACTTGCTGGGCATAGTG
GCTCATGCCTGTAATCTCAGCGACTTGGGAGGCTGAGGTGGGAGGATTGCTTGAGCCCAGGGCTGCAGTG
ATTGCGGCACTACACTCCAGCATGAGTGACAAAGACCCCATCTCTGAAAAAACAAAAACAAAAACAAATT
TTTTTTAAAGAAACAGAAACAAAAATCCAAACTTGTAACCACTGTAAAACAAATCAGAATTTACGATAGT
GGATATTATTAATAGTGCAGAATGGATACCCAGATCTTGCTTCCTTTCTAGCTAATGATGCAATGTTGGC
CTGAAATGCATTACTTATAGCCAGGGATTTTCTCAGCATCCTGATGATATAGCCTCATTTCGTGCTAACT
CTCCACTTCTGCACATCTTCCCCTAAGTCCTTTACTCATCTTTAGAAAGAGCTACTTTTGGTGAAATTTT
AAAACCAAGGAATATCATTCTTTATAGAATCACACTTCTGTGTTTTCCCCTTCCCCATTTCTGTCTCGAA
AGCGACAGACTGCTACATAACCTGTGAATACTTTTTTTAAAAAAAGTTTGGTATTGTAAACAGAAGATT
TAAGATTAAAATGTAGCATTGAGAAAAATAGATTTATTAATAATGCCCTCTTAACACAACCTAAATTCTG
GTCAGTGGAATAAAGCCTGGGTCCTAAAGTTTTAGACGCTTGCTTGCTTTTCCACACTGGCTCTTACTTG
GGGATCCTTTTAGAAATTTGTTTAGAATAATACTGTAAAAACATATTTAAGCTACTTTGTGTGTACATTT
GGGATCTTTTGGTTTGAAGACGGCTTGACTCAAGACTTTCTAAATATTTTCACACACACACACATACCCT
GTAGTGAGAAAAAAATCCGTTTATATGGTTCTATAAAAATCTCTAGCTGCTTCGAGCTTTAATTTCTTGA
ATCAAAAGAGTATTGTTTTTAATACTGAGCTTCTATCTAAATAAATGCTTTATTTACTTAAATGTGTGCT
TTTCAAAAACTAGTATGATTAAGACATTAACAGGATCTTAGACGTAAAGGAACAGTCCTGTTGCTTCTTC
CAGAAGATAATATGACTCGTTTGGAATTTTCCTATAGTGTAGTTTTTTGTCTAGTGTTGTGAGAATTAAA
GGGATTTCAGGATCTTAAGGTAGGTTATTATTTGATGTTTTCTTGGAACATTTTACATTCTTGAAAATAC
ACATGGCTAAATTAATTTTTGCCAGCAATCCACATAACTTTAAGATAATGTAGAGAAGAACGTGATTCAG
GTTAGTATCAAATAAGGTCAGATTTCTAGTGCCATCAGTAGCTTTCAGCAAAGATGAGGTGTTGGTAAGA
TAGCATTAGTCTCTTAGAATCTCTTAGAGAGATTTTCCAAAATTCAGCCATTTCTAGTGAATGCTCCATT
CCACCCCCAGCTGAGTCCTGCTGCTCTGGGGAACTCCCTCAGCACACTCTTGGCTCTTAGAATTGCTAGC
AATGGGAGTAGTGCTGCTGGTGGAGCTGGCAGCTAAGCCCAGAGGTGGATTAATGCTTTTATTCCCTGAT
GTACAGGTACACACACTCATACCTACCCACACCTAGTTTGGGATAAGAAGAGGTTAGAATTAGCTAGGCT
TGAAGTTCCATGCTTAAATTTGCTGGCTCAGATTTCTTATTTTGGCATCACTTTGCCCATTAGGGAGACA
ATGACAGTTATAGAAGCATTGCCAAATAAAAAATCCATCTGGAATAACCTCTTTTGTAGGAGTATTGTGT
GTTTAGTTGTTGATTCGTCCCTTCCTCCTCTTAGTGGCAACTTACAGTACTGGGAAGGAACAGTGGCTGG
GAGCTTATATTCCTCAGCAGAGCCAGATCAGCAGAAGTATTACTCCTTAGTTCGTAGTAGGTGGTACCCT
ATGGGTCCAGTCATTTAAATGCAAGCCTGTATCTACAGAGCGTTTCCTAGTGCCATCATTGCCCAGTGGG
CCTTTATTTAGCTGAGTCTAACTCCCAACTAGAGAAAATTTCCTGTGCCAGACAGCAGTATGGTCAGCTA
ACATGTGGATGCTACATTTGCTTTCATAAGTCAGTACTCTTCAATAACATTAGTAGAAGAGAAGAGGACA
CAAAGTGAGAGTGTGTTAATAGGAAGTCCAGGTATGCCTGCTACCTGAACTTTCTGAGACAGGTAATACT
GTAGGGCCTGAACTTTGTAGCAGAGTGGTTATATATGAAGAAGTGGGTTCTGGGAGGGGTTAAACCACTT

AGAATGGCTTCATTTACTAATGGCAAGAGTTTGCTGGGATATTGACCACTGTACATAGACATGAATATGG
AAAGTTAAAAACAAAATCCACATATATTTGGCTGCAAGTACTCCGAAGGTATATCTAATTAGTGCATCCA
TTAAACAAAAGAGATATTTTAGGCCGGGCATGGTTGCTCACACCTGTAATCCCAGCACTTTGGGAGGCCA
AGGTGGGTGGATCACCTGAGGTCAGGAGTTCGAGACCAGCCTGGCCAACATGGTGAAACCCTGTCTCTGC
TAAAAATACAAACATTAGCTGGGCGTGTTGGTGGGCGCCTGTAATCTTAGCTACTTGGGAGGCTGAGGCA
GGAGATTCCCTTGAACCTGGAAGGTGGATGTTGCAGGGAGCCGAGATGGTGTCACTGCACTCCGGTCTGG
GTGAAAGAGCAAGCTCCATCTCAAAAAAGAAAAAAAAAAAAAGAGATATTTTTGATGGATTGATAGAAAT
TTTCTTTTTCTTTTTTTTTTTGAGACAGGGTCTCACTCTGTCGCCAGGCTGGAGCACAGTGGCGTGATCT
CCATTCATTGCAACCTCCACCTCCCGGGTTCAAACGATTCTCCTTCCTCAGCCTCCCGAGTAGCTGGGAC
TACAGGCATGTGCCACCATGCCCAACTAATTTTTGTATTTTTAGTAGAGAGAGGGTTTCACCATGTTGGC
CAGGATGGTCTCGATCTCTTAACCTCATGATCCACCTGCCTGGGCCTCCCAAAGTGCTGGTATTACAGGC
ATGAGCCACCACATCTGGCCAGAAATTTTCTTGGTCACTTCTGAGACATGCAGAGTAATTACCTGTAATA
TAATTTAATGAATTATGTCAATATATTAAAATATGCTTCATGTGGGCTGGGCATGGTGGCTCATGCCTGT
AATCCCAGCACTTTGGGAGGCCAAGGTGGGGGTATCACTAGGTCAGGAGATCAAGACCAGCCTGGCTAAC
ACGGTGAAACCCCGTCTACTAAAAATACAAAAAATTATCCGGGCGTGGTGGTACACACCTGTAGTCCCAG
CTACTCGGGAGACTGAGGCAGGAGAATCGCTTGAACCCGGGAGGCAGAGGTTGCAGTGAGCCGAGATCAC
GCCACTGCATTCCAGCCTGGGCAACAGAACGAGACTCTATCTCAAAAAAAAAAAAAAAATGCTTCGTGTG
GCTTAAAATTATATGAAAAGAAAATACCTTTACTGATAGTCATCTGTGATTCCATTTGCTAAATTAAACG
TGAAAGCATACTTTTACTGAATACTATATATTCCGTATCAGTTTAGATAGCAGTTTATCTTCACATACAT
AAGTTTTAAGTTTACCTTTATTATAGTGCATTGGTCTTTTGTTTTCATCAACCTAAATTATGTTCAATAA
ATGTTTCTGTTAGATTTTAAGTTAAACAATTATGTGAAATTCATTTTTCGTAATTGTTTTTTAACATATG
TCTTTGTTGGTAATTCACGTGTGTGAGTGTAACTGATTGCCAGATTATATAAACTTTCAACCAAAACCAT
TCTTTGCAGATGCTTTTACTGACTCTGCTATCAGTGCTAAAGTGAATGGCGAACACAAAGAGAAGGACCT
GGAGCCCTGGGATGCAGGTGAACTCACAGCCAATGAGGAACTTGAGGCTTTGGAAAATGACGTAGTAAGT
AACATCTTTGTAATTATTGCTAGACTCTGGTCAGTATGACATCCTGTCACTTGGTTGTAATTTAAATGTG
CTTTTGTTGTTGTTGTTATTGTAGTGAGTGTATTTAGAGCAGCAGGTTTGTTGTATAACTAGAGACTTTC
TCCCAAGCAATATATAAAGAAAAATGTTTGTCATTTTACTTGTAGGGGTTAAGCAGGAGTACTGTCTGTT
CTTGTGGATGCTCATGAATTACTTCTTTGTGATTAAAATAAATAATAAGAAGTAGCTTAAATTAAAATTA
GAAACCATGGGAAATGCCGGTGTGTTTTGCTTTAACACCCAGCCAAATAAGGTAGCCTAAGGAAAGTGGT
GTCTTAATTGTTGACTTCACCTAGAGAAGAGGTTGAAGTAGGACATTTTAAGCCTCTTGTCTGAAGAAAA
GGTTGTCATTAAGATAAATAATTAGGTTACATTGGAATTAAAGCATTACATAAATTTCTTGGTCTTAAAT
TTGGATTATTCTCCACAAAATTCTTTTATTTCTAAAACGCCTCTTGTCACATACTAGTTTTGTTTCTCTC
TTTAATGCATTATCTGTACTTGAAGTGCTTAGCTGGGTATGCTGGCACATGCCTGCAGTCCCAGCTACTT
GGGAGGCTGAAGCAGGAGGATCACTTGAGCCCAGGAGTTGGAGTCCAGCCTGAATGACATAAGGAGACCC
CTTCTCTAAGAAATAAAAATAAAAACAAATACTTAATAAAGACTCTGTCTTTAGGATAGAGAGCATAGAG
ATATAAAGCAAAGTGTCTTGCCAAAAATGAGTGTTATGGTACCAATATTTGAGTAGAATGAAGAATCTTC
CATTGAGTAGAAAGAGAATTTGTAACATATCTGTGTTTGATGTTTAAGGCATAACAGCTTAATAATGACA
CTCTTCCTCAGACAGGAAGCCTGAAATGTCCTACTTTGACCTAAAGTCTAGTAATAAAACTGGACATACA
CAGGCAACATGTCATTAATTCTCAAACTTTAACAAATCATATATAACCTAATATAATGGTTCTCAAGTCT

Usually constitutive exon 6, encoding bridge sequence between LSM and LSMAD, with residues DAFTDSAISAKVNGEHKEKDLEPWDAGELTAN EELEALENDV

GTACATCACGTCACCTGTATGAAAAATATGAGGAAACAGAGACTTCTTTTACACTATTGGTGAGGTGGAT
AAATTGATAGAGTCTTTCTGGAGAGAATCTGGCAATGCTAATCAAAATTTAAAATGCACATACACTTTGT
TCCAGCAGTTCTATCTCTAGTAATTTATTTTTGCCCTCATATATCCATAAGACATGCAAATAATTATATG
TGAAGATTTTTTTTTTTTCTTTTTCTGCAGAGACAGGGTTTTACCATGTTGCCCAGGGTGATCTGGAACT
CCTGAGCTCAGGTAATCCACCCACCTCAGCCTCCCAAAGTGCTGGGATTACAGGTGTGAGCCATCATGCC
TGACCAGGATTTTTTTTTTTTTTTTCAGCATTATTTCTTTTGTTGTTGCTGTTGTTTTGAGAGATGGAGT
CTCACTCTGTCACCCAGACTGGAGTGCAGTGGTGCGATCTCGGCTCCCTGTAACCTCCACCTCCTGGGTT
CAAGTGATTCTACTGCCTCAGCTTTCCAAGCAGCTGGGACTATAGGCGTGCGCCACCACACCCAGCTAAT
TTTTGTATTTTTAGTAGAGACGGGGTTTCACCATATGTTGGCCAGGCTGGTCTTGAACTCCTGACCTCAG
GTGATCTGCCCACCTCGGCCTCCCAAAGTGCTGAGATTATAGGCGTGAACCACCATGCCTGGCCATAGCA
TTATTTCTAATAGTGAAAAATTGGAAACATGCTAAGTGTCTATCAATATAGCATGAGTTAGATTTATGAT
GTCACCATTCAATTGAAACACTACATATCTCCCAAAAAGAATGGTGTTCCAATATGGAAAGATATCTAAG
ATTTATTAAGAGAAAAAGCACATTGCAGAACACTGGGATCCTATTTGCTTTTTTTTTTCTTTTTTTGAGA
CAGAGTCTTGCTCTGTCACACTGCAACCTCCGCCTCCCGGGTTCAAGCGATTCTCCTGCCTCAGCCTCCT
GAGTAGCTGCCACCATGCCCAGCTAATTTTTGTGTTTTTAGTAGAGAAGGGGTTTCACCATGTTTGTCAG
GCTGGTCTTGAACTCCTGAACTCGTGATCCACCTGCCTCAGCCTCCCAAAGTGCTGCGATTACTGGCATG
AGCCACCGCACCTGGCCATGAAATTTTTTTTTTTTTTTAAAGAGCTGTTCATATTCTTATTGCCTAGAAG
ATGTCTGAAATTACACCCAAGAAACTCTTTTTGAGACGGAGTCTTGCTCTGTTGTCCAGGCTGGAGTGCA
ATGGCGTGATCTTGGCTCACTGAAACCTCTGCCTTCCAGGTTCAAGCGATTCTCCTGCTTCAGCCTTCTG
AGTAGCTGGGACTACAAGCGCCCGCCACCACATCTGGCTAATTTTTTGTATTTTTAGTAGAGACAGGGTT
TCAACATGTTGGCCAGGCTGGTCCCGAACTCCTAATCTCAGGTGATCCACCCACCTTGGCCTCTCAAAGT
GCTGGGATTACAGGCATGAGCCACTGCGCCCGGCTGAAACTCTTTTTTTTTCTTTTAAGATGGAGTCTCG
CTCTGTCGCCCAGACTTGAGTGCAGTGGTGTGATCTCAGCTCACTGCAAGCTCTGCCTCCCGGGTTCACA
CCATTCTCCTGCCCTAGCCTCCCAAGTAGCTGGGACTACAGGCTCCCGCCACCACACCTGGCTAATTTTT
TGTATTTTTAGTAGAGACAGGGTTTCACCATGTTAGCCAGCATGGTCTCAATCTCCTGACTTCGTGATCC
TCCTGCCTCGGCCTCCCAAAGTGCTGGGATACCAGGCATGAGCCACCGTGCCCGGCCAGAACTCTTAATA
GTAGTTATTTATGCACGCTGGGATTGGAAGACATTTACTTTTTACTGGATGTCTTTCCGTATTGTGTGCT
TTTTTTTTTTTTTTTTTTATGTAGGGCATACATTACTTAAGTAATTTAAAGCCTCCATAAGTAAGTGTGA
TTTCCTGCCCATGTGTTTGGCAAAAGGAATTGCATTGGTGGTAGACTTACATTATAGTCTTACCTGGAGT
AGCACAGGAGGACCCAAGGTTAATAGGTGAACTTCGAGGCAAGCCTTAGCATTGAGGTTGCCATCAGCAT
TGCTTGGTTGATGTGTTCATTCTTCTGGGATGGATTACAACCTTTACTGGACTTTATACTTTTCACCAGT
AAGGCTTTAAAAAAGGAGTTGAAACATTAGAGAATAATTATCCAGGCAGTAATATTCACTGGTAAATAGT
CTTCCAGCCTGTGGCCCAATTGGTTGATTCTTTTACGTTAAAGAATGCAGCCTCAGCTGCTCTGCCTATG
GAGTAGGATTCTTTTATTTACTTTCTTAATAAACTTGCTTGCCCCTGGCTCCCCCCCACCAAAAAAAGAA
GGCAGCCTCCCTTTTGCGAATGGTAATTTCCTATAGTTTCCTCGTAGAATTGTGGAGTTACCTATGCTGA
GGTTATAGGTTAGGGTATTGAGATCCAGAGTTGCCACTTCTGAGGTGTCACAACTGCTAATGGTAAAACC
ATTTCTAAAGCCCAGTTCTTGTGACTTTGTCCAGTGATTGCCTGTTCACCGTTTCATGCTGCCTTCCCAT
TTGAGCATTCCCAGGAGGAAGGGGAGGTTGCCAGGGACCTAGTACCATAGTCCGACCTTGGAATCGTTGA
ATATGAGGGAAAGCGTTGGCTTCTCCCTTCTTTCTCCCAAACATTGGAAGTATTTTTGGCTGTTAAAAAG

CACCCCTTGTTCCATGTGGAATCCCTTGTTTAAAAGAAGTAAAATATGTACCTCCTGTCCTCCACAGACC
TGAGGACCAGTGTGATCTCAAGAAGGTTACAGGTAAATGTAGATGTCTCTAACTGAAAGGTGGCTTTTAC
AGGTTAGAGAAAAGAGAGAACCCTGATCTGAAGGCTATTTTATGAAGTAATTAAAATGTTCTAAACTTTA
AAAATAACTGCTCAAATAATTGTGTTGTATAGTTACTTATCAACTGGAGGGGCTGATAAGTATTTTTCTA
AAACATTTTTAAGGAAATTTTTTCCTATTTTCTAATTTGCTAATTTTGCTCAAGTAGTTTGTTAGATATT
GTTAATATAGATGTTGGTTATAACTGAATGAAAGGGAACAACTACTTTGACATTTTGAAAAACAAGCTTC
ATTTTCTTCTAGTCTAATGGATGGGATCCCAATGATATGTTTCGATATAATGAAGAAAATTATGGTGTAG
TGTCTACGTATGATAGCAGTTTATCTTCGTATACGTAAGTTTGAAAAGTTTGTTTTTATTTTAGTGCATT
TGTCTTTGATTTTCATCAGCTTAATTTATGATGAATAAATGTTTGTTAGTTTTTAAGTTAAACAATTACA
TGAAATAATTTTTCTCTTATTACCAACTGTGATAAATTTCCATTAAAAAAAGGGAATAAATGTAGTTTGC
CTATACCCTGTTTTTATGCTCTAAACAAATTTTGGTTTTGTCTTTTTTTTTCTTTTGAGAGGGAATCTCG
CTGTGTCTCCAGGCTGGAGTGCAGTGGTGCAATCTCGGCTCACTGCAACCTCTGCATCCCGGGTTCAAGC
GATTCTCCTGCCTCAGCCTCCCGAGTAGCTGGGACTATAGGCGCGTGCTACCATGCCCATCTAATTTCTG
TATTTTTAGTAGAGACGGGGTTTCACCATGTTGGCCAGGATAGTCTCGATCTCTTCACCTCGTGATCCAC
CTGCCTCGGCCTCCCAAAGTGCTGGGATTACAGGTGTGAGCCACTGTGCCTGGCCGGTTTTGTCTTCTAA
GTTGTTAAAAAATATCTAAATTTGCAAGGGCAGAGATTATGGTGAACAGTTTAACCAGTTTTTGAAATAT
GTTCCTCTGGAGAAAAGGTAACAGAAAAAAAAAGTTAGAATTTTGATTTATAAATACACAGATCACTATAA
CTTTTAGTTTTAGTTTTAGTTTTAGTTTCTGTTTTTACCAGTATTCTAAACTCTAAACTTTCTTAGTAGT
TGATTATGACAGATACATAAACTGTGGCTTTAAAGGACTCATTTTGCTTTTCTTTTCCTCATGTTTCAGA
GTGCCCTTAGAAAGAGATAACTCAGAAGAATTTTTAAAACGGGAAGCAAGGGCAAACCAGTTAGCAGAAG
AAATTGAGTCAAGTGCCCAGTACAAAGCTCGAGTGGCCCTGGAAAATGATGATAGGAGTGAGGAAGAAAA
ATACACAGCAGTTCAGAGAAATTCCAGTGAACGTGAGGGGCACAGCATAAACACTAGGTATTTAAAGGAA
ATCATGATGCAGTATTTTGGATACACAACTCAAGGTCTGTGTGAGACGGTGTATTGTTATTATATTTCCT
CTTCCTTTAATATAGCTTAGGTAGAGAATGCAAGTAGAATTGGTTTAAGATCTGTTAGAGAAAAGGTTAT
GGTGATCTTGGAAAATATGCTTTTGAGAGTAAGCTCTGTGGAGCCAAGTGTTGGTATATCACGGTGAGCA
ATCCAAGATCTTGAAGAGCTTGTTAAAATAGTTATCTGGTGGGGGACACGTGTAACAATCACAGCAGTAC
AATATGATTTGCTTGGTTAAAGGCATGTTCAAAGTACTAGGAACATACAGAATGAGGAGGAGCTAGCATA
ACCTGTAGAGTCAGAGAAAACCTCATTGAGGAGGTGACATTTTGTGATAAGATAATAGGGTCTTTGACAC
TTAGAGAAGAGTTGGGAGAAGAGTTTATCACCTGATGAAAAGCCATGTACAAGCATGGCTATGAGAAAAT
TTGGCCAGCTCAGGAGAGGGCTGGTTGTTGCATGTGTCTGGAACACAGGATCTGTGTCAGGTGCAGCAGT
GGCAGTTGATAGTAGGAACTGAGGTCATTAAAGGACTTGGCATGTCATGCTAAAGAGCACCCTGTTGGAA
GGAGATGGGGTGAATAAACCCTGGGGCATTGAGGACTGGCTGAGACACAGAGAACAGTTAGTGCACTGAA
ATAGTTCAACTGTGAGAATTTGGTAACCACCTAGTTAAGGGATGAGCCTGAGGTTTATTTGATAACTAAG
TGACTTAATGGATGTACTGGTAAGAGAGAGAGGAAACATGGAGCAAGTTTGAGGGGAAAAACAGTGACTC
CGTTTGTGCAGCTAATTGCATATGTGGGCTTGTGGGTCTTTCATTTATTCATAAACGTGTTGAGAAATAC
CTGCTACCTATCTAGTAAAGTAAGAGATGCATCCTCTCTTAAAGGCAGTCAGCTTAGAGTCTGGTGATTT
GAATTGACATGTCCACTGATAGATGTTGACACTGTGAGACTGGCGGTTCAGTTTGAGGTTTCATCAGCAT
TGCCGATATTGGAGCCATGAAAAACCAAAGAACAGCCAGTGAGAGAAGAGATCTCAGAGAAAATAAAATT
GAGAAAGTGAAGGACAAAAAATGTTGTGAAGATAGACCAAGATTGATGGAATCAGCCATAGAGAGGTCAA

Usually constitutive exon 7, encoding residues SNGWDPNDMFRYNEEN**YGVVSTYDSSLSSYT** (with the LSMAD first part in bold letters)

Usually constitutive exon 8, encoding the LSMAD end (bold letters) and intrinsically disordered sequence (green letters), with residues **VPLERDNSEEFLKREARANQLAEEIESSAQYKARV**ALENDDRSEEEKYTAVQRNSSEREGHSINTR

GTGGGATGAGAATGAGCACGCATCTGTTAAACTTTGTGCTTAGGAGCAGAATCTAAGGGAAGGGACAGTC
CAGAGGTTAGAACTCAGGGTAAGATGGAAGAACAGGGGCATCTGGGAGTGAGGCAGTTTGGTTTAGTGTA
GAACCTTTTTGTAACAAGCATTCCCTTCTGTCTAGATGACTTTTAGATATGTTTCATTGGCTTGGTACCT
TTTAGAATAAAATGATTTAGAGGATCTCTCATTTTCAGGGAAAATAAATATATTCCTCCTGGACAAAGAA
ATAGAGAAGTCATATCCTGGGGAAGTGGGAGACAGAATTCACCGCGTATGGGCCAGCCTGGATCGGGCTC
CATGCCATCAAGATCCACTTCTCACACTTCAGATTTCAACCCGAATTCTGGTTCAGACCAAAGAGTAGTT
AATGGAGGCAAGTATTTTGACCAGACTTGTCAATATCATTGATAAAATAGTTTTCTAAATACTTAAAATA
CTTAAAATAGTTTACATAACTGATATGAATGTGCACTTTAATGATTTGGTGAGTAGCTTTCACTTCAGCA
TTACTTAAAATTGGCTTTTGTGGATATTAAATTAGTAAAACATTGTATATGTCATTGACATATATATTAT
TTAGCATGATGAAATATTCATGATGTACTAAGATAAAGTGCTACATTTAACCCAAGACAATCACTTGGCC
AAAAACACTTCACATATAAAGAAATTGGAAACTTTGGGTAGGTTCTCAATTTTAAAAACACTGGATAATA
AAATTTTTTAGACATAATTTATATGGAAAATTCTAACCTATGTGCAACACTGTGGTTAATATAGATCAAT
TTTCATTATTTGTTTCTATATTATGCTTACTTCAAGAAAGGATCTGAGGTAACTTATAATACAAGACATG
ATCAAGAGTCATGTGAAGAAAGTGACTAGAGAAATTTGCTTAAAAAACAACAAAAACAACCCTTAGTCTA
AGGGTGGATGTTACAGTTTAGCAACTTAAGTAAAAGAAACCTGAATCTTTAGTAGGAAGACATTTTTTAC
TCTACCTCTAAATCTAGGTTGAATATATCTTGTAGGTTGTGGATCTTTTCCATAAATCAGGGATACTGAA
CAACAGTTCTATGGATGGTATGGAAATAGTAATAGCAATAGTATGTTACTAACTTTGTGGGAAAAGAGTG
GACATTCAATTTTAGCTATTTAAATTTGGAAAGTTAGATGAAAATAGAGAACACTAAGTTTCCAATTTCA
TTTGTTTTCATTGAGTCTTTTCTCCAGAATTCCTCTCCAAATGGACACTCTTGAGTATTTTCAGTACTTA
ATATTGGGGGTGAAATTTCTTTGCTCACTGAGGAAAGATTTTAGTTGTTTATAAACAGAATTTTAAAGTT
AAAAAACCTGAAGGGGGCTGAGAAATATATGATACTTAAGTGTGTGGAACCCTATGGAGAGGAGACCTGG
ACTGTTTGATAAGATTAAGGTAAGTGATATGTAATGTTAAATACTAGCTGTATCTTTACCTAGGCATATC
CATCAGTATAAATTTATTTGGTGATGACTGCTTTGTAGTTGCAGTATTTATTAAGCAGTCGCTTAGATAA
GTGTTTAACTGTATAAATTATTTAGAAGGTCTCCCTTTTTCTAGTTTAATGAGGTCAAGACTTTTTTTTT
GAAATAGCAATGAATATTATCATTTGATACTCACAGGAGTCACAAACTCTAGAAGAGTAATGTTTTATTT
CTACTTAAATGGGACTTGCTTAATAAGATTCCAAACTGAGTTCTGGGTTCAAGTGTAAACCTGATGAAAA
TCATAGATAATTGTAAGGAACCAGCATTTCTAATTGGATATAATAGCTACTGCTTATTTTCGTTATGCCT
CAGAGTTAAAACTAATACAGTAAATAATCTTACTCCTGAGTAGGAATTATTGTGATTTATTATGTGAAAT
TATCTAGTGTATGTTATATTCCTTTAAACAACCAGTTACTGAGAAACAGTTATAGAAGCAGGATTAATAG
GCAAAGTCTTAACTGTCTTCTTCAATAGTGTGTATAGATCCTAATTAACCCTTTGGGAACGTGTATTCAT
TTAAACAGACTTAATCTTAAGGAGGTTAAAGTAAAATGTGAATTTATGTCAGTTAAGTTATGCTAAAACT
TATCACAAATCAAATGACTGTCCTCAAAGGGTTAAAATGTACAAGAAATCATTTTTGTCATTTTACTTTT
TTTCTGTTTACTTTTTTCCCTCATTTTTTTCTTTAGTTTTTATACTTTCCTTCATATCATTTGTTCTGTC
AGGTGTTCCCTGGCCATCGCCTTGCCCATCTCCTTCCTCTCGCCCACCTTCTCGCTACCAGTCAGGTCCC
AACTCTCTTCCACCTCGGGCAGCCACCCCTACACGGCCGCCCTCCAGGCCCCCCTCGCGGCCATCCAGAC
CCCCGTCTCACCCCTCTGCTCATGGTTCTCCAGCTCCTGTCTCTACTATGCCTAAACGCATGTCTTCAGA
AGGTACAATACCACAATTTGTTCATGTTTTTGTTTGTCTTTGTTTAACTCCTATGTGAGTTTATAATTAC
AAAATAGTTTCCTCTTCATTATTTAATAACCTATAATTTCTGTGTTTTAACTTTAGTTTATTAAAACTAT
TTCTATTAACCTTTTGTTCATTAGAGAGAAATTTGATAAATGTGTGAAGCTATAAACTCTCTTGAATTGT

Usually constitutive exon 9, encoding an IDR2 fragment (green letters), with residues
ENKYIPPGQRNREVISWGSGRQNSPMGQPGSGS
MPSRSTSHTSDFNPNSGSDQRVVNG

Usually constitutive exon ante-10, encoding ISD2 fragment (green letters) with residues
VFILSFISFVLS,
spliced e.g. in Homo sapiens KAI2568021.1, KAI4068270.1
and in chimpanzee
XM_063784946.1,
XM_063784951.1,
XM_063784947.1,
XM_063784952.1,
XM_063784948.1,
XM_063784949.1

Alternatively spliced exon 10, encoding ISD2 fragment (green letters) containing a proline-rich motif (bold letters) and enhancing arginines (underlined letters), with residues
GV**P**W**P**S**P**C**P**S**P**SSR**PP**SRYQSG**P**NSL**PP**RAAT**P**T
R**PP**SR**PP**SR**P**SR**PP**SH**P**SAHGS**P**A**P**VSTM**P**KRMS
SE

TGTTAAAAAGGGGGTTTATCTCTGCCTGATAATTATGCTTCTTTACAGCCCCAGAAGGGTCTGCCCCACA
GCCTTCCCCCTCCTTATTTGCACTGTATACAGTAGTTAAACAAATGAACTTTCTTCAGCCAGTCTTGAAC
TTAGGTTCATTTTACAGCTCTTTGGCCAAGGTCCTAGTGAACCTTCCTATTGGCCATAAGCAGGGATGGT
GTTTTCTGGGTCTTTTTTGAGAGCGACAGCCCATGTAGCTGACTTTGCGTGTCTGCCCTTAGATTAAAGT
AGTTGATTTTTAGAATGCCAGAAGAATTCTAAATTTAACTGAGTAATTTTTTTAAAGTTAGCTTTGCAAT
CTTACATAGTGAAAGGCTGCTTTAATCTGGAAGAAGTCCTTGATCTGAGATAAAATTGATAAAAACGACA
TATGAATTTGAATATTTAGCTATTTCTTTCCTCGTCAAAAATAAGAATAAAATCTTGTAATTCTTATTCA
GTATTTGGCGCTAAATCCATCATTGCCACATATCAAATACAGGGATATGTTGTAGAAAGGTAACATTCTA
ATTTAAATGCCACCCATATATTAAAAACCTGTTTTCTGAATCATAATGTCCTTTTGATACTAGTTCTGAA
TATTTGTGTTAAAATTTTAATCTGATTTGTTCATTAAAATTAGTTAATATTGCTTATGTTGGGACTAATA
AAGTTTTCCGCACAAAATGTGTTTCTCCTGCTTCCCTGGAGAAAACTGTATTGGCTACTTTTAAATAAAT
TGTTACCATCTAAGCAGGCAGGTCATATGACTTTGACTGAAGCATCTAACCTTGAAGAGCAAGTTCCACT
GATTTTCAAGGTGACTTCTTTGCTCAAAAGGGCCTTAATAGTGGTCACTAAATGCAAAATTCTGTTGATA
TTTTTCTTGTAGTCCATCATTTGAGTAAGCGATGTTTATTTAATGAGAATATATTAAATAAAACATGATC
ATTAATGACTGTGAACATCTTTATTACATTAAGATTTAAGGACTGCTCATGTATTAACTTCACACAGAAA
TATACTTTCTGTGTCATTCAGAGATGTTGAATATTTCCATTTGAAAATTATAGTGTATAACATTAGCATT
CTTCTAAAGATCATGTTCGTGTTTAAATTCCTGTTGGAAGCCAGGCATGGTGGCTAACGCCTGTAATCTC
AGCACTTTGGGAGGCTGAGGCAGGTGGATCACTTGAGGTCAGGAGTTTGAGACCAGCCTGGCCAACATGG
TGAAACCTCGTCTCTACTAAAAATACCCAGCTACTTGGGAGGCTGAGGCAGGAGAATCACTTGAACCTGG
GAGGCAGAGGTTGCAGTGAGTTGAGATCGTACCACTGCACTCCAGCCTGGGCGACAGAGACAGACTCTGT
CTTATAAAAATAAAAATAAAATAATAATTCTATTGGCAACATATATTAATTTGAAGTTCTAAAGAGTTTG
GCAGCCGGGTGAGAGAGTGAGGAGATTTGGCTTTGACATTAGGGAAGTTTTCGCTTGGTGTTAACACCAG
TAGGCTTCTCTGATGAGGGCCATTCTGTCCACTCTTTTACCTGATAGATTGGTCTAATGCACAGTAGACT
GATTTAGAAAGAGTAGTCACTAGTGGCATGGCAGAATCAATAATGTAGAATTTTGACAATTCATATAGTG
CTGATTTCTCCCCCAAATGTCAGTTATTTTGGTCATCTATTAATAGACTAATACAAGTCATCCCTTTAAT
AGAATTTTCAGCTCACAGCCTGCTAAGCCTAAGAAACTGCTTACAGGTTACTGCTTACTGTTTTAAGCCG
AGTTTTAAAATTGATGATCATGATAGAAGAGATAAATAAACTAAAATTTTAGAGAAATTTAAGAAGGGTA
TGTACATATGTTTTAGTGGTATCGGGGTGTATAGGGATTAATAGTCTTCTGTTTAAATTTTTTTTTTCTA
ATTTTAGAAGTAATGTAGAAAATTCGGGTCAGGGAAAGGTAAAATATATGGAAAGTTAAAAATATTTTAT
CATGTAGTCATAATTTCTAGTAACATATTTCTTTACAAATAAGACATAGTTGAAACAGATTGCTACAGTT
CTTTTAAGAGTTGACATCTTATTGTTGATTTCTTACCACCAACTTCATCCCTCCCTTTCTTTAAAAATAA
AGGGAAATAATAAAATTTATTTATAAAACTTTGTGGCATTCCACAAAATAATTCTGAAAGAATTAGTATG
GCCAAAAAAATATGTATGGTGTTTTTTTTTTTTCTATTTTTAACCAAGGAAAAACTGTAGAGTGAGTGAG
TGTGTGTGCATGTGTGTGTGAATGGGTGTATTTAGCAGAAAAGTAGTACTGATGAATATCATGGAATTTA
TGTGATGTTCACTGTTTCTTCCTTAGGGCCTCCAAGGATGTCCCCAAAGGCCCAGCGACATCCTCGAAAT
CACAGAGTTTCTGCTGGGAGGGGTTCCATATCCAGTGGCCTAGAATTTGTATCCCACAACCCACCCAGTG
AAGCAGCTACTCCTCCAGTAGCAAGGACCAGTCCCTCGGGGGGAACGTGGTCATCAGTGGTCAGTGGGGG
TAGGTAACACTTGGGCATAATGATGGTACTCATTTTGTCATTACACTAGATATAAAGAGGGCTGAGCTAC
AACTCTGTTTGAGGAAGTGTAAGTATGTATATGTTAAAAATAGTAGAATCACCAGGAATTGGGAAACCCA

Usually constitutive exon 11, encoding ISD2 fragment (green letters), with residues GPPRMSPKAQRHPRNHRVSAGRGSISSGLEFVS HNPPSEAATPPVARTSPSGGTWSSVVSG

TATTTTTATTCTGGGCTCTACCACTTATTCATCATATATTAAAGCAAGTCAGACACTCATTCTGAAGTTG
AGATTTCGCAGTGAGTAAAGTGTTAATAATTCTTGCCTAGTCTACATTATGGGATTGTGATGAGATTCCT
ATAAGGTTCATAAATACAGATATATTGTAAAACTATAAAGTTTTGTAAAGTACCTCTCTAATATGAGGCA
AACACAGTATGTAACACTATTTGGAGGGACCGTATTTCCTTATCTTTTTAGCAGCTTTGTTTATCAGTAC
ATTCTATAAACATTTATTTTTGGCTTACATTGTAGTGTGTTTCTATAGCATCTGTATATGGCACTAATTC
CCAACTATATTTCCATAATAAGGAATATCAAATACAAATAAAGGGTCCAAGTTTTATTTGTGATTAGCAT
AAGGAATATGCTGACAGCAGCTATAAAAGTATAAAAATTAGGCTGGGTGTGGTGGCTCACGCCTGTAATC
CCAGCACTTTGGGAGGCTGAGGTGGGCGGATCACAAGGTCAGGAGATCGAGACCATCCTGGCTAACACGG
TGAAACCCCGTCCCTACTAAAAGTACAAAAAAAATTAGCCGGGCATGGTGGCGGGTGCCTGTAGTCCCAG
CTACTTGGGAGGCTGAGGCAGGAGAATGGCATGAACTCGGGAAGCGGAGCTTGCAGTTAGCTGAGATCAC
GCCATTGCACTCCAGCATGGGCAACAGAGCAAGACTCTGTCTCAAAAAAAAAAAAAAAAAAAAAAGTTTAA
AAACTAGACGTTGACATGATTTTACAATAAGGCTGACTGCTTTTGCTACTTTGCCAATCAGTCCTTAGTG
CTTTGTTCCCATAACTGTGGTAAGCAAGAGCTTACAAAGAATACTTAAAACAAACAAACAAACAAACAAA
AAAAACACTTTTTCTCTTTTAATCAGTCCAGAGAACCTTTAAAAGAAACAAGATCGGCCAGTTGCTGTGG
CTCATGCCTGTAATCCCAGCACTTTGGGAGGCTGAGGTGGGTGGATCACTTGAGGTCAGGAGTTCAAGAC
TGGCCTGACCAACATGATGAAACCCCATCTCTACTAAAAATACAAAATTAGCTGAGTGTGGTGGCTATTT
GAGAGGCTGAGGCAGGAGAATCATTTGAACCCAGGAGGTGAAGGTTGCAGTGAGCCAAGATCACACCATT
GCACTCCAGTCTGGGTGACAAGAGCGAAACTCTATCTCAAAAAAAAGAAAAAAGAAACAAGATCTTCAAG
CTTAAGGAAACAAAAACAAAACTCAGCTGTGTTAAATCTGTTTTTAGTTGCTATACATTTCTGCTCAGCT
TCATGTGATGCACATTCATGTAATTGTATCCTAAATTCCTTTGTACTTTTTATTTTCTTCCTTGGTCTTC
AATTATCTTAAGACTACCAAGAAAACAAAAATTTTAAAAATCTTCTTCAGCCGGTCAGGCGCAGTGGCTC
ACGGCTGTAATCCCAGCACTTGGGGAGGCTGAGGCGGGTGGATCACGAGGTCAGGAGTTCAACACCAGCC
TGGCCAACATGGTGAAACGTCGTCTCTACTAAAAATACAAAAATTAGCTGGGCATTGTGGCGCGTTCTTG
TAATCCCAGCTGCTCAGGAGGCTGAGGCAGGAGAATTGCTTGAACCAGGACCCGGGAGGTGTAGGTTGCG
GTGAGCGGAGATCGCGCCACTGCACTCCAGCCTGGGCTATAGAGTGAGACTCCATTTCAAAAAAAAAAAA
AAAAATCTGCTTCAGCTATTCTGTTAATCTTTTGACATTACTTAGATGGTCTGGAAATAAATTTTGAGAA
TAACATGATTAGAAGTGAGAGAGTATAAGCATAGTTTTGGAGATACACTCAGAATAGCATTATAGATTTT
CTCTTTTTACTAATTGGAAAAATGGCAGTTGTTGAATAATAGTTTTCTTCCGTGACCCTTGTGACTTAAA
AAAAAAAAAACACTGAAATGAAATAATCGAACCATTTTCTCTAAACCTTTGAATCTGAGCTCTGCAGTTA
GGTTTATAATGGTATATGAAACCTATTAGATATATACTTGGAAGTCATATGGGATACAAACCCTGCTTTT
ATTATCTTCCCCTTTTGACTAACTTGGGTCTCAAGTTTCCTTAATTACTGCACAGTGGACCTTGATGTTG
CTATAAAGAATGTGTAGGGCTGGGCATGGTGGCTCATGCCTGTAATCCCAGCACTTTGGGAGGCCAAGGT
AGGCAGATCACCTGAGGTCAGGAGTTTGAGACCAGCCTGGCCAGCATGGTGAAACCCCGTCTCTACTAAA
AATACAAAAAAATTAGCTGGTTGTGGTGGCGAGTGCCTTTAATCCCAGCTACTCCAGAGGCTGAGGCAGG
AGAATCACTTGATACATTTAGTTAGGAGAGAAAATCATACTTATGTTAGTAATTGCTGCTGTTCTTCATA
TACTTGTGGTTTTGATTGCCAGCAAATTCCTAACATTTTGGAAAAGAAAACAGTAATGGGATAAAGGGTA
AGGGCTAGAGAGGACAGTTTTATTTACCTAGATCTTCAGAGAAGCCTGAAGCCTCTTTTAGGAAGTAACA
TTTGAACTGAGAATGTAATAAATACATTTTCCCTTTCTTCTAGTTCCAAGATTATCCCCTAAAACTCATA
GACCCAGGTCTCCCAGACAGAACAGTATTGGAAATACCCCCAGTGGGCCAGTTCTTGCTTCTCCCCAAGC

Usually constitutive exon 12, encoding IDR2 fragment (green letters), with residues VPRLSPKTHRPRSPRQNSIGNTPSGPVLASPQAG IIPTEAVAMPIPAASPTPASPASNRAVTPSSE

TGGTATTATTCCAACTGAAGCTGTTGCCATGCCTATTCCAGCTGCATCTCCTACGCCTGCTAGTCCTGCA
TCGAACAGAGCTGTTACCCCTTCTAGTGAGGGTATGTAACAAAGGGCTTCTGGATCCATAATCTCAGCTG
TGAAATTGAATGTTAGAGGGTGATATTATATGAAAAAATTCTAGGTTATTTTTATTCATAGACAAGTATT
TTTAGTGCACATTTAAAAGTTTATGTAAATTTTGATGTTGTTTAATACTACTAATTTAATATAGTGTCTG
TGTTACAAAGGTTAACATTCCTGGGTGTCAAATACCTACATAAATAAAATTATTGGTGTTTCATATGACA
TCTGCAAAGGAAAAAAAGCCTCTGTTTAAATGAAAGCATTATTTTCCAAAAACATAGGAAATCAAAATTA
TTGTTCAGTGTTTTCTTGTTTTGCTTTTCTAACTTATCTGAATTTTTTTTAAAAAATTGTTTTCTAGCTA
AAGATTCCAGGCTTCAAGATCAGAGGCAGAACTCTCCTGCAGGGAATAAAGAAAATATTAAACCCAATGA
AACATCACCTAGCTTCTCAAAAGCTGAAAACAAAGGTTAGAGTTTAAAGAGTCATTAAGCTTAACTGTAG
GAATAGGAAGAAGTATGTCTAATTTCATGCCCATACAGAATATTTTTGTTCAACATTTCTTCTTACTATT
GTGATAGATAAATGTATTGCTTGACAAATTCCAAAATCCAAATTTAATATTTGAAATTATTTTCTGATCT
TATATCTTATTCTAATTTCTATCATCTCATACTAAAAAGAATGTGATGTTAAAGTTTAAAAATAAACCTG
TGTCTTAACAGTTCTTAATTTTACAGGTATATCACCAGTTGTTTCTGAACATAGAAAACAGATTGATGAT
TTAAAGAAATTTAAGAATGATTTTAGGGTAAGTATTGTACTAACTGATGAATTTGAGTTTTAGAAAATAA
GCATTACTAAAGATTTATCTATTTATAAAAATGCGTTATGTATACAGTCAGAAACATCAAACCATATATG
TAGAAAGCAGAACATTTTTAAAGTGGTCTTTGCCTATCCTTTAAGTGGGATAACTAAAATCATGAGATTT
GGTAACAACAATATGTAGGTATCAAATGAGAGTATAGCCCTGACATTTGAAACCACCATAGCACAGCTTA
CTATTTGATGGTCATTTGTACTTTGTTCAGTGAAGCTAGATATTAGTAGAGCAAGGCCAAGTCATTAATA
ATCTAGTGTGGCAAATGGAAGATGTACTGGACTCTGGTGTTCTGAGGTAGTTGGAGATTTATACTTTGTA
CACAAATATATTGTGGTCAAAATCTTTCTGTAACATTATTTCTCTGTCTTAGCACAGGCTTTACTTAACA
TCTCTCCTTGATTGTCATTTCATTCTTTTGCATGTTATTTACTATAGGTATCGAGGTAGATTTTGAGACC
AACCAATAAATCTTCTTGAAACTTAGCTTCTTAGAAAGGAAAATCTAAATACCAGCCTTTTAAAAAAAGT
AGCTGAATTAAAGGATGAGTGAACCAAAGGCAAAGGTAGCCTTTCCTCAGCCTGTGTTTTAGCTTTCTAA
ATGTTAACAATAGCTTCATTCTTGACTTATTGGTAACATTCAAAATACTACTTATTATTTCATACTTTAG
CACATGTATCTATTCAGCTTTAATGCTATTAACAGTTGTTAACCTAAGTTTTCATTTGTTGGCGGGCACG
GTGGCTCACACCTGTAATCCTAGCACTTTGGGAGGCCGAGGTGGGCAGATCACCTAAGGTCAGGAGTTCG
AGACCAGCCTGGTCAACATGGTGAAACCCTGTCTTGACCAAAAATAGAAAAATTAGCTAGGCATGGTGGC
GCACACTTGTAATCCCAGCTACTTGGCAGGCTGAGGCAGGATAATCGCTTGAACCCAGGAGACAGAGGTT
GCAGTGAGCCGAGATCACACCACTCCACTCCATCCTGGGCGACAGAGCAAGACTGCATCTCAAAAAAAAA
AAAAAAAAAAAAAAGTTTTTCAATTTGTTAAACAATAGTTAACACATACAAATGATACAAAGAATATTGA
ATATGATCATGTGCCCACTACCCAGCTTAGTAAATAAAGCATTCTAACACAGTTAAACTCCTCTTATGTA
TCTGCCCCTCCTCAGCTGCTTCCCCCTGTTTCCTTCCAAAAGGAAGGGTTTCTTTTCTGTGCAGTTCTTT
ATATTTATACTGCATATGAATATATCTGTGAGCAATAGATGATATTTTGCATAATCTTAAATTTGCTATA
AAGTCTTTTTTTTTTTTTTAATTGATCATTCTTGGGTGTTTCTCGCAGAGGGGGATTTGGCAGGGTCATA
GGACAATAGTGGAGGGAAGGTCAGCAGATAAAAAGTGAACAAAGGTCTCTGGTTTTCCTAGGCAGAGGAC
CCTGCGGCCTTCCGCAGTGTTTGTGTCCCTGGGTACTTGAGATTAGGGAGTGGTGATGACTCTTAACGAG
CATGCTGCCTTCAAGCATCTGTTTAACAAAGCACATCTTGCACCGCCCTTAATCCATTTAACCCTGAGTG
ACACAGCACATGTTTCAGAGAGCACAGGGTTGGGGGTAAGGTCATAGATCAACAGGATCCCAAGGCAGAA
GAATCTTTCTTAGTACAGAACAAAATGAAAAGTCTACCATGTCTACTTCTTTCTCCACAGACGCAGCAAC

Usually constitutive exon 13, encoding IDR2 fragment (green letters), with residues AKDSRLQDQRQNSPAGNKENIK PNETSPSFSKA EN

Usually constitutive exon 14, encoding IDR2 fragment (green letters) with noteworthy alpha-fold confidence (blue letters), and residues KGISPVVSEHRKQIDDLKKFKNDFR

CATCCGATTTCTCAATCTTTTCCCCACCTTTCCCCCTTTTCTATTCCACAAAGCCGCCATTGTCATCATG
GCCCGTTCTCAATAAGCTGTTGGGTACACCTCCCAGACGGGGTGGTGGCCGGGCAGAGGGGCTCCTCACT
TCCCAGAAGGGGCGGCCGGGCAGAGGTGCCCCCCACCTCCCGGACGGGGCGGCTGGCTGGGCGGGGGCTG
ACCCCCCACCTCCCTCCCGGATGGGGCGGCTGGCCGGGCGGGGGCTGACCCCCACCTCCCTCCCGGACGG
GTTGGCTGCCGGGTGGAGATGCTCCTCACTTCCCAGACGGGGTGGCTGCCAGGCGGAGGGGCTTCTCACT
TCTCAGACGGGGCGGCTGCCGGGCAGAGGGGCTCCTCACTTCTCAGACGGGGCGGCCAGGCAGAGACGCT
CCTCACCTCCCAGACGGGGTCGCGGCCGGGCAGAGGCGCTCCTCACATCCCAGACGGGGCAGCGGGGCAG
AGGCGCTCCCCACATCTCAGACGACGGGTGGCCGGGCAGAGACGCTCCTCACTTCCTAGACGGGATGGCG
GCCGGGAAGAGGTGCTCCTCACTTCCCAGACTGGGCAGCCGGGCAGAGGGGCTCCTCACATCCCAGACGA
TGGGTGGCCAGGCAGAGACGCTCCTCACTTCCCAGACGGGGTGGCGGCCGGGCAGAGGCTGCAATCTCGG
CACTTTGGGAGGCCAAGGCAGGTGGCTGGGAGGTGGAGGTTGTAGCGAGCCGAGATCACGCCACTGCACT
CCAGCCTGGGCACCATTGAGCACTGAGTGAACGAGACTCCGTCTGCAATCCCGGCACCTCGGGAGGCCGA
GGCTGGCAGATCACTCGCGGTTAGGAGCTGGAGACCAGCCCGGCCAACACAGCGAAACCCCGTCTCCACC
AAAAAAATACGAAAACCAGTCAGGCGTGGCGGCGCGGGCCTGCAATCACAGGCACTAGGCAGGCTGAGGC
AGGAGAATCAGGCAGGGAGGTTGCAGTGAGCCGAGATGGCAGCAGTACAGTCTAGCTTCGGCTCGGCATC
AGAGGGAGACCGTGGAAAGAGAGGGAGAGGGAGACCGTGGGGAGAAGGAGAAGGAGGGGGAGGGGGAGGG
GGGGAGAGGGAGAGGGACAATGATGTCTTGCTGTAGGTATTCTTCCCCATTTGAATTTTTTCCTCAGCAT
TATTTTTTTTAACATCATTCAGTCTCCTCTTATACTACACTTGGATTGAATTTAATATCTCATGAAGAAA
AAACATTTCTACTTTGAAGCATGTGAATTAGCATGTTTTTATAACAGCTTTATTGAGATATAATTTACAT
ATATAAATAAACCGTTTAAAGTGTATAAATCAGTGGTTTTTAATGAGATATAATTTACATATATAAATCA
ACCATTTAAAGTGTATAAATCAGTGGTTTTTAAAATATTCACAATGTTGTACAACCGTCTTCTCAGTTGA
TTTTAAAACATACTCTTCACCCCCAAAAGAAACCCCGTGCCCAGTTTAGCAGTCGTTCCACATTTGCCTC
CAGCCCTTCTCTTTCCCCTACTCCCAACCCTAAGCAACCGTTAATCTACTTTCTGTCTCTATGGATGGGC
TTATTTGGGGCAAATTCCATTTCATACAAATGGAATAATAAAATATGTGGCTTTTATGACTGGCTTCTTT
CACTCAGAGTAGTGTTATAAAAGTTCATCCATGTTGGAGCATGTTTCAGTACTTCATTTCTTTTTGTGAC
TGACTAATATTCCTTGATGTGGATAATACCACATTTTGTTTATCCATTAATCAGTTTGTAGCTATTTGTG
GTGTTCTCACTGTTTGACTATTCTGAATAACACTGCCACAAACATGAGTGTGCAGTTTTTTTCTCGTCCT
ATCTTTTCATTTCTTTTGTGTACCTACCTAGGAGTTGAATTGCTGGGTCATATGGCAACTGTGTTTAACC
TTTTGAGGAACTACCAAGCTATTTGCCAAGATATCTACACTATTTTACATTCCCACCAGCAGGGTATGAG
GGTTTCTGTTTCTCCACATCCTTGCTAACACTTATTGTCTTGTCTTTTTTGATTATAGTCATCCTTGTGG
GTGTGAAGTGTTAACCTCATTGTGGCTTTAATGTGCAGTTCTTTCATGGCTAATGATGTTGAACATCTTT
TGTGTTTATTGGCCATTTATATATCTTCTTTGGATTGATGTCTGTTCAAATCTTTACCCATTTTAAAAAT
TGAGTTGTCTTTTTATTATTGGGTTGTGGGAGTTCTTTATATATTGTGTGTACAAGTCCCTGTTAGATAC
ATGGTTTGCAAATGTTTTCTCCTGTTCTGTTGGTTGTCTTTTTACTTTTTCATCCCTTGAAGCACAAAAA
TTTTTAATTTTGATGAAGTCCAATTTATCTGATTTTGAAGTAAGCTTTTGGTGTCGTATCTAAGAAAATA
CTGTTTCATCAATCATTAAGGTTTATTACTCTTCTGGGTTTTTTTAAGAATTACATTTAGAGGTGTGATC
CATTTGGAGCAACTTTTTTTTTCTTTTGACACAGAATCTCGCTCTTTTGCTTAGGCTGGAGGGCAGTGGT
GCAATCTTGGCTCACAGCAGCCTCAGCCTCCTGGGCTCAAATGAGTAGCTGGTACTACAGGTGTGCACCA
CCACACCTTGCTATTAATAACTTTTGTATTTTTTTGTAGAGACAGAATTTCGCCATGTTGCCCAGGCTGG

TCTCAAACACTTGGACTCAAGTGACACGCCCACCTCAGCCTCCCAAAGTGAAAAATTGCTTTCACCTTGC
ACTGCGGACTCGCCCTGAATTCTTTCTTGTGCAAGATCCAAGAGCCCTCTCTGGGGGTCTGGATCGGGAC
CCCTTTCCTATAACAATATTATGAGAATAACATTTGATTTTTTTTAAGTGAAACAAATTGTTATTAAAAA
ATTAAAAAAGGTCATAGGAGAGTGACTTGGTGCTCAGCCCATTTTGAGCAGTTATTTAATATAGCATAAG
GTGGGGTTCAAATTCATTCTTTATATTAATTTTTTATTTCTAATTGACACATAACCATACACTTATAACC
ATTTTTACTGTGTAAGTTCAGATTCATTCTTCCGTATGTAGGTATTAGTTGTCCCAGCACCATCTGTTAA
AAAGACTATTCTTGGCCAGGCACAGTGGCTCTCAACGCCTGTAATCCCAGCACTTTGGGAGTCCCAAGCA
GGCAGATCACATGAGGTCAGGAGTTCGAAACCAGTCTGACCAAATGGTGAAACCGCATGTCTACTAAAAA
TACAAAAATTACCTGGGTGTGGTGGCGCACACCTGTAGTCTAGTCCCACTACTGTAGTGGCTGAGGCAGG
AGATTCGCTTGAACCCAGGAGGTAGAGGTTGCAGTGAGCTGAGATCATGCACTCCAGTGTGGGCGACAGA
GTGAGACTCCATCTCAAAAAAAAAGACTATTCTTTCCTCCATTGAATTATCTTCACATGCTTGTTGGAAGT
CTGTTGACTACAAATGTGAAAGTTTATTACTGGACTCTGAATTGTCCTCCACTGAATCTCTATGTCTTAT
CCTTATGGCAGTACCATACTGTCTTGATTAGAGTTACTGTATTTTAAAAGGCTGTACTTTTTCAGTTAGC
AGAAAACATTTTAGCTATCAGCACAACTTTCTGTAAACCTTCATTAATGCTTGACTTAAATTCCAAGAAG
GAGCAACATAAAAAGTCTTATCTCTTTAGGAGTTTTAGTCTTACTACTTTTAGGTGCCTGAATAACCAAA
TGTATTATTTAGCCTCTTACTAATAACTCCTTGATCCATAGGGGCATACCAGGAAGAAAAGAAGTGGTTT
TTAAAAAATGAGAGTGGGCCGGGCACGGTGGCTGATACCTATAATCCTAACACTTTGGGAGGCTGAGGCG
GGTGGATCACTTGAGGTCAGGAGTTTGAGACCAGCCTGGATAACATGGCGAAACCCTATCTTTATTAAAA
ATATATAAATTAGCCGGGCATGGTGGCACATGCCTGTAATCCCAGCTACTCAGGAGGCTGAGGCAGGAGA
ATCACTTGAATCCAGGAGGTGGAGGTTGCAGTGATCCGAGATTGCATCAGTGGGCGACAGAGCGAGAATC
TGTCTCAAAGAAAAAAAAAGAGAGTGGAAAAAAAAAAATATGTGTCCCAGAACTTAAATTTTAATTAAAAA
AAAATAAAAGAGTGAACTTTCTAATTGTTCTCTTCAGATAATATAATGTTATTCTCTTATGTTTTATTGC
GTATTTCCTGTGTACCAGATGCTGTTCTTCATGCTTGTATGTTAAATCTTGTCTAACATCTCTGTCAAGC
AAGTTCTGTTTGTATCTGCACTGTGTATATTAGGCAGCTTGGGCAAAGAGAAGTTAAGTAATCTGCCCAA
ACTCACATGGCTAGTAAGTAAGAGGGCTGACCATCTGGTGTTTAAGCTTCTAGCAGTGCTTTGAATAGTA
ACTAATGCATAGTGCATGCTGCACTGTCAGTCAGTGATTCATTAGAGCTAACTTCATGACATGCTCATAG
CCCCAAACTGCATTTGTTCACAAATATCTGTAGTCCTTCATTTAGGCAGAAATAGAAATACCTTGTGTGT
TTGTTGTTCCTTCCCTTTTGAGCCATATGCAGAGTGCTGATAGCTTTATTTGTGTAAGAATTGCTAGTAA
TTTGATCTGTTTTGGGTTAATAATGTGGGTTTTAGAGGTAAATGGACCTAGGTTTGAATGTTGGCCTCTA
TACATCATGTGCGTAACATTGTGGCATGCTATCTACTTCCCCCAAGCCAAAATGGGTTAATTTTAGAACC
TGCTTCATAGTGTTCCTGTGAGAGCTCGATGAGATATTGCCTATAAAGTGTTTAGCATAGTGCCTAGCAC
ATGGTATGTATTCAATACATGTTCATTCTTACTAGCAAAATATAGATGACCCAGTATTGTACAGAGTATG
TACAATGGTGTCATTGTACCATTTCATGTGGAGTCACATAAGAATTTCAGTTTTCTGCTGGGCATGATGG
CTCACTCCTGTAATCCCAGCACTTTGGGAGGCTGAGGTGGATGGATCAGCTGAGGTCAGGAGTTCCAGAC
CAGCCTGGCCGACATGATGAAACCCCATCTCTACTAAAAATACAAAAAATTAGCCAGGCGTGGTGGCAGG
TGCCTGTAATCCCAGCTACTCGCAAGACTGAGGCAGGAGAAATGCTTGAACCCGGGAGGCGGTGGTTGCC
ATGAGTTAAGATCGTGCCGCTGCACTCCAGCCTGGGCAATAAGAGCGAAACTCCGTCTCCAAAAAAAAAGA
AAAAAAAAGAACTTAAGTTTTCCATTAGATTTAGTATAGTGCAGAGAGGAAATACAGCAGAGTGCTATAT
TCCATATATAGCAATATAGCATTAGAACAATATATTCCAATACAGCAGAGTGCTATATTCAGATACCAAC

TAGTGGACTTGCTATTTGTAAGATGGCAATAATAGTATCTACATCAAATAGGGCTGTTGTGAAGACTAAA
TGAATAAGTCTATAAATAGTTTAGAACAGTGTCTGGACAGGTACAGTGGCTCATGCCTGAATCTTAGCAC
TTTGGGAGGCTGAGACAGGTGGATAGCTTGAGCTCAGGCATTAAAGACCAACCTGGGTAACATGGTAAAA
CCCTGTTTCTACAAAAAAATACACACATTAGCCAGGTGTGGTGGCACATGCTAATAGTACCAGCTACTCA
GGAGGCTGAGGTGGGAGAATCACTTGAGCCTGGGAGATGGAGGTTGCAGTGAGGTGAGCTTGCACCACTG
CGCTCCAGTCTGGGCAACGGAGTCAGACCCTGTTTGGAAAAAAAAAAAAAAAGTGTCCAACCCATAGTAAG
AAATGCAGATGTGTTTGACATTGTAAGAAAAAGCAACACCAAAAGTCTGATTTTTGCCTTCACTCAAGAA
CTCTTATGATAATTAAACTCCGAAGTCCTTGGCAATATATATAGTTGGTCTGTTATGTGGATCGCCTCTA
CTAAAGATTTTTGTGAACAAATGAAAGTTTAAGTAGTAAGTTCCTACATCGTGACTTAAATTGCCAGTGT
GCCCACATAAATACCCTGTCAACATTTGCCCTTAGCCACTTGACTCTTTAGCTATATTGGTAATGCAGTA
AAGCTTGCGATGCGCCAGAGTTGCATAATGCTGTTTGCCATGACACCAAGAGCCTTGGTAATGAAACCAT
TGAAATTGGTTTGCCTATACTGAGGCTGAAGAGGTATCTTGGCTCTCTAATTTTAAGGCAACCTTTTTGG
CTGTGTAGGTTTCTCTTTAGCTTGTTTCTCACCACCTGGGGCTGTGGCTTAGGTCCGTTGTCCTAACCTG
TGGCTTAGGTTCTGTTTTTGTTGCTTGTACTTGCTCCCCCTTTTTTCAGCCATTCCTGTTTTCTTTCTTT
TGTAGAGGATGCCATCTTAAATCATCTTCAGCCAGTGGTAGCATTTTATTTTTTCTGGTCTGCAAACTTA
AAAACCTCATCACTTATTTTGCTAATATCTTTGTCTTCTGTTCTTTTTGATGGTCCTTGGTTTTGCAGTC
TACTTTAAAGGTTTTTATTTTTTTATGGGTACATAGTAGACGTATTATTCATAGGGTCTGTGAGATATTT
AGATAAAGGCATATAATGTGTAATAATCACATTAGGGTAAATGGGGTATCCATCACCATCATCATTCATC
ATTTCTTTGTGTAATGAACGTTGCAATTGTACTCCCTCAGTTATTCTAAAAAGTACAACAAATTAATGCT
GACTGTAGTCACCCTGCTTTGTTGTCAAATACTAGATCTTATTCATTCTTTATTTAACTTTTTAAATTTT
AAACTTATTTTATTTATTTATTTTTAGACGGAGTCTCACTCTGTCGCCAGGCTGGAGTGCGGTGGCGCAG
TCTCAACTCACTGCAACCTCCGCCTCCAGGGTTCAAGTGATTCTCCTGCCTCAGCCTCCTGACTAGCTGG
AACTACAGGCACGTGCCACCACGCCCAGCTAATTTTTGTATTTTTAGTAGAGACGGGGTTTCACTATGTT
GGCTGGGATGGTCTTGATCTCTTGACCTTGTGATCCGGCTGCCACAGCCTCCCAAAGTGCTGGGGTTGCA
GGCGTGAGCCACCGTGCCCGGCCTTTAAAATTATTTAAATCATTTTAATATCTTTTTCATTTCTGCCTC
CGGTCCTGCAGAGTTCTTATTCGTTCTTTCTAAATTTTCTTTGCACCCACTAATCACCTCATTTCCCTTC
TTCTCCCCATTACCCTTCCCAACTTCTGGTAACCATTCTGCTATCTCCATGTGTTCAATTGTTTTTATTT
TTAGTGCCTGCAAACGAGTAAGAATATGCAAAGTTTATCTTTCTGTCCCTGGCTTATTTTACTTAACATA
ATGTCCTCCAGTGCCATCTACATTGCTGCAAATGACAGGATCTCATTCTTTTTTATGGCTGAATGGTAAT
CTATTGTGTATATATACCACATTTTCTTTCTCCATTTGTCTGTCAGTGGACACGTAGGTTGATTCCAAAT
CTTGGCTGTTGTGTATATAGTGCCGTAGTAAACATGGGAGTGCAGATATTCCTTCAATAAACTGATTTCC
TTTCTGAGTATATACCTAGCAGTGCAATTGCTGGATCATATGGTAGCTCTATTTTTAGTTTTTTGAGGAA
TTTCCATACTGTTCTCCATAGTGGTTTTACCAATTTACATGTCCACCAACAGTGTGTGAAGGTTCCCCTT
TATCCACATCGTTACCAGCATTTGTTATTGCCTGTCTTTTGGATAAAAGCCATTTTAACTGGGGTGAGAT
GATATCTTGTTGTAGTTTTAATTTCCATTTTTCTGGTGATCAGTAGTATTGAATACCTTTCATATACCTG
TTTGCCATTCATAAATAACGATGAGGTCTTGCTGTTTGGCCCAGGCTGGTCTCGAACTCCTGGGCTCAAG
CAATCCTCCCACCTTGGCTTCCCAAAATGCTGAAATTATAGTTGTGAGCCACTGCACCTGGCCTTGTATG
TCTTCCTTTTTTTTTTGTTTTGTTTTGTTTTTGAGACAGAGTCTCACTTTGTTGCCCAGGCTGGAGCGTA
GTGGTGTGATCTTGGCTCACTGCGCCCTACACCTCCCGGATTCAAGCAATTCTCCTGCCTCCTGCCACCA

TGTCTGCCTAATTTTTGTATTTTTAGTAGAGACGGGATTTCTCCTTGTTGCCCAGGCTGGTCTTGAACTC
CTAACCTCAGGTGATTTACCTGCCTCAGCCTCCCAAAGTGCTAGGATTACAGGCGTGAGCTGCTGCGCCC
AGCCTGTATGTCGTCTTTTGAGAAATGTCTATTCAGATCTTTTGCCCATTTTTAATTGAGTTACTAAAAT
TTTCCCTATGGAGTTGCTTGAGTGCCTTTTATATTCTGGTTATTGATCCCTTGTCAGATGAGTAGTTTGC
AAATATTTTCTCCCATTCTGTGGGCTGTCTCTTCACTTTGTTGATGGTTTCCTTTGCTGTGCAGAAGCTT
TTTAACTTGATGTGATCCCATTTGTCCATCTTTGCTTTGGTTGCCTGTACTTTTGGGGTATTACTCAAGA
AATCTTTGCCCAGAGTAATGTCCCTGGGAGTTTAATGTTTTCTTTTAGTAGTTTCATAGTTTGAGGTCTT
AGATTTAAATCTTTAGTCCATTTTGATTTGATTTTTTTTTAATATGGTGGGACACAGGGGTCTGGTTTCA
TTCTTCTGCATATGGATATCCAGTTTTCCCAGCACCATTTATTGAAGAGACTGTCCTTTCCCCAGTGTAT
GTTCATGGCTTCTTTGTGGAAAATGAGTTCACTTAGACGTATGGATTCATTTCTGAGTTCTCTGTTCTGT
TTCATTGATCTATATCTTTTTTTATGCCAGTACCATGCCATTTTGGTTACAATAATTTGAAGTCAGATAA
TGATTCCTCCCGTTTTGTTCATTTTGCTCAGTATGGCTTTTGCTCTTTTGGGCCTTTTGTGGTTCCCTAC
AAATTTTAGAATTATTTTTGTCTACTTCTGTGAGGAATGTCATTGGTATTTTGATAGGGATTGCACTGAA
TCTGTAGATTGCTTTGAGTATTATCAACATTTTAGCAATATTAATTCTTCTAATCCATAAACATGGAATC
TCTTTTCATGTTTTTTCTGTGTCATCAATTTCAGTGTTTTAAAGTTGTCATTATAGAAATCTTTTACTCA
TTTGGTTAAGTTTATTCCTAAGTATTTTATTATATTTGTAGCTATTGTAAATGGGATTGCGTTTAAAAAA
TTTTTCAGATTGTTTGCTGTTAAATATAAAAATGCTCCTGATTTTTGTGTGTTGATTTTTGTATCCTGCA
ATTTTACTGAATTTGTTTGTCAGTTCTAATAGGTTTTTCTTTTTTGGAGTCTAGGTTTTTCCAAATGTAA
GATCATATTATCTGCAAACAAGGATAATTTGACTTCTTCCATTCCAGTGTGGATGCTTTTTATTTCTTTC
TGTTGTCTGATTGCTCCAATTAGGACTTCCGAGTATTATGTTGAATAACAATGGTGAAAGTGGGCATCCT
TGTCTTGTTCCAGATCTTAGAGGAAAGCCTTTCAGTTTTTCCCTTTTCAGTATGGTACTAGTTATGGGTC
TGTCATATATGGCTTCTGTTTTGTTGAGGTATATTCCTTCTATACCCAGTTCTTTGGGGTTTTTTTGTTT
GTTTGTTTTTGAGATGGAGTCTCACTCTGTCACCCAGGCTGGAGTGCAGTGGCGCAATGTTGGCTCACTG
CAAGCTCCACCTCCTGGGTTCATGCCGTTCTCCTGCCTCAGCCTCCCGAGTAGCTGGGACTACAGGTGTC
CGCTAACACGCCCGGCTAATTTTTTGTATTTTTAGTAGAGACGGGGTTTCACCGTGTTAGCCAGGATGGT
CTCGAACTCCTGACCTCATGATCTGCCCGTCTCAGCCTCCCAAAGTGCTGGGATTACAGGCGTGAGCCAC
CACGCCCGGCCAAGGGTTTTAATCATAAGGGGATGTGGCATTTTATGTGATATAAATTATATATTTATAT
CATGTGATATATATTTATATCATACACAGTATAAATAATATATATATATATTTTTTAGTCTTTGTCTTTT
ATTCTGTTAAGATGTACCATGTTTATTGATTTGCGTATGTCGAACCATCCTTGCATCCCTGGGATGAATC
CCACTTAGTCATGATGAATGATCTTTTTAATGTGTTACTGAATTCGGTTTGCTAGTATTATATTGAGGAT
TTTTGCATAATGTTCTTCAGAGACACTGGCTTCTAGTTTTCCCTTTTTGATGTGTCCTTTGGTTTTGTAT
AGGGTAATAGTGGCCTTGTAGAATGAGTTTAGAAGTATTCCCTCTTCCTGTATTGTGTTGGAATAGTTTG
AGTAGGATTGGTATTAGTTCTTCTTTAAAGGTTTAGTAGAATTCAGCAGTGAAGCCATCAGGTCCATGGC
TTTTCTTTGCTGGGAGACTATTTCTTATAGCTTTGATCTCGTTACTTGTTATTGGTCTCGTTACTTGTTA
TTGTATTTGGGTTTTGGATTTCTTTGTGGTTCAGTCTTGGTAGGTTGTATGTGTCTAGGAATTTATCCAT
TTCTTCAAGGTTTTCCAATGTATCAGCATATAGATGCTCATAGTAGTCTCTAATGATCCTTTGAATTTCG
GTGGTAACAATTATAATGTCTCCTTTTTCATCTCTCATTTTATTATTTGGGTTTTCTCTTTTTTTTCTGA
GTCTGGCTAAAGGTTTGTCAGTTTTGTTTATCTCTTCAAAACAATTTACTGTTTTATTGATCTTTTGTAT
TTTCTTCATTTCAATTTTATTTATTTCTGCTTTGATTTTTTTTATTTCTTCTACTGATTTTAGGTTTTGT

CCTTGCTTTTCTAGTTCTTTAGGATGTATTGGCAGATGAAGTTTTTCCACTTTTTTGATGTAGGCACTTA
CTGCTGTAAACATTCCTCTTATTGTTGCTTTTACTGTATCCTATAGGTTTTGATAAGCTGTGTTTCCATT
TTCATTTGTTTCAAGGAATTTTCCAGTTTTCTTCTTAATTTCTTCATGGACCCACTGGTCATTCAGGAGC
ATATTGCTTAATTTTCATGTATTTGTATACTTTCCAAAGTTCCTCTTGTTATCTAGTGTTATTTTATTTT
ATTTTTATTTTTGTTTTTTTGAGATGGAGTCTCGCTCTGTCACCCATGCTGGAGTGTAGTGGCGCGATCT
CGGCTTACTGCAACCTCTGCCTCCCCAGTTCAAGTGATTCTTCTGCCTCAGCCTCCTGAGTAGCTGGGAT
TACAGGCATGTACCACCACTCCTGGCTAATTTTTTTTTGTATTTTTAGTAGAGAGGGGGTTTCACCATGT
TGGTCAAGCTGATCTCGAACTCCTGACCTCAGATGATCCACCCACCTTGGCCTCCTAAAGTGCTGGAATT
ACAGGCATGAGCCACCGTGCCCGGCCTCTAGTGTTATCTTATTGTGATCAGAGAAGATAGTTGATATGAT
TTTAACTTTTTTGAATTTTTATTTATTTATTTGTTTGTTTGTTTGTTTGTTTGTAACAGAGTCTCACTCT
GTTACCCAGGCTGGAGTACATGTCATGATCTTGGCTCACCTGCAACCTCCGCCTTCCTGGCTCAAGCAAT
CCTCCCACCTTAGCCTTCCAAGTAGCTGGGACTACAGGCACATGCCGTCACATATGGCTGATATTTTTGG
ATTTTTTTTTTTTTTGTAGAGATGGGGCTTTGCGATGTGTCCCAGGGTTGTTTCGAACTCCTGAGCTCAA
GCAATCCACCTATTTCGGCCTCCCAAGGTGCTGGGATTACAGACATGAGCCACTGTGCCACGTCAAATCT
TTAGACTTGTTTTGTGGCTTAACATAGGGTCTATCTTTGAGAGCAATCCATATGTTGAGGAGAAGAATGT
GTATTCTATAGCTGTTGGACACAATGTTCTGTAAATATGTATTGGGCCTATTTGGTCTATAGAGCAAATT
AGGTCTAATGTTTCTTTGTTGATTTTCTGTCTGAATGATCTGTCCATTGCTGAGAGTGGGGTGTTGAAGT
TTCCGACTGTTACTGAGGTCTGTTTCTCTTTTTTGCTCTAATAATGTTTGCTTTATATATCTGGATGCTC
CAGTATTGGTTGCATATGTATTTATACTTGTTATAACCTCTTGCCGAATTGATCCCTTTATCATTATACA
ATAATCTTCTTTGTCTGTTTTTATAGACTTTGTCTCAAAATCTATTTTATCTAAGCATAGCTACTCCTGT
TCTTTTCTGGTTTCCATTTGCATGGAATATTGTTTTCCAGCTCTTCAATTTTAGTCTATGTGTGATTTTA
TAGGTAAAGTGTGTTTCTTGTAGGCAATGGATCTTTGGTTTTTTTTTTTTTTTTTTTGAGACAGAGTTTT
GCTATTGTTGCCCAGGCTGGAGGGCAATGGCGCTATCTCAGCTCACTGCAACCTCCGCCTCCTGAGTTCA
AGCGATTCTCCTGCCTCAGCCTCCCAAGTAGCTGGGATTACAGGCGCCTGCCACCAAGCCCAGCTAAATT
TTTTGTATTTTCAGTAGAGATGGGGTTTCAGTATGTTCGTCAGGCTGTTCTTGAACTCCTAACCTCAGGT
GATTTGCCTGCCTTGGCCTCCCAAAGTCCTGGGATTACAGGCGTGAGCCACCGCACCCAGCCTTTTTTTT
AAATCCATTTAGCCACTCTGTATCTTTTGATTGGAGAGTTTAGTCGATTTACATTCAGTGTTGTTACTGA
TTAGTGAGGACTTAACTACTACCATTTTGTTACTTATTATCTGGTTGTTTTGTAGTCCTACTCCCTCCCT
TCCCCCTTCTTTTTTACTTCCTCTTCGCTCCTTTTTTCCCTCCCTCCCTTCCTTGTTTTGAAAGTGATTT
TCTCTGGTGGTATGTTTTAATTTCCTGCTTTATATTTTTTGTGTATCTGTTGTAGGTGTTTTTGATTTAA
GATCACCATGACAGCTGGGTGCAGTGGTTCACACCTGTAATCCCAGCACTTTGGGAGGCCGAGGTGGGTG
GATCAAGAGGTCAGGAGATTGAGACCAGCCTGGCTAACATGGTGAAACCCCATCTCTACTAAAAATACAA
AACTTAGCCAGGCGTGGAGGCACGTGCCTGTAATCTCAGATACTCAGGAGGCTGAGGCAGGAGAATTGCT
TGAACCCAGGAGGCAGAGGTTGCAGTGAGTCAATATTGTGCCACTGCACCCCAGCCTGGGCGACAGAGTG
AGACTCCGTCTCAAAAAAAAAAAAAAAAAAAGAGATCACATAAGGGTTGCAAATAACATTTTATAACCCAT
TATTTTAAACCAATGACAACTTGAAACTTTGATTGCAAAAACAAGCAAGCAAAGAGAAAACTAATAAAAA
CTCTACACTTCATCTGCCCGCTTTTTAACTTTTGTTGTTTTTATTTATATCTTTATTATACTATGTCTTA
AAAAACTGTAGTTATAAGCCAGGCGCAGTGGTTCACGTGTGTAATCCCAGCACTTTGGGAGGCTGAGGTG
GGCGGATCACCTAAGGTCAGGAGTTCGAGACCAGCCTAGCCAATATGGCAAAACCCCCTCTCTACTAAAA

ATAGAAAAATTAGCCGGACATGGTGGCGGGTGCCTGTAATCCCAGCTACTCGGAGGCTGAGGCAGGAGAA
TCACTTGAACCCAGGAGGCCCAGGTTGCAGTGAGCCGAGAGTGCGCCACTGCACTCCAGTCTGGGCAACA
GAGTAAGACTGTCTCAAAAAACAATACAAAACAAAACAAAACCCTGGCCTAGTGGCTCACGCCTAATCCC
AGCACTTTGGAAGGCAAAGGTGGGGCGAATCACAAGGTTAGGAGTTCGAGACCAGCCTGACCAACGTGGT
GAAACTCTGTCTCTACTAAAAATACAAAAATTAGCCAGGCGTGGTGGCACGCACCTGTAATCCTAGCTAC
TCAGGAGGCTGAGGCAGGAGAATCGCTTGAACCTGGGAGGCGGAGGTTGCAGTTAGCCGAGATCGCGCCA
CTGCCGTCCAGCCTGGGCAGCAGAGCAAGACTCTGTCTCACAAAAAAAAAAAAAAATTGTAGTTCTTATTT
TTGAAAGGTTCATTTTTTATTCTTCCTGCTCAAAATATGAGTAGTAGTTTATACACCACAATTACAGTGT
TACAATATTCTGTATTTTTCTGTGTACTTGTTACCAGTGAGTTTTTGCACCTTCAGGTGATTTATTATTG
TTTGTTAACATCCTTTTCTTGCAGATTGAAGAACTTTTTTTTTTTTTTTTTTTTTTGAGACAGAGTCAT
GCTCTGTTACCAGCCTGGAGTGCAGTGGTGCCATCTTGGCTCACTACAACCTCCAACTCCCAGGTTCAAG
CGATTCTTCTGCCTCAGCCTCCCAAGTAGCTGGGATTACAAGCATGTGCCACCACGCCCAGCTACTTTTT
GTATTTTTAGTAAAGACGGGGTTTTGCCATATTTGCCAGGCTGGTCTTGAGCTCCTGACCTCAGGGTGAT
CCGCCCGCCTTGGCATCCTAAAGTGCTAGGATTATAAGCGTGAGTCATCGTGCCCAACTTGGTTGTTTAT
TTTCAAATAGCCTGAATTCAAGCTCACTAATGTTTTCTGCTGCTTGATACATTTCTGCTATTGAGAGACT
GATGCATTTTTCAGTTTGTCAATTGAATTTTTCCACTTTGGGATTTCTGCTTGATTCTTTTTACTAATAA
TTATTGCAGTCTCTTTTTTAAATTTATAGGATTCTGAATTTGTTCTCTGTATTATCTTGGATTTCGTTGA
ACTTTCTCAAAGCATTCAGCTTGAATTCTGTCTGAAAGTTCACATATCTCTTATCACTTGGGAATTGGTC
ACTGGTGTCCTTTATTTTTAGTTCATTTGGTGAGGTCATGTTTTCTCAGATGGCCTTGATGCTTGTGGAT
GTTCATCAGTGTCTGGGCATTGAAGAGTTGGGTATTCTGTTCTTTGTAGTCTGGTTTTGTTTGTACGCAT
TCTTTTTTTTTTTTTTCTGTTTTTGAGACAGAGTCTCGCTCTGTCGCCCAGGCTGGAGTGCAGTGGCACA
GTCTTTGCTCACCGCAACCTCCGTCTCCCGGATTCAAGCAATTCTCCTGCCTCAGCCTCCTGAGTAGCTG
GGATTACAGGTGCGTGCCACCACGCCTGGCTAATTTTTGTATTTTTAGTAAATATGGTGTTTCACCATGT
TGGTCAGGCTGGTCTCGAACTCCTAACCTCGTGATCTGTCCGCCTTGGCCTCTCAGAGTGTTGGGATTAC
AGGCGTTAGCCACTGCATCCGGCTCCCATTCTTCTTGAGAAGGTTTTTCAAGTATTCAAAGGGAATTAAG
TGTTGTCATCTAAGTCTTCGCTCACTGCAGCCATACATGCATTAGAGGGCACCCCAAGACTAGTAATGTT
GTGACTCTGTAGAGGTATCACCTTGGTAGTCTTGGGGAAGATCTGGGAGAATTCCCTGTATTACCAGGCA
GTCTCTTGTCCTCTTACATTTCTCCAAACAAATGGAGTCTCTCTTTGTGCTGAGCTGCTTGGAGTTTGGG
GAAGGGTGACACAAGCACTGCCATGGCCACCGTCACTGGAACTGTACTTGGTCTCACCCAAGGCCTGTGG
CAGCTATTTTCTGGCCACCACTGATGTTAATTTAAGGCCCAAGGGTGCTTTAGTCAGTAGGTGAAGAATC
CTGCAAGAACTGGGTCTTTACTTTCAGTGCAGCAGGTTCCCTTCTGGCCCAGGGTGTGTCTAGAAATGCT
GCCCAGGAGCCAGGGCCTGGGATCGGGAGCTTTAGGAATCTGCTTTATTGTACTGGGGCTGAGCTGGCAC
CCACTTGCAAGATAAAGTCCTTTTTACTCTTCTCTCACCTCAAGCAGGTGGGTCTCCCCATGGACACCAC
AGCTGTGAATGTGCGGGGTCATATCTGAAGCTGGCACAATACGACATGGCACCTTGTTTTTTATTCAAGG
CACAAGGGCTCTTTAGTCAGCTGGTGGTGAATCCTACTAGGACTAGGTATTTCCCTTCAAGGCAATGGGT
TCCCTTCTGGTCCAGAATATGTCTAGAAATGTCATCTGGGAGCTATGGCCTAGAATTGAGGCTTCAGAAC
TATGCTTGGTGCTTTATTTTACTGTGGCTGAACTAGTATCCACATTGCAAGACAAAGTCCTCCCTACTCT
TCCCTCTCCTCCCAGAGCTGTGAGCTGTGGTACCTGGAGTTGGGGGAAGGCTGGCACAAGCACTCCCTTG
GCCACCCTAGCTGGTGTCTCAGTGGGTCACATGTACCCCAAGTCCACTGACTATGAGCCCAGCACAGTAC

CATGACTTGTCCAGGAATTGCAGTCCTTCTGGTCTAGACTGCCTTTCAAGTTTATTTAGGACCCCAGAGG
ACTTTACCCACGGTGGTGGGGCTTACCAAAATTAAGATTCTTTTGGTTTTTTTTGGCAGAGTTTCGCTCT
TATTGCCCAGGCTGGAGTATAGTGACGCAATCTCAGCTCACCACAACCTCCGCCTCCCGGGTTCAAATAA
TTCTCCTACCTCAGCCTCCTGAGTAGCTGGGATTACCGGCATGCGCTACCACCTCTGGCTAATTTTTTTG
TTTTTTAGTAGAGATGAGGTTTCTCCATGTTGGTCAGGCTGGTCTTGAACTCCCGACCTCAGGTTATCCG
TCCGCCTCGGCCTCCCAAAGTGCTGGGATTACAGACCATAGTGCCCAGCCCGAAATTCAGATTCTAATCA
CTGGGATGGACAATTCCCCTCTGACTAGGGCTAGTCTAAATACTCCCTCTGTGGGTGCTGGCTGAATTCT
GTCCTATGCTGCTTTCCACTGTGACAGGGCAGCACTGAGTTTCAATGCAAAATCCCACAGTCATTTCTCT
CTCTCTCCCCCGAGCACACAGATTCTTTCTCCACCCCACACTGCATTGTGGGGGAATGTCAGGGGTGTTG
GAGGGGCAGTTCAAGACTATCTTCCTTATCTTTTTTGGTGTCTTTTTCCTTGATAGGATGTCAAAACTGG
GTACTGTGATCGCTTACCTAATTTTTGGTTCTTATGAAGGTGCTTTCTTGTGTGGATAGTTGTTCAATTT
GGTGCTCCTTGTTGGGGATGATCACTGGAAGGTTCTGTTTGGCCACCATGCTCTGTCTCTTCTCCCCTGC
CATCTCCTTTTTTTACTTAGGGGTTTAGAATGTCTAACTGACCAATATGTACAGTCAGGTCTCATTCTGA
ATTCACCTACTTAATGACCTTCCAAGCTGACTAGGCCCAGCGCTTAGTCCAGCCTCCATGACGGTCCCTC
CACATCCTAATTAGCCTCCCTCCAGTTCATTTCACACAAAGCTGCTGTGTTCACCTTTCTGAACTATAAA
TCTGCCCAGTACTCTACCCTACTTAAAATTCCGTATAGACTGCCCATTTGCCCTGAGAATTAAAAGCCAA
AGTCCTAAACGTAGCTTTTTAAAACTTTTTTTTTTTTTTTTTTAATTTTTAGATGGAGTCTTGCTCTGTC
ACCCAGGCTGGAGTGCAGTGGTGTGATCTTGGCTCACTGCAACCTCCGCCTCCTGGGTTCAAGCAATTCT
CATATGTCAGCCTCCCAAGTAGCTGGGATTTACACGTGTGCCATCACGCCTGGCTAATTTTTTTTTTTTA
TCTTTAGTAGAGACGGAGTTTCACCATGTTGGCCAGTCTGGTCTTAAACTCCTGACCTCAAGTGATCCAC
CTGCCTTGGCTTCCCAAAGTGCTAGGATGATAGGTGTTAGCCACTGCACGCAGCCCTGAACATAGCTTTT
AAGTTCCTTTATTGTCATATTCCTTTTGACGAGTCTATCATTTTCTGACTCACTTGTACATGTGTGTCTC
ACCCTTGGTCCAGCCATTGGTGCTTTTCTTTACTTCTTTATTTTTGTTATTTTATTTTATTTTATTATTA
TTTTTTAAATGAGACAGGGTATCACTATGTTGCCCAGGCTGGTCTTGAACTCCTGAGCTTAAGCAGTCTG
CTTGTCTCAGCCTCCCAAAGGGCTGGAATTACAGTGATGAGCTACTGTGCCCAGCTCATTGGTGCTATCT
TTTTTTTTTTTTTTGAGACGGAGTCTCGCTCTGTCACCCAGGCTGGAGTGCAGTGGCGTGATCTTGGCTC
ACTGCAGCTCCACTTCCCAGGTTCACACCATTCTCCTACCTCAGCCTCCCGAGTAGCAGGGACTATAGGC
GCCTGCCACCATGCCTGGCTAATTTTTGTATTTTTAGTAGAGATGGGGTTTCAGCGTGTGAGCCAAGATG
GTCTCGATCTCCTGACCTCGTGATCCGCCTGCCTTGGCCTCCCAAAGTGCTGGGATTACAGGCGTGAGCC
ACCGTGCCCGGCCCCCATTGGTGCTATTGTTTTATGTGATAGAGCCAGCTTCTCCCTTTTCTTTGGATTT
TTAAACATACTCTTCCTTTTACTTAGACTATTCTCCATCCCAACACCTTTCCTAAACTTCTTTCACACCT
TAGACTAGCTGACACTTTACTGAGAAACCTTTCTTTTTTATAGGTTGCTTTTTCTATAGACTCTCTTAGC
ATTTACTCATTTTATTGTGAAGTGTCTGATCTTATTTAAATGACAAGTATAAGAGGATAGAAACTATTTC
ATATTTTTCTCACCCAGCAGGCACAATTTCTGACATGTGGTAAGCACTCAGTAAATATTGAACTTTAGAG
GCTAGGACATTTGAGTGCTTTGGTGACTGTGGTTGTGCTATATAGGTACTCTGTTATTGTTAGTTTATAG
TAAAAGCATTACTCTTAAAGTATGAAAAAAGCCTTATTCAGAACATTTCATGCGTATAGTTAATATTACG
TAGCTTGTGCTCATGGCAAAAATGTATTACTAAAGTTATTTAAGATATTTAAGTATAATTGTTTCCTTTA
TTTAGTTACAGCCAAGTTCTACTTCTGAATCTATGGATCAACTACTAAACAAAAATAGAGAGGGAGAAAA
ATCAAGAGATTTGATCAAAGACAAAATTGAACCAAGTGCTAAGGATTCTTTCATTGAAAATAGCAGCAGC

Usually constitutive exon 15, encoding IDR2 fragment (green letters), with residues
LQPSSTSESMDQLLNKNREGEKSRDLIKDKIEPS
AKDSFIENSSSNCTSGSSKPNSPSISPSILSNTEHK
RGPEVTSQGVQTSSPACKQEKDDKEEKKDAAE

AACTGTACCAGTGGCAGCAGCAAGCCGAATAGCCCCAGCATTTCCCCTTCAATACTTAGTAACACGGAGC
ACAAGAGGGGACCTGAGGTCACTTCCCAAGGGGTTCAGACTTCCAGCCCAGCATGTAAACAAGAGAAAGA
CGATAAGGAAGAGAAGAAAGACGCAGCTGAGTGAGTAAACCTGGAACTTAGACCATCCTGTTACTCAATT
AACTTTTTTTTTTTTAAAGGCATTTAGGTCCTTCCAACTGTGAAGAATCCATCTGGACTTTTAGACTACT
TTATACATTGCCCTTAGTTTACAAACAGCTAGTCCAAACAAATGACATCTTAAGTAAATGAGGTTATTGC
ACCCTGTGCTACTCTTCTGTTCTTCCCCTTTTTTGTACCCCAGGGCTAGAAAAACAAGGCATAAATTAAG
AAAAGTTTTTCTGTAAATGAACAGGAGTTGAAAAATTATCAATTCAGGGGACCTATCTTTACTGGATTCC
ACTCATTAGTCACCCTCACTGTGCTGCTAGGTTGAAAAACTGCCACTGTCAAGGAGAGAAGCATGCGGTG
CTTCTACTTGGAATTCAAAATATTTTTCATCAGAAACTGTGTTTTAGTTAATGTTTAGATTTGTTAAGAT
AGACTTAATTCTGCACATTCAGTATATTAATTAAATGGACTTTTAGGGGCTAACCTCAGAACTTAACTAC
CATTGACTTAGGTGTTTGGGTACCAAACAATCCAGTTAAAGCTGAAGTTTTGGAATGCAGCTTATTGATA
AATTGGGGACTGCTTATTCTTGATTTGAGGCAATTTTTTTTTACAGCCATGACTTTTTCCAGGTATGTCA
TGTAAAATATCTTCTCACATAAGAATTACTGCATGCTAGAATATTGGTATGTTGACTGGTAGCTCATACC
TATAATCCCAGCACTCTGGGAGGTCCAAGCAGGTAGATTACTTGAGGTTAGGAGTTGAAGACCAGCCTGG
CCAACATGTGAAACCCTGTCTGTACTAAAAATACAAAAATTAGCCAGGCATGGTGGTAGGTGCCTGTATC
CCAGCTACTCGGGAGGCTGAGGCAGGAGAATTGCTTGAACCCAGAAGGTGGAGGCTGCAGTGAGCCGAGA
TCATGCCACTGCACTCCAGCCTGGGTGACAGAGCGAGACTCTGTCTCAAAAATAAATAAATAAATAAATA
AAAGGATACTGTTATGTTAAGAATTGCTTTTAAGGATATTTCATAAGTAGCTACTGTCTTTTCAGCTCAA
GTGTTTGTTGATTGGCCAGGCGTGGTAGCTCATACCTGTAATCCCAGCACTTTGGGAGGCTGAGTCAGGC
AGATCACTTAAGGTCAGCGTGGCCAAAATGGTGAAACCCCATCTTTACTAAAAATAAATATTAAAAAAAA
TTAGCTGGGCGTGGTGGCAGTCTCCTGTAATCCCAGCTAATCAGGAGGCTAAGGCAAGAGAATGGCTTAA
ACTCGGGAGGCAGAGGTTGCAGTGAGCCAAGATTGCACTGCTGCACTCCAACCTGAGCAACAGAGTGGGA
CTCTGTGAAGGAAAAAAAAAAAGTATTTTTTGATTGCCTTTGAGAGGAACGGTTGTATATTACTCAGATT
TTTAAAAAATTGTTCTTTTATGGCTGTATTCTTTAAGGGATTAAGGAATGGGCAATATAAGTGTATATGT
TTCAATAAAAACGATTAGTGATCTTCTAGTGAGAACAGTTTAAATCTATATTTAGCAATTTTTTTTAAAT
TGTCAGGTATGGAAGATTTTAGAGCAACGTAAAGTCCATGTAGATTTCACTGGCCTTTATATTTTTTTTA
GGCAAGTTAGGAAATCAACATTGAATCCCAATGCAAAGGAGTTCAACCCACGTTCCTTCTCTCAGGTAGG
TTTATTACTTTCTTTGAGGTTATCTAGTCCCAAAAAAAGAAAAATTATTAGTAATAGTCCTTCTTCCATA
CCTGCCATCTGAATTTTGTTTTAGTGTGCTGAACCAACCTTCTTTCTTTTTTTTACATGGCCATTAATGA
ATACTTTTTAAACATTAAAAAAAGGTCTTTGTTTTGTCATCAATTAGATGTGATCTTGGGCAAATCTTTG
AATTTCTCTGACCCAGAATTTGACGATGGTTGGCTAGCTAGGCTGTCAGGTTTATAGATACGTCCTCTGC
ACCTGAGGGTTTTGCATCACTGGATTCAACCAACCATGGATCAAAAACATAGTTAGGATAATCTATACTG
AACACATGCAGACGTTTCCTTGTCATTATTCCAAAACAATACAGTAAAGCATTTACCTTGTTTTAGGTAT
TATAAATAATCTAGAGATGATGTAAAGTATATAGGAGGATATGCATAGGTTGTATGCGAATACTACATGA
TTTTATGTAAGGGACTTGAGCATTCCAAGACTTTGGTATCTTCACAGGGTACTGTAACCAATCCCCCACA
GATACTAAGAGATGACTGTACTATTGTTATTATTCGACTGAGATCATAAGAAGATATATTTATTTTTAAT
TTTTAAAAACACTTCCATCAGTTTCTTAAAAATAGCTGCCACTGTTTTTAATATTTTTTAATTGACAAAG
TTTTAAGTTCCTACTGAAACATTTTTTCTTTTATTGAAATGTGAAAATTTATGTGCTGTGTTTTTGTTTT
CAATAAAAGGGACATAGTTAAAGCAAGTAAAATTAGAAAGACTGGGAAAATCCGTCTTTAAATTGCAATA

Usually constitutive exon 16, encoding intrinsically disordered sequence that contains PAM2 motif (in bold letters), with residues
QVR**KSTLNPNAKEFNPRSF**SQ

ATAGTTCATCTGTTACCTTGAGATAATTGAATTTATTGTTGTTTTTGTAGCCAAAGCCTTCTACTACCCC
AACTTCACCTCGGCCTCAAGCACAACCTAGCCCATCTATGGTGGGTCATCAACAGCCAACTCCAGTTTAT
ACTCAGCCTGTTTGTTTTGCACCAAATATGATGTATCCAGTCCCAGTGAGCCCAGGCGTGCAAGTAAGTC
ATAGAATTTGATGTTCACTTAGCCTCCCCAATTGTTTGTATCTGACACCAAGCACTCTTTAGGTTTTCAG
TGACTTGAGGGTGTGATGGTTATGCATATGCATTTGAAACAGACAGGCATGCAGAGATTCAGTGTGTTGT
TAAGTATGAGGACCTAAATCTGAGAATGTTTTCTGTGAAAAAGATGGTTTAGATTTACTGTAGTTTGGGG
TTTGTTCCTTTTAGCTGTGGGTATGATCTAATTTTTTAATGACTAATGGAGAATCAGGAAACCTTCTCAT
GCCTAGCTCTCTAGCAATATAAAACTAAGAGTGACAGAATACCTTGTTATTATCATAGGTGCCTAATGTT
AATTTTTTTTTTAATTCTCTCAAGCCTTTATACCCAATACCTATGACGCCCATGCCAGTGAATCAAGCCA
AGACATATAGAGCAGGTAAAGGTGAGAATAATCCTGCCTGTGTTTGCTTGTAGTTTGCATGCTGCATGAA
TTGAGTAACTAAGTTTATAATGAATAAATAGTTGTAGTTTAGCTCTGACTTTTTGATGAGGCTATGCATT
GGCTTTTGATGAACAACATTACATAGATATTCACATGGATTTTATGAAGAAAAACAGGGGAGAAAAAATG
CCCATCAGTTGTGATTATATAGTATCCTCTTCAAAAAGAGTAATTGGAGGCCTGGTGTGATGGCTCACAC
CTGTAATTTTAGCACTTTGGGAGGCCAAGGCAGGAGGATTGCTTGAGCTCAGGAGCCCAAGATCAGCCTG
GACAACAGAGACTTTGTCTCTACTAAAATTCAAAAAAATTAGCTGGGCATGGTGGCATATGCCTGTAGCC
CCAGCTGTTTGGGGGACTGAGGCGAGAGGATCACTTGAGCCCAGGAAGTAGAGGCTGCAGTGAGCTGTGA
TTATGCCACTGCCCTCCAGCCTGGGCGACAGAGTGAGACCCCGTCTCAAACATAAATACTGGCTGGGCAT
GGTGGCTTATGCCTGTAATCCCAGCACTTTGGGAGGCCGAGGTGGGTGTATCACCTGAGGTCAGTAGTTT
GAGACCAGCCTGGCCAACATGGCGAAACCCCATCTCTACTAAAATACAAAAATTAGCCGGACATGGTGGC
ACCTGCCGCCTGTAATCCCAGCTACTAGGTGGGGCTGAGGCAGGAGAATTGCTTGAACCCGGGAGGCAGG
GGTTGCAGTGAGCCAAGATCGTGCCACTGCACTTCAGCCTGGGCAACAGAGTGAGACTCCATCTCAAAAC
AAACAAACAAACAAAAAACAAACAAACAAAAAAACCAGACTAATTGGCTGGACACAGTGGCTCCATGCCT
GATATCCCAGCTGGAGGATGACTTGAACCCATGAGTTCGAGAGCAGCATGGGCAATATAGTGAGACCCTA
TCTCAAAAAAAAAAAAAAAAGTTAATTCCAAAGCTTTTTGATCTGAAATCTGATTTAAATCTGAACTTAA
ATTTGAAGAAGAGGGTTTGCTAGATTAATTTACTAGATTGCTAACCTTGCTTTATATATACCTACAGTTA
TTTCCCCAAAGCCAGAATTTCTTTTGAAGCAGAGGGGCAACTAACTTCAACCAATGTTAAGATCCTATTA
GAAGGATGTTTCGGCTAGGCTTGGTGGCTCACGTGTAATTCCAGCACTTTGAGAGGCTGAGGTGGGCAGA
TCACATGACCGGGAGTTTTAAGACCAGCCTGGGCAACATGGCAAAAACCTATCTCTGCAAAAAAAAAATA
GAAATCTTAGCCAGCCGTCATGGTGTGCTCCTGTAGTCCTAGCTACTTGGGAGACTGAGGTGGGAGGATC
AATTGAAACCAGAAGGTCCAGGCTGCAGGGAACTGTGACTGCACCACTGGGCTCCAGCTTGGGTGAAAGA
GCGAAACCCTGCCTCAAAAAGAAAAATAAGATGGATGTTTCTGCATTAAAATTAGGGAGTTGTCGTATAA
TGTAGTTGCATAAACTAGTATTCTGTGCTTGTGTGGTTAAAGAGCCTTCGTAGAAAAAATCCCACATTTT
TCTTAAAAGGAAATCTTTTGGCCAGGTGTGGTGGCTCACATCTGTAAGCCCAACACTCTGGGAAGCCGAG
GTGGGCAGATCACTTGAGGTCAGGAGTACAAAACCATCCTGGCCAACATGGTGAAAACCCGTCTCTACTA
AAAATACAAAGATCAGCTGGGCATGGTGGTGCGTGCCTGGGTGACAGAGCGAGACTCCGTCCAAAAAAAA
AAAAAAAAAAAAAGAGTTCTTTTAATGTTGGAAAATGCTAAAGGGTTTTTTTTTTGCCAACCAGTTAATT
TAGAGTGATTAACTGCTATCAGTTGAGAAACTATAGAAAGTAGAATAATTTATACAGAAAAGACATTTCT
CAGTGCCCAATAATTGCCTTTCTGACATAAAGTTTTCATTTTTCCTGAATTAATAAGATTTCCTCAATGT
GTTTTTTTGGGTGTTTTGTGTGTGTGTGTGTGTGTGTGTGTGTGTGTGTGTGTGTGTGTGTATGTGTTTG

Usually constitutive exon 17, encoding first intrinsically disordered sequence (green letters), then structured sequence, with residues PKPSTTPTSPRPQAQPSPSMVGHQQPTPVYTQP VCFAPNMMYPVPVSPGVQ

Usually constitutive exon 18, encoding structured sequence, with residues PLYPIPMTPMPVNQAKTYRAgk

These final two amino acids GK are alternatively spliced

ATACAGGGTCTTGCTTTGCTGCTGAGGCTGGAACGCAGTGGCGCTATCATGGCTCAATGCAGCCTTGACC
TCCTGGGCTCAAGCGATCCTCCCTTCTCAGTCCCCTGGATAGCGGGGGCTACAGGTGCACACCACCACAC
CTAGCTAATTTTTGTATTTTTTGTAGAGATGGGTTTTGCCATGTTGCCTAGGCTGGTCTCAAACTCCTGG
GCTCAAGCGATCTGCCTGGCTCTGCTTCCCAAAGTGCCTGCGCCCAGCCAATTTTCTCCATGTTTGACCT
AATTGTGATTTCATAGATGTTAACTAAAACTCTTAATTTTCGTTTTCTCAGTATGCTATTTTTTTTTTTT
TTAGCCTTGGAACATATGAACCTGTTGAAAGAACTCTGCCTGAAATAATGTAATCAAATTATAGAGTTTA
ATCTTATTTTGAGGGCCTTTAGAAATTCTGAGAAGAAAGTGGGTTTTTTTTTTTACTGCCATTTTAATGT
AGTGTTAAGGTGTTCATGTATCACCAGCAGGTGTAGCTGTTTTCAATGATTACTTAAAACAATGCAATGG
GAACTTTTTGTTGTCATTAAAATATAAAAGGTTACTGTAGTAAGAGCAAGCATGACAGTTTGGCTATCTG
ATGGGAGAGTCACATTCTAACTTCAGGAGGTACTGTCTTTTTAATAGAAATGATATACTCAGAGTCTGGG
CACGGTGGCTCACGCCTGTAATCCAGCACTTTGGGAGGCCGAGGTGGGCAGATCACGAGGTCAGGAGATC
AAGACCATCCTGGCTAATACAGTGAAACCGTGTCTCTACTAAAAATACAAACAATTAGCTGAGCGTGGTG
GCAGGTGCCTATAGTCCCAGCTACTCGGGAGGCTGAGGCAGGAGAATGGCATGAACCTGGGAGGCAGAGC
TGGCAGTGAGCTGAGATGGTGCCACTGCACTCCAGCCTGGGTGACAGAGCGAGACTCCGTCTCAAAAAAA
AAAAAAAAAAAAAAATAGTAGAGAAAGGGCTTTGCCATGTTGGCCGGGCTGGTCTTGAACTCCTGGCCTC
AAGTGATCCACCTCCCTCGGCCTCCCAAAGTGCTGGGATTACAGGTGTGAGCCACTGCTCCTGGCCTGAA
TATACCACTTTTACCTATCATCAGTTGATGAACATTTGGATTATTTCCTTTTTCTGGCAATGAGTAATGC
TTTTGTGGATTTTCATGTACAAATTTTCATATGAGGCTGGGAGCAGTGGCTCATGCCTATAATCCCAGCA
GTCTGGGAGGCTGAGGTGGGCAGATGACTTGAGGTCAGGAGTTTGAGACCAGCCTGGCCAACATGGTGAA
ATCCCATCTCTACTAAAAATACAAAAATTACACTGGCATGGTAGCGTGCACCTATAATCCCAGCTATTCA
GGAGGCTGAGGCAGGAGCATCAGAATCGCTTGAACCTGGGAGGCGGAGGCTGCAGTGAGCTGAGATCACA
CCACTGCACCCCAGCCTGAGTGAAAGAGTGAGTCTCAAAAAATAAAAAATAAAATTTTTTTTCATGTGGC
CTTAGATTTTCATTTCTCCTAAAGTAGAAATGCTGTGATGGAACTGCCAAACTTTTCCAAAGCAGCTGCA
TCATTTTGTATTTCTACCAGTAATGTACAAGTGTTCCAGTTTCTCCACATCCTCATAAATAACCGATATG
TCTTTGGTTTGGGTTATGTCCATTCTAGTGGTTATGAAGTGTCATTGTGGTTTTTTGTTTTTTTGTATTG
TTTTGAGATCGTGCCCAGGCTGGAGCACAGTGGCACAATCTCGGCTCACTGCAGCCTTCGCTTCCTGGGT
TCAAGCAATTCTCCTGCCTCACCCTCCCAGATAGCTGGGGCTGCAGGCATACGCCACCACACCAGGCTAA
TTTTTATATTTTTTGTAGAGATGGAGCTTCTCCGTGCTTCCCAGGCTGGTCTCGAATTCCTGAGCTCAAG
CGATCCCCCTGCGTCAGCCTCCAGAGTAGCTGGGGTTATAGGCGTGCACCACCGCGCTCGGCCCATTTTT
GTATTTTTAGTAGAGATGGAATTTCACCATGTTGGCCAGGCTGGTCTTGAACTCCTGACCTCAAATGATC
CGCCTGCCTCACCTTCCCAAAGTGCTGAGATTTTAGACGCGAACCACCATGCCCTGACTATAGGTTATCT
TTTTACTTGCTTGATGGTGTTCTTTGTAACACAGTTTTTAATTTTGATGAAGTTCAATTTATCTGTTTGT
TTTTTCTTTTGTTGCTGTTGCTCCTGATGTCATATCAGACAAAGCATTGCCTAACTCAAGGCCACAGAGA
TTTACTCCTATGAAACGCCTATAAAACTCCTATGATTTTTATAGTTTAGCTCTTAACATTTAAGTCTACA
ATCTCTTTTGAGTTAATTTTTGTGTATGAGATGAGAGTAGTGGTCCAGGTTTTTCCTTTTGCTTGTGGAT
ATCCGTTGTCCCCACCTCATTTGTTGAAAAGACTATTCTTTCCTCTTAAATTGTTTGTTTGTTTATTTAT
TTTTGAGATGGAGTGTCGCTCTGATGGAGTGGCGCTAACTTAGCTTCACTGCAACCTCCGCCTCTCAGAT
TCAAGCGATTCCCCTGCCTCAGCCTCCTGAGTAGCTGGAATTACAGGGGTGCGCCACCACACCCAGCTAA
TTTTTGTATTTTTAGTAGAGACGGGGTTTTACCGTGTTGGTCAGGCTGGTCTCGAACTCCTGATCTCGTG

ATCTGCCTGTCTCCTGGCACCCTGGGAGGCTGAGAGGCTGAGGTGGGAGGATCACTTGAGCTCAGGAGTT
TGAGACCAGCCTGTACCATTATGCCTGGCTAATTTTAGAATTTATCTTAAAGTATAAAATGTGAATCCAA
TTTATCTTGTTCTAAATGACTATCCAAAATGTTTTAACCAGTTTTATTAGTCTGTAATTTACATACAAGA
AAATGCTCATCTTTTTATGTTTACATTTTAATGAGTTTTGACAAATATATTTGCTCATGTAACTACTTGC
TTCATCAGTGAAGATGGAAAACATTGTGCCTGTTCCTCTTCTCTGTCCAACTGTACTTTATTACCACTAG
CTCCAGTTAACCAGTAATCTGCCTTCTTTTACTATAGATTAGATTTATCCTCTTTAGATTTCTTTTTCTT
TTTTTTTTTTGATTAGGTTTTTTTTTTCTTTTTTTACGTAAAAAAATCTTTTTTTGGAGACGTCTCAT
TATATTGCCCAGGTTGGTCTCGAACTCTTGAGCTCACCTCAGCCTCCCAGAGTGCTAGGATTACAGATGT
GAGCCACCTCAGCCAGCCCCTAGATTTTTTTTTTTTTTTTAATAAATGGAATCAAACAGCGTGTAACAGA
GGTGTTCAATCTTTTGGCTTCCCTGGGTCATATTGGAAGAAGAATTGTGTTGGGCCACACATAAAATACA
GTAACACTAATGATAGCTGATGAACAAAACAAAAAAAAATAGCAAAACTTATAATGTTTTAAGAAAGTTT
ATGAATTTGTGTTGGGCCACATTCAAAGCCGTCCCAGGACGCAAGTTGGACAAGCTTGGTATATAATTTC
ATATGTGTGTCCTAAACAGTGTAGTAATTTGAATTTCATGTTAGTATCAGCTTATTCCTTTTTGTTTGTT
TGTTTGTTTTTGAGATGGAGTCTTGTTCTGTGTCCCAGAATTGGTCTGCAATTCCACTGCCTCAGCCTCC
CAAGTAGCTGGGATTACAGGCACGTGCCACCACACCTGGCTAATTTTTGTCTCTCTCTCTTTTTTTTTTT
TTTTTTTTTTTTTTTAGCAGAGACGGGATTTCACCATGTTGGCCAGGCTGGTCTCAAACTCCTGACCCCA
AATGATCCACCTGCCTTGGCCTCCCAAAGTGCTGGGATTACAGGTGTGAGTCACCGTGCCCAGCCAGCTT
ATTCCTTTTTATTGCTGGGTAGCATTTCATTTTATGATTATACCACAGTTAATTTACCCATTACTAGTCG
ATGGGCATTTGAGTTATTGCCAGCTTTTGGCTATTATGAATGAAGCTGCTGTGAGCATTTGTGTACAAGT
GTTTGTGTTTTTATTTCTTTTAGTTAAATACCTAGAATTGGAATTGCTGAGGTATGGTAAGTGCATATTT
CATTTTTTTAAAAAATTTATTTTATTTTTTTATTTATTTATTTTTTTTGAGATGAAGTCTCACTCTGTTGC
CCAGGCTGGAGTTCAGTGGCGTGATTTCAGCTCATGGCAACCTCCCTGTCCCGGGTTCAAGCAATTCTCC
CGCCTCAGCCTCCCAAGTAGCTGGGATTACAGGCGCGCACCACCATGCCTGGCTAATTTTTTTGTATTTT
TAGTAGAGACGGGGTTTCACCACGTTGGCCAGGCTGGTCTCGAACTCCTGACCACAAGTGATCCACCCGC
CCCAGCCTCCCAAAGTGTTGGGATTACAGATGTGAGCCACCACACACTGCCTGGTAAATACATATTTCAA
TTAATAAGAAACTAGCAATCTTCTAAAGTGATTGTGTCATTTTACATTCCAACTGATCAGGTACATGTGT
AGGTTCCATGTGTTCTGCATCCTTGCCAACACTTGGTATTGTGTTATCTTTTTAATTTCAACAGGTCTAA
TGGGTGTCTTATGGTATCTCATTGTGATCTTAAATGTACATTTCTCTGATGATGACTGATCCAGGAGCAC
CTCATCATGTGTGTGTTTGTTTTCAGCTGTCAACCTTTTTTTAGTAAATGGTTCAAATCTTTTTTCCATT
TTATTTATTTATTTATTTATTTGATGGAATCTCACTCTATTGCCCAGGCTGGAACGCAGTGGTGCCATCT
TGGCTCACTGCAACCTCCGCCTCCCAGGTTCAAGCAATTCTTACGCCTTAGCCTCCCAAGTAGCTGGGAT
TACAGGCATGCGCCACCATGCCTGGCTAATTTTGTATTTTTAGTGTAGGTGGGGTTTCACCATGTTGGTC
ATGCTGGTCTCTAACTCCTGACCTCAGGTGATCTACCTGCCTCGGCCTCCCAAAGTGCTGAGATTACAGG
TGTGAGCCACTGCGCCTGTCCTAATAATTTCTTTTTGTCTCAATGTTTCTGCCTGGGTGCACTGGCTCAC
GCCTGTAATTCCAGCACTTTGGGAGGCCAACCTGGATGGATCATTTGAGCCAACAGTTTGAGACCAGCCT
GAGGAACATGACAAAACCCTGTCTTTGCAAAAAAAAAAAAAGAAAAAAGAAAAATTAGCCAGGCACAGAAG
CGCATTCCTATGGTCCCAGCTACTTGGGGGGCTGAGGTGGGACAATCGCTTGAGCGAGGTTGCGGGGGTT
TGGAGGGCGATGGAGGGGTGATCGAGGTTGCAGTGAGCTGAGATTGCACTACTGCACTCCAGCCTGGGCA
ATAGAGCCAGACCCTGTCTCACAAAAAAAAAGAAAAAAAAAGTCATGTTTCTTTTCTTACTGTGAAAATAAA

Alternatively spliced exon 18.5, encoding a structured sequence, with residues VKYLELELLRY

This G is part of the exon

GTTACTACTTTTAGTAAATTATTTTAAGTTATTTATATATTCTGGTTACAAGTCCTTTCTCAGAATATTG
TGAATATTTTCTCCCAGTCTGCGGTTTTTTTTGAAGAGCCAGTATTGTTAATTTTAATGAAGCCTTATTT
ATCAAGCTTTTCTCTTAAGGTTCATGCTTTTTTGTATCATAATAAGAAATCTTTTACGTACCCTAGGTTA
TGAATGTTTTTATGGTTAGGTATATGGTTGATTTCAGGTTAGGTTTTGTGTAGGGTGTGATGTAAAGGTC
TAGCTTCATTTTCTCCACCATAAATATTTACTCGGTTTCTCTGGCACCAGCCTCTGTTTTCCATTGGTGG
CTTTATTTTTTTTCTGTTCTTGAAACAAGAGTCTCGATCTTGTTACCCAGGCTGGAGTGCAGTAGTGTGA
CCTTGGCTCACTGCAACCTCCACTTCCCAGGGTCAAGCGATTCTGCCTCAGCCTCTCGAGTAGCTAGGAT
TACAGGTGCCCGCCACTACACCCAGCTAATTTGTATTTTTTTTTTTTTTTTTTTAGTAGAGACAGGGTC
TCACCATGTTGGCCAGGCTAGTCTCGAACTCCTGACCTCAGGTGATCTGCTCATCTCAGCCTCCCAAAGT
TCTGGGATTACAGGCATGAGCCACTGCGCCCAGCCATAGTAGCTTTATTGAATTCAGTTGACTGTATTGT
ATGTGTGTCTATTTGTGAACTGTTTTGTTGTATTGATCTTTGTATATATCCTTATGCCAATTCTCTCTTT
ATTGCTGTTACTTTGTAACCAACCTTTAAGTTCATATGAGTCTCCCAGTTTTATTCTCGTCAAAATTACT
CTTATTCTGCGTTCTTTGAATTTGCAAATAAATTTTAGAATCAGCTTGGGATTGTGCACTGAATCTTTAT
ATCAGTTCTGGGAGAAATATCTTAACAATATGGAATCTTCATTGAGGTCATCATATACTGCTCCATTTAT
TTAAGTCTTAAGTTTCACCAGTGTTTTCTAGTTTTCTTTGTATCAGTTTTGTGCCTGCTTTCTTAAATTT
ATCCCTTAATATTTCATCTGTTTTGTGCTGTTGTGAGTTATATTTTAAAAACTTTCAACGTTTGTTTATT
CGTAAATAGAGATGCACTTGATTTTTGAATATTGACCTTGTGTCTTGATGTGTTGGTAAACCCACTGTTT
CTGGCAGCCCTTTAAGACTTAAACATACAATCATGATCTAATCACCATGTTGGTGTTTTTGGGTTTTTTT
TTTTTGTCTTATTGTACTGGTGCATTACTGAAAAAGGCATGAGATTTTGCCATGCTCCCATTTTTAGGGG
TGAGACATTGTCTTTCACTATTAAGCATACAGTTAGGTGTTACTTCAGTTCCTAATTTGCAGAGGTGGGT
TTGTTTTCTTTTTAATCATGAATGGTTGTTGGATTATGTTCAAATACTTATCATCTACTAAGTATATCAT
ATTGACCAGGAACAGTGGCTCATACCTGTAACCTCAGAGCTTTGGGAGGCCAAGGCAGGAGGATCGCTTG
AGGCCAGGAGTTCAAGACCAACCTGGGTGATGTAGGAAAACCCCATATCTACAAAACAATTTAAAAATTT
GCTGGGTGTGGTGGCACACACCTGTAGTCCTAACTACTTGAGAGGCTGAGGAAGGAGAATTGCTTGAGCC
CAGTAGTTTAAAGCAGCAGTGAGCTGTGATTGTACCACTGTACTCCAGCCTGGGTGACAGAAGGAGACCC
TGTATTTAAAGTGTGTGTGTATGCGTGCGCATAGATGGATAGATAATAATGTAATTCCATTATGGTCATA
CAAACTGATATGAAATGCCATTTTATCATATAACAAGTGTCTTTTTGTGGTTGAATTTGTTTCTGGATTT
TTCACTCTGCTTCACTAATCTAATAGGACTACCTTCTCATCCACTCACTGCCAACATTGATTTTTTTTTT
CAGATTACCTTGAATTTTCTGTTTATTTTTCCATATGAACTCTATAATTAACTTACTACTAAAAAAATCA
GTTGCCTTTTTAAAACCAACTGATCTTTAAAATATATCTTGGCTGGGCCCGGTGGCAGGCACCTGTAATT
CTAGCTACTTGGGAGACTGAGGCAGAAGAATTGCTTGAACCCAGGAGGCGGAAGTTGTAGTTGAGTTGAG
ATTGCGCACCTGTACTCCAGCCTGGGTGACAGAGCAAGATTCCCTCTTAAAAAAAAAAAAAAAAAAAAGA
AACAGAAAAGATAAATCTTTTTACAATAATTTGTTCCAATTAGGGTCCAAGTCAGGCTTGCAATTTGGAT
TTGTTTATATGTTGAAGTCTTTTTTTTTTTTTTAATTGTTTCATATTGTGGTAACTTTTTTTTTTTTTTT
TGAGATGGAATCTTGGCTCTGTCACCTAGGCTGGAGTACAGTGGCACAATCTCAACTCACTGCAACCTCC
CCCTCTGGGGTTCAAGCAATTCTCCTGCCTCAGCCTCCCAAGTAGCCCAGCCTTTTTTTTTTGAGACAGA
GTCTCGCTCTGTTGCCCAGGCTGGAGTGCAGTGATGCGATCTCGGCTCACTGCAAGCTCCGCCTCTTGGG
TTCATGCCATTCTCCTGCCTCAGCCTCCTGAGTAGCTGGGACTACATTCGCCCGCCACCACACCCGGCTA
ATTTTTTTGTATTTTTAGTAGAGACAGGGTTTCACCGTGTTAGCCAGGATGGTATCGATCTCCTGACCTC

GTGATCCGCCCGCCTCGGCCTCCCAAAGTGCTGGGATTACAGGTGTGAGCCACTGCGCCCGGCCTTGTAT
TTTTAATAGAGATGGGGTTTCACCATGTTGGCCAGCCCGGTCTTGAACTCCTGACCTCAAATGATCCACC
CGCCTCGGCCTCCCAAAGTGCTGGGATTACAGGTGTGAGCCATCGCTCTCAGCCTTGCGGTAACTTTTTA
TTACGAATGTATTGAGACATTAATAACCTAGGCCAGTCATGTTTCATCCCTACCCATTGTCTCTTAAAAG
CTTTGAGTCCACTGGATTATTCTGAAGCAAATTCTAGACATTGCATCAGTTTATCCACCAACATTTTAGT
GTGTATCTTTAAGTTGGTTTTGGTTTTGTTTTTTGTTTTTGAGATGGGGTCTGGCTTTGTTGCCCAGGCT
TGGAGTGCAGTAGTGCAATCATAGCTCACTGCTGCTGCGAATTCCTGGTCTCAAAGGATCCTCCCTCCTC
AGCCTCTCAAGTAACTGTGACTACAGGCACATGCCACCTTGCCAGCTTTTCTTTTCTTGTCTTGTCTTTC
TTCTTCTTTGTTTTTTTGTTTGTTTTTTGTTTTTTTTTGAGACAGAGTCTCACCATCTTTCTATCTTGCC
CAGGCTAGTCCTAAATTCCAGGGCTTAAGTTATCTTTCTACCTCAGCCTCCTAAAGTGCTAGGATTACAG
GCCAGCACTTTAGGAGGTGCTGGATGAGCCATCACACCCAGCCAAGTCATAGGTTTTTTTGTTTGTTTGT
TTTTTGAGACAGTGTCTAACTCTGTCACCCAAGCTGGAGTGCAGTGGCATGATTTCAGCTCAGTGCAGTC
TCTACCAATTGGGCTTAGGTGGTCCTCCCACCTCAACCTCCCAAGTAGCTGGGACTAAAGGTGCGCGCCA
CCATACCTGGCTAATTTTTGTATTTTTTGTAGAGACAGGGTTTCGAATTCCTGAGCTCAAGCAGTCTGCC
TGCCTTGACTCCCAAGGTGCCAGGATTACAGGCATGAGCCACTGCACTCAGCCCTCACAGTTTTAATTAC
AGTTTTTCCCTTAGTTTTTGTCTTGTTCATATCCAGCTTGTCTTGTATTTTTTTCCCACGATCTGAATTT
TGCTGACTGTATCCCTGTGTTGATATTTAAAGTAGACTTCTGTCCCCTGTAATCTTTGTAAACTGATAGT
AAATAATGAAGGCTTGATCAGATTGGGTTTTTTTTTTTTTTCCCCAATGTTTCACAGATGTGTGTACTTT
CAGTGAGGAGTCATGTAATCAGTCTTTTTCCTGATAGGAGTAGTCAGTGAGTTCCTAGATGTTTTATCTA
TCCAGGAGATAATATGTCCCTTTAGCGCCTTAATTTTTTTGGTGTGTTTTTTAGCAGCCATTGATGATAA
TTGTCTAGCCCAAGATCAGTTATTTCCTTAGGGGTTGTAAAATGGTGACATTCTTTTCCTTTCATCCCTT
CTTCAATTATTGCCTGGAATATTTCTATAAAGAAAAACTTTCCCATATCCAGCTGTTTGGTTACCCTGAG
GTATAGCTTTCTTAGGAAAAGTAATTTAAAATGTTAATCATTTCCCTTTTTAAGGCAGTCTTCAAAATAA
TGAGTTGGTTTTCTGTTATCCTCCAAAGGTAACCAGTGAGGTGGTTTTTTTGTCGTTGGTTCTTACTATC
AGTATAAACTTCTGGAATTTTTTTTTTTTTTTTAATTTTTTGGAGACAAGGTCTGGCTCTGTTACCTAGG
CTGGAGTGCAGTGGGATGATCTGGGCATACTGCAGCCTCAACTTCCCGAGCTAAGGCAATCCCCCCACCT
CAGCCTCCCAAGTAGCTGGGACTACAGGCAAGCACCACCGTGCCTGGCTTAATTTTTGTATATTTTGCAG
AGACAGGGTTTCACCATGTTGCCCAGGCTGGTGTCGAACTCCTGAGCTCAAGCAGTCTGCCTGTGTCAGC
CTCACAAAGTGGTGGGACTACAGGCATGAGCCACCATGGCAGGCCAGAATCACAATAAACTTATAAATTA
ACTTGAGAAGAAATGATTGATGTCTTCATGATGTTGAGTCTTCCTGTTCAAGAACAAAGTATACCTTCAA
TAGCATATTAAAGTTTATCCTTGGCTGGATGCAGTGGCTGACGCCTGTAATCCCACCTCTTTGGGAGGCA
GAGGTGGGCAGATCACCTGAGGTCTGGAGTTCGAGACCAGCCTGGCCAACATGGTGAAACCCCGTCTCTA
CTAAAAATATTTTAAAAAAAGTATTAGCTGGGTGTGGTGTGCACCTGTAGTCCCAGCTACTCTGGAGGCT
GAGGTAGGAGAATCGCTTGAACCCAGGAGGCAGAGAGTGCAGTGAGTCAAGATTGCACCACTGCACTCCA
GCTTGGGCAACCGAGCGACACTCTGTCTCAAAGAAAATAAATAAATAAAAATAAAGTTTATCTTTAAGGT
TTTGTACATTTTTTTCAGTGTATGCCTTAGGTAGGTTCTTTTTTAATGTTAGTGTAACCCAGGGACTTCT
CTTCCATTGCATCTTCTAAGTAATTACTTATGAAGTACCATATATGAAGGCTATTGCTGTTTATATGTTA
GTTTTTACCCTGCTCCTTTACTAAATTCCAATCCTTTGAGGTATTGGATAAAAATATTTTTAGCATTTTT
CAAATAACAGGCAGAGTCAAGGGCTTGGTTTCTTTTCTTCCCCTCCTGTCCCCTACCCTCCCCTTTTTG

AGACAGGGTCTCACTTCTTCGCCGAGGCTGGAGTGCAGTGGTGCAGTTACGGCTTACCGCGGCATCTGCC
TCCCTGGCTGAAAAGTTCCTCCCACCTCAGCCTCCTGAGTAGCTGGGACCATAGATGCACAGCACCGCAG
CTGGCTAATATTTTTGTATTTTTTGTGGAGGCAGTGTCTCCCCATGTTGCCCAGGGTGGTCCCAAACTCA
TGAGCTCAAGCAGTCCGCTCGCCCTGGCCTCCTAAAGTGTAGGGATTATAAGCGTGAGCCACTGCGCCTG
GCCTGGGGATCATGTTTTAACATGAGAATTAGTGGAGACAAACACATGATATCTAAATAATAGCACCATA
GTATACTTGACTAGCTTTTTAATTATTTTTTAAATATACAGGAAGGTAATAAGTAACAAAGTAATAATAG
TGAATAGTTTAAGCTCAGTTAGCATAATCGGGCAAACTTTCATTTGATAAAAGTGATAAGTAGTTTTCAG
TGGCTTTTTTGTTTACCAGAAGGAGGTGGTTTTTAAATACGTGCATCCAAGATAAAATATAAAAAAATGT
TCAGGTTTGCTTTCCTACATAGATAAAATAATATGTAACTAGCTCTCCCAAATTTCAGCAACAGTTAGTG
AATGTTTAGCCACAAATTTGCAGTTAATTATATAATCAGTTCTTAGGATTTTATGAACAAGTTCTATATT
CTTTGTGCCTTATACCTAGTTGTAAGCAGTCATTCCACAATTATTTTCCTGAAGTGGCTTGGTTAATGCC
ACACCAGAAACAGGTCACAGACAATAGTGCTGTAAGAAATGTGTGAGGAAAGAGGCACATGGGAAGTAGC
TAGCTCGTGCTGGAGGAACTGGAAAAAAACCTCACATGGGAGATGACAGTTGAGCTGAATTCTTAACTAG
AGTTGTAACAGGGCGAGGCCCTTACATGCAGACCACCTGTGTGGATTAAGATAAGACATAAAGTAATCTT
TTAAAAGAACTATTATTTAGAAACCTGGTATATGCTACATGGTGCTGTGTTATACTGGGTTTGAGAAAGA
ATGGGAAGTGTTACAAGGATTCAGTGGTTGGAAATTAAGGAAGATAGAAAGTTAGTGTTGGATCTGTTTT
GGCTCTTTGGTCATGCCTTTGTTTTTCTCAAAATGAATGCAGTGCCCGTCCCAGAAAATACCATATGAGA
AGCGATTTCATAATGCTGTGAGAGTCTGTTACAGGGACTTGATCAAGTCTGAGGGCCATGAGAGAAAGTC
CCTCTGAGGAAGTTGCTTTCAAGCTGACACCTGAAGGATGAAGCAGAATTATCCCAGCTGGGATTTGGGA
ACTGGTGTTTGAGGCTGAGGACTAGCATGCATGATAGGAAAATAACCCAGAGTGGCAGAAGTGGGAGTGG
TATGAGATGGCATCAGAGACGCAGATTCAGGGTCAAATCATTCAGAGCCTCCTAGACCATGTGAACACAT
GTATTATGCTGTGGAGATACTGTTTAATAGGCAGTCTGCTTTTTTTTCTGCAGTACCAAATATGCCCCAA
CAGCGGCAAGACCAGCATCATCAGAGTGCCATGATGCACCCAGCGTCAGCAGCGGGCCCACCGATTGCAG
CCACCCCACCAGCTTACTCCACGCAATATGTTGCCTACAGTCCTCAGCAGTTCCCAAATCAGCCCCTTGT
TCAGCATGTGCCACATTATCAGTCTCAGGTAAGGCTGGTAAGGCCTAACTCTTAATTTTTGTACCATATA
AAAAAACTTTTAATATGGTAAAGGGATTTTCCTTTATAATTTTTGCTTTTGTGTGATGGTAGGGTAGATA
GCTAAGGACTTGGGGACCCTTTTCAATATATATTCGAAGGTTACTGATGATTGTAAGAGGTTCAGAGGAA
ACAGCCAAGAAAGATTTGAGAGTTTACAGCTGTTTCTGGAAATCTGGAAACCATGGAGTTAAAAATCTTA
ACTAAAGTCTGCTTGGCTCTATTTGCAGTGTTAATGTGCTTTCTTTATTTTTTGTTTGAACACAGCATCC
TCATGTCTATAGTCCTGTAATACAGGGTAATGCTAGAATGATGGCACCACCAACACACGCCCAGCCTGGT
TTAGTATCTTCTTCAGCAACTCAGTACGGGGCTCATGAGCAGACGCATGCGATGTATGGTAGGAAGCACT
TTGTTTGTCTCTTCCAGTGTGTGTGACTCTTCTTAATTTAAGTTTCTGAAAACATACTCTATCTAAGAAT
AACCTGACCTTTTATGACATTGAGGGTCAAGAATCTGAAGGAAAAGATGAACCCATTTCTTTGCCTGACT
TGCTTTATAACTTTTGGCAAATAGTTTCTACTTCTGTACCTGGTCTTCAGATCTCTTTCCTGCTTTAACT
AAAATGTAATGATGTATATAATGGCAAAGCATCTTTGTGGAGAAAGGTACCTTTCTCCTCTTCCTCATCA
ATATTATGCTTTGGTATATCCTGCCTACGACATGCAAGAGAATTTTATAATAATAAAAGCATAAAGGTGT
TCTCCAGCATGAAAACATTTTGCTTCACTACTTGATCTGAGGGTCACTGGCATTACATATTTTTTTTGCT
GTTTGTTATAATGATAATACTATGTTTCTACATCATGCTGTATTTTAATGGTTGAATATTATGTCATATT
AGATATATTTTAGACATGAGTCACACTTTAAATATAACCAATGTGAACAGAATGCTGAAATGAAAATGAG

The first G is in the intron: exon starts with "GTAC"

Usually constitutive exon 19, encoding structured sequence, with residues VPNMPQQRQDQHHQSAMMHPASAAGPPIAA TPPAYSTQYVAYSPQQFPNQPLVQHVPHYQSQ

Usually constitutive exon 20, encoding structured sequence, with residues HPHVYSPVIQGNARMMAPPTHAQPGLVSSS AT QYGAHEQTHAMY

AAGTATTTTATGTAAAACTAAGCAGTATTTATATGTGAGAATAATAAGCAAAAAAACCCATCTTCGTTTT
GTGACTAAACAGAGAAATTTGTGTAGATCAACTTAGCAGCTGTCTAAAGTACCAAAATAATAGATTTTTC
ACTGTTGATAATTTAAAATAAAATGTCCATTTGTATATCTTATGATACAGAATTAATGGATTGCTTCAAA
TGTTTTTCAGAATATGTTTTTAAATAGTACTGATTTCATTAAGATGTTTTGTTCTGAATATTTCTGAGAA
CTACCGTAGTGTCGTTTAGTTTTCCTATTTGCGTTTTTGGTTGTTTGGAGTAGGGGATAATTTTGGTTTA
TTCATACAGTTGAAAAGTGTACTGCTATGAGAATGAGATTATGGTTACATGTAACTACATGGGCATTTCA
TTTTTAAAGCCTCTTTGAACTTTTTGAAATACTAAGAATATAAAATTTTTATTTTTTAAGTTTAGATGTC
CTGAACGAGTATGTTTAGGCAAAATTGAGTTATTTAAGAATTTATAGGCTGGGCGCAGTGGCTCACGCCT
GTAATCCCAGCACTTTGGGAGGCCAAGGCTGGCGGATCATGAGGTCAGGAGATCGAGACCAGCCTGGCCA
ACATGGTGAAACCCCATCTCTACTAAAAATCCAAAAAATTGGCCGGGTGTGGTGGCATGTGCCTGTAGTC
CCGGCTACTTCGGAGGCTGAGGCAACAGAATTGCTTGAACCCGGGAGGCAGAGGTTGCAGTGAGCCGAGA
TCGCGCCACTACACTCTAGCCTGAGCGACAGAGTGAGACTCCATCTCCAAAAAAAAAAAAAAAAAAAAAG
AATTTACAGATTTCTGGCAAACCTTCTTCTTGAGACATTACTACTTTTCATACCACCTCTGTCCTTTTTG
AAGAATAAAAGTTTTAACATTCCGTAGGTTAATGAGAATAGGACTTGGGCAGCAGCAATCATCCTTCCTG
TCACCTGTAACCCACAGCTTATGCTTTCTTCCTGGAGGTTCTTGTCTGCCACAAAGGCTCACTGCTGATA
GGAATTTGTATATGATCAAAGGTGTTTAGTTTTATAAAACAGTTAAGTCCAGTCTTAATTTTCCACATTA
TCACTTTCAATTTTGTATTGTGGATTACGCATTTTAAATAAAAAATTGTGTGATTGCTACATTTTGGAAA
ACATTTTTTTCAAGAGGCCCATCCGTAATTTAATTGTAAAAGATACTGACAAACTAACTTGGTTTATTAT
TTTGGTTATGACCCCGTCATTTGACTTGTCTTTAGTTGTCTTAACGGGGACTGAATATGCGTGCAAAGGC
ACGATTGATTTATCATGCTGGCTTTTATGCAACTTGTATATATTTTAACAATTTTCCTGTTTGCTAAAGG
CTTAGGTTAAAAGTTCATTATGATTGTTTATACATTTCTGGTGAATACATCATGATTTAACAAGTGGAAA
GAACATCTCTTTCCTTCCATTTTCTGGCATACTCCCCTTGGAATCAGATCTGAAACTTTTAAGCTAAAAT
TTCCATTGCATTTGGAGAGTAGTTATTTGTGTATGCATGCTTTTGAGACATTGTAGCAATAATACTGTAA
TGTTGAGCCGAATCTTTCTCCTCATTGTGTTCATTCACTGCCAACATCTGGCTTCATCTTTTGGATGAAT
GTTCATTGGTTTTGAAACAGCCTATAGGGTAAATACTGTGTTTGAGGTACAGATGATTTTCATAACTACT
TCCTAGAACATGTCCATTTGAAGAGCAGTGGGGCCTTAGACCCCAAAGTCCATTTATGTGTGGGCAAATA
GGAAATGTTGCAAACAAAACAAAGCACTAGATCTAATGTCCAGTGAAATCTGGAATGAACTAGTCATTAG
AGCCGGTTCTTTCATGCCAGGAAAAAGTTACTCAGCCAAATCTGAACTACTCTCCTGCAGTTTACACAGG
TGGTATTTAATTGCTGTCTGTATGGAGGCAGGCTAGGAGCAAGGCTGTGGACTTGTTGTGATTGTCACTA
GTTAATCAAGATTCCCTTTGTGGTGCTTAAGACCCTAAAAAGGACACTAGGAGCTGGGCATGGTGGCTGA
CACCTGTAATCCAAGAACTTGGGGAGGCTGAAGTGGAGGATCGCTTAGCCCAGGTGTTCAAGACCAGTCT
AGGCAAGATGGCGAGATCCCATCTCTACCAAAAAAAAAAAAAAAAAAAAAAAAAAAAAAGCCCAGTCATGG
TGGCACATGCCTGTAGTCCCACCTACACAGGAAGCTGAGATGGGAGGATCACTTGAGTCCAGGACTTTGA
GGCTACAGTGAGCTATCATGGCACCACTGTAATCCAGCCTGGGTGACAGAGCAAGACCCTGTCTCTATTT
AAAAAAAAGAAAACATAAGAAAGAATTGTTTTGTTCTATGCCATCATAAGCCATAATTTAATCTGCTTAA
GCATGTTCTTCATTAAATCTGCAGTGATTTATTTGAATTATTAGACTTTCAAAGCCTTATTATATCAAAT
ATAAACAAAATTTGAAGTACATTCTTATAAACTACAACAAACTTACATAGAAGTGTTAATTTTATACTCA
TCTTCCCTGAACAATTTATATTTTATAAATATATTAAATATATTGTCATAAATTTTCTCAAAGGAACCAA
ATACTTTGAGTATGAATTGTGCTTTTCTTTTTAAGCTACATCATATCTAGGTTTTTAAAACATTTAATGC

AAACAGAAGAACATGCACCCAGATGTTGGTGACAATTTTATGTCACCTTTTCTCATTCATTAATTGTTAT
AGCCATAGCCAAAGGCATTGAAAACATAGGACCACTAATGACTGCAAAATGAAATCCTGATTATTGTTTT
TAAATTTTTAGTATGTTTAATACACATATGCTAACATTACTGAACAGTTAAATGATAAAATAGGATAATT
ATTTTATTCTAAAAAAGTATTGACCTTGACCTCTTTCTAGCTATCTTAGAAAGGGCTTTTGTCAAAAACC
TTATCTCTTTGATGTCTCTTTTTTTGAGATGGAGTCTCTCCCTGTCGCCCAGGCTGGAGTGCAGTGGCGT
GATCTCAGCTCACTGCACGCTCCGCCTCCTGCGTTCACGCCATTCTCCTACCTCAGCCTCCCGAGTAGCT
AGGACTACAGGCGCCCGCCACCATGCCCGGCTAATTTTTTGTATTTTGTTTAGTAGAGATGGGGTTTCAC
TGTGTTAGCCAGGATGGTCTTGATCTCCTGACCTCGTGATCCGCCTGCCTCAGCCTCCCAAAGTGCTGGG
ATTACAGGCGTGAGCCACTGTGCCCAGCCTCTTTTTTTTTTTTTATTTTTTATTTATTTTTTATTTTTTT
TTTAATTTTTGAGAAGGAGTCTCCCTCTGCCACCCAGGCTGGAGTGCAGTGGCGCGATCTCAGCTCCCTG
CAAACTCCGCCTCCTGGGTTCAAGCAGTTCTCCTGCCTCAGCCTCCTGAGTAGCTGGGACTACAGGTGCC
CGCCACCACACCTGGCTAATTTTTGTGTTTTTAGTAGAGACAGGGTTTCACCATGTTGGTCAGGCTGGTC
TTGAATTCCCGACCTCAGGTGATCCACCCACCTCAGCCTCCCAAAGTGCTGGGATTACAGGCGTGAGCCA
CTGCCCCGGCCTCTTTGATGTCTCTTAATCTAACTTCCATCATTGCCTCTACCCCATCCCTTCTAAGAAG
TTACTTTAATTTTTTTTCCTCTCACATCTACTCTTTTTTTTTTTTTTTTTTTTTTTTTGAGGTAGTCTCA
CTCTGTCACCCATTCTGAAGTGCAGCGGTGCGATCTCAGCTCACTGCAACATCTGCCTCCCAGGTTCAAG
CGGTTTTTCTGCCTCAGCCTCCCGAGTAGGTGGGACTACAGGTGTGCGCCACCACGACCGGCCAATTTTT
GTATTTTTAGTAGAGACGGGGTTTCACCGTCTTGGCCAGGCTGATCTCGAACTTCTGACCTTGTGATTTG
TCTGCCTAGGCCTCCCAAAGTGCTGGGATTACAGATGTGAGCCACCACGCCCAGCCTCACATCTACTCTT
CTAATCCATCTAATTTTGTTTTATGGTGATGCTTTTACCTTTCAGAAACAGTAATAATACAACTTTTCCG
ACTAACTAGAGCCATTAGGAAGAATTAGATCCAGAATCCTTTTTTGATTTGTTTTTGGTAGTTTAATGCA
GATAAGTAAGAAAATATAGTTAAGTTAAAAAAAAAAAAAATGAAAAGCATCCATAATCCCTCCACCTGAC
AACTGCCTTTTAACATTTTGATGTGTATCCTTCCAGGTGTATTTAAATACACTCAAATACCCTACCCCTT
TATGTAGACATGTTTTAATAAGAAATAATATTCATGTTTATATTCTTGCTATGATCCTAAATTTTTGGAT
CCATTACTAGATAATCTTTCAGGATAATGACATTTCCATTAGTAATGTTTTTGCAAAATTGTGTGTCTAT
TGAATTAAACTTGTAAAATAGTTTTATTTTGGTACATGATTTATATCAAGGTTGTTCAGTAGAATGCCAT
GTTGGTGTTTTTATTAGATAATGATTTTATTCCTTTTACTTTTAAGCAAGTCAGCATGACAACTTGACAC
CTAAGTACAGAAGAACAGTGTCTTCCGGTTTAGTCCTTTCTTTTAAAATTCTGTAGCAGTGTTTAAAGTG
CTTGTCATCTCTTATGAAAATGAATTATGCATGAATACAAAAAGAAATTACTAATATGTCAACCTTTCCA
GAAAATTTGGAAAATGCACACCTCAAAAGGCTAATTTACCTTTCTATTTCCCAAATTCAGCATGTCCCAA
ATTACCATACAACAAGGAGACAAGCCCTTCTTTCTACTTTGCCAGTGAGTTGGGTTTTTTATACTAATTT
TTAATTGTACAGTAAAACACTTTTTAAAGGATACATGTTAAGGGAGTAGACTTGTTGAACAATATTTTCC
TTGTGCCAGTCAAATTATTGAAAGTACTTATATATATAAATAATTCAGTTTTTAAAATGGAAATACCCAA
TTTAAGAAGGCTGGAGTTAATGAAAAATGGAGTTGTTTCAGAAATCAATTTTTGCATACCAAGCAAATGT
GACTGGGAAATGCCTAATATTTTCCTTGTTAGAGAAACTTCCTAAACAGCTTTATACACACACACACACA
CACACACACACACACACACAAACACACACACCCAAGCCACAAGCTTGGTATAAATTTAAAATGTTTATTT
ATACACACACACACACACACACACACACACACACACACACCCCAAGCCACAAGCTTGGTATAAACTTAAAAT
GTTTATTTATATTCTGATAAGATGAAATTTATGCCTACCAGGATTTTTAATTGAATAGGATTGATGAAAT
ACTAAGGGAAAAACTTTTCAGTCCTGTGCATGGCTAAAGGTTTAAAATACTCAGGAAGGGCCAGGCACGG

Alternatively spliced exon 21, encoding structured sequence, with residues ACPKLPYNKETSPSFYFA

TGGCTCACACCTGTAATCCCAGTGCTTTGGGAGGCTGAGGCGGGTGGATCATCTGAGGTCAGCAGTTCAA
GACCAGCCTAGCCAACATGGTAAAACTCCATCTCTACTAAAAAATACAAAAATCAGCCATGCATGCTGGC
ATGCGCCTATAATCTCAGCTACTAGGGAGGCTGAGACAGGAGAATTGCTTGAACTTGGGAGGCAGAGGTT
GCAGTGAGCCGAAGTCGTGCCACTCCACTCCAGCCTGGGTGGCAGAGCGAAATTCTGTCTCAAAAAATAA
AATATTCAGGAAGCAGACCCCTCAGGATATCTTGAGCTTAAGCAAGAGATCATGACCTCTCAGGTCATTA
TCTTGGACAGCACAGGTCCCCTCTCCCCACCTGGCAAAAAGTACAGAAATAGTTGCTCCTTCATGGAGAA
AGTCTGGGCAGAGCTTTCTTCTGGAAATGAACTTTTAAGGTACATTTTTCCTATTTGTAGGGCAATTTGT
AAAAATAAGGGCCGGACGTGGTGGCTCACGCCTGTAATCCCAGTACTTTGGGAGGCCGAGGTGGGTGGAT
TGCTTGAGGCCAGGAGTTCGAGAACAGCCTGGCCAACATGGTGAAACCCTATCTCTACCAAAGCATGGTG
GCACGCACCTGTAGTCCCAGCTACTTGGGAGGCGGAGGCACAAGAGTTCCATGAACCCTGGAGGTGGAGG
TTGCAGTGAGCTGAGATTGTACCACTGCACTCAGGCCTGGGCAACAGAGAGAGACTCTGTCTCAAAATAA
AAAATAAAAATAAGGCTAGTCTTGGACTTTGGTATTTAAATAGGAAGGAGTACTAATATTTGTAGAAATC
CTTTAGAAATTTGTGCCATTAATATTGTCACCTTGTATGAAATGTTGTGTTCTAGAGGATATTAAGGATT
CAAATTTTATGTTAGGCACATTTTGAGTTATTTTGGGGTGACTCAATGTCTGACTCTACTAAATGCCATA
TTAGCATTTAAAATGCATTTGACCTTAAATCTTTGTTAATTATGCCATGACTTGGTATCCAAAAATAAGC
TGATACATACATACATACATATATGTGTGTGTGTGTGTGTGTGTGTGTATATATATATATATGTATGTGT
GTATATATAATTTATTTGGTGCTAGGAAATGTTAAATTTAATCCTTTAATAGATGCTCTTTAAAAAGGAG
TCTTGCTGTATGTATATACTATTAAAGGGGAAACTATGTCTGTGATTGTAGTGTGTAAAAGATAGTAGGT
GATTTTATTATGTACTCAATTTGAGGTCTCAAATGTAGTTATCCTCACCATCTTACTGTCTCTGTTAGTA
GTTTGGTGTTGTTTTCCTGGTAAGTAGCTAAGGTCCTTAATCATTAACACCTAAGCCTTAATTGCCTTAG
CACAACTTCCCCTAAAAGGGAGTATCAGTACTTTTTAAAAGAAACTAACAGTTGGGCTGCTAATTTAATC
TGCTGCTTCATTTCCCCCTGTTCTAAGCCATTTTATGATGGTTTGGTCAAGTTGCCTTTTATTCCCCTTT
TAGAGTTTTCAACTTTCCTTCACTTCCCTTTTTCTGAATTTAACATCAGATTTACAAGTTGGAAGATTTT
GTTTTGTTTTATAAGTTTTGCAATGCTGGTGATCTCTTATGACTTGTGCATCCAAAGTCAAAATGACAAA
ACCTAGTTACAAATTAAACACACAGCTTTCTGTACTTAATTTGCTTCAGTGAGATCACAGCTGAGGAAAC
TAGTTCTGGAATGTGGTTAGTGTTATTAAGGATTTTTGACTGATCATATGTTTAGAATCTTAAATATTTA
TGTCAAGGAACACTGAGTGGGAAACTTCTGGACTAGGTCTGGACCAAAGAAGCATATGTCTTTGATTATC
TTTAATCTAAAAGATTTTATGAAGACTAAAGTTTTATAAATAGAAGTTTAACTGATGAATAAATCAGTAT
TACAAATAAAATTAACTTTATTTTTAACCTCTCTGGGATCTTTAGCCAGAATGAGCATATATAACAAAAG
CAGTGAAATAATATGTGTGGGTCAGAACCCACTGCCCTTCCCACTCCACTCTCCTTTTCCCTGATTCTCC
TGTGTTTTTTCCTTCTTTACCTTATCTTGGTTCCTTTTTTTTTTTTTTTCTTTTGAGATGGAGTCTCACT
CTGTCGTCCAGGCTGGAGTGCAGTGGTGCGATCTCGGCTCACTGCAACCTCCGCCTCCTAGGTTCAAGCA
ATTCTCTGCCTCAGCTTCCAGAGTAGCTGGGATTACAGGCGCCTGCTGCCACACCCAGCTAATTTTTTTT
GTATTTTTAGTAGAGACAGGGTTTCACCATCTTGGCCAGGCTGGTCTTGAACTCCTGACCTCGTGATCAC
CTACCTCGGCCCCTGGTTCCTTTTTTGTCTCTCTTGTCTTCCAAGCTATTTTTTTCCTTGGCTTTTAAAT
TTTCTTCCTACCCTGCTTTGTGTCACTGTCACTTAACTGGCCTATCAAGGAACCGAACTGTATTTTTGTT
ACTAGTATTGATTTAAAGTATAAGTTTCACATTTCTCCCAATTTATTATTATTATTTATTTATTTATTTG
TTTATTTTATTTTTTGAGACGGAGTTTCGCTCTTGTTGCCCAAGCTGGAGTGCAATGGTGTGATGTCGGT
TCACTGCAACCTCCACCTCCCGGGTTCAAGCTATTCTCCTTCCCCACTCTCCCTAGTAGCTGTGATTACA

GGTGCCTGCCACCACGCCCAGCTAATTTTTGTATTTTTAGTAGAGACAGGGTTTCGCCGTGTTGGCCAAG
CTGGTCTCGAACTCCTAACCTCAGGTGATCCGCCCGCCTCGGCCTCCCAAAATGCTGGGATTACAAGCGT
GAGCCACCGTGCCCGGCTCCATTTCTCCCAATTTCAAATTCAAGGAGGAAAAGAATTCCTGATTAAGGTA
CTTCTTTCAGATCTTTTGAGCTAGAACAAAAAAACAAAGGGAAATATTTCTAATTAACTCTTTTTAAATT
TTGTTTACAACGTATGATACATATTTTACACATCCTTTGTGGTTTTTGTTCGTCTTGTTTTTAATCAATG
CCTTGCAAGTTTACCGGTATTTAGGTAGGGAAAGGATTTTGTTTTTGTTTTTTTAAACAAAGCCTATGTA
CATTCACTCAGCTTGGGTATTTGTGCTATGCATGCAAATTAGCTATAGATTAGAAAACCGTATTATAGTC
TTTAAATACTGGTAAACTTAAATTGCAGAGATGCCTTTTAAAAATGCATAGTAAAAATATTTCATCTTTA
CTTTTCTCTTCAAATGATTTTAAGATTTTTACATTTTTCCAGTTGATGAATAACTTAAATTATGAGATTT
CATGGGCATAATTATTTTCTATATTTATTGTTACTTTTTAATATTCTTAATACTTTGCTTAGAAGGTATT
TAAAAGTGAAATTTCAAACTTTTTAGTACAAAATTTCTTGAATAAATAAAGTTACAAAAAAAAAACAAAA
ACCTCTGAGATTCCGTACTGTATCTTTATGAACCTCCATGAACAGAATTTGGGATTTGGGAATTGCTTTT
CCTTAGACAGATTTAGATTGTTACAAATGACATTTTTAAGAGGCTGGGGTGGCGGTAGGGGTTAGTGCTA
ATGGTTTAACAGTAGGGGACCATGGACAACTGTAGACATCACTATCCAGTAGAACATTTTGTGGCTGGGC
GCGGTGGCTCACGCCTGTAGTCCCAGCACTTTGGGAGGCCAAGACAAGTGGATCACCTGAGGTCAGGAGT
TCAAGACCAGCCAGACCAACATGGTGAAACCCTGTCTCTACTAAAAATACAAAAAAGTTAGCCAGGCGCG
CCTGTAGTCCTAGCTACTCAGGAGGCTGACACAGGAGAATCGCTTGAACCCGGGAGGCAGAGGTTGCGGT
GAGCTGATATCACGCCACTGCACTCCACCCTGGGCAACAGAGCGAGACTCCGTCTCAAAACAACAACAAA
ACTGCACTGTCCACCGTATTAGCTACTTAGCTACATGTGGCTTTTTTATTATTCAAAAATAAATTTTTAG
GCCGGGTGCAGTTGCTCACACCTGTAATCCCAACACTTTGGGAGGCCGAGATGGACGGATCACTTGAGGC
CAGGAGTTTGAGACCAGCCTGGCCAACATGGTGAAACCCCGTCTCTACTAAAAATACAAAAATTAGCCAG
GTAATCCCAGCTACTCAGAGGCTGAAGCAGGAGTATCACTTTAACCCAGGAGGCGGAGGCTGCAGTGAGC
CGAGATCGCTCCACTGCACTCCAGCCTGGGTGACAGCAAGACTGGGTCTCAAAAATAAACAAACATGGCC
GGGCGCAGTGGCTCATGCCTGTAATCCCAGCACTTTGGGAGGCCGAGGCGGATGGATCACTTGAGGCCAG
TAGTTCGAGACCAGCCTGGCCAACATGGTGAAACCCGTCTCTACTAAAAATACAAAAATCAGCCAGGCAT
GGTGATGCTTGCCTATAGTTCCAGCTACTCGGCAGGCTGAGGCAGGAGAATCGCTTGAACCCGGGAGGCG
GAGGTTGCAGTGAGCCGAGATGGTGCCCCTGCACTCCAGCCTGGGCAACAGAGCGAGACTCTGTCAAAAA
TTAAACAAATAAATACATTTTTAAAATGAACGTAAGATTTTTACAAGTACAACAAACTCAGGTTCGAAAT
TTACATCAAATCTTTTAGACCAAGTCAGTGCCTATACAACTTGGAGGAGCTGGAAGTAAACTTAATGAGT
ATGATGATGATGGAGGGCCTGTTAATAAGCCACCAAGTTAGAAAAAAAGGACTGTCTTATAGACTTATGG
GACTGTGAAGCTCAGGAAGGCTTCATCGTTTGTACATCATTTGTTCTAGCTCCCAGAAGACGTTCACTAC
TCTTAAAAACATTCAGAGACTATGTTGCCACAGTTTTCTTGTTAAAATATTCTGGCATATGTTAATTCCT
ACAGTCTGGAAAATTTTCCCAGTGTATAAACAAAGCTGCTGTATCCAGTCTAAACTGGATATGAAGGAAT
ATTAATGCCAGCTGTGGCATTGGCAGTGGATGCACAGGTGATCCTAGAACTGGCTCTTTGCCTTGCCCTT
TCCCCTGCTAAGAGATAGCTTTGCAGCTGGAGACGTAACTGTTAGGGCTGGAGAGTTGGTGGCCCTTAGC
CCTACAACACCTAGGATTATAGAACTGCTCCATGTGCCTAGCCTAACCCTCTGCACACCATTTACGTGGA
ATATACCCAGAGCCGTCTATGCTGGTGACTCGGCAGCCTTGCCTACCAGACTGCTGGAACTAGGGTGCCT
CTTCCCAAAGCTGTGCTTGCTTCTCTCACCAATCAGTCCTGCATATGTCTGTGTTTGCTAACACGTTATA
TGAAGAATGTGGGGAACTATTTTGGAATCATTTCTGTGTATGGGCTTATTATCTTGAGGGATTTTAGGAT

TTGTTTCTCAAGAGAGGGCTGGGAACTATACCTTGCTAGAGTTGTCTTGAGAACGCTCTATTCTCAGCTC
ATTGCCTCGTGGAGGTTAGTTTTTATCATCGGTGTGCTGTCCATAGTCACTGGAAGCAGTGAACACATC
CTACTCTGCTTCTGATTCTCAACTTACTGTTTTTGAAGCACATGAACAGGCCAGGCACGGTGGCTCACGT
CTGTAATCCCAGCACTTTGGGAGGCTGAAGTGGGCGGATCATTTGAGGTCAGGAGTTTGAGATCAGCCTG
GCCAGCATGGCGAAACCCCATCTCTACTAAAAATACAAAAATTAGCTGGGCGTGGTGGCACATGCCTGTA
ATCTCAGCTACTCGGGAGGCTGAGGCAGGAGAATTGCTTGAACCTGGGAGGCAGAGGTTGCAGTGAGCCT
GGGCAACAGAGTGAGTGAGACTTATATCTCAAAAAAAAACAAAAAACAAAAAACTGAAAGACATGAAGAA
ATGGTTTTTGTACCAAGGTTTGGCCCACGCTGAGATTCACAAAGAACTGGCTTTCAGTTCTTATCTTTAT
TTTGATTTAAACTGGCCCATCATGTTGTCCTTTGAAGTTAGTCTAGTAAATTTCTTTCCAAAGGGCTGGG
GCACTCAGAAGGGAGTTTACTTTTCTATATTTATTTCATAAAGCAAAGATGGGAGATCCTCCATTAGGGC
TTGGGAAAGTAAACTGAGTGGCAGAAGGGCTCCTGTGATTAGCTGAGAGAGACTGTGGTCCTTCGGCCCT
GATGATAGATCCCTGGCCTTGCCACATACCATACACAGTGCCCGCACCCCCATCCCCCACCACACCCAAT
ATAGTCTGTGCCCTCAGGACATTGCTCCAGGGCAGTAGCATGGTGAGGTTAGCCTGATGATGGCCTTGAG
CTAAAGAGTGTGCACCTAAAATGCACTTGTTTGAGTAGTTTCTGCCTATGCCTTCAAGTTGCCTTTTTGG
GAAAACCTAGTGACCGTTAAGAGTAAATGCAAACTAATTTGATTTTAATATCATATGTAGAGCTGTATTA
TATGAACCAAATGCTAGTCTGTTAAGCAATAGCTACACTTATTTTTTCAAGACAATGGATGGTTTAAATG
GAGTCATCTATAGAAATTGGTAGTGGCGTGAGTTATGCATTGTAACCATCAAGAAAGTTCAGTTGATGAA
GTGTAGAGGAGCGATGGAGGTTGTCAGACATCGGTTGTGTACATGCTCCTTTTTCTTTCACTTTAGTTTC
CACGGGCTCCCTTGCTCAGCAGTATGCGCACCCTAACGCTACCCTGCACCCACATACTCCACACCCTCAG
CCTTCAGCTACCCCCACTGGACAGCAGCAAAGCCAACATGGTGGAAGTCATCCTGCACCCAGTCCTGTTC
AGGTAAGGGCAACTCAGAGGTCTGCATGGAGTGGCTTCTTTATCCTAGTATCTGAGTGCTTTCTTCAGGT
GCCAGGTATCGCATCGTCAGAACACATGGCATGTCCACCCTCGTGAAGATGGATACAGCTGTGCCCCTGG
GGTGGTGGTTTTAAGAATCACATTTAAAGGCTGGGCGCAGTGGCTCACGCCTGTAATCCCACCACTTTGG
GAGGCCGAGGCGGGTGGATCACGAGGTCAGGAGATTGAGACCATCCTGGCGAACACTGTGAAACTCCGTC
TCTAATAAAAATACAAAAAAATTAGCCGGGCGTGGTGGTGGGCGCCTGTAGTCCCAGCTTCTCGGGAGGC
TGAGGAAGGAGAATGGCGTGAACCCGGGAGGCGGAGCTTGCAGTGAGCAGAGATCGCGCCACTGCACTCC
AGCTTGGACAACAGCGAGACTCTGTCTCAAAAAAATAAAAAATTAAAAAAAATCACATTTAAGATACATG
TTGATAATAAGGTGATTGGATAAGCTCTGGAAACTTGCAGTAATGAAAAATCAAATTTAACATAAAGTTC
ATAAGGCAAATTCCTATTTGCTTGGGACTTTTTAATTTCTAAGGTTTATGTGATGAGGTTATTTTCCTAT
GAGCTTCTTGAATTATGTTTGCTAATGGAGGCAGTTAAAGATGTCTTTGATATCTATCAGTTCCCTGGGG
CAGTAGTCTTTTTTGACTTTAGTATGTATGCTCAGAAGTTTCTAACTGCCAGACTGAGAATCAGGCTTCT
GTACCCTAGAAAGGAGTTGTCCAGATGGGAGGCACCTCCAGCCTTGCTCTTACCACCCTGTACATTCTCC
TGTACTTTCCAGTGACCCTCATCATAGGCCCAAGTGTGCAAAGCTTAGCTTTGTGGGTATCCCTTGGCTG
CTTTTCATTAAAGAAGTTTTCCTCTCAATTCTTTCCTGTCGCTTTGCAGCACCATCAGCACCAGGCCGCC
CAGGCTCTCCATCTGGCCAGTCCACAGCAGCAGTCAGCCATTTACCACGCGGGGCTTGCGCCAACTCCAC
CCTCCATGACACCTGCCTCCAACACGCAGTCGCCACAGAATAGTTTCCCAGCAGCACAACAGACTGTCTT
TACGATCCATCCTTCTCACGTTCAGCCGGCGTATACCAACCCACCCCACATGGCCCACGTACCTCAGGTA
ATACCAGCTTTAGCCAACTTTCTGTGAAGGCCAAGTAGAATGTGAAGGTTATCAGTAAGCAGCTAGAGGC
TCTCCCAGCTAGGAAACCCTGTGTGTCATGCCATTTGCCTGTCTCCCTTTCCCTCTCAAATACACGTGAT

Indeed constitutive exon 22, starting with V (GTT) instead of I (ATT) when exon 21 gets spliced out. Encodes first structured sequence, then intrinsically disordered domain 3 (IDR3, green letters), with residues ISTGSLAQQYAHPNATLHPHTPHPQPSATPTGQQQSQHGGSHPAPSPVQ

Indeed constitutive exon 23A, encoding first IDR3 second part (green letters), then structured sequence, with residues HHQHQAAQALHLASPQQQSAIYHAGLAPTPPSMTPASNTQSPQNSFPAAQQTVFTIHPSHVQPAYTNPPHMAHVPQ

CTGGCCCTAAGGGAATGTTTGTGTGGTTTTGTCATGGGATCAGTGAAGGTGCTGATTGGTCAGTCCTTTA
GTTTTCCAACTGAGACCTTAAAAATATCTTTGACTCTGGAATGCAACCCAGTCCTTCTTTCCTTTCTGTG
TCTGCTTTGCTATGTCTATATAGCCTCACTACTATATATATGTGTACATATATATTCCCCTACACACTTA
CCTTGGAAGCCAGGCAGGGATGATGGCCTTCACAGAGTCTCAGCTCTCCGAAGTGACTACCGGGGCCTGT
CAACTTGATTGTTACTCACATGAGTTCCAGACACATCTCTCCAATTGTTTTCCCTGGTTATCCATATATC
TGCTTTGACCATAAGTTGTACTCTTGAGAGGGCTTGGCCTTGGACATTGGTGCAGTGTAACTAGAAGCTG
GAAGCACCCAGGTGGTCCCATTTTTCTTTAAGAGCAGCCCTGGAAGCACTTTGGAGCTCACCTCCAGTGT
AAGCTGCTACAGGTGAAAGGTGTGCTTGCCATCTCAGTGGTTGCTGTCTGCATCAGCTGCTGACAAAGGT
CCCTGCACTCCAGGGCCCAGGGGATTGTCTTAATGAGGAGAAGGAGCTGCACTGAAGTTGGGCTCTAACG
CTGGCCTTGAGGCCCTCCCTGGGGCTGTTACGGGTGAATTGGCTGTATTAGATGTCTCTGCTACTTTCAT
AACAGAACTCTCTGAGGCGGGTCTAAGTGAGACCTGCCACAATGAATTCCATTTCCTGTTAAATAGTGCG
CCAGTGAGGCTCTGGCAAGGTGTGGGCTAGAGATGCGACTCAGTTGGATCTATCTCTCAGAAGGCTACCT
TGTAAGTAGAGTTCCACAGCTCTGGGAAGTTTGGGCGTCCTCACCCTGCAAAGTTTAGGTTCTGTGGTGT
AGCGCACTGCAGTTGATTTGCTTTTTGATAGTGGGGAGGGAAGCCGGTTTGGTCCGTGTGGGCCAGCGTG
GTTTGGTGGAGTCAGCTTCATAAGAGCTGGGGTCCTGTAGGTGTCTACCAGAGGCTGGTGGCTAAGTAGG
CATGTGAACTTACATGTAAGTCAGGGATCCCTAAAACCTCACTCTGTTTTTGTGCTGAAAGGGCAAAAAG
GTTAACACAGGGAAGCTCAAATTTGCCATGTGCCCGTTTGAATATGTGAGAGTAAAAACGGCATTTCATC
CAAGGCTTATCGTAGTCTAGAACAGTGCACAGTGTGGGAAAAAGGAAACAAGGGCTCTTCCTGGCCCTGC
CAACCCCCTGCAGAGCTGGAATCCAGCTGTTTGGGCTGACTAAAATCACCTTTCCAACTTGACAGTGAGT
GAGACCAGGTTGAACTTGGTACAGAGACGCTGGGCTGGCCCAGATGACTTCAGGTTACTCCTTTCCATCT
CACTGGAGCCATTAAAAACTCCAACTCCTCCTCCTCCTCCTGCTCCATCAGCATATCTCTGAGAGAGTCA
CGGGGGCCTAAGAGTCTCTTTTCACTGCCTGGTGAGCAGACCAGAAGCAGAGGGAGAGAGGCAAATGAAC
AGAGGTCCAAGTAATTCACATACTTGACTGTGACAGTCTCTGCTTATTAATGTAATCTGTTTTCCTATTT
GAAAGGGATGTTATCTGCAAAACTACCTCAGGCCCCACATGGCAGCCTGATTCTGAAGCATCATTGAATC
TTGTATGATATTAAGTTGAGAAAGCTGCCCTTGGATCCAGTGTCTAATCTTTGTGAAGATCTTACCCCAT
ACATAGAATACAATGATCAGAAATGTCAAGGGTTAGGACAGCACAGCCCTGACTTCTACCCAGGCTCACT
TGTTGCCTGCTCCCTGACCCTTGCAGGATCTGCCCAAAGGTGAAGCGCGTCTTCAGGTCAATAGATAATC
TACTAGAGATTGTCCCCAGAGAACAGAACTGGGCCCTGAGGCCCACCGTTGCCCTTTCCTGAGAGTCCCA
GCCCAGTGAAAGGAACACAGTTGACATGTTGTTGAAGCCGGAGATGTTGCCTGTATGCGTAAAAGAGCTC
TCTGTTTCAGGCTCATGTACAGTCAGGAATGGTTCCTTCTCATCCAACTGCCCATGCGCCAATGATGCTA
ATGACGACACAGCCACCCGGCGGTCCCCAGGCCGCCCTCGCTCAAAGTGCACTACAGCCCATTCCAGTCT
CGACAACAGCGCATTTCCCCTATATGACGCACCCTTCAGGTGAGGCGTGTGTGTGCAGGGGCCGCCGGGG
CACCCCAA**AGCATTCTGCTCGCACAGGTGGAATGG**CAGGCAGGGCCAGTGCTTCAAGCCCGCATTTGAG
AACTAGCAAGACCCGTCCAGGAGTGTGCACAGGAGGGACTGTGACGATCAGTTCAGCATCAGGGCCTGAG
GCTTCCGGGAGCCGAGTCTGTGTGTGTTCTGATGGTATACAGGATTTGGCTTGATGAGAAGCAGCAGCAG
CAGCAACAGCAGCCTGATGCATGCCTAGGACTCAGTTGGCCTTCCTTGTTATGACAGGCTGGACAGGGCA
GTGTTTTCCTTCCTGAGTCCCAAAAGTCTGACATGTGGGGGTTATTACCATGGCAGAGTTTGATTGTAG
CTCTGGAGAAGATACTGCTGAGAAAGCGCTGTGGATGGACTGGCTTTGAGTGTAGCGTTAGCCCCAGCCC
CTGAACAGGGGAGAGCGCCCTGTGATTGTGCTCTACTACTTGATGGCTGCCATGGCGATACTTCACAGTC

This AG sequence can be alternatively spliced in or out.

Alternatively spliced exon 23B, encoding structured sequence, with residues CASEALARCGLEMRLSWICLSEGYL

Alternatively spliced exon 24A, encoding structured sequence, with residues AHVQSGMVPSHPTAHAPMMLMTTQPPGGPQ AALAQSALQPIPVSTTAHFPYMTHPS missing for example in NCBI GenBank entries for chimpanzee XM_054663566.2, XM_054663567.2, XM_054663568.2, XM_054663570.1, XM_054663571.1, XM_063784959.1, XM_063784962.1

Alternatively spliced exon 24B, encoding structured sequence with an embedded intrinsically disordered region (green letters), with residues GEACVCRGRRGTPKHSARTGGMAGRASASSPH LRTSKTHPGVCTGGTVTISLASGPEASGSRVCVC SDGIQDLA
Upon frameshift +1 to alternative exon 24C, **BOLD** letters encode residues SILLAQVEW

TGACCTGTTATTCTGAAAGCAATACTGGTGCTTGGCTAATATTTGGGGAGGGGGTTTGTTAAGGCCTTTT
TTTCTACCCCATGAACAAGTCTTCTGGGAGTTTTATCTGAAGTGGTTTTACGTCTGACTGGTTTGTTTCT
ACCCACCCACCCAACCCTCCCCACTTTGGTGCAGATGGGAGGGGGAAAAGCGAATTCAATTTTGAGTTTT
GTTCAGCTAGCACGAGGATAGTTTACAATCATGTGCTGCAGAGACACTAGGCTGATGTGTGGTGTTGCCA
GTTTTCTGTTTCAATGTTCGCTTTTCTTTTTACAGTACAAGCCCACCACCAACAGCAGTTGTAAGGCTGC
CCTGGAGGAACCGAAAGGCCAAATTCCCTCCTCCCTTCTACTGCTTCTACCAACTGGAAGCACAGAAAAC
TAGAATTTCATTTATTTTGTTTTTAAAATATATATGTTGATTTCTTGTAACATCCAATAGGAATGCTAAC
AGTTCACTTGCAGTGGAAGATACTTGGACCGAGTAGAGGCATTTAGGAACTTGGGGGCTATTCCATAATT
CCATATGCTGTTTCAGAGTCCCGCAGGTACCCCAGCTCTGCTTGCCGAAACTGGAAGTTATTTATTTTTT
AATAACCCTTGAAAGTCATGAACACATCAGCTAGCAAAAGAAGTAACAAGAGTGATTCTTGCTGCTATTA
CTGCTAAAAAAAAAAAAAAAAAAAAATCAAGACTTGGAACGCCCTTTTACTAAACTTGACAAAGTTTCAG
TAAATTCTTACCGTCAAACTGACGGATTATTATTTATAAATCAAGTTTGATGAGGTGATCACTGTCTACA
GTGGTTCAACTTTTAAGTTAAGGGAAAAACTTTTACTTTGTAGATAATATAAAATAAAAACTTAAAAAAA
ATTTAAAAAATAAAAAAAGTTTTAAAAACTGA

Alternatively spliced exon 25, encoding structured sequence, with residues VQAHHQQQL.
Upon frameshift +1, the sequence encodes the frequent C-terminus YKPTTNSSCKAALEEPKGQIPSSLLLLLPTGSTEN e.g. in *Sciurus carolinensis* and *Rhinolophus sinicus*.
