# Supplementary material for: Bioinformatic Analyses of the Ataxin-2 Family Since Algae Emphasize Its Small Isoforms, Large Chimerisms, and the Importance of Human Exon 1B as Target of Therapies to Prevent Neurodegeneration
Source: Int J Mol Sci. 2026 Feb 3;27(3):1499. doi: 10.3390/ijms27031499 (PMC12898128; doi:10.3390/ijms27031499)
Supplement: Supplementary file 1 [file ijms-27-01499-s001.zip › AuburgerSen_SupplMaterialS5_HomoSapiensMultipleSequenceAlignment-ATXN2L-ATXN2-mATXN2-mATXN2L-GA.pdf]

```
exon #         1A
hATXN2         MRSAAAAPRSPAVA-TESRRFAAARWPGWRSLQRPARRSGRGGGGAAPGPYPSAAPPPPGP   60
exon #         2
mATXN2         MRSSTAAAQRPAAGDPEPRRP—-AGWAARRSLPRTARRGGRGGAVA----YPSAGPPPRGP  55
exon #
hATXN2L        ------------------------------------------------------------    0
exon # (analysis by genOway, Lyon)
mATXN2L        ------------------------------------------------------------    0


hATXN2         -GPPPSRQSSPPSASDCFGSNGNGGGAFRPGSRRLLGLGGPPRPFVVLLLPLASPGAPPA    119

mATXN2         -GAPPRGPRSPPCASDCFGSNGH—-GASRPGSRRLLGVCGPPRPFVVVLLPL----APAA   108

hATXN2L        ------------------------------------------------------------    0

mATXN2L        ------------------------------------------------------------    0


exon #                                                   1B
hATXN2         APTRASPLGARASPPRSGV-SLARPAPGCPRPACEPVYGPLTMSLKPQQQQQQQQQQQQQQ   179

mATXN2         TPARACPPGVRASPPRSGVSSSARPAPGCPRPACEPVYGPLTMSLKPQPQ-----------  158
exon #                                                   1
hATXN2L        ------------------------------------------MLKPQPLQQPS-------    11
                                                         1
mATXN2L                                                  MLKPQPPQQTS------    11


hATXN2         QQQQQQQQQPPPAAA--NVRKPGG--------SGLLASPAAAPSPSSSS------VSSSS    223

mATXN2         ----------PPAPA—-TGRKPGG---------GLLSSPGAAPA-SAAV------TSASV   190

hATXN2L        -----QPQQPPPTQQAVARRPPGGTSPPNGGLPGPLATSAAPPGPPAAASPCLGPVAAAG    66

mATXN2L        -----QPQQPPPTQQAVARRSPGGTSPPNGGLPGPLTATAAPPGPPAAVSPCLGPAAAAG   66
```

```
                                                              LSM domain
                                     2           3A      3B
hATXN2   -------------ATAPSSVVAATS---GGGRPGLGRGRNSNKGLPQSTISFDGIYANMRMVH   270
                                     5           6
mATXN2   -------------VPAPAAPVASSSAAAGGGRPGLGRGRNSSKGLPQPTISFDGIYANVRMVH   240
                                     2           3
hATXN2L  SGLRRGAEGILAPQPPPPQQHQERP---GAAAIGSARGQSTGKGPPQSP-VFEGVYNNSRMLH   125
                                     4B          5
mATXN2L  SGLRRGAESILAASAPP—-QHQERP---GAVAIGSVRGQTTGKGPPQSP-VFEGVYNNSRMLH   123


               4                        5
hATXN2   ILTSVVGSKCEVQVKNGGIYEGVFKTYSPKCDLVLDAAHEKSTESSSGPKREEIMESILF   330
               7                        8
mATXN2   ILTSVVGSKCEVQVKNGGIYEGVFKTYSPKCDLVLDAAHEKSTESSSGPKREEIMESVLF   300
               4                        5
hATXN2L  FLTAVVGSTCDVKVKNGTTYEGIFKTLSSKFELAVDAVHRKASEPAGGPRREDIVDTMVF   185
               6                        7
mATXN2L  FLTAVVGSTCDVKVKNGTTYEGIFKTLSSKFELAVDAVHRKASEPAGGPRREDIVDTMVF   183


                             6
hATXN2   KCSDFVVVQFKDMDSSYAKRDAFTDSAI--SAKVNGEHKEKDLEPWDAGELTANEELEAL   388
                             9
mATXN2   KCSDFVVVQFKDTDSSYARRDAFTDSAL-—SAKVNGEHKEKDLEPWDAGELTASEELE-L   357
                             6
hATXN2L  KPSDVMLVHFRNVDFNYATKDKFTDSAIAMNSKVNGEHKEKVLQRWEGGDSNS--DDYDL   243
                             8
mATXN2L  KPSDVLLVHFRNVDFNYATKDKFTDSAIAMNSKVNGEHKEKVLQRWEGGDSNS--DDYDL   241


                             LSMAD
             7                              8
hATXN2   ENDVSNGWDPNDMFRYNEENYGVVSTYDSSLSSYTVPLERDNSEEFLKREARANQLAEEI   448
             10                             11
mATXN2   ENDVSNGWDPNDMFRYNEENYGVVSTYDSSLSSYTVPLERDNSEEFLKREARANQLAEEI   417
             7                              8
hATXN2L  ESDMSNGWDPNEMFKFNEENYGVKTTYDSSLSSYTVPLEKDNSEEFRQRELRAAQLAREI   303
             9                              10a
mATXN2L  ESDMSNGWDPNEMFKFNEENYGVKTTYDSSLSSYTVPLEKDNSEEFRQRELRAAQLAREI   301
```

```
                                                    9
hATXN2     ESSAQYKARVALENDD-RSEEEKYTAVQRNSSEREGHSINTRENKYIPPGQRNREVISWG    507
                                                    12
mATXN2     ESSAQYKARVALENDD-RSEEEKYTAVQRNCSDREGHGPNTRDNKYIPPGQRNREVLSWG    476
                                                    9
hATXN2L    ESSPQYRLRIAMENDDGRTEEEKHSAVQRQGSGRESPSLASREGKYIPLPQRVREGPR-G    362
                                                    11
mATXN2L    ESSPQYRLRIAMENDDGRTEEEKHSAVQRQGSGRESPSLVSREGKYIPLPQRVREGPR-G    360


                                                   11
hATXN2     SGRQNSPRMGQPGSGSMPSRSTSHTSDFNPNSGSDQRVVNGGPPRMSPKAQRHPRNHRVS    567
                                                   14
mATXN2     SGRQSSPRMGQPGPGSMPSRAASHTSDFNPNAGSDQRVVNGGPPRMSPKAQRHPRNHRVS    536
                                                   10
hATXN2L    GVRCSSSRGGRPGLSSLPPRGPHHLDNSSPGPGSEARGINGGPSRMSPKAQRPLRGAKTL    422
           .                                       12b
mATXN2L    GVRCSSSRGGRPGLSSLPPRGPHHLDNSSPGPGSEARGINGGPSRMSPKAQRPLRGAKTL    420


                                                    12
hATXN2     AGRGSISSGLEFVSHNPPSEAATPPVARTSPSGGTWSSVVSG------VPRLSPKTHRPRSPRQNS    627
                                                    15b
mATXN2     AGRGSMSSGLEFVSHNPPSEAAAPPVARTSPAGGTWSSVVSG------VPRLSPKTHRPRSPRQSS    596
                                                    11
hATXN2L    SSPSNRPSGETSVPP-PPA-----------------------------VGRMY----PPRSPKSA-    467
                                                    13a
mATXN2L    SSPSNRPSGEASVPP-TSA-----------------------ALPFLPVGRMY----PPRSPKSA-    457


                                                  13
hATXN2     IGNTPSGPVLASPQAGIIPTEAVAMPIPAASPTPA----SPASNRAVTPSSEAKDSRLQD    683
                                                  16
mATXN2     IGNSPSGPVLASPQAGIIPAEAVSMPVPAASPTPA----SPASNRALTPSIEAKDSRLQD    652
                                                  12
hATXN2L    APAPISASCPEPPIGSAVPTSSASIPVTSSVSDPGVGSISPASPKISLAPTDVK-----E    514
                                                  14
mATXN2L    APAPVSASCPEPPIGSAV-ASSASIPVTSSVVDPGAGSISPASPKLSLTPTDVK-----E    511
```

14 15

hATXN2 QRQNSPAGNKENIKPNETSPSFSKAENKGISPVVSEHRKQIDDLKKFKNDFRLQPSSTSE 743

17 18b

mATXN2 QRQNSPAGSKENVKASETSPSFSKADNKGMSPVVSEHRKQIDDLKKFKNDFRLQPSSTSE 712

13 14

hATXN2L LSTKEP---GRTLEPQEL----ARIAGKVPGLQNEQKRFQLEELRKFGAQFKLQPSSSPE 567

15 16

mATXN2L LPTKEP---SRNLEAQEL----ARIAGKVPGLQNEQKRFQLEELRKFGAQFKLQPSSSPE 564

hATXN2 SMDQLLN-----KNREGEK-SR-DLIKDKIEPSAKDSFIENSS--SNCTSGSSKPNSPSISP 796

mATXN2 SMDQLLS-----KNREGEK-SR-DLIKDKTEASAKDSFIDSSSSSSNCTSGSSKTNSPSISP 767

hATXN2L NSLDPFPPRILKEEPKGKEKEVDGLLTSE--P--MGS-----PVSSKTESVSDKEDKPPLAP 620

mATXN2L TGLDPFPSRILKEEAKGKEKEVDGLLTSD--P--MGS---PVSSKTESILDKEDKVPMAG 624

PAM2 motif

16

hATXN2 SILSNTEHKRGPEVTS--QGVQTSSPACKQEKDDKEEKKDAAEQVR**KSTLNPNAKEFNPR** 854

19

mATXN2 SMLSNAEHKRGPEVTS--QGVQTSSPACKQEKDDREEKKDTTEQVR**KSTLNPNAKEFNPR** 825

15

hATXN2L SG-----GTEGPEQPPPPCPSQTGSPPVGLIKGEDKDEGPVAEQVK**KSTLNPNAKEFNPT** 675

17

mATXN2L VG-----GTEGPEQLPAPCPSQTGSPPVGLIKGDEKEEGPVTEQVK**KSTLNPNAKEFNPT** 672

17 18

hATXN2 **SF**S--QPKPSTTPTSPRPQAQPSPSMVG---HQQPTPVYTQPVCFAPNMMYPVPVSPGVQ-P- 910

20 21a

mATXN2 **SF**S--QPKPSTTPTSPRPQAQPSPSMVG---HQQPAPVYTQPVCFAPNMMYPVPVSPGVQ-P- 881

16 17

hATXN2L **KP**LLSVNKSTSTPTSPGPRTHSTPSIPVLTAGQSGLYSPQYISYIPQIHMGPA-----VQAPQ 733

18 19

mATXN2L **KP**LLSVNKSTSTPTSPGPRTHSTPSIPVLTAGQSGLYSPQYISYIPQIHMGPA-----VQAPQ 730

```
                    19
hATXN2     LYPIPMTPMPVNQAKTYRAGKVPNMPQQRQDQHH---QSAMMHPASAA-GPPIAATPPAYS   967
                    23b
mATXN2     LYPIPMTPMPVNQAKTYRAGKVPNMPQQRQDQHH---QSTMMHPASAA-GPPIVATPPAYS   938
                    18
hATXN2L    MYPYPVSNSVPGQQGKYRGAK-GSLPPQRSDQHQPASAPPMMQAAAAA-GPPLVAATP--Y   790
                    20b
mATXN2L    MYPYPVSNSVPGQQGKYRGAK-GSLPPQRSDQHQPASAPPMMQAAAAAAGPPLVAATP--Y   788

                          20
hATXN2     TQYVAYSPQQFPNQPLVQ-HVPHYQSQHPHVYSPVIQGNARMMAPPTHAQPGLVSSSATQ   1026
                          24b
mATXN2     TQYVAYSPQQFPNQPLVQ-HVPHYQSQHPHVYSPVIQGNARMMAPPAHAQPGLVSSSAAQ   997
                          19
hATXN2L    SSYIPYNPQQFPGQPAMMQPMAHYPSQ--PVFAPMLQSNPRMLTSGSHPQA-IVSSSTPQ   847
                          21b
mATXN2L    SSYIPYNPQQFPGQPAMMQPMAHYPSQ—-PVFAPMLQSNPRMLTSGSHPQA-IVSSSTPQ   845

                        22
hATXN2     YGAHEQTH--AMY ISTGSLAQQYAHPNATLHPHTPHPQPSATPTGQQQSQHGGSHPAPSPVQ  1086
                        27b
mATXN2     FGAHEQTH—-AMYAISTGSLAQQYAHPNAALHPHTPHPQPSATPTGQQQSQHGGSHPAPSPVQ  1075
                        20
hATXN2L    YPSAEQPTPQALY -ATVHQSYPHHATQLHAHQP--QPATTPTGSQP---QSQ--HAAPSPVQ  901
                        21c
mATXN2L    YPAAEQPTPQALY -ATVHQSYPHHATQLHGHQP—-QPATTPTGSQP---QSQ--HAAPSPVQ  899

           23
hATXN2     HHQHQAAQALHLASPQQQSAIYHAGL-APTPPSMTPASNTQSPQNSFPAAQQTVFTIH-PS   1145
           28b
mATXN2     HHQHQAAQALHLASPQQQSAIYHAGL-APTPPSMTPASNTQSPQSSFPAAQQTVFTIH-PS   1134
           21
hATXN2L    ---HQAGQAPHLGSGQPQQNLYHPGALTGTPPSLPPGPSAQSPQSSFPQPA-AVYAIH-HQ   957
           21d
mATXN2L    ---HQAGQAPHLGSGQPQQNLYHPGALTGTPPSLPPGPSAQSPQSSFPQPA-AVYAIHPHQ   956
```

```
                        24A
hATXN2     HVQPAYTNPPHMAHVPQAHVQSGMVPS---HPTAHAPM--MLMTT-QPPGGP-QAALAQS    1198
                        29
mATXN2     HVQPAYTTPPHMAHVPQAHVQSGMVPS---HPTAHAPM--MLMTT-QPPGGP-QAALAQS    1187
                        22A
hATXN2L    QLPHGFT---NMAHVTQAHVQTGITAAPPPHPGAPHPPQVMLLHPPQSHGGPPQGAVPQS    1014
                        22b
mATXN2L    QLPHGFT---NMAHVTQAHVQTGVTAAPPPHPGAPHPPQVMLLHPPQGHGGPPQGAVPPS    1013


                                24B
hATXN2     ALQPIPVSTTAHFPYMTHPSGEACVCR-GRRGTPSILLAQVEW    1240
                                30a
mATXN2     ALQPIPVSTTAHFPYMTHPSVQAHHQQQL                  1216
                                22B
hATXN2L    GVPALSASTPSPYPYIGHPQGEQPGQAPGFPGGADDRI-----    1052
                                22i
mATXN2L    GVPALSASTPSPYPYIGHPQVQSHPSQQLPFHPPGN           1049
```
