# Supplementary material for: Bioinformatic Analyses of the Ataxin-2 Family Since Algae Emphasize Its Small Isoforms, Large Chimerisms, and the Importance of Human Exon 1B as Target of Therapies to Prevent Neurodegeneration
Source: Int J Mol Sci. 2026 Feb 3;27(3):1499. doi: 10.3390/ijms27031499 (PMC12898128; doi:10.3390/ijms27031499)
Supplement: Supplementary file 1 [file ijms-27-01499-s001.zip › AuburgerSen_SupplMaterialS6_hATXN2exonExpressionSpinalCord_encodedResidues.pdf]

Human *ATXN2* expression of individual exons in spinal cord,
according to https://www.gtexportal.org/home/gene/ATXN2

GTEX exon count		differs from	Traditional exon number		# of encoded residues
Exon 1	111599554	0	--	not in UCSC	
Exon 2	111598784	0.0417	1B	with one M, then polyQ	84
Exon 3	111597916	0.0427	--	not in UCSC	
Exon 4	111597749	0	--	not in UCSC	
Exon 5	111555883	0.174	2	pre LSM	12
Exon 6	111554158	0.211	3A+3B	**LSMa** with two Met residues	20
Exon 7	111552906	0.252	4	**LSMb**	24
Exon 8	111552280	0.439	5	**LSMc** with Met	50
Exon 9	111525192	0.529	6	pre LSMAD	42
Exon 10	111520882	0.444	7	**LSMADa**	31
Exon 11	111519832	0.394	8	**LSMADb**	66
Exon 12	111518249	0.475	9	post LSMAD with two Met	59
Exon 13	111516154	0.407	10	alternatively spliced with two Met	12+70=82
Exon 14	111513357	0.512	11	with Met	61
Exon 15	111510385	0.548	12	with Met	66
Exon 16	111509891	0.573	13		36
Exon 17	111509549	0.550	14		24
Exon 18	111488476	0.644	15	with Met	102
Exon 19	111486761	0.879	16	**PAM2**	21
Exon 20	111485713	0.892	17	with three Met	51
Exon 21	111485265	0.861	18	with two Met	22
Exon 22	111482787	0.000	--	not in UCSC	
Exon 23	111479070	0.110	--	alternatively spliced sequence VKYLFLFIIHYV	12
Exon 24	111470558	0.926	19	with three Met	62
	111470108		20	with three Met	44
Exon 25	111468358	0.107	--	not in UCSC	
Exon 26	111464662	0.805	21	alternatively spliced as ACPKLPYNKETSPSFYFA	18
Exon 27	111461139	0.0393	--	not in UCSC	
Exon 28	111457214	1.48	22		49
	111456028		23	with two Met	76
	111453676		24A	with five Met	56+22=78
Exon 29	111453320	0.375	--	not in UCSC	
Exon 30	111452214	1.26	--	alternatively spliced sequence VQAHHQQQL*	9
Exon 31	111451947	0.00	--	not in UCSC	
Exon 32	111443485	0.0149	--	not in UCSC	
