# Supplementary material for: Bioinformatic Analyses of the Ataxin-2 Family Since Algae Emphasize Its Small Isoforms, Large Chimerisms, and the Importance of Human Exon 1B as Target of Therapies to Prevent Neurodegeneration
Source: Int J Mol Sci. 2026 Feb 3;27(3):1499. doi: 10.3390/ijms27031499 (PMC12898128; doi:10.3390/ijms27031499)
Supplement: Supplementary file 1 [file ijms-27-01499-s001.zip › AuburgerSen_SupplTableS3_AddedRNAprocessingDomains.pdf]

Table S3. List of Ataxin-2 orthologs with additional RNA quality control domain

| Database entry | Species | Family | Added domain | Function | PubMed-ID |
|---|---|---|---|---|---|
| A0A9W7Z4B9 | *Coemansia sp.* | fungi | RRM | RNA binding | 25120199, 39526332 |
| A0A4U0VMY8 | *Rhodotorula sp.* | fungi | HhH-GPD, like NTH1 | Oxidative nucleotide stress | 10706276, 8805338 |
| A0A2H9TMQ1 | *Paramicrosporidium saccamoebae* | fungi | Tetratricopeptide, SAM-dependent 23S rRNA methyltransferase | Spliceosome assembly, Oxidative nucleotide stress | 12509417, 20184321, 30704115 |
| KAF9164688.1 | *Actinomortierella ambigua* | fungi | 23S rRNA methyltransferase | Oxidative nucleotide stress | 20184321, 30704115 |
| A0A5C3F1S9 | *Pseudozyma flocculosa* | fungi | 28S rRNA (cytosine-C5)-methyltransferase | Oxidative nucleotide stress | 20184321, 30704115 |
| A0A4T0IZQ6 | *Wallemia ichthyophaga* | fungi | SAM RNA (C5-cytosine) methyltransferase | rRNA, tRNA and mRNA modification | 31522668, 10026269, 15121902 |
| A0A9W8LDP4 | *Coemansia interrupta* | fungi | Ribosomal protein L9N L9C | 23S rRNA binding | 8306963 |
| A0A9W8GBK0 | *Coemansia spiralis* | fungi | Ribosomal protein L9N L9C | 23S rRNA binding | 8306963 |
| A0A9W8CWC1 | *Coemansia biformis* | fungi | Ribosomal protein L9N L9C | 23S rRNA binding | 8306963 |
| A0A9W8H1X6 | *Coemansia pectinata* | fungi | Ribosomal protein L9N L9C | 23S rRNA binding | 8306963 |
| A0A074SQD8 | *Rhizoctonia solani 123E* | fungi | Ribonuclease inhibitor LRR repeat | Repression cytotoxicity RNase A | 12560499, 24941155, 7817399 |
| A0A8H8MCZ2 | *Ceratobasidium sp.* | fungi | Ribonuclease inhibitor LRR repeat | Repression cytotoxicity RNase A | 12560499, 24941155, 7817399 |
| A0AAV6I6Y5 | *Rhododendron griersonianum* | shrub | CBF-B/NF-YA transcription factor | RNA polymerase II regulation | 1549471, 1899284 |
| UPI001FC18B4E | *Certonardoa semiregularis* | sea stars | ACID domain, MED25 complex | RNA polymerase II regulation via RAR/RXR | 21785987, 17641689 |
| A0A6L2P9Q7 | *Coptotermes formosanus* | termites | DEAD box | RNA helicase | 38810698, 38594920 |
| A0A6H5ISX2 | *Trichogramma brassicae* | wasps | DEAD box | RNA helicase | 38810698, 38594920 |
| ROL51429.1 | *Anabarilius grahami* | bony fish | RIO2 kinase | rRNA processing | 31390939, 38324746 |
| XP_070807217.1 | *Pituophis catenifer* | snakes | BRAP2 with RRM, C3H2C3, UBP | Oxidative nucleotide stress | 9497340, 30297383 |
