# Supplementary material for: Bioinformatic Analyses of the Ataxin-2 Family Since Algae Emphasize Its Small Isoforms, Large Chimerisms, and the Importance of Human Exon 1B as Target of Therapies to Prevent Neurodegeneration
Source: Int J Mol Sci. 2026 Feb 3;27(3):1499. doi: 10.3390/ijms27031499 (PMC12898128; doi:10.3390/ijms27031499)
Supplement: Supplementary file 1 [file ijms-27-01499-s001.zip › AuburgerSen_SupplTableS4_AddedMembraneStressDomains_AlgaeToPlants.pdf]

Table S4. Ataxin-2 orthologs with added membrane stress domain in algae to plants

Database entry	Species	Family	Added domain	Function	PubMed-ID
A0A2P6U097	*Chlorella sorokiniana*	green algae	very-long-chain 3-ketoacyl-CoA synthase	adaptation of lipid membranes to abiotic stress	27803703, 32242934, 36864046
A0AAD5DQN1	*Chlorella ohadii*	green algae	very-long-chain 3-ketoacyl-CoA synthase	adaptation of lipid membranes to abiotic stress	27803703, 32242934, 36864046
V5NG01	*Auxenochlorella protothecoides*	green algae	very-long-chain 3-ketoacyl-CoA synthase	adaptation of lipid membranes to abiotic stress	27803703, 32242934, 36864046
A0A2P6V2R2	*Micractinium conductrix*	green algae	very-long-chain 3-ketoacyl-CoA synthase	adaptation of lipid membranes to abiotic stress	27803703, 32242934, 36864046
A0A813ABV5	*Symbiodinium necroappetens*	microalgae	SerA	L-serine homeostasis as precursor of sphingolipids and phospholipids	35565953, 9489019, 30013591
A0AA36E8E6, A0AA36E8L8	*Lactuca saligna*	lettuce	BRX	brassinosteroid biosynthesis	19037657
A0A4U5R0B4	*Populus alba*	poplar tree	LACT/PDAT	seed lipid droplet reserves	30252128, 37702708, 26883557
