# Supplementary material for: Bioinformatic Analyses of the Ataxin-2 Family Since Algae Emphasize Its Small Isoforms, Large Chimerisms, and the Importance of Human Exon 1B as Target of Therapies to Prevent Neurodegeneration
Source: Int J Mol Sci. 2026 Feb 3;27(3):1499. doi: 10.3390/ijms27031499 (PMC12898128; doi:10.3390/ijms27031499)
Supplement: Supplementary file 1 [file ijms-27-01499-s001.zip › AuburgerSen_SupplTableS5_AddedMembraneStressDomains_HexapodsToAnimals.pdf]

Table S5. Ataxin-2 orthologs with added membrane stress domain in hexapods to animals

Database entry	Species	Family	Added domain	Function	PubMed-ID
A0AAD9K567	*Paralvinella palmiformis*	worm	Ser/Thr hydratase	production of acetyl-CoA and lipids	40042273, 36468870
A0A226E5R7	*Folsomia candida*	hexapod springtail	oxysterol-binding	lipid transport, oxidative stress	39210909, 39915357
A0A1D2NII5	*Archesella cincta*	hexapod springtail	oxysterol-binding	lipid transport, oxidative stress	39210909, 39915357
A0A8J2NQ72, A0A8J2J4F7, A0A8J2NIC3	*Allacma fusca*	hexapod springtail	oxysterol-binding	lipid transport, oxidative stress	39210909, 39915357
A0A7R8YXR3	*Hermetia illucens*	fly	C2HC-type, MYT1-like	myelin transcription factor	24753812, 1280325, 14962745
A0A8S1C5C0, A0A8S1C908, A0A8S1C9E1	*Cloeon dipterum*	mayfly	Ser/Thr protein kinase, CamKK-like	cholesterol homeostasis and adiposity in human	21862616, 34845988, 40483692
A0A2A3ESF8	*Apis cerana*	bee	Apolipophorin-III	triacylglycerol recruitment from stores	25483322, 32209325
A0A4S2KN76	*Temnothorax longispinosus*	ant	Apolipophorin-III	triacylglycerol recruitment from stores	25483322, 32209325
KAF5904804.1	*Clarias magur*	bony fish	RALDH1 retinol dehydrogenase plus MAPKAP5	protects against lipid peroxidation, controls cPLA2 and mTORC1	21336308, 21666810, 25383140, 37298333
G5AKU3	*Heterocephalus glaber*	naked mole rat	sesquipedalian family pleckstrin homology	modulation of PtdIns4P levels	15107860, 28904204
