# Supplementary material for: Bioinformatic Analyses of the Ataxin-2 Family Since Algae Emphasize Its Small Isoforms, Large Chimerisms, and the Importance of Human Exon 1B as Target of Therapies to Prevent Neurodegeneration
Source: Int J Mol Sci. 2026 Feb 3;27(3):1499. doi: 10.3390/ijms27031499 (PMC12898128; doi:10.3390/ijms27031499)
Supplement: Supplementary file 1 [file ijms-27-01499-s001.zip › AuburgerSen_SupplTableS6_AddedMembraneStressDomains-Fungi.pdf]

Table S6. Ataxin-2 orthologs with added membrane stress domain in fungi

Database entry	Species	Family	Added domain	Function	PubMed-ID
A0A8H6QL38	*Aspergillus felis*	eurotiomycetes	ARM-like fold with CIK-related	orthologs SCYL1-3, involved in Golgi traffic regulation, MTORC1, diseases SCAR21 & ALS	17571074, 18556652, 20505071, 23175812, 26581903, 26981075, 29437892, 32583741, 35948564
KAJ9652119.1	*Neophaeococcomyces mojaviensis*	eurotiomycetes	KA1/Ssp2_C	AMPK sensor, exocytosis	9759505, 15182702, 15563607
A0AAI9WVV1	*Candida oxycetoniae*	pichiomycetes	SEC6	regulates polarized vesicular membrane transport in sterol-dependent manner	21819498, 25838123
A0A2V1AQ46	*Candidozyma haemuli*	pichiomycetes	SEC6	regulates polarized vesicular membrane transport in sterol-dependent manner	21819498, 25838123
A0A0E9NFC8	*Saitoella complicata*	taphrinomycetes	longin	controls membrane dynamics	31562761
A0A9N9AHJ9	*Racocetra fulgida*	glomeromycetes	cytochrome c oxidase, subunit Va/VI	stabilization of OXPHOS complex-IV	30598554
A0A9N8VJW7	*Diversispora eburnea*	glomeromycetes	calcineurin-like phosphoesterase	like sphingomyelin phosphodiesterases, for stress-induced ceramides	16981685
A0A261XY39	*Bifiguratus adelaidae*	mucoromycetes	cysteine desulfurase, with TPX2	like NFS1 acting in iron-sulfur proteins for lipid and sterol synthesis, assembly factor for spindles	18784075, 31395877, 31935115, 19208764
A0AAD5T663, A0AAD5XG15	*Physocladia obscura*	chytridiomycetes	phospholipid transporter P-type ATPase	phospholipid transport	37838176
A0A4T0FHH4	*Wallemia hederae*	basidiomycetes	alpha-aminoadipate reductase	lysine biosynthesis	16943623
G7E1R3	*Mixia osmundae*	mixiomycetes	chalcone isomerase	protects from excess UV light	10966651, 36739946, 40841532
