# Supplementary figures and images for: Bioinformatic Analyses of the Ataxin-2 Family Since Algae Emphasize Its Small Isoforms, Large Chimerisms, and the Importance of Human Exon 1B as Target of Therapies to Prevent Neurodegeneration
Source: Int J Mol Sci. 2026 Feb 3;27(3):1499. doi: 10.3390/ijms27031499 (PMC12898128; doi:10.3390/ijms27031499)

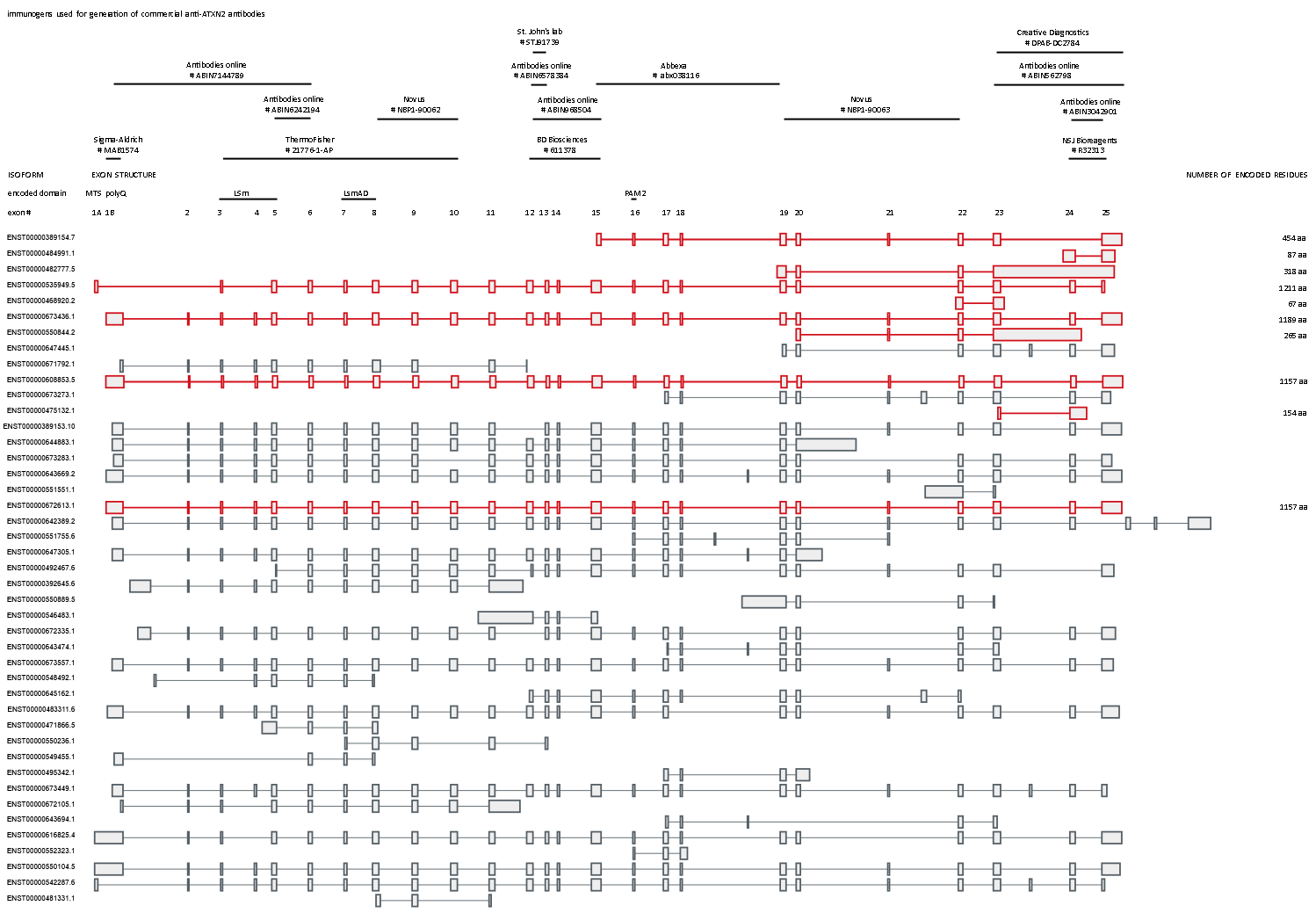

Supplement: Supplementary file 1 [file ijms-27-01499-s001.zip › AuburgerSen_FigureS1_GTex-IsoformsSpinocerebellarTissue.tif]
